# Development of diverse adjustable axially chiral biphenyl ligands and catalysts

**DOI:** 10.1016/j.isci.2023.106344

**Published:** 2023-03-07

**Authors:** Jiyang Jie, Haijun Yang, Yufen Zhao, Hua Fu

**Affiliations:** 1 Key Laboratory of Bioorganic Phosphorus Chemistry and Chemical Biology (Ministry of Education), Department of Chemistry, Tsinghua University, Beijing, China

**Keywords:** Organic synthesis, Stereochemistry, Organic chemistry methods

## Abstract

Development of highly efficient and practical chiral ligands and catalysts is an eternal theme in asymmetric synthesis. Here, we report the design, synthesis, and evaluation of a new kind of adjustable axially chiral biphenyl ligands and catalysts, in which six model reactions including asymmetric additions of diethylzinc or alkynes to aldehydes in the presence of axially chiral [1,1′-biphenyl]-2,2′-diol ligands, palladium-catalyzed asymmetric cycloadditions in the presence of phosphoramidite ligands, and chiral phosphoric acid-catalyzed asymmetric synthesis of 1,1′-spirobiindane-7,7′-diol derivative and [4 + 3] cyclization were attempted. The results showed that variation of 2,2′-substituent groups could provide different types of ligands and catalysts, and adjustment of substituent groups at the 3,3′, 5,5′, 6,6′-positions could make ligands and catalysts more efficient in the asymmetric catalytic synthesis. Therefore, our present research should provide a new and useful strategy for development of diverse axially chiral ligands and catalysts.

## Highlights

• Rational design and synthesis of ligands and catalysts
• Wide diversity of ligands and catalysts
• Adjustable axially chiral ligands and catalysts
• The present research provide diverse adjustable axially chiral ligands and catalysts

## Graphical abstract

**Figure:**
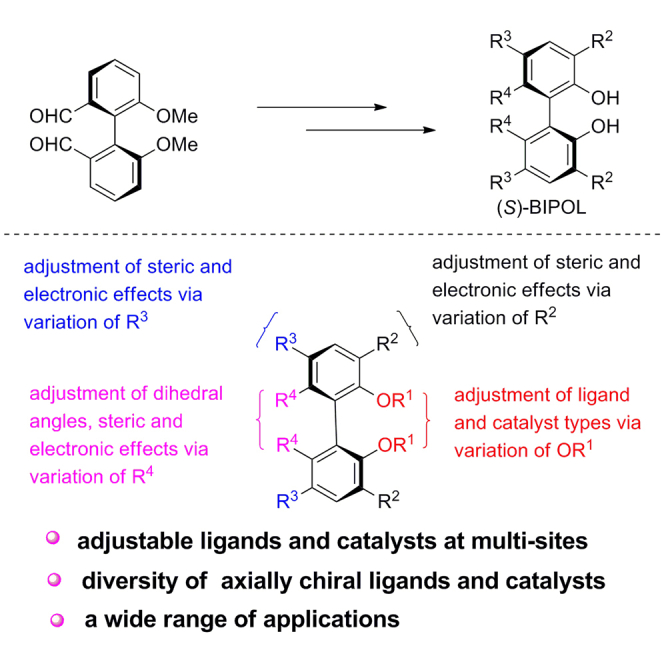

## Introduction

**Figure 1:**
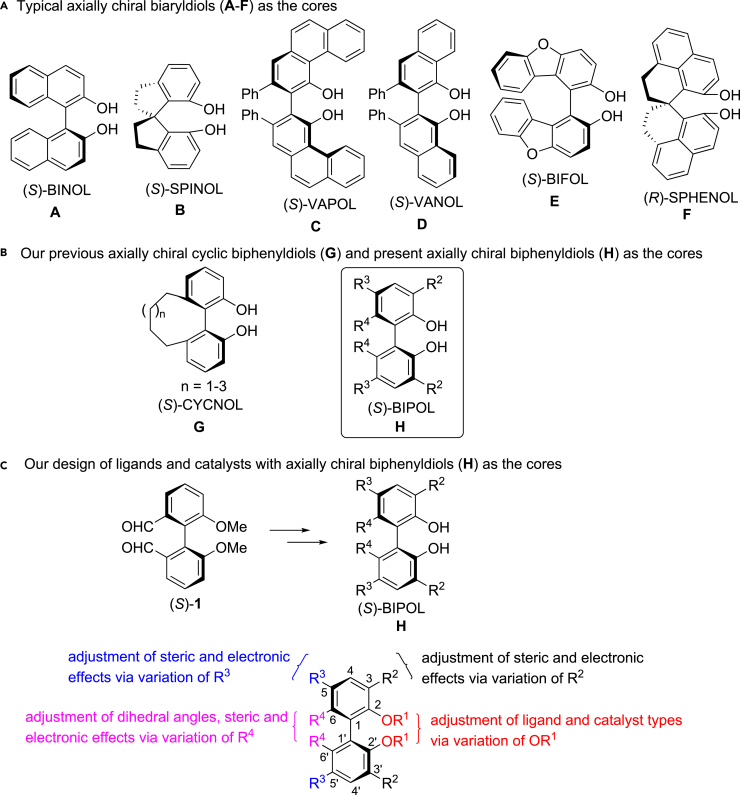
Axially chiral biaryldiol cores (A) Previous typical biaryldiols. (B) Our previous cyclic biphenyldiols and present adjustable biphenyldiols. (C) Our design of ligands and catalysts based on axially chiral biphenyldiols.

Chiral compounds widely occur in various fields, and they are often found in biomolecules, natural products, and drugs.^1^^,^^2^^,^^3^ Asymmetric chemical synthesis is an effective strategy for obtaining chiral molecules, and their efficiency highly depends on the chiral ligands^4^^,^^5^^,^^6^^,^^7^ and catalysts.^8^^,^^9^^,^^10^^,^^11^ Therefore, development of chiral ligands and catalysts is crucial in asymmetric synthesis. In the past decades, various chiral ligands and catalysts have been developed,^12^^,^^13^^,^^14^^,^^15^ in which the axially chiral ligands and catalysts have found widespread applications in areas of asymmetric synthesis. The representative axially chiral cores should be [1,1′-naphenyl]-2,2′-diol (BINOL) (**A**),^16^ 1,1′-spirobiindane-7,7′-diol (SPINOL) (**B**),^17^ VANOL (**C**), VAPOL (**D**),^18^ BIFOL (**E**)^19^ and SPHENOL (**F**)^20^ (Figure 1A). For example, BINOL^8^^,^^21^^,^^22^^,^^23^^,^^24^^,^^25^ and SPINOL^26^^,^^27^ derivatives have been extensively evaluated as the useful chiral ligands and catalysts in asymmetric synthesis. However, any ligand and catalyst has a limited range of applications because the slight changes in geometric, steric, and electronic properties of chiral ligands or catalysts can cause dramatic variations of reactivity of substrates and enantioselectivity of products. Recently, we have developed a new kind of axially chiral cyclo-[1,1′-biphenyl]-2,2′-diol (CYCNOL) cores (**G**) with adjustable dihedral angles by varying the chain length of the full-carbon 6,6′-tether (left one in Figure 1B),^28^ and CYCNOL-based phosphoramidite^29^^,^^30^^,^^31^^,^^32^ and diphosphine^33^ ligands and chiral phosphoric acid catalysts^34^ were effectively developed. Here, we design a novel kind of adjustable axially chiral [1,1′-biphenyl]-2,2′-diol (BIPOL) cores (**H**) (right one in Figure 1B). As shown in Figure 1C, BIPOL cores (**H**) can be prepared by using (*S*)-6,6′-dimethyl-[1,1′-biphenyl]-2,2′-diol ((*S*)-**L1**) as the material. Our imaginations are as follows: Variation of 2,2′-substituent groups would adjust types of chiral ligands and catalysts, variation of substituent groups at the 3,3′, 5,5′-positions would adjust steric and electronic properties of chiral ligands and catalysts, and variation of 6,6′-substituent groups would adjust dihedral angles, steric and electronic properties of chiral ligands and catalysts.

## Results and discussion

### Synthesis of diverse axially chiral (*S*)-biphenyldiols and crystal structures of representative axially chiral (*S*)-biphenyldiols

With our design above in hand, we first made the diverse biphenyldiols Supplemental information.

#### Synthesis of four axially chiral (*S*)-biphenyldiol cores

**Figure 2:**
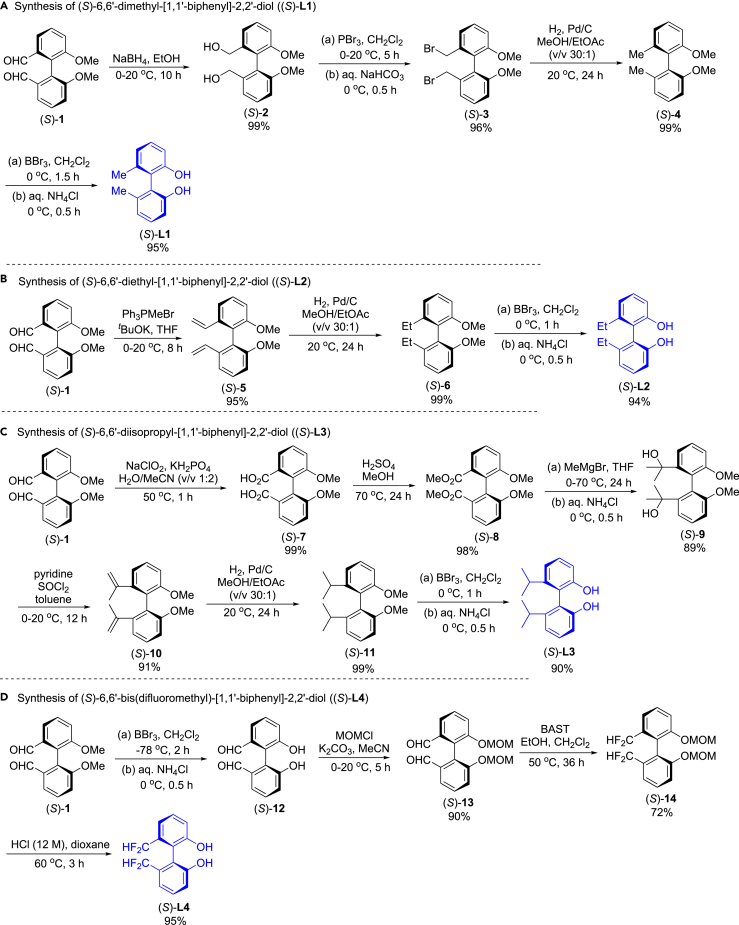
Synthesis of four axially chiral biphenyldiol cores, (*S*)-L1, (*S*)-L2, (*S*)-L3 and (*S*)-L4 (A) Synthesis of (*S*)-**L1**. (B) Synthesis of (*S*)-**L2**. (C) Synthesis of (*S*)-**L3**. (D) Synthesis of (*S*)-**L4**.

Synthesis^35^ and resolution^36^ of racemic 6,6′-dimethoxybiphenyl-2,2′-dicarbaldehyde (*Rac*-**1**) were performed according to the previous procedures. At first, (*S*)-6,6′-dimethyl-[1,1′-biphenyl]-2,2′-diol ((*S*)-**L1**) was prepared. As shown in Figure 2A, reduction of (*S*)-**1** in ethanol with NaBH_4_ provided the corresponding diol (*S*)-**2** in 99% yield, and bromination of (*S*)-**2** with phosphorus tribromide in anhydrous dichloromethane (CH_2_Cl_2_) led to (*S*)-**3** in 96% yield. Hydrogenation with Pd/C at atmospheric pressure provided (*S*)-**4** in 99% yield, and demethylation of the two ethers in (*S*)-**4** with BBr_3_ in CH_2_Cl_2_ followed hydrolysis afforded (*S*)-**L1** in 95% yield.

Subsequently, synthesis of (*S*)-6,6′-diethyl-[1,1′-biphenyl]-2,2′-diol ((*S*)-**L2**) was performed (Figure 2B). The Wittig coupling of Ph_3_P^+^MeBr^−^ with (*S*)-**1** in dry THF in the presence of ^*t*^BuOK provided (*S*)-**5** in 95% yield, reduction of (*S*)-**5** with hydrogen in the presence of Pd/C at atmospheric pressure gave (*S*)-**6** in 99% yield, and demethylation of the two ethers in (*S*)-**6** with BBr_3_ in CH_2_Cl_2_ followed hydrolysis led to (*S*)-**L2** in 94% yield.

Next, we prepared (*S*)-6,6′-diisopropyl-[1,1′-biphenyl]-2,2′-diol ((*S*)-**L3**). As shown in Figure 2C, oxidation of (*S*)-**1** with NaClO_2_ in the presence of NaH_2_PO_4_ in mixed solvent of H_2_O and MeCN produced the corresponding dicarboxylic acid (*S*)-**7** in 99% yield, methyl esterification of (*S*)-**7** provided (*S*)-**8** in 98% yield, and reaction of (*S*)-**8** with 10 equiv of MeMgBr gave (*S*)-**9** in 89% yield. Dehydration of (*S*)-**9** in the presence of SOCl_2_ and pyridine afforded (*S*)-**10** in 91% yield, hydrogenation with Pd/C at atmospheric pressure of (*S*)-**10** provided (*S*)-**11** in 99% yield, and demethylation of the two ethers in (*S*)-**11** with BBr_3_ in CH_2_Cl_2_ followed hydrolysis gave (*S*)-**L3** in 90% yield.

Finally, (*S*)-6,6′-bis(difluoromethyl)-[1,1′-biphenyl]-2,2′-diol ((*S*)-**L4**) was prepared (Figure 2D). Demethylation of (*S*)-**1** with BBr_3_ in CH_2_Cl_2_ followed hydrolysis gave (*S*)-**12**, then etherification of (*S*)-**12** with MeOCH_2_Cl (MOM-Cl) in the presence of K_2_CO_3_ led to (*S*)-**13** in 90% yield for the two step reactions, and the dialdehyde was transformed into the corresponding gem-difluorides (*S*)-**14**) with bis(2-methoxyethyl)aminosulfur trifluoride (BAST)^37^ in 72% yield. Desorption of MOM-protecting group with 12 M HCl in dioxane provided (*S*)-**L4** in 95% yield.

### Synthesis of axially chiral (*S*)-5,5′-dihalo-biphenyldiols

**Figure 3:**
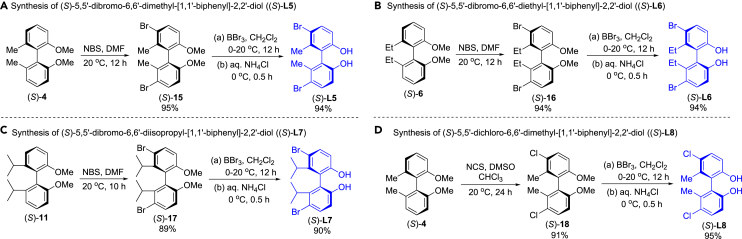
Synthesis of axially chiral (*S*)-5,5'-dihalo-biphenyldiols, (*S*)-L5, (*S*)-L6, (*S*)-L7 and (*S*)-L8 (A) Synthesis of (*S*)-**L5**. (B) Synthesis of (*S*)-**L6**. (C) Synthesis of (*S*)-**L7**. (D) Synthesis of (*S*)-**L8**.

Subsequently, we investigated synthesis of 5,5′-dihalo**-**biphenyldiols, (*S*)-**L5**, (*S*)-**L6**, (*S*)-**L7** and (*S*)-**L8** (Figure 3). Brominations of (*S*)-**4**, (*S*)-**6** and (*S*)-**11** with *N*-bromosuccinimide (NBS) in DMF gave (*S*)-**15**, (*S*)-**16**, (*S*)-**17** in 95%, 94%, 89% yields, respectively, and their demethylations with BBr_3_ in CH_2_Cl_2_ followed hydrolysis provided (*S*)-**L5**, (*S*)-**L6**, (*S*)-**L7** in 94%, 94%, 90% yields, respectively. Chlorination of (*S*)-**4** with *N*-chlorosuccinimide (NCS) in CHCl_3_ formed (*S*)-**18** in 91% yield, and demethylation of (*S*)-**18** with BBr_3_ in CH_2_Cl_2_ followed hydrolysis led to (*S*)-**L8** in 95% yield.

### Synthesis of axially chiral (*S*)-5,5′-substituted biphenyldiols

**Figure 4:**
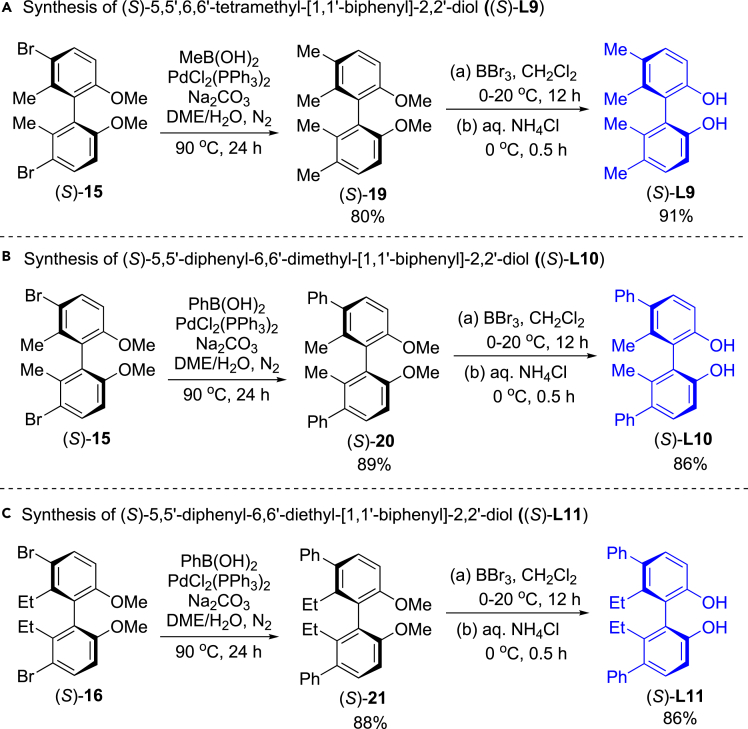
Synthesis of (*S*)-5,5′-substituted biphenyldiols (*S*)-L9, (*S*)-L10 and (*S*)-L11 (A) Synthesis of (*S*)-**L9**. (B) Synthesis of (*S*)-**L10**. (C) Synthesis of (*S*)-**L11**.

Next, we investigated synthesis of 5,5′-dimethyl or 5,5′-diphenyl biphenyldiols, (*S*)-**L9**, (*S*)-**L10** and (*S*)-**L11** (Figure 4). The Suzuki couplings of (*S*)-**15**, (*S*)-**16** with MeB(OH)_2_ or PhB(OH)_2_ led to the corresponding methylating or phenylating products (*S*)-**19**, (*S*)-**20** and (*S*)-**21** in 80%, 89% and 88% yields, respectively. Demethylation of the two ethers in (*S*)-**19**, (*S*)-**20** and (*S*)-**21** with BBr_3_ in CH_2_Cl_2_ followed hydrolysis provided (*S*)-**L9**, (*S*)-**L10** and (*S*)-**L11** in 91%, 86% and 86% yields, respectively.

### Crystal structures of representative axially chiral (*S*)-biphenyldiols

**Figure 5:**
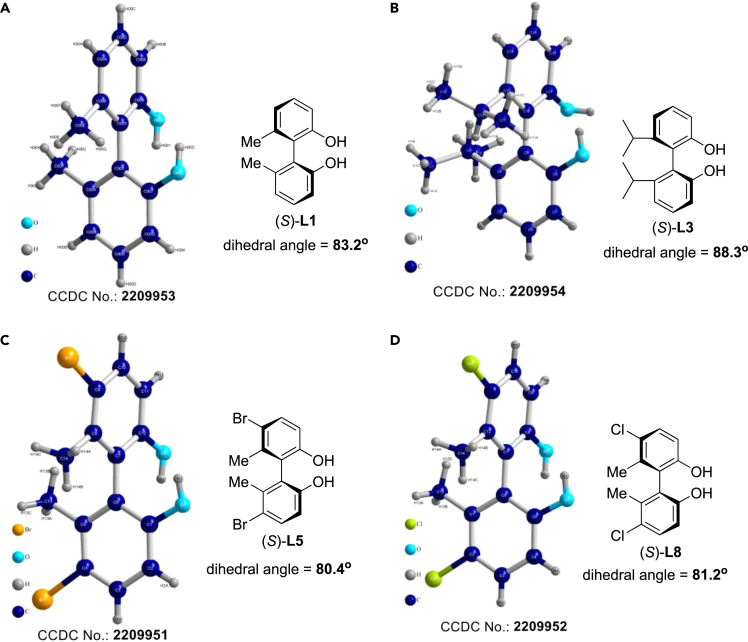
Crystal structures of (*S*)-L1, (*S*)-L3, (*S*)-L5 and (*S*)-L8 (A) Crystal structure of (*S*)-**L1** (Reference number: CCDC 2209953). (B) Crystal structure of (*S*)-**L3** (Reference number: CCDC 2209954). (C) Crystal structure of (*S*)-**L5** (Reference number: CCDC 2209951). (D) Crystal structure of (*S*)-**L8** (Reference number: CCDC 2209952).

Several representative biphenyldiols, (*S*)-**L1**, (*S*)-**L3,** (*S*)**-L5** and (*S*)-**L8**, were chosen as the examples, their single crystals from mixed solvent of hexane and diethyl ether were prepared, and the corresponding structures were unambiguously confirmed by X-ray diffraction analysis (Figure 5) (see Supplemental information for details). According to their X-ray diffraction data, dihedral angles of (*S*)-**L1**, (*S*)-**L3,** (*S*)**-L5** and (*S*)-**L8** are 83.2°, 88.3°, 80.4° and 81.2°, respectively. The results show that introduction of substituents with bigger steric hindrance at 6,6′-positions leads to bigger dihedral angles. Of interest, introduction of halos at 5,5′-positions makes dihedral angles of the axially chiral biphenyldiols become smaller. It is well known that the dihedral angles for the axially chiral ligands and catalysts are a key factor for reactivity of substrates and enantioselectivity of products in asymmetric synthesis.

### Evaluation of BIPOL-based axially chiral ligands and catalysts

Subsequently, we chose six model reactions to evaluate reactivity and enantioselectivity in asymmetric catalysis in the presence of BIPOL-based axially chiral ligands or catalysts including additions of diethylzinc or alkynes to aldehydes in the presence of axially chiral [1,1′-biphenyl]-2,2′-diol ligands, Pd-catalyzed asymmetric cycloadditions in the presence of phosphoramidite ligands, and chiral phosphoric acid-catalyzed asymmetric synthesis of 1,1′-spirobiindane-7,7′-diol (SPINOL) derivative and [4 + 3] cyclization.

### Addition of diethylzinc to aldehydes in the presence of chiral biphenyldiols

**Figure 6:**
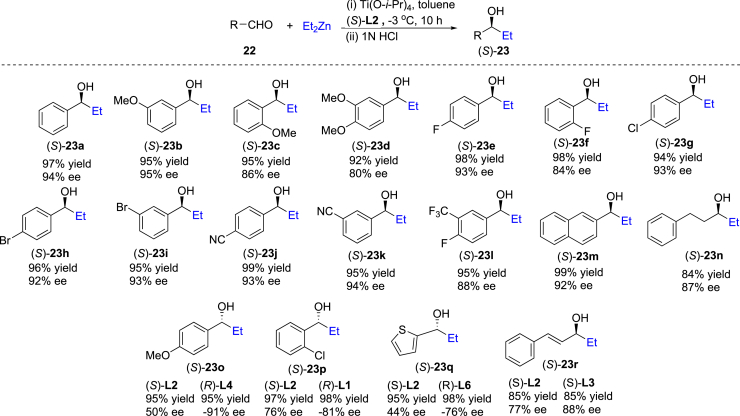
Enantioselective addition of diethylzinc to different aldehydes (22) leading to 23 Conditions: **22**:ZnEt_2_:Ti(O^*i*^Pr)_4_:ligand = 1:1.6:3:0.1 (molar ratio), aldehyde (**22**) (0.2 mmol), ZnEt_2_ (0.6 mmol), Ti(O^*i*^Pr)_4_ (0.32 mmol), lignad (0.02 mmol), toluene (2.0 mL), temperature (−3°C), time (10 h), 1N HCl (2.0 mL). Absolute configurations known, determined or assigned by analogy (see Supplemental information). Isolated yield. ee values determined by HPLC analysis (see Supplemental information).

It is well known that the enantioselective addition of diethylzinc to aldehydes with biaryldiol ligands is a standard reaction to evaluate the reactivity and enantioselectivity of newly developed chiral ligands.^38^^,^^39^^,^^40^^,^^41^ We chose addition of diethylzinc to benzaldehyde (**22a**) as the model reaction to test ligands including our newly developed (*S*)-**L1** ∼ (*S*)-**L11** and previous (*S*)-BINOL using titanium tetraisopropoxide as the Lewis acid, dry dichloromethane (CH_2_Cl_2_) as the solvent at −3°C or room temperature. As shown in Table 1, every ligand showed high reactivity, but the enantioselectivity was different (entries 1–12), and (*S*)-**L2** (entry 2), (*S*)-**L5** (entry 5) and (*S*)-**L8** (entry 8) provided higher ee values (>90% ee). Effect of solvents was investigated (13–17), and toluene was the best solvent for the reaction (entry 13). Subsequently, the substrate scope on the enantioselective addition of diethylzinc to aldehydes (**22**) was investigated. As shown in Figure 6, most of the tested aldehydes afforded excellent yields and good to excellent ee values. An aliphatic aldehyde, 3-phenylpropanal was used as the substrate, and it gave 84% yield and 87% ee (see (*S*)-23n). We found that several aldehydes provided poor ee values with (*S*)-**L2** as the ligand, for example, 50% ee was afforded for 4-methoxybenzaldehyde. When (*R*)-**L4** replaced (*S*)-**L2** as the ligand, higher enantioselectivity (−91% ee) was observed (see (*S*)-**23o**). Similarly, ligands (*R*)-**L1**, (*R*)-**L6** and (*S*)-**L3** instead of (*S*)-**L2** were used in the addition reactions of diethylzinc to 2-chlorobenzaldehyde, thiophene-2-carbaldehyde and cinnamaldehyde, respectively, and the corresponding ee values were obviously improved (see (*S*)-**23p**, **23q** and **23r**). The results show that our diverse adjustable chiral biphenyldiol ligands are very useful for enantioselective regulation of different substrates. The reaction above can tolerate various functional groups including ether, cyano, CF_3_, C-F, C-Cl, C-Br bonds and *S*-heterocycle.

**Table 1:** Optimization of conditions on enantioselective addition of diethylzinc to benzaldehyde (22a)^a^


Entry	Ligand	Solvent	Temp	yield (%)^b^	ee (%)^c^
1	(*S*)-**L1**	CH_2_Cl_2_	−3°C	97	86
2	(*S*)-**L2**	CH_2_Cl_2_	−3°C	97	93
3	(*S*)-**L3**	CH_2_Cl_2_	rt	96	84
4	(*S*)-**L4**	CH_2_Cl_2_	−3°C	93	81
5	(*S*)-**L5**	CH_2_Cl_2_	−3°C	97	93
6	(*S*)-**L6**	CH_2_Cl_2_	−3°C	90	83
7	(*S*)-**L7**	CH_2_Cl_2_	rt	91	80
8	(*S*)-**L8**	CH_2_Cl_2_	−3°C	95	91
9	(*S*)-**L9**	CH_2_Cl_2_	rt	92	85
10	(*S*)-**L10**	CH_2_Cl_2_	rt	91	81
11	(*S*)-**L11**	CH_2_Cl_2_	rt	89	83
12	(*S*)-BINOL	CH_2_Cl_2_	−3°C	95	86
**13**	(*S*)-**L2**	**Toluene**	**−3**°**C**	**97**	**94**
14	(*S*)-**L2**	THF	−3°C	75	42
15	(*S*)-**L2**	Et_2_O	−3°C	97	92
16	(*S*)-**L5**	Toluene	−3°C	96	91
17	(*S*)-**L5**	Et_2_O	−3°C	92	92

a Conditions: **22a**:Et_2_Zn:Ti(O^*i*^Pr)_4_:ligand = 1:3:1.6:0.1 (molar ratio), **22a** (0.2 mmol), ZnEt_2_ (0.6 mmol), Ti(O^*i*^Pr)_4_ (0.32 mmol), ligand (0.02 mmol), solvent (2.0 mL), temperature (-3°C or rt), time (10 h), 1N HCl (2.0 mL).

b Isolated yield.

c Absolute configurations known, determined or assigned by analogy (see Supplemental information), ee values determined by HPLC analysis using a chiral stationary phase (Chiralpak OD-H column) (*n*-hexane: *i*-PrOH= 96:4).

### Addition of alkynes to aldehydes in the presence of ZnMe_2_ and chiral biphenyldiols

**Figure 7:**
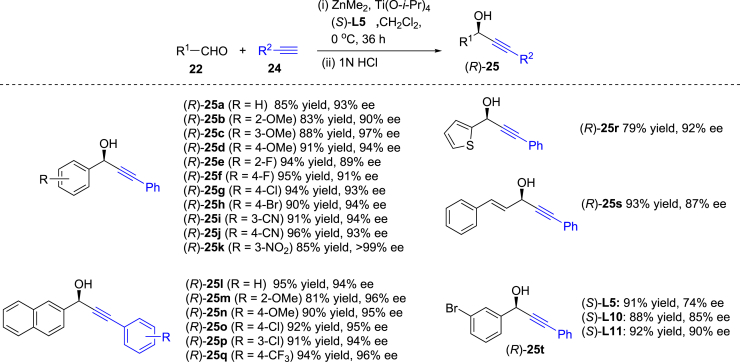
Enantioselective addition of alkynes (24) to aldehydes (22) leading to 25 Conditions: **22** (0.5 mmol), **24** (1.25 mmol), ZnMe_2_ (1.0 mmol), Ti(O^*i*^Pr)_4_ (0.125 mmol), ligand (0.1 mmol), solvent (2.0 mL), temperature (0°C), time (36 h), 1N HCl (2.0 mL). Isolated yield. Absolute configurations known, determined or assigned by analogy (see Supplemental information), and ee values determined by HPLC analysis using a chiral stationary phase (see Supplemental information).

To further explore application of our diverse adjustable chiral biphenyldiol ligands, we investigated Ti(O-*i*-Pr)_4_-catalyzed addition of alkynes to aldehydes in the presence of ZnMe_2_. Chiral propargylic alcohols are versatile synthons in organic chemistry, and catalytic asymmetric alkynylzinc addition to aldehydes can provide various chiral propargylic alcohols with high enantioselectivity.^42^^,^^43^^,^^44^^,^^45^ As shown in Table 2, Ti(O-*i*-Pr)_4_-catalyzed addition of phenylacetylene (**24a**) to benzaldehyde (**22a**) was selected as the model reaction to test our ligands and (*R*)-BINOL in the presence of ZnMe_2_ using dry dichloromethane (CH_2_Cl_2_) as the solvent at 0°C (entries 1–12)), and (*S*)-**L5** provided higher yield (85%) and highest ee value (93% ee) (entry 5). Other solvents, toluene, diethyl ether and THF, were attempted (entries 13–15), and they were inferior to DCM (entry 5). Subsequently, we surveyed substrate scope on the enantioselective addition of alkynes (**25**) to aldehydes (**22**). As shown in Figure 7, most of the tested aldehydes (**22**) and alkynes (**25**) afforded high yields and good to excellent ee values. During our screening, we found that addition of phenylacetylene (**24a**) to 3-bromobenzaldehyde only afforded 74% ee with (*S*)-**L5** as the ligand. When (*S*)-**L10** and (*S*)-**L11** instead of (*S*)-**L5** were used as the ligands, and 85% ee and 90% ee were obtained, respectively (see (*R*)-**25t**). The results also indicate that our diverse adjustable chiral biphenyldiol ligands are very useful for enantioselective regulation of different substrates. The reaction above can tolerate various functional groups including ether, cyano, CF_3_, NO_2_, C-F, C-Cl, C-Br bonds and *S*-heterocycle.

**Table 2:** Optimization of conditions on enantioselective addition of phenylacetylene (24a) to benzaldehyde (22a)^a^


Entry	Ligand	Solvent	Yield (%)^b^	ee (%)^c^
1	(*S*)-**L1**	CH_2_Cl_2_	82	81
2	(*S*)-**L2**	CH_2_Cl_2_	82	91
3	(*S*)-**L3**	CH_2_Cl_2_	89	79
4	(*S*)-**L4**	CH_2_Cl_2_	89	57
**5**	(*S*)-**L5**	**CH**_**2**_**Cl**_**2**_	**85**	**93**
6	(*S*)-**L6**	CH_2_Cl_2_	84	84
7	(*S*)-**L7**	CH_2_Cl_2_	87	60
8	(*S*)-**L8**	CH_2_Cl_2_	81	91
9	(*S*)-**L9**	CH_2_Cl_2_	88	28
10	(*S*)-**L10**	CH_2_Cl_2_	80	10
11	(*S*)-**L11**	CH_2_Cl_2_	86	9
12	(*R*)-BINOL	CH_2_Cl_2_	82	−90
13	(*S*)-**L5**	toluene	84	84
14	(*S*)-**L5**	THF	81	29
15	(*S*)-**L5**	Et_2_O	77	91

a Conditions: **22a** (0.2 mmol), **24a** (0.5 mmol), ZnMe_2_ (0.4 mmol), Ti(O^*i*^Pr)_4_ (0.05 mmol), ligand (0.04 mmol), solvent (2.0 mL), temperature (0°C), time (36 h), 1N HCl (2.0 mL).

b Isolated yield.

c Absolute configurations known, determined or assigned by analogy (see Supplemental information), and ee values determined by HPLC analysis using a chiral stationary phase (Chiralpak OD-H) (*n*-hexane: *i*-PrOH= 90:10).

### Pd-catalyzed asymmetric cycloadditions in the presence of chiral phosphoramidites

**Figure 8:**
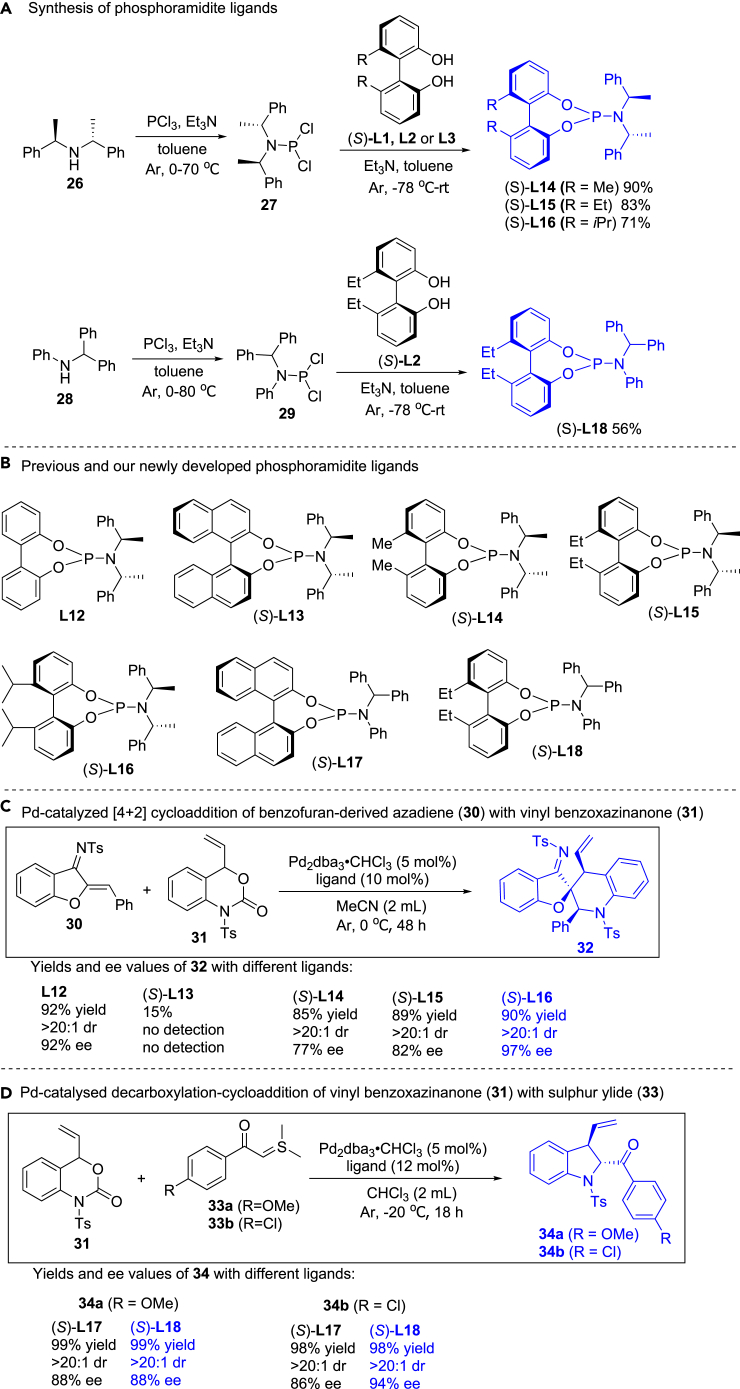
Synthesis and applications of phosphoramidite ligands (A) Synthesis of phosphoramidite ligands. (B) Previous and our newly developed phosphoramidite ligands. (C) Pd-catalyzed [4 + 2] cycloaddition of benzofuran-derived azadiene (**30**) with vinyl benzoxazinanone (**31**). (D) Pd-catalysed decarboxylation-cycloaddition of vinyl benzoxazinanone (**31**) with sulfur ylide (**33**).

Next, we investigated derivatization of our newly developed axially chiral biphenyldiol cores. It is well known that the phosphoramidites of axially chiral biaryldiols are the privileged ligands in the asymmetric synthesis.^46^^,^^47^^,^^48^^,^^49^^,^^50^^,^^51^ At first, we prepared the BIPOL-derived phosphoramidite ligands according to previous procedures.^46^^,^^47^^,^^48^^,^^49^^,^^50^^,^^51^ As shown in Figure 8A, reaction of chiral secondary amine **26** with PCl_3_ in dry toluene provided **27**, and treatment of **27** with our axially chiral biphenyldiols ((*S*)-**L1**, **L2** or **L3**) gave the corresponding phosphoramidite ligands (*S*)-**L14**, **L15** or **L16** in 90%, 83% and 71% yields, respectively, for the two step reactions. Subsequently, we prepared chiral phosphoramidite ligand (*S*)-**L18** in 56% yield via the similar procedures.

To evaluate reactivity and enantioselectivity of our newly developed chiral phosphoramidite ligands in asymmetric catalysis, two reactions were selected as the examples. Yang and Zhao reported the Pd-catalyzed [4 + 2] cycloaddition of benzofuran-derived azadienes with vinyl benzoxazinanones, and they found that cycloaddition of **30** with **31** in the presence of phosphoramidite ligand **L12** provided high yield (92%) and ee value (92% ee). However, the reaction was incomplete when (*S*)-**L13** was used as the ligand (Figures 8B and 8C).^52^ We attempted the cycloaddition of **30** with **31** in the presence of our chiral phosphoramidite ligands (*S*)-**L14**, **L15** or **L16**. Inspiringly, (*S*)-**L16** provided excellent diastereo- and enantioselectivity (>20:1 dr, 97% ee) (Figure 8C). Subsequently, another example was surveyed. In 2014, Lu and Xiao developed the Pd-catalyzed asymmetric decarboxylation-cycloaddition of vinyl benzoxazinanones with sulfur ylides.^53^ We investigated efficiency of the previous (*S*)-**L17** and our newly developed (*S*)-**L18** by using asymmetric decarboxylation-cycloaddition of **31** and **33**, and the results showed that (*S*)-**L18** was better than (*S*)-**L17** in asymmetric reaction of **31** and **33b** (Figure 8D).

### Chiral phosphoric acid-catalyzed asymmetric synthesis of 1,1′-spirobiindane-7,7′-diol (SPINOL) derivative and [4 + 3] cyclization

**Figure 9:**
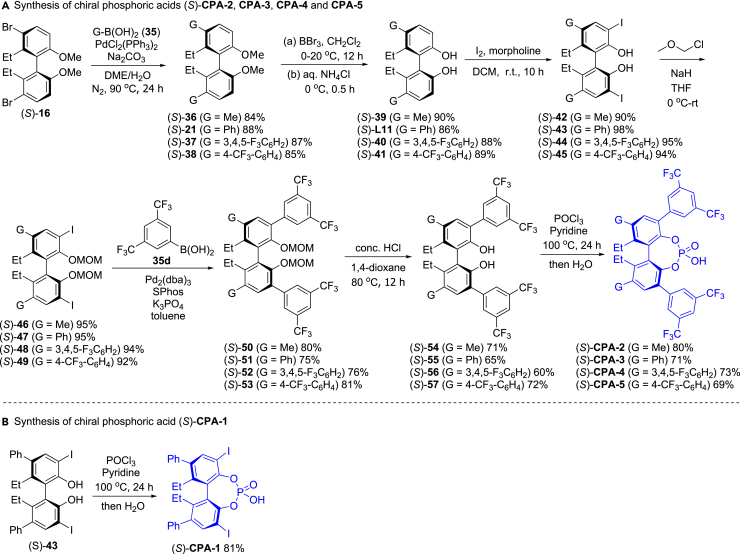
Synthesis and applications of chiral phosphoric acid catalysts (A) Synthesis of chiral phosphoric acids (*S*)-**CPA-2**, **CPA-3**, **CPA-4** and **CPA-5**. (B) Synthesis of chiral phosphoric acid (*S*)-**CPA-1**.

Since the pioneering research from the groups of Akiyama^54^ and Terada^55^ in 2004, chiral phosphoric acids are widely used as the organocatalysts in the asymmetric synthesis,^8^^,^^10^^,^^25^^,^^56^ in which the previous chiral phosphoric acid catalysts usually are BINOL and SPINOL-based derivatives. Here, we first performed synthesis of BIPOL-based chiral phosphoric acids. As shown in Figure 9A, the Suzuki couplings of (*S*)-**16** with alkyl boric acids (**35**) led to (*S*)-**36-39** in 84–88% yields, and demethylation of the two ethers in (*S*)-**21** and (*S*)-**36-38** with BBr_3_ in CH_2_Cl_2_ followed hydrolysis provided (*S*)-**39**, (*S*)-**L11**, (*S*)-**40** and (*S*)**-41** in 86–90% yields. Diiodization of (*S*)-**39**, (*S*)-**L11**, (*S*)-**40** and (*S*)**-41** at 3, 3′-position with iodine in CH_2_Cl_2_ in the presence of morpholine gave (*S*)-**42-45** in 90–98% yields, and couplings of (*S*)-**42-45** with chloro (methoxy) methane (MOM-Cl) using NaH as the base afforded the corresponding diethers ((*S*)-**46-49**) in 92–95% yields. The Suzuki couplings of (*S*)-**46-49** with (3,5-bis(trifluoromethyl)phenyl)boronic acid (**35d**ays) formed (*S*)-**50-53** in 75–81% yields, and deprotection of (*S*)-**50-53** with conc. HCl in dioxane provided substituted biphenyldiols (*S*)-**54-57** in 60–72% yields. Finally, couplings of (*S*)-**54-57** with POCl_3_ followed hydrolysis provided the corresponding chiral phosphoric acids (*S*)-**CPA-2**, (*S*)-**CPA-3**, (*S*)-**CPA-4** and (*S*)**-CPA-5** in 69–80% yields. Similarly, chiral phosphoric acids (*S*)-**CPA-1** was obtained in 81% yield using (*S*)-**47** as the material (Figure 9B).

**Figure 10:**
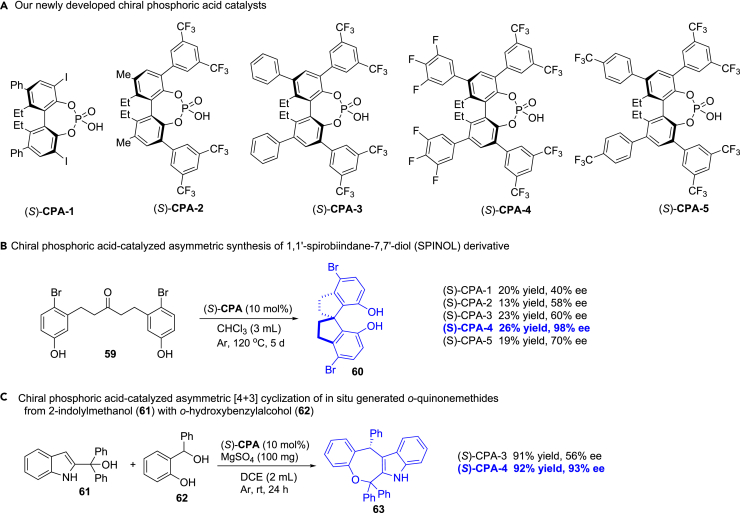
Applications of chiral phosphoric acid catalysts (A) Our newly developed chiral phosphoric acid catalysts. (B) Chiral phosphoric acid-catalyzed asymmetric synthesis of 1,1′-spirobiindane-7,7′-diol (SPINOL) derivative. (C) Chiral phosphoric acid-catalyzed asymmetric [4 + 3] cyclization of *in situ* generated *o*-quinonemethides from 2-indolylmethanol (**61**) with *o*-hydroxybenzylalcohol (**62**).

To evaluate reactivity and enantioselectivity of our newly developed chiral phosphoric acids in asymmetric catalysis (Figure 10A), we selected two reactions as the examples. In 2016, Tan et al. developed chiral phosphoric acid-catalyzed asymmetric synthesis of 1,1′-spirobiindane-7,7′-diol (SPINOL) derivatives (Figure 10B).^57^ When they used **59** as the substrate, the SPINOL-based chiral phosphoric acid-catalyzed reaction for five days only provided 19% yield with 93% ee. We attempted our chiral phosphoric acids to perform the same reaction, fortunately, (*S*)-**CPA-4** afforded 26% yield with 98% ee (Figure 10B). In 2019, Shi et al. reported chiral phosphoric acid-catalyzed asymmetric [4 + 3] cyclizations of *in situ* generated *ortho*-quinonemethides from *o*-hydroxybenzylalcohols with 2-indolylmethanols, and 90% ee was provided when reaction of **61** with **62** was performed with (*R*)-8H-BINOL-based chiral phosphoric acid (Figure 10C).^58^ We chose the reaction to evaluate our chiral phosphoric acids, and the results showed that (*S*)-**CPA-4** provided higher enantioselectivity (93% ee) than (*R*)-8H-BINOL-based chiral phosphoric acid (Figure 10C).

### Conclusions

We have developed a new kind of diverse adjustable axially chiral biphenyl ligands and catalysts. Six model reactions were performed including asymmetric additions of diethylzinc or alkynes to aldehydes in the presence of axially chiral BIPOL ligands, Pd-catalyzed asymmetric cycloadditions in the presence of chiral phosphoramidite ligands, and chiral phosphoric acid-catalyzed asymmetric synthesis of 1,1′-spirobiindane-7,7′-diol (SPINOL) derivative and [4 + 3] cyclization to evaluate reactivity and enantioselectivity of our biphenyl ligands and catalysts. We found that variation of 2,2′-substituent groups could provide different types of ligands and catalysts, and variation of substituent groups at the 3, 3′, 5,5′, 6,6′-positions could make ligands and catalysts more efficient in the asymmetric catalytic synthesis. We believe that our newly developed diverse adjustable axially chiral biphenyldiols will find wide applications in enantioselective catalysis.

### Limitations of the study

The synthesis of ligands and catalysts needs many steps in this study, and more concise synthetic pathways are required.

## STAR★Methods

### Key resources table

**Table:** 

REAGENT or RESOURCE	SOURCE	IDENTIFIER
Ti(O-*i*Pr)_4_	J&K Scientific	CAS: 546-68-9
ZnEt_2_	Energy-Chemical	CAS: 557-20-0
ZnMe_2_	Energy-Chemical	CAS: 544-97-8
Pd_2_(dba)_3_·CHCl_3_	Bidepharm	CAS: 52522-40-4
PdCl_2_(PPh_3_)_2_	Bidepharm	CAS: 13965-03-2
BBr_3_	Energy-Chemical	CAS: 10294-33-4
MeOCH_2_Cl	Sinopharm	CAS: 107-30-2
Benzaldehyde	Ouhechem	CAS: 100-52-7
4-Methoxybenzaldehyde	Bidepharm	CAS: 123-11-5
3-Methoxybenzaldehyde	Bidepharm	CAS: 591-31-1
2-Methoxybenzaldehyde	Ouhechem	CAS: 135-02-4
3,4-Dimethoxybenzaldehyde	Ouhechem	CAS: 120-14-9
4-Fluorobenzaldehyde	Bidepharm	CAS: 459-57-4
2-Fluorobenzaldehyde	Bidepharm	CAS: 446-52-6
4-Chlorobenzaldehyde	Bidepharm	CAS: 104-88-1
2-Chlorobenzaldehyde	Bidepharm	CAS: 89-98-5
4-Bromobenzaldehyde	Bidepharm	CAS: 1122-91-4
3-Bromobenzaldehyde	Bidepharm	CAS: 3132-99-8
4-Cyanobenzaldehyde	Bidepharm	CAS: 105-07-7
3-Cyanobenzaldehyde	Bidepharm	CAS: 24964-64-5
4-Fluoro-3-(trifluoromethyl)benzaldehyde	Bidepharm	CAS: 67515-60-0
2-Naphthaldehyde	Ouhechem	CAS: 66-99-9
2-Thenaldehyde	Ouhechem	CAS: 98-03-3
*trans*-Cinnamaldehyde	Ouhechem	CAS: 14371-10-9
Phenylacetylene	Bidepharm	CAS: 536-74-3
1-Ethynyl-2-methoxybenzene	Bidepharm	CAS: 767-91-9
4-Ethynylanisole	Bidepharm	CAS: 768-60-5
4-Chlorophenylacetylene	Bidepharm	CAS: 873-73-4
3-Chlorophenylacetylene	Bidepharm	CAS: 766-83-6
4-(Trifluoromethyl)phenylacetylene	Bidepharm	CAS: 705-31-7

**Deposited data**		

(*S*)-6,6'-dimethyl-[1,1'-biphenyl]-2,2'-diol ((*S*)-**L1**)	CCDC	CCDC 2209953
(*S*)-6,6'-diisopropyl-[1,1'-biphenyl]-2,2'-diol ((*S*)-**L3**)	CCDC	CCDC 2209954
(*S*)-5,5'-dibromo-6,6'-dimethyl-[1,1'-biphenyl]-2,2'-diol ((*S*)-**L5**)	CCDC	CCDC 2209951
(*S*)-5,5'-dichloro-6,6'-dimethyl-[1,1'-biphenyl]-2,2'-diol ((*S*)-**L8**)	CCDC	CCDC 2209952

**Software and algorithms**		

ChemDraw Ultra 12.0	PerkinElmer	https://www.perkinelmer.com/category/chemdraw

### Resource availability

#### Lead contact

Further information and requests should be directed to the lead contact, Hua Fu (fuhua@mail.tsinghua.edu.cn).

#### Materials availability

This study did not generate new unique materials.

The chemicals used in this study were obtained from standard commercial suppliers and used as received. Reactions were monitored by thin layer chromatography (TLC) and the products were obtained by column chromatography on silica gel. ^1^H NMR, ^13^C NMR, ^19^F NMR and ^31^P NMR were recorded on JEOL ECS-400 and were internally referenced to tetramethylsilane (TMS) and residual portion solvent signals (note: TMS referenced at 0.00 ppm; CDCl_3_ referenced at 7.26 ppm and 77.16 ppm respectively; DMSO-*d*_6_ referenced at 2.50 ppm and 39.52 ppm respectively). Data for ^1^H NMR are reported as follows: chemical shift (δ ppm), multiplicity (s = singlet, d = doublet, t = triplet, m = multiplet, dd = doublet of doublets, td = triplet of doublets, br = broad), coupling constant (J Hz). High-resolution mass spectra (HRMS) were recorded on FTICRMS BRUKER 7T and FTICRMS BRUKER 15T using matrix-assisted laser desorption ionization (MALDI-TOF) and LCMS-IT/TOF (SHIMADZU, Japan) with an electrospray ionization source (ESI-TOF). Chiral HPLC analysis was achieved using an Agilent 1100 Infinity series normal phase HPLC unit and Agilent Chemstation software. Daicel Chiralpak columns (250 × 4.6 mm) were used. Solvents were used of HPLC grade (Sigma Aldrich).

### Method details

#### Synthesis of (*S*)-2,2'-bis(bromomethyl)-6,6'-dimethoxy-1,1'-biphenyl ((*S*)-**3**)

To a solution of (*S*)-6,6'-dimethoxy-[1,1'-biphenyl]-2,2'-dicarbaldehyde ((*S*)-**1**) (50 mmol, 13.5 g) in EtOH (200 mL) was added NaBH_4_ (125 mmol, 4.7 g) in portions at 0^o^C, then allowed to warm to room temperature. After stirring for 8hat room temperature, the reaction mixture was cooled to 0^o^C and added to aqueous NH_4_Cl solution slowly until no more gas was released. Then concentrated under reduced pressure, the mixture was extracted with EtOAc. The combined organic phase was washed with water, brine and dried over Na_2_SO_4_, filtered, and evaporated in vacuo, affording the target product (*S*)-**2** as a white solid (13.4 g, 99% yield). The product was used for next step without further purification. To (*S*)**-2** (30 mmol, 8.2 g) in CH_2_Cl_2_ (DCM) (50 mL) was added a DCM (50 mL) solution of PBr_3_ (75 mmol, 1.5 M) dropwise at 0^o^C, the mixture was allowed to warm to room temperature after addition completed. After 5 h, the reaction mixture was put in ice bath and added to saturated Na_2_CO_3_ aqueous solution dropwise. The mixture was extracted with DCM, the combined organic layers was washed with water, brine, dried over Na_2_SO_4_, filtered and concentrated in vacuum, affording the target product (*S*)-**3**.

#### (*S*)-2,2'-Bis(bromomethyl)-6,6'-dimethoxy-1,1'-biphenyl ((*S*)-**3**)

^1^H NMR: (400 MHz, Chloroform-*d*) δ 7.38 (t, *J* = 8.0 Hz, 2H), 7.20 (d, *J* = 7.7 Hz, 2H), 6.94 (d, *J* = 8.1 Hz, 2H), 4.18 (s, 4H), 3.72 (s, 6H) ppm. ^13^C NMR: (101 MHz, Chloroform-*d*) δ 157.0, 137.9, 129.6, 124.3, 122.7, 110.9, 55.9, 32.0 ppm.

#### Synthesis of (*S*)-6,6'-dimethyl-[1,1'-biphenyl]-2,2'-diol ((*S*)-**L1**)

To a solution of (*S*)-**3** (30 mmol, 12.0 g) in MeOH/EtOAc (93 mL v/v 30:1) was added palladium on activated carbon (1 g, 10% wt). Then the resulting mixture was degassed, and charged with hydrogen balloon and stirred at room temperature for 24 h. After the reaction completed (monitored by TLC), the mixture was filtered and concentrated, affording the target product (*S*)**-4**. The residue was used for next step without further purification. To a flamed-dried 100 mL flask which contained (*S*)-**4** (30 mmol, 7.3 g) was charged dry DCM. The solution was cooled to 0^o^C before adding a DCM (50 mL) solution of BBr_3_ (105 mmol, 2M) dropwise. After addition completed, the reaction mixture was stirred for 1.5hat 0^o^C. Saturated aqueous NH_4_Cl solution was added to quench the reaction. The mixture was extracted with DCM, and the combined organic layer was washed with water, brine, dried over Na_2_SO_4_, and concentrated. The residue was purified by a column chromatography (eluent: PE:EtOAc = 10:1) to afford the desired product (*S*)-**L1** as a white solid (6.0 g, 95% yield).

#### (*S*)-6,6'-Dimethyl-[1,1'-biphenyl]-2,2'-diol ((*S*)-**L1**)

^1^H NMR: (400 MHz, Chloroform-*d*) δ 7.22 (t, *J* = 7.9 Hz, 2H), 6.89 (t, *J* = 8.0 Hz, 4H), 4.81 (s, 2H), 2.00 (s, 6H) ppm. ^13^C NMR: (101 MHz, Chloroform-*d*) δ 154.0, 139.1, 130.1, 122.6, 119.8, 113.3, 19.6 ppm. HRMS (ESI-TOF) *m*/*z* calcd for [C_14_H_15_O_2_] [M+H]^+^ 215.1072, found 215.1091.

#### Synthesis of (*S*)-2,2'-dimethoxy-6,6'-divinyl-1,1'-biphenyl ((*S*)-**5**)

In an oven dried 500 mL three-neck round-bottom flask which contained methyltriphenylphosphonium bromide (82 mmol, 29.3 g) was charged with dry THF (300 mL). The solution was cooled to 0^o^C, and *t*-BuOK (80 mmol, 9.0 g) in portions was added under argon atmosphere. After a stirring for 0.5 h at 0^o^C, (*S*)-**1** (20 mmol, 5.4 g) in THF (50 mL) was added dropwise via a syringe to the resulting solution. The reaction mixture was allowed to warm to room temperature and stirred for 8 h. The resulting solution was then diluted with EtOAc and H_2_O. The organic layer was separated and the aqueous layer was extracted with EtOAc. The combined organic layer was washed with brine, dried over Na_2_SO_4_, filtered, and then concentrated in vacuo. The residue was purified by silica gel chromatography (eluent: PE:EtOAc = 10:1) to provide compound (*S*)-**5** as colorless solid (5.0 g, 95% yield).

#### (*S*)-2,2'-Dimethoxy-6,6'-divinyl-1,1'-biphenyl ((*S*)-**5**)

^1^H NMR: (400 MHz, Chloroform-*d*) δ 7.41 – 7.31 (m, 4H), 6.97 – 6.89 (m, 2H), 6.30 (dd, J = 17.5, 11.0 Hz, 2H), 5.66 (d, *J* = 17.5 Hz, 2H), 5.08 (d, *J* = 11.0 Hz, 2H), 3.71 (s, 6H) ppm. ^13^C NMR: (101 MHz, Chloroform-*d*) δ 157.5, 138.2, 138.2, 135.1, 135.1, 128.5, 124.8, 117.2, 114.7, 110.2, 56.1 ppm.

#### Synthesis of (*S*)-6,6'-diethyl-[1,1'-biphenyl]-2,2'-diol ((*S*)-**L2**)

To a solution of (*S*)-**5** (19 mmol, 5.0 g) in MeOH/EtOAc (62 mL v/v 30:1) was added palladium on activated carbon (0.5 g, 10% wt). Then the resulting mixture was degassed, and charged with hydrogen balloon and stirred at room temperature for 24 h. After the reaction completed (monitored by TLC), the mixture was filtered and concentrated affording the target product (*S*)-**6**. The residue was used for next step without further purification. To a flamed-dried 100 mL flask which contained (*S*)-**6** (15 mmol, 4.0 g) was charged dry DCM. The solution was cooled to 0^o^C before adding a DCM (25 mL) solution of BBr_3_ (53 mmol, 2 M) dropwise. After the addition completed, the reaction mixture stirring for 1.5hat 0^o^C. Saturated aqueous NH_4_Cl solution was added to quench the reaction. The mixture was extracted with DCM, the combined organic layer was washed with water, brine, dried over Na_2_SO_4_, filtered and concentrated. The residue was purified by a column chromatography (eluent: PE:EtOAc = 10:1) to afford the desired product (*S*)-**L2** as a white solid (3.4 g, 94% yield).

#### (*S*)-6,6'-Diethyl-[1,1'-biphenyl]-2,2'-diol ((*S*)-**L2**)

^1^H NMR: (400 MHz, Chloroform-*d*) δ 7.30 (t, *J* = 7.9 Hz, 2H), 6.96 (d, *J* = 7.6 Hz, 2H), 6.90 (d, *J* = 8.2 Hz, 2H), 4.72 (s, 2H), 2.29 (q, *J* = 7.4 Hz, 4H), 1.06 (t, *J* = 7.6 Hz, 6H) ppm. ^13^C NMR: (101 MHz, Chloroform-*d*) δ 153.9, 145.1, 130.4, 120.9, 118.8, 113.2, 26.2, 14.7 ppm. HRMS (ESI-TOF) *m*/*z* calcd for [C_16_H_19_O_2_] [M+H]^+^ 243.1380, found 243.1379.

#### Synthesis of (*S*)-dimethyl 6,6'-dimethoxy-[1,1'-biphenyl]-2,2'-dicarboxylate ((*S*)-**8**)

To a 500 mL three-neck round-bottom flask which contained (*S*)-**1** (50 mmol, 13.5 g) and KH_2_PO_4_ (30 mmol, 4.1 g) was charged 200 mL MeCN and 100 mL H_2_O. The suspension was heated at 50^o^C before adding NaClO_2_ (166 mmol, 15 g) in one portion. 30% aqueous H_2_O_2_ (11 mL) was added dropwise via a syringe, the mixture was stirred at 50^o^C for 1 h. After the mixture was cooled to room temperature, the reaction mixture was acidified to pH = 7 with 1N HCl aqueous solution (100 mL). The solution was concentrated in vacuo, then extracted with EtOAc, and the combined organic layer was washed with water, brine, dried over Na_2_SO_4_, filtered and concentrated. The residue was used for next step without further purification. To a solution of (*S*)-**7** (50 mmol, 15.1 g) in MeOH (100 mL) was added concentrated H_2_SO_4_ (1 mL). Then the solution was heated at 70^o^C for 24 h. After the resulting solution was cooled to room temperature, concentrated in vacuo, and diluted with H_2_O. The mixture was extracted with EtOAc, and the combined organic layer was washed with water, brine, dried over Na_2_SO_4_, filtered and concentrated. The residue was recrystallize in DCM/PE (v/v=1:10) to afford the desired product (*S*)-**8** as a white solid (16.2 g, 98% yield).

#### (*S*)-Dimethyl 6,6'-dimethoxy-[1,1'-biphenyl]-2,2'-dicarboxylate ((*S*)-**8**)

^1^H NMR: (400 MHz, Chloroform-*d*) δ 7.61 (d, *J* = 7.8 Hz, 2H), 7.39 (t, *J* = 8.1 Hz, 2H), 7.10 (d, *J* = 7.7 Hz, 2H) 3.69 (s, 6H), 3.59 (s, 6H) ppm. ^13^C NMR: (101 MHz, Chloroform-*d*) δ 167.6, 157.0, 131.4, 128.2, 127.8, 122.2, 114.3, 56.2, 51.8 ppm.

#### Synthesis of (*S*)-2,2'-(6,6'-dimethoxy-[1,1'-biphenyl]-2,2'-diyl)bis(propan-2-ol) ((*S*)-**9**)

To a 500 mL three-neck round-bottom flask which contained (*S*)-**8** (20 mmol, 6.6 g) was added 200 mL dry THF. To the solution was added THF solution of methylmagnesium bromide (200 mL, 1M) dropwise via a syringe at 0^o^C under argon atmosphere. After the addition was finished, the reaction was maintained at 0^o^C for another 30 min, then the solution was heated to reflux for 24 h. The resulting mixture was cooled by ice bath, and then saturated aqueous NH_4_Cl solution was added dropwise to quench the reaction. The resulting mixture was extracted with EtOAc, and the combined organic layer was washed with water, brine, dried over Na_2_SO_4_, filtered and concentrated. The residue was purified by a column chromatography (eluent: PE:EtOAc = 5:1) to afford the desired product (*S*)-**9** as a white solid (5.8 g, 89% yield).

#### (*S*)-2,2'-(6,6'-Dimethoxy-[1,1'-biphenyl]-2,2'-diyl)bis(propan-2-ol) ((*S*)-**9**)

^1^H NMR: (400 MHz, Chloroform-*d*) δ 7.30 (t, *J* = 8.1 Hz, 2H), 7.04 (d, *J* = 8.1 Hz, 2H), 6.84 (d, *J* = 8.1 Hz, 2H), 3.64 (s, 6H), 2.93 (s, 2H), 1.61 (s, 6H), 1.33 (s, 6H) ppm. ^13^C NMR: (101 MHz, Chloroform-*d*) δ 156.9, 147.4, 128.3, 125.2, 119.0, 109.3, 74.6, 55.7, 32.0, 31.9 ppm.

#### Synthesis of (*S*)-2,2'-dimethoxy-6,6'-di(prop-1-en-2-yl)-1,1'-biphenyl ((*S*)-**10**)

To a solution of (*S*)-**9** (1 mmol, 330 mg) in MeCN was added thionyl chloride (3 mmol, 217 μL) at 0^o^C, the mixture was stirred at 0^o^C for 15 min before adding pyridine (4 mmol, 323 μL) dropwise. After the addition was finished, the reaction mixture was allowed to warm to room temperature and stirred for 12 h. After completion, the reaction was quenched by H_2_O. The organic layer was separated and the aqueous layer was extracted with DCM. The combined organic layer was washed with brine, dried over Na_2_SO_4_, filtered, and concentrated. The residue was purified by a column chromatography (eluent: PE:EtOAc = 50:1) to afford the desired product (*S*)-**10** as gum (244 mg, 83%).

#### (*S*)-2,2'-Dimethoxy-6,6'-di(prop-1-en-2-yl)-1,1'-biphenyl ((*S*)-**10**)

^1^H NMR: (400 MHz, Chloroform-*d*) δ 7.32 – 7.24 (m, 2H), 6.91 (d, J = 7.6 Hz, 2H), 6.84 (d, J = 8.2 Hz, 2H), 4.82 (s, 2H), 4.65 (s, 2H), 3.72 (s, 6H), 1.73 (s, 6H) ppm. ^13^C NMR: (101 MHz, Chloroform-*d*) δ 157.6, 144.9, 144.6, 128.0, 124.7, 120.7, 115.0, 109.1, 55.7, 23.3 ppm.

#### Synthesis of (*S*)-6,6'-diisopropyl-[1,1'-biphenyl]-2,2'-diol ((*S*)-**L3**)

To a solution of (*S*)-**10** (5 mmol, 1.5 g) in MeOH/EtOAc (16 mL v/v 30:1) was added palladium on activated carbon (0.15 g, 10% wt). Then the resulting mixture was degassed, and charged with hydrogen balloon and stirred at room temperature for 24 h. After the reaction completed as monitored by TLC, the mixture was filtered and concentrated affording the target product (*S*)-**11**. The residue was used for next step without further purification. To a flamed-dried 100 mL flask which contained (*S*)-**11** (5 mmol, 1.5 g) was charged dry DCM. The solution was cooled to 0 ^o^C before adding a DCM (10 mL) solution of BBr_3_ (17.5 mmol, 1.8 M) dropwise. After addition completed, the reaction mixture stirring for 1.5hat 0^o^C. Saturated aqueous NH_4_Cl solution was added to quench the reaction. The mixture was extracted with DCM, and the combined organic layer was washed with water, brine, dried over Na_2_SO_4_, filtered and concentrated. The residue was purified by a column chromatography (eluent: PE:EtOAc = 10:1) to afford the desired product (*S*)-**L3** as a white solid (1.2 g, 90% yield).

#### (*S*)-6,6'-Diisopropyl-[1,1'-biphenyl]-2,2'-diol ((*S*)-**L3**)

^1^H NMR: (400 MHz, Chloroform-*d*) δ 7.36 (t, *J* = 8.0 Hz, 2H), 7.04 (d, *J* = 7.8 Hz, 2H), 6.90 (d, *J* = 8.0 Hz, 2H), 4.65 (s, 2H), 2.54 (p, *J* = 6.8 Hz, 2H), 1.20 – 1.00 (m, 12H) ppm. ^13^C NMR: (101 MHz, Chloroform-*d*) δ 153.8, 150.4, 130.6, 118.3, 118.0, 113.0, 30.5, 24.9, 23.5 ppm. HRMS (ESI-TOF) *m*/*z* calcd for [C_18_H_23_O_2_] [M+H]^+^ 271.1698, found 271.1711.

#### Synthesis of (*S*)-6,6'-bis(methoxymethoxy)-[1,1'-biphenyl]-2,2'-dicarbaldehyde ((*S*)-**13**)

To a 100 mL three-neck round-bottom flask which contained (*S*)-**1** (5 mmol, 1.35 g) was charged dry DCM. The solution was cooled to -78^o^C before adding a DCM (10 mL) solution of BBr_3_ (17.5 mmol, 1.8 M) dropwise under argon atmosphere. After the addition completed, the reaction mixture was stirred for 2hat -78^o^C. Saturated aqueous NH_4_Cl solution was added to quench the reaction. The mixture was extracted with DCM, and the combined organic layer was washed with water, brine, dried over Na_2_SO_4_, filtered and concentrated. The residue was used for next step without further purification. The above residue was dissolve in dry MeCN, K_2_CO_3_ (12.5 mmol, 1.7 g) was added at 0^o^C, and then chloromethyl methyl ether (13 mmol, 1.0 mL) was added dropwise. The mixture was stirred for 5 h until TLC indicated reaction completion. The reaction was diluted with EtOAc and H_2_O, the organic phase was separated and the aqueous solution was poured into 20 mL EtOAc. The combined organic layers was washed with water, brine, dried over Na_2_SO_4_, filtered and concentrated. The residue was purified by a column chromatography (eluent: PE:EtOAc = 10:1) to afford the desired product (*S*)-**13** as a yellow solid (1.5 g, 90% yield).

#### (*S*)-6,6'-Bis(methoxymethoxy)-[1,1'-biphenyl]-2,2'-dicarbaldehyde ((*S*)-**13**)

^1^H NMR: (400 MHz, Chloroform-*d*) δ 9.70 (s, 2H), 7.73 (d, *J* = 7.4 Hz, 2H), 7.53 (t, *J* = 8.0 Hz, 1H), 7.47 (d, *J* = 8.2 Hz, 1H), 5.10 – 4.99 (m, 4H), 3.26 (s, 6H) ppm. ^13^C NMR: (101 MHz, Chloroform-*d*) δ 191.5, 155.2, 136.0, 130.0, 126.6, 121.3, 120.1, 94.9, 56.3 ppm. EI-MS *m*/*z* 330.

#### Synthesis of (*S*)-2,2'-bis(difluoromethyl)-6,6'-bis(methoxymethoxy)-1,1'-biphenyl ((*S*)-**14**)

A flame-dried 50 mL Schlenk tube equipped with a magnetic stirring bar was cooled to room temperature. To this flask were added (*S*)-**13** (0.8 mmol, 264 mg) and DCM (5 mL). To the mixture was added bis(2-methoxyethyl) aminosulfur trifluoride (BAST) (12.8 mmol, 2.4 mL) at 0^o^C dropwise, and the reaction was maintained at 0^o^C for another 5 min. Then a drop of EtOH was added and the tube was sealed at once. The reaction was heated at 50^o^C for 36 h. The resulting mixture was cooled by ice bath, and then saturated aqueous Na_2_CO_3_ solution was added dropwise to quench the reaction. The mixture was extracted with DCM, and the combined organic layer was washed with water, brine, dried over Na_2_SO_4_, filtered and concentrated. The residue was purified by a column chromatography (eluent: PE:EtOAc = 10:1) to afford the desired product (*S*)-**14** as a white solid (215 mg, 72% yield).

#### (*S*)-2,2'-Bis(difluoromethyl)-6,6'-bis(methoxymethoxy)-1,1'-biphenyl ((*S*)-**14**)

^1^H NMR: (400 MHz, Chloroform-*d*) δ 7.50 (t, *J* = 8.0 Hz, 2H), 7.44 (d, *J* = 7.7 Hz, 2H), 7.35 (d, *J* = 8.1 Hz, 2H), 6.21 (t, *J* = 55.3 Hz, 2H), 5.06 (q, *J* = 6.9 Hz, 4H), 3.31 (s, 6H) ppm. ^13^C NMR: (101 MHz, Chloroform-*d*) δ 154.8, 134.81 (t, *J* = 22.5 Hz), 130.2, 123.1 (t, *J* = 6.4 Hz), 118.6 (t, *J* = 5.4 Hz), 116.7, 113.0 (t, *J* = 237.4 Hz), 94.7, 56.0 ppm. ^19^F NMR: (282 MHz, Chloroform-*d*) δ-109.93 (d, J = 55.6 Hz),-110.74 (d, J = 55.1 Hz),-112.07 (d, J = 55.2 Hz),-112.88 (d, J = 55.6 Hz) ppm. EI-MS *m*/*z* 374.

#### Synthesis of (*S*)-6,6'-bis(difluoromethyl)-[1,1'-biphenyl]-2,2'-diol ((*S*)-**L4**)

To a solution of (*S*)-**14** (0.5 mmol, 187 mg) in 1,4-dioxane (10 mL) was added concentrated HCl (12 N, 0.1 mL). The mixture was heated at 60^o^C for 3 h, after completion, and then the reaction mixture was cooled and diluted with DCM and water. The solution was extracted with DCM, and the combined organic layer was washed with water, brine, dried over Na_2_SO_4_, filtered and concentrated. The residue was purified by a column chromatography (eluent: PE:EtOAc = 8:1) to afford the desired product (*S*)-**L4** as a white solid (136 mg, 95% yield).

#### (*S*)-6,6'-Bis(difluoromethyl)-[1,1'-biphenyl]-2,2'-diol ((*S*)-**L4**)

^1^H NMR: (400 MHz, DMSO-*d*_6_) δ 9.78 (s, 2H), 7.37 (t, *J* = 7.9 Hz, 2H), 7.20 – 7.00 (m, 4H), 6.27 (t, *J* = 55.5 Hz, 2H) ppm. ^13^C NMR: (101 MHz, DMSO-*d*_6_) δ 155.3, 134.61 (t, *J* = 21.8 Hz), 130.0, 120.82 (t, *J* = 5.9 Hz), 118.1, 114.02 (t, *J* = 235.9 Hz), 115.9 ppm. ^19^F NMR:(282 MHz, DMSO-*d*_6_) δ-109.78 (d, J = 55.7 Hz),-110.59 (d, J = 55.7 Hz),-112.73 (d, J = 54.7 Hz),-113.54 (d, J = 55.1 Hz) ppm. HRMS (ESI-TOF) *m*/*z* calcd for [C_14_H_11_F_4_O_2_] [M+H]^+^ 287.0695, found 287.0711.

#### Synthesis of (*S*)-3,3'-dibromo-6,6'-dimethoxy-2,2'-dimethyl-1,1'-biphenyl ((*S*)-**15**)

To a solution of (*S*)-**4** (1.5 mmol, 363 mg) in DMF (10 mL) was added NBS (3.15 mmol, 561 mg) in one portion. The reaction mixture was stirred at room temperature for 12 h. TLC indicated full consumption of the starting material. H_2_O was added to quench the reaction. The mixture was extracted with DCM, and the combined organic layer was washed with water, brine, dried over Na_2_SO_4_, filtered and concentrated. The residue was purified by a column chromatography (eluent: PE:EtOAc = 50:1) to afford the desired product (*S*)-**15** as a colorless solid (570 mg, 95% yield).

#### (*S*)-3,3'-Dibromo-6,6'-dimethoxy-2,2'-dimethyl-1,1'-biphenyl ((*S*)-**15**)

^1^H NMR: (400 MHz, DMSO-*d*_6_) δ 7.52 (d, *J* = 8.8 Hz, 2H), 6.71 (d, *J* = 8.8 Hz, 2H), 3.67 (s, 6H), 2.01 (s, 6H) ppm. ^13^C NMR: (101 MHz, DMSO-*d*_6_) δ 156.2, 137.6, 132.0, 127.8, 116.5, 110.0, 56.0, 20.12 ppm.

#### Synthesis of (*S*)-5,5'-dibromo-6,6'-dimethyl-[1,1'-biphenyl]-2,2'-diol ((*S*)-**L5**)

To a flamed-dried 100 mL flask which contained (*S*)-**15** (5 mmol, 2.0 g) was charged dry DCM. The solution was cooled to 0^o^C before adding a DCM (15 mL) solution of BBr_3_ (30 mmol, 2.9 mL) dropwise. After the addition completed, the reaction mixture was allowed to warm to room temperature and stirred for 12 h. Saturated aqueous NH_4_Cl solution was added to quench the reaction. The mixture was extracted with DCM, and the combined organic layer was washed with water, brine, dried over Na_2_SO_4_, filtered and concentrated. The residue was purified by a column chromatography (eluent: PE:EtOAc = 10:1) to afford the desired product (*S*)-**L5** as a white solid (1.7 g, 94% yield).

#### (*S*)-5,5'-Dibromo-6,6'-dimethyl-[1,1'-biphenyl]-2,2'-diol ((*S*)-**L5**)

^1^H NMR: (400 MHz, Chloroform-*d*) δ 7.53 (d, *J* = 8.7 Hz, 2H), 6.81 (d, *J* = 8.7 Hz, 2H), 4.67 (s, 2H), 2.06 (s, 6H) ppm. ^13^C NMR: (101 MHz, Chloroform-*d*) δ 153.0, 138.2, 134.3, 121.6, 116.6, 115.3, 20.2 ppm. HRMS (ESI-TOF) *m*/*z* calcd for [C_14_H_13_Br_2_O_2_] [M+H]^+^ 370.9277, found 370.9270.

#### Synthesis of (*S*)-3,3'-dibromo-2,2'-diethyl-6,6'-dimethoxy- 1,1'-biphenyl ((*S*)-**16**)

To a solution of (*S*)-**6** (1.5 mmol, 405 mg) in DMF (10 mL) was added NBS (3.15 mmol, 561 mg) in one portion. The reaction mixture was stirred at room temperature for 12 h. TLC indicated full consumption of the starting material. H_2_O was added to quench the reaction. The mixture was extracted with DCM, and combined organic layer was washed with water, brine, dried over Na_2_SO_4_, filtered and concentrated. The residue was purified by a column chromatography (eluent: PE:EtOAc = 50:1) to afford the desired product (*S*)-**16** as a colorless gum (603 mg, 94% yield).

#### (*S*)-3,3'-Dibromo-2,2'-diethyl-6,6'-dimethoxy- 1,1'-biphenyl ((*S*)-**16**)

^1^H NMR: (400 MHz, Chloroform-*d*) δ 7.54 (d, *J* = 8.8 Hz, 2H), 6.72 (d, *J* = 8.8 Hz, 2H), 3.66 (s, 6H), 2.58 – 2.43 (m, 2H), 2.35 – 2.21 (m, 2H), 0.93 (t, *J* = 7.5 Hz, 6H) ppm. ^13^C NMR: (101 MHz, Chloroform-*d*) δ 156.6, 142.6, 132.7, 127.3, 115.8, 110.1, 55.7, 27.5, 12.8 ppm.

#### Synthesis of (*S*)-5,5'-dibromo-6,6'-diethyl-[1,1'-biphenyl]-2,2'-diol ((*S*)-**L6**)

To a flamed-dried 100 mL flask which contained (*S*)-**16** (5 mmol, 2.1 g) was charged dry DCM. The solution was cooled to 0^o^C before adding a DCM (15 mL) solution of BBr_3_ (30 mmol, 2.9 mL) dropwise. After the addition completed, the reaction mixture was allowed to warm to room temperature and stirred for 12 h. Saturated aqueous NH_4_Cl solution was added to quench the reaction. The mixture was extracted with DCM, and the combined organic layer was washed with water, brine, dried over Na_2_SO_4_, filtered and concentrated. The residue was purified by a column chromatography (eluent: PE:EtOAc = 10:1) to afford the desired product (*S*)-**L6** as a white solid (1.8 g, 94% yield).

#### (*S*)-5,5'-Dibromo-6,6'-diethyl-[1,1'-biphenyl]-2,2'-diol ((*S*)-**L6**)

^1^H NMR: (400 MHz, Chloroform-*d*) δ 7.56 (d, *J* = 8.8 Hz, 2H), 6.84 (d, *J* = 8.7 Hz, 2H), 4.64 (s, 2H), 2.63 – 2.51 (m, 2H), 2.39 – 2.25 (m, 2H), 0.99 (t, *J* = 7.5 Hz, 6H) ppm. ^13^C NMR: (101 MHz, Chloroform-*d*) δ 153.3, 143.8, 135.0, 120.8, 116.1, 115.5, 27.8, 13.2 ppm. HRMS (ESI-TOF) *m*/*z* calcd for [C_16_H_17_Br_2_O_2_] [M+H]^+^ 400.9575, found 400.9592.

#### Synthesis of (*S*)-3,3'-dibromo-2,2'-diisopropyl-6,6'-dimethoxy -1,1'-biphenyl ((*S*)-**17**)

To a solution of (*S*)-**11** (1.5 mmol, 447 mg) in DMF (10 mL) was added NBS (3.15 mmol, 561 mg) in one portion. The reaction mixture was stirred at room temperature for 12 h. TLC indicated full consumption of the starting material. H_2_O was added to quench the reaction. The mixture was extracted with DCM, and the combined organic layer was washed with water, brine, dried over Na_2_SO_4_, filtered and concentrated. The residue was purified by a column chromatography (eluent: PE:EtOAc = 50:1) to afford the desired product (*S*)-**17** as a colorless gum (609 mg, 89% yield).

#### (*S*)-3,3'-Dibromo-2,2'-diisopropyl-6,6'-dimethoxy -1,1'-biphenyl ((*S*)-**17**)

^1^H NMR: (400 MHz, Chloroform-*d*) δ 7.54 (d, *J* = 8.7 Hz, 2H), 6.69 (d, *J* = 8.7 Hz, 2H), 3.65 (s, 6H), 2.84 (s, 2H), 1.40 – 1.20 (m, 12H) ppm. ^13^C NMR: (101 MHz, Chloroform-*d*) δ 156.2, 145.4, 134.8, 128.3, 113.3, 109.7, 55.6, 32.4, 20.0, 19.6 ppm.

#### Synthesis of (*S*)-5,5'-dibromo-6,6'-diisopropyl-[1,1'-biphenyl]-2,2'-diol ((*S*)-**L7**)

To a flamed-dried 100 mL flask which contained (*S*)-**17** (5 mmol, 2.2 g) was charged dry DCM. The solution was cooled to 0^o^C before adding a DCM (15 mL) solution of BBr_3_ (30 mmol, 2.9 mL) dropwise. After the addition completed, the reaction mixture was allowed to warm to room temperature and stirred for 12 h. Saturated aqueous NH_4_Cl solution was added to quench the reaction. The mixture was extracted with DCM, and the combined organic layer was washed with water, brine, dried over Na_2_SO_4_, filtered and concentrated. The residue was purified by a column chromatography (eluent: PE:EtOAc = 10:1) to afford the desired product (*S*)-**L7** as a white solid (1.9 g, 90% yield).

#### (*S*)-5,5'-Dibromo-6,6'-diisopropyl-[1,1'-biphenyl]-2,2'-diol ((*S*)-**L7**)

^1^H NMR: (400 MHz, Chloroform-*d*) δ 7.53 (d, *J* = 8.7 Hz, 2H), 6.79 (d, *J* = 8.7 Hz, 2H), 4.67 (s, 2H), 2.82 (s, 2H), 1.4 – 1.2 (m, 12H) ppm. ^13^C NMR: (101 MHz, Chloroform-*d*) δ 153.2, 146.3, 137.1, 122.0, 115.5, 113.8, 33.0, 27.0, 20.2, 19.9 ppm. HRMS (ESI-TOF) *m*/*z* calcd for [C_18_H_21_Br_2_O_2_] [M+H]^+^ 428.9888, found 428.9901.

#### Synthesis of (*S*)-3,3'-dichloro-6,6'-dimethoxy-2,2'-dimethyl-1,1'-biphenyl ((*S*)-**18**)

To a solution of (*S*)-**4** (2 mmol, 484 mg) in chloroform (10 mL) was added NCS (8 mmol, 1.1 g) in one portion,^1^ and DMSO (0.4 mmol, 28 μL) was added. The reaction mixture was stirred at room temperature for 24 h. TLC indicated full consumption of the starting material. H_2_O was added to quench the reaction. The mixture was extracted with DCM, and the combined organic layer was washed with water, brine, dried over Na_2_SO_4_, filtered and concentrated. The residue was purified by a column chromatography (eluent: PE:EtOAc = 25:1) to afford the desired product (*S*)-**18** as a colorless gum (564 mg, 91% yield).

#### (*S*)-3,3'-Dichloro-6,6'-dimethoxy-2,2'-dimethyl-1,1'-biphenyl ((*S*)-**18**)

^1^H NMR: (400 MHz, Chloroform-*d*) δ 7.34 (d, *J* = 8.8 Hz, 2H), 6.77 (d, *J* = 8.8 Hz, 2H), 3.68 (s, 6H), 1.98 (s, 6H) ppm. ^13^C NMR: (101 MHz, Chloroform-*d*) δ 155.6, 136.0, 128.8, 127.5, 126.5, 109.5, 56.0, 17.2 ppm.

#### Synthesis of (*S*)-5,5'-dichloro-6,6'-dimethyl-[1,1'-biphenyl]-2,2'-diol ((*S*)-**L8**)

To a flamed-dried 100 mL flask which contained (*S*)-**18** (5 mmol, 1.55 g) was charged dry DCM. The solution was cooled to 0^o^C before adding a DCM (15 mL) solution of BBr_3_ (30 mmol, 2.9 mL) dropwise. After addition completed, the reaction mixture was allowed to warm to room temperature and stirred for 12 h. Saturated aqueous NH_4_Cl solution was added to quench the reaction. The mixture was extracted with DCM, and the combined organic layer was washed with water, brine, dried over Na_2_SO_4_, filtered and concentrated. The residue was purified by a column chromatography (eluent: PE:EtOAc = 10:1) to afford the desired product (*S*)-**L8** as a white solid (1.3 g, 95% yield).

#### (*S*)-5,5'-Dichloro-6,6'-dimethyl-[1,1'-biphenyl]-2,2'-diol ((*S*)-**L8**)

^1^H NMR: (400 MHz, Chloroform-*d*) δ 7.35 (d, *J* = 8.7 Hz, 2H), 6.86 (d, *J* = 8.7 Hz, 2H), 4.62 (s, 2H), 2.03 (s, 6H) ppm. ^13^C NMR: (101 MHz, Chloroform-*d*) δ 152.4, 136.6, 131.1, 126.9, 121.2, 114.8, 17.3 ppm. HRMS (ESI-TOF) *m*/*z* calcd for [C_14_H_11_Cl_2_O_2_] [M-H]^+^ 281.0131, found 281.0104.

#### Synthesis of (*S*)-6,6'-dimethoxy-2,2',3,3'-tetramethyl-1,1'-biphenyl ((*S*)-**19**)

To a 100 mL three-neck round-bottom flask which contained (*S*)-**15** (2 mmol, 800 mg), PdCl_2_(PPh_3_)_2_ (0.3 mmol, 210 mg) and MeB(OH)_2_ (14 mmol, 839 mg) was added DME (9 mL). After adding an aqueous (3 mL) solution of Na_2_CO_3_ (14 mmol, 1.5 g) via a syringe at room temperature under argon atmosphere, the mixture was reflux for 24 h. The reaction solution was cooled to room temperature and extracted with EtOAc, and combined organic layer was washed with water, brine, dried over Na_2_SO_4_, filtered and concentrated. The residue was purified by a column chromatography (eluent: PE:EtOAc = 25:1) to afford the desired product (*S*)-**19** as a white solid (432 mg, 80% yield).

#### (*S*)-6,6'-Dimethoxy-2,2',3,3'-tetramethyl-1,1'-biphenyl ((*S*)-**19**)

^1^H NMR: (400 MHz, Chloroform-*d*) δ 7.13 (d, J = 8.3 Hz, 2H), 6.76 (d, J = 8.3 Hz, 2H), 3.68 (s, 6H), 2.28 (s, 6H), 1.85 (s, 6H) ppm. ^13^C NMR: (101 MHz, Chloroform-*d*) δ 155.4, 136.6, 129.1, 128.7, 127.0, 108.2, 56.0, 20.1, 16.4 ppm.

#### Synthesis of (*S*)-5,5',6,6'-tetramethyl-[1,1'-biphenyl]-2,2'-diol ((*S*)-**L9**)

To a flamed-dried 100 mL flask which contained (*S*)-**19** (5 mmol, 1.35 g) was charged dry DCM. The solution was cooled to 0^o^C before adding a DCM (15 mL) solution of BBr_3_ (30 mmol, 2.9 mL) dropwise. After the addition completed, the reaction mixture was allowed to warm to room temperature and stirred for 12 h. Saturated aqueous NH_4_Cl solution was added to quench the reaction. The mixture was extracted with DCM, and the combined organic layer was washed with water, brine, dried over Na_2_SO_4_, filtered and concentrated. The residue was purified by a column chromatography (eluent: PE:EtOAc = 10:1) to afford the desired product (*S*)-**L9** as a white solid (1.1 g, 91% yield).

#### (*S*)-5,5',6,6'-Tetramethyl-[1,1'-biphenyl]-2,2'-diol ((*S*)-**L9**)

^1^H NMR: (400 MHz, Chloroform-*d*) δ 7.13 (d, J = 8.3 Hz, 2H), 6.81 (d, J = 8.3 Hz, 2H), 4.54 (s, 2H), 2.26 (s, 6H), 1.90 (s, 6H) ppm. ^13^C NMR: (101 MHz, Chloroform-*d*) δ 151.9, 137.0, 131.4, 129.3, 120.3, 112.7, 19.9, 16.4 ppm. HRMS (ESI-TOF) *m*/*z* calcd for [C_16_H_19_O_2_] [M+H]^+^ 243.1385, found 243.1401.

#### Synthesis of (*S*)-4',6''-dimethoxy-2',2''-dimethyl-1,1':3',1'':3'',1'''-quaterphenyl ((*S*)-**20**)

To a 100 mL three-neck round-bottom flask which contained (*S*)-**15** (2 mmol, 800 mg), PdCl_2_(PPh_3_)_2_ (0.3 mmol, 210 mg) and PhB(OH)_2_ (10 mmol, 1.2 g) was added DME (9 mL). After adding an aqueous (3 mL) solution of Na_2_CO_3_ (10 mmol, 1.06 g) via a syringe at room temperature under argon atmosphere, the mixture was reflux for 24 h. The reaction solution was cooled to room temperature and extracted with EtOAc, and the combined organic layer was washed with water, brine, dried over Na_2_SO_4_, filtered and concentrated. The residue was purified by a column chromatography (eluent: PE:EtOAc = 25:1) to afford the desired product (*S*)-**20** as a white solid (701 mg, 89% yield).

#### (*S*)-4',6''-Dimethoxy-2',2''-dimethyl-1,1':3',1'':3'',1'''-quaterphenyl ((*S*)-**20**)

^1^H NMR: (400 MHz, Chloroform-*d*) δ 7.47 – 7.31 (m, 10H), 7.30 – 7.25 (m, 2H), 6.94 (d, *J* = 8.4 Hz, 2H), 3.80 (s, 6H), 1.92 (s, 6H) ppm. ^13^C NMR: (101 MHz, Chloroform-*d*) δ 156.2, 142.6, 135.6, 135.2, 129.8, 129.7, 128.0, 127.1, 126.4, 108.2, 55.9, 17.8 ppm.

#### Synthesis of (*S*)-5,5'-diphenyl-6,6'-dimethyl-[1,1'-biphenyl]-2,2'-diol ((*S*)-**L10**)

To a flamed-dried 100 mL flask which contained (*S*)-**20** (5 mmol, 1.97 g) was charged dry DCM. The solution was cooled to 0^o^C before adding a DCM (15 mL) solution of BBr_3_ (30 mmol, 2.9 mL) dropwise. After the addition completed, the reaction mixture was allowed to warm to room temperature and stirred for 12 h. Saturated aqueous NH_4_Cl solution was added to quench the reaction. The mixture was extracted with DCM, and the combined organic layer was washed with water, brine, dried over Na_2_SO_4_, filtered and concentrated. The residue was purified by a column chromatography (eluent: PE:EtOAc = 10:1) to afford the desired product (*S*)-**L10** as a white solid (1.57 g, 86% yield).

#### (*S*)-5,5'-Diphenyl-6,6'-dimethyl-[1,1'-biphenyl]-2,2'-diol ((*S*)-**L10**)

^1^H NMR: (400 MHz, Chloroform-*d*) δ 7.44 – 7.35 (m, 4H), 7.36 – 7.28 (m, 1H), 7.25 – 7.22 (m, 2H), 7.02 – 6.88 (m, 2H), 4.80 (s, 2H), 1.94 (s, 6H) ppm. ^13^C NMR: (101 MHz, Chloroform-*d*) δ 153.0, 141.8, 136.1, 135.9, 132.0, 129.6, 128.2, 126.8, 120.5, 113.2, 17.8 ppm. HRMS (ESI-TOF) *m*/*z* calcd for [C_26_H_23_O_2_] [M+H]^+^ 367.1693, found 367.1685.

#### Synthesis of (*S*)-2',2''-diethyl-4',6''-dimethoxy-1,1':3',1'':3'',1'''-quaterphenyl ((*S*)-**21**)

To a 100 mL three-neck round-bottom flask which contained (*S*)-**16** (2 mmol, 800 mg), PdCl_2_(PPh_3_)_2_ (0.3 mmol, 210 mg) and PhB(OH)_2_ (10 mmol, 1.2 g) was added DME (9 mL). After adding an aqueous (3 mL) solution of Na_2_CO_3_ (10 mmol, 1.06 g) via a syringe at room temperature under argon atmosphere, the mixture was reflux for 24 h. The reaction solution was cooled to room temperature and extracted with EtOAc, and the combined organic layer was washed with water, brine, dried over Na_2_SO_4_, filtered and concentrated. The residue was purified by a column chromatography (eluent: PE:EtOAc = 25:1) to afford the desired product (*S*)-**21** as a colorless gum (743 mg, 88% yield).

#### (*S*)-2',2''-Diethyl-4',6''-dimethoxy-1,1':3',1'':3'',1'''-quaterphenyl ((*S*)-**21**)

^1^H NMR: (400 MHz, Chloroform-*d*) δ 7.47 – 7.30 (m, 10H), 7.25 – 7.20 (m, 2H), 6.91 (d, J = 8.4 Hz, 2H), 3.78 (s, 6H), 2.44 (dq, J = 14.9, 7.4 Hz, 2H), 2.27 (dq, J = 14.8, 7.4 Hz, 2H), 0.73 (t, J = 7.5 Hz, 6H) ppm. ^13^C NMR: (101 MHz, Chloroform-*d*) δ 156.8, 142.9, 141.8, 135.1, 130.2, 129.8, 128.0, 126.5, 126.2, 107.9, 55.6, 24.2, 14.2 ppm.

#### Synthesis of (*S*)-5,5'-diphenyl-6,6'-diethyl-[1,1'-biphenyl]-2,2'-diol ((*S*)-**L11**)

To a flamed-dried 100 mL flask which contained (*S*)-**21** (5 mmol, 2.1 g) was charged dry DCM. The solution was cooled to 0^o^C before adding a DCM (15 mL) solution of BBr_3_ (30 mmol, 2.9 mL) dropwise. After the addition completed, the reaction mixture was allowed to warm to room temperature and stirred for 12 h. Saturated aqueous NH_4_Cl solution was added to quench the reaction. The mixture was extracted with DCM, and the combined organic layer was washed with water, brine, dried over Na_2_SO_4_, filtered and concentrated. The residue was purified by a column chromatography (eluent: PE:EtOAc = 10:1) to afford the desired product (*S*)-**L11** as a white solid (1.7 g, 86% yield).

#### (*S*)-5,5'-Diphenyl-6,6'-diethyl-[1,1'-biphenyl]-2,2'-diol ((*S*)-**L11**)

^1^H NMR: (400 MHz, Chloroform-*d*) δ 7.48 – 7.30 (m, 10H), 7.23 (d, J = 8.3 Hz, 2H), 6.98 (d, J = 8.3 Hz, 2H), 4.84 (s, 2H), 2.62 – 2.37 (m, 2H), 2.38 – 2.16 (m, 2H), 0.76 (t, J = 7.5 Hz, 6H) ppm. ^13^C NMR: (101 MHz, Chloroform-*d*) δ 153.3, 142.8, 142.0, 135.9, 132.6, 129.6, 128.2, 126.8, 119.5, 113.2, 24.4, 14.7 ppm. HRMS (ESI-TOF) *m*/*z* calcd for [C_28_H_27_O_2_] [M+H]^+^ 395.2011, found 395.2020.

#### General procedures for addition of diethylzinc to aldehydes (**22**) in the presence of biphenyldiols

A flame-dried 10 mL Schlenk tube equipped with a magnetic stirring bar was cooled to room temperature. To this flask was added (*S*)-**L2** (0.02 mmol, 4.8 mg), and evacuated and back-filled five times with argon. Then Ti(O-*i*Pr)_4_ (0.32 mmol, 95 μL) in dry toluene (1 mL) was added by syringe subsequently. The mixture was stirred at room temperature for approximately 10 min. Then, hexane solution of ZnEt_2_ (0.6 mL, 1 M) was added dropwise via a syringe at room temperature. After the addition completed, the mixture was stirred at room temperature for approximately 10 min. The mixture was cooled to -3^o^C and toluene (1 mL) solution of aldehyde (**22**) (0.2 mmol) was added dropwise by syringe. The reaction was stirred at -3^o^C for 10 h, then 1N HCl aqueous solution (2 mL) was added to quench the reaction and the mixture was stirred at room temperature for 0.5 h. The mixture was extracted with EtOAc, and the combined organic layer was washed with water, brine, dried over Na_2_SO_4_, filtered and concentrated. The residue was purified by a column chromatography (eluent: PE:EtOAc = 10:1) to afford the desired chiral alcohol (**23**).

#### (*S*)-1-Phenylpropan-1-ol (**23a**)

Product **23a**^59^ was obtained by silica column chromatography (PE:EtOAc = 10/1) as clear oil (26 mg, 97% yield, 94% ee). ^1^H NMR (400 MHz, Chloroform-*d*) δ 7.53 – 7.03 (m, 5H), 4.58 (t, J = 6.6 Hz, 1H), 1.97 (s, 1H), 1.78 (m 2H), 0.91 (t, J = 7.4 Hz, 3H) ppm. ^13^C NMR (101 MHz, Chloroform-*d*) δ 144.7, 128.5, 127.6, 126.1, 76.1, 32.0, 10.3 ppm.

#### (*S*)-1-(3-Methoxyphenyl)propan-1-ol (**23b**)

Product **23b**^59^ was obtained by silica column chromatography (PE:EtOAc = 10/1) as clear oil (31 mg, 95% yield, 95% ee). ^1^H NMR (400 MHz, Chloroform-*d*) δ 7.25 – 7.21 (m, 1H), 6.89 – 6.87 (m, 2H), 6.80 – 6.78 (m, 1H), 4.51 (t, J = 6.6 Hz, 1H), 3.78 (s, 3H), 2.30 (s, 1H), 1.83 – 1.66 (m, 2H), 0.89 (t, J = 7.4 Hz, 3H) ppm. ^13^C NMR (101 MHz, Chloroform-*d*) δ 159.7, 146.5, 129.4, 118.4, 113.0, 111.5, 75.9, 55.3, 31.9, 10.3 ppm.

#### (*S*)-1-(2-Methoxyphenyl)propan-1-ol (**23c**)

Product **25d**^59^ was obtained by silica column chromatography (PE:EtOAc = 10/1) as clear oil (31 mg, 95% yield). ^1^H NMR: (400 MHz, Chloroform-*d*) δ 7.30 (d, J = 7.5 Hz, 1H), 7.23 (t, J = 8.4 Hz, 1H), 6.95 (t, J = 7.5 Hz, 1H), 6.86 (d, J = 8.2 Hz, 1H), 4.79 (t, J = 6.3 Hz, 1H), 3.81 (s, 3H), 2.82 (s, 1H), 1.80 (p, J = 7.2 Hz, 2H), 0.94 (t, J = 7.4 Hz, 3H) ppm. ^13^C NMR: (101 MHz, Chloroform-*d*) δ 156.7, 132.6, 128.2, 127.1, 120.8, 110.6, 72.2, 55.3, 30.3, 10.6 ppm.

#### (*S*)-1-(3,4-Dimethoxyphenyl)propan-1-ol (**23d**)

Product **23d**^60^ was obtained by silica column chromatography (PE:EtOAc = 10/1) as clear oil (36 mg, 92% yield). ^1^H NMR: (400 MHz, Chloroform-*d*) δ 6.85 – 6.80 (m, 1H), 6.79 – 6.72 (m, 2H), 4.43 (t, J = 6.6 Hz, 1H), 3.90-3.70 (m, 6H), 2.31 (s, 1H), 1.79 – 1.59 (m, 2H), 0.83 (t, J = 7.4 Hz, 3H) ppm. ^13^C NMR: (101 MHz, Chloroform-*d*) δ 149.0, 148.3, 137.5, 118.3, 110.9, 109.1, 75.9, 56.0, 31.9, 10.3 ppm.

#### (*S*)-1-(4-Fluorophenyl)propan-1-ol (**23e**)

Product **23e**^59^ was obtained by silica column chromatography (PE:EtOAc = 10/1) as clear oil (30 mg, 98% yield, 93% ee). ^1^H NMR (400 MHz, Chloroform-*d*) δ 7.28 – 7.16 (m, 2H), 7.04 – 6.92 (m, 2H), 4.50 (t, J = 6.6 Hz, 1H), 2.47 (s,1H), 1.85 – 1.54 (m, 2H), 0.85 (t, J = 7.5 Hz, 3H) ppm. ^13^C NMR (101 MHz, Chloroform-*d*) δ 162.2 (d, J = 245.0 Hz), 140.4 (d, J = 3.1 Hz), 127.7 (d, J = 7.9 Hz), 115.2 (d, J = 21.3 Hz), 75.4, 32.0, 10.1 ppm. ^19^F NMR (282 MHz, Chloroform-*d*) δ-115.26 ppm.

#### (*S*)-1-(2-Fluorophenyl)propan-1-ol (**23f**)

Product **23f**^28^ was obtained by silica column chromatography (PE:EtOAc = 10/1) as clear oil (30 mg, 98% yield). ^1^H NMR: (400 MHz, Chloroform-*d*) δ 7.53 – 7.34 (m, 1H), 7.28 – 7.19 (m, 1H), 7.16 – 7.12 (m, 1H), 7.07 – 6.92 (m, 1H), 4.93 (q, J = 5.7 Hz, 1H), 1.99 (s, 1H), 1.87 – 1.76 (m, 2H), 0.96 – 0.92 (m, 3H) ppm. ^13^C NMR: (101 MHz, Chloroform-*d*) δ 160.0 (d, J = 245.2 Hz), 131.6 (d, J = 13.5 Hz), 128.8 (d, J = 8.3 Hz), 127.4 (d, J = 4.4 Hz), 124.3 (d, J = 3.1 Hz), 115.3 (d, J = 22.0 Hz), 69.8, 31.0, 10.1 ppm. ^19^F NMR: (282 MHz, Chloroform-*d*) δ-119.66 ppm.

#### (*S*)-1-(4-Chlorophenyl)propan-1-ol (**23g**)

Product **23g**^28^ was obtained by silica column chromatography (PE:EtOAc = 10/1) as clear oil (32 mg, 94% yield, 93% ee). ^1^H NMR (400 MHz, Chloroform-*d*) δ 7.30 – 7.24 (m, 2H), 7.21 – 7.19 (m, 2H), 4.48 (t, J = 6.6 Hz, 1H), 2.61 (s, 1H), 1.79 – 1.57 (m, 2H), 0.85 (t, J = 7.5 Hz, 3H) ppm. ^13^C NMR (101 MHz, Chloroform-*d*) δ 143.1, 133.1, 128.5, 127.5, 75.3, 32.0, 10.1 ppm.

#### (*S*)-1-(4-Bromophenyl)propan-1-ol (23h)

Product **23h**^28^ was obtained by silica column chromatography (PE:EtOAc = 10/1) as clear oil (41 mg, 96% yield, 92% ee). ^1^H NMR (400 MHz, Chloroform-*d*) δ 7.45 – 7.41 (m, 2H), 7.17 – 7.13 (m, 2H), 4.48 (t, J = 6.6 Hz, 1H), 2.61 (s, 1H), 1.89 – 1.51 (m, 1H), 0.86 (t, J = 7.4 Hz, 3H) ppm. ^13^C NMR (101 MHz, Chloroform-*d*) δ 143.6, 131.5, 127.8, 121.2, 75.3, 31.9, 10.1 ppm.

#### (*S*)-1-(3-Bromophenyl)propan-1-ol (**23i**)

Product **23i**^28^ was obtained by silica column chromatography (PE:EtOAc = 10/1) as yellow oil (40 mg, 95% yield). ^1^H NMR: (400 MHz, Chloroform-*d*) δ 7.48 (s, 1H), 7.39 – 7.38 (m, 1H), 7.30 – 7.12 (m, 2H), 4.53 (t, J = 6.5 Hz, 1H), 2.22 (s, 1H), 1.82 – 1.64 (m, 2H), 0.89 (t, J = 7.4 Hz, 3H) ppm. ^13^C NMR: (101 MHz, Chloroform-*d*) δ 147.0, 130.6, 130.1, 129.2, 124.7, 122.6, 75.3, 32.0, 10.1 ppm.

#### (*S*)-4-(1-Hydroxypropyl)benzonitrile (**23j**)

Product **23j**^61^ was obtained by silica column chromatography (PE:EtOAc = 10/1) as clear oil (32 mg, 99% yield). ^1^H NMR: (400 MHz, Chloroform-*d*) δ 7.61 (d, J = 8.2 Hz, 2H), 7.44 (d, J = 8.4 Hz, 2H), 4.67 (t, J = 6.4 Hz, 1H), 2.24 (s, 1H), 1.88 – 1.62 (m, 2H), 0.91 (t, J = 7.4 Hz, 3H) ppm. ^13^C NMR: (101 MHz, Chloroform-*d*) δ 150.1, 132.3, 126.7, 119.0, 111.1, 75.1, 32.1, 9.9 ppm.

#### (*S*)-3-(1-Hydroxypropyl)benzonitrile (**23k**)

Product **23k**^61^ was obtained by silica column chromatography (PE:EtOAc = 10/1) as clear oil (30 mg, 95% yield). ^1^H NMR: (400 MHz, Chloroform-*d*) δ 7.58 (s, 1H), 7.55 – 7.46 (m, 2H), 7.42 – 7.38 (m, 1H), 4.59 (t, J = 6.5 Hz, 1H), 2.74 (s, 1H), 1.78 – 1.63 (m, 2H), 0.86 (t, J = 7.4 Hz, 3H) ppm. ^13^C NMR: (101 MHz, Chloroform-*d*) δ 146.3, 131.0, 130.6, 129.7, 129.2, 119.0, 112.2, 74.7, 32.1, 9.9 ppm.

#### (*S*)-1-(4-Fluoro-3-(trifluoromethyl)phenyl)propan-1-ol (**23l**)

Product **23l** was obtained by silica column chromatography (PE:EtOAc = 10/1) as clear oil (42 mg, 95% yield). ^1^H NMR: (400 MHz, Chloroform-*d*) δ 7.79 (d, *J* = 8.0 Hz, 1H), 7.58 – 7.46 (m, 1H), 7.12 (t, *J* = 9.2 Hz, 1H), 4.98 (q, *J* = 6.1 Hz, 1H), 2.13 (d, *J* = 4.0 Hz, 1H), 1.80 (p, *J* = 7.2 Hz, 2H), 0.96 (t, *J* = 7.4 Hz, 3H) ppm. ^13^C NMR: (101 MHz, Chloroform-*d*) δ 161.5 (d, *J* = 251.1 Hz), 132.7 (d, *J* = 14.6 Hz), 127.0 (q, *J* = 33.1 Hz), 126.2 (dq, *J* = 7.3, 3.4 Hz), 125.1 (dt, *J* = 8.0, 3.6 Hz), 123.9 (q, *J* = 272.0 Hz), 115.9 (d, *J* = 23.7 Hz), 69.2, 31.1, 9.8 ppm. ^19^F NMR: (282 MHz, Chloroform-*d*) δ-61.88, -114.13 ppm.

#### (*S*)-1-(Naphthalen-2-yl)propan-1-ol (**23m**)

Product **23m**^28^ was obtained by silica column chromatography (PE:EtOAc = 10/1) as clear oil (37 mg, 99% yield). ^1^H NMR: (400 MHz, Chloroform-*d*) δ 7.85 – 7.80 (m, 3H), 7.74 (s, 1H), 7.54 – 7.40 (m, 3H), 4.71 (t, J = 6.6 Hz, 1H), 2.46 (s, 1H), 2.46 – 1.77 (m, 2H), 0.94 (t, J = 7.5 Hz, 3H) ppm. ^13^C NMR: (101 MHz, Chloroform-*d*) δ 142.1, 133.4, 1331, 128.3, 128.1, 127.8, 126.2, 125.9, 124.9, 124.3, 76.2, 31.9, 10.3 ppm.

#### (*S*)-1-Phenylpentan-3-ol (**23n**)

Product **23n**^61^ was obtained by silica column chromatography (PE:EtOAc = 10/1) as clear oil (27 mg, 84% yield). ^1^H NMR: (400 MHz, Chloroform-*d*) δ 7.31 – 7.24 (m, 2H), 7.23 – 7.16 (m, 3H), 3.57 – 3.51 (m, 1H), 2.83 – 2.75 (m, 1H), 2.70 – 2.62 (m, 1H), 1.83 – 1.67 (m, 2H), 1.60 (s, 1H), 1.58 – 1.41 (m, 2H), 0.94 (t, J = 7.4 Hz, 3H) ppm. ^13^C NMR: (101 MHz, Chloroform-*d*) δ 142.1, 128.3, 128.3, 125.7, 72.5, 38.5, 32.0, 30.2, 9.7 ppm.

#### (*S*)-1-(4-Methoxyphenyl)propan-1-ol (**23o**)

Product **23o**^28^ was obtained by silica column chromatography (PE:EtOAc = 10/1) as clear oil (31 mg, 95% yield). ^1^H NMR: (400 MHz, Chloroform-*d*) δ 7.24 – 7.21 (m, 2H), 6.86 – 6.83 (m, 2H), 4.48 (t, J = 6.7 Hz, 1H), 3.77 (s, 3H), 2.24 (s, 1H), 2.24 – 1.62 (m, 2H), 0.86 (t, J = 7.5 Hz, 3H) ppm. ^13^C NMR: (101 MHz, Chloroform-*d*) δ 159.0, 136.9, 127.3, 113.8, 75.7, 55.3, 31.9, 10.3 ppm.

#### (*R*)-1-(2-Chlorophenyl)propan-1-ol (**23p**)

Product **23p**^61^ was obtained by silica column chromatography (PE:EtOAc = 10/1) as clear oil (33 mg, 98% yield). ^1^H NMR: (400 MHz, Chloroform-*d*) δ 7.54 – 7.52 (m, 1H), 7.35 – 7.24 (m, 2H), 7.21 – 7.17 (m, 1H), 7.07 – 7.04 (m, 1H), 2.10 (s, 1H), 1.90 – 1.64 (m, 2H), 0.98 (t, J = 7.4 Hz, 3H) ppm. ^13^C NMR: (101 MHz, Chloroform-*d*) δ 142.2, 132.2, 129.6, 128.6, 127.4, 127.2, 72.2, 30.7, 10.3 ppm.

#### (*S*)-1-(Thiophen-2-yl)propan-1-ol (**23q**)

Product **23q**^28^ was obtained by silica column chromatography (PE:EtOAc = 10/1) as clear oil (27 mg, 98% yield). ^1^H NMR: (400 MHz, Chloroform-*d*) δ 7.22 – 7.21 (m, 1H), 7.01 – 6.89 (m, 2H), 4.78 (t, J = 6.6 Hz, 1H), 2.55 (s, 1H), 1.94 – 1.72 (m, 2H), 0.93 (t, J = 7.5 Hz, 3H) ppm. ^13^C NMR: (101 MHz, Chloroform-*d*) δ 148.8, 126.7, 124.5, 123.9, 71.8, 32.3, 10.3 ppm.

#### (*S*,*E*)-1-Phenylpent-1-en-3-ol (**23r**)

Product **23r**^61^ was obtained by silica column chromatography (PE:EtOAc = 10/1) as clear oil (27 mg, 85% yield). ^1^H NMR: (400 MHz, Chloroform-*d*) δ 7.39 – 7.37 (m, 2H), 7.33 – 7.29 (m, 2H), 7.27 – 7.21 (m, 1H), 6.57 (d, J = 15.9 Hz, 1H), 6.24 – 6.18 (m, 1H), 4.20 (q, J = 6.5 Hz, 1H), 1.81 (s, 1H), 1.72 – 1.58 (m, 2H), 0.97 (t, J = 7.5 Hz, 3H) ppm. ^13^C NMR: (101 MHz, Chloroform-*d*) δ 136.8, 132.4, 130.5, 128.7, 127.7, 126.6, 74.5, 30.3, 9.9 ppm.

#### General procedures for addition of alkynes to aldehydes (**22**) in the presence of ZnMe_2_ and biphenyldiols

A flame-dried 10 mL Schlenk tube equipped with a magnetic stirring bar was cooled to room temperature. To this flask was added (*S*)-**L5** (0.1 mmol, 37 mg) and evacuated and back-filled five times with argon. Then Ti(O-*i*Pr)_4_ (0.125 mmol, 37 μL) in dry DCM (1 mL) was added by syringe subsequently. The mixture was stirred at room temperature for approximately 15 min to prepare the titanium complex. To another dry Schlenk tube was charged with alkyne (1.25 mmol), evacuated and back-filled five times with argon. Then a solution of 1M ZnMe_2_ in toluene (1 mmol, 1 mL) was added by syringe at 0^o^C with continued stirring for 15 min. The titanium complex was added via a syringe and the homogenous solution was stirred at 0^o^C for 15 min. A solution of aldehyde (**22**) (0.5 mmol) in DCM (1 mL) was added and the mixture was allowed to stir at 0°C for 36 h, then 1N HCl aqueous solution (2 mL) was added to quench the reaction and the mixture was stirred at room temperature for 0.5 h. The mixture was extracted with DCM, and the combined organic layer was washed with water, brine, dried over Na_2_SO_4_, filtered and concentrated. The residue was purified by a column chromatography (eluent: PE:EtOAc = 10:1) to afford the desired chiral propargylic alcohol (**25**).

#### (*R*)-1,3-Diphenylprop-2-yn-1-ol (**25a**)

Product **25a**^62^ was obtained by silica column chromatography (PE:EtOAc = 10/1) as clear oil (88 mg, 85% yield, 93% ee). ^1^H NMR (400 MHz, Chloroform-*d*) δ 7.66 – 7.59 (m, 2H), 7.50 – 7.45 (m, 2H), 7.43 – 7.38 (m, 2H), 7.37 – 7.28 (m, 4H), 5.69 (s, 1H), 2.36 (s, 1H) ppm. ^13^C NMR (101 MHz, Chloroform-*d*) δ 140.7, 131.9, 128.8, 128.7, 128.6, 128.4, 126.9, 122.5, 88.8, 86.8, 65.2 ppm.

#### (*S*)-1-(2-Methoxyphenyl)-3-phenylprop-2-yn-1-ol (**25b**)

Product **25b**^63^ was obtained by silica column chromatography (PE:EtOAc = 7/1) as yellow solid (98 mg, 83% yield, 90% ee). ^1^H NMR (400 MHz, Chloroform-*d*) δ 7.68 – 7.66 (m, 1H), 7.51 – 7.48 (m, 2H), 7.38 – 7.27 (m, 4H), 7.01 (t, J = 7.5 Hz, 1H), 6.93 (d, J = 8.2 Hz, 1H), 5.96 (d, J = 6.0 Hz, 1H), 3.90 (s, 3H), 3.23 (d, J = 6.1 Hz, 1H) ppm. ^13^C NMR (101 MHz, Chloroform-*d*) δ 157.0, 131.9, 129.9, 128.9, 128.5, 128.4, 128.2, 122.9, 121.0, 111.0, 88.5, 86.2, 61.7, 55.7 ppm.

#### (*R*)-1-(3-Methoxyphenyl)-3-phenylprop-2-yn-1-ol (**25c**)

Product **25c**^63^ was obtained by silica column chromatography (PE:EtOAc = 10/1) as yellow solid (106 mg, 88% yield, 97% ee). ^1^H NMR: (400 MHz, Chloroform-*d*) δ 7.53 – 7.44 (m, 2H), 7.37 – 7.27 (m, 4H), 7.25 – 7.16 (m, 2H), 6.92 – 6.86 (m, 1H), 5.67 (d, J = 5.8 Hz, 1H), 3.80 (s, 3H), 3.16 (d, J = 6.0 Hz, 1H) ppm. ^13^C NMR: (101 MHz, Chloroform-*d*) δ 159.9, 142.4, 131.9, 129.8, 128.7, 128.5, 122.6, 119.2, 114.2, 112.3, 89.0, 86.6, 65.0, 55.1 ppm.

#### (*R*)-1-(4-Methoxyphenyl)-3-phenylprop-2-yn-1-ol (**25d**)

Product **25d**^63^ was obtained by silica column chromatography (PE:EtOAc = 8/1) as yellow solid (108 mg, 91% yield, 94% ee). ^1^H NMR (400 MHz, Chloroform-*d*) δ 7.61 – 7.43 (m, 4H), 7.33 – 7.21 (m, 3H), 6.93 – 6.90 (m, 2H), 5.64 (s, 1H), 3.79 (s, 3H), 2.88 (s, 1H) ppm. ^13^C NMR (101 MHz, Chloroform-*d*) δ 159.7, 133.2, 131.9, 128.7, 128.5, 128.4, 122.7, 114.1, 89.2, 86.5, 64.7, 55.5 ppm.

#### (*S*)-1-(2-Fluorophenyl)-3-phenylprop-2-yn-1-ol (**25e**)

Product **25e**^64^ was obtained by silica column chromatography (PE:EtOAc = 10/1) as clear oil (106 mg, 94% yield). ^1^H NMR: (400 MHz, Chloroform-*d*) δ 7.77 – 7.72 (m, 1H), 7.55 – 7.40 (m, 2H), 7.39 – 7.26 (m, 4H), 7.22 – 7.15 (m, 1H), 7.13 – 7.04 (m, 1H), 5.98 (d, J = 5.5 Hz, 1H), 2.79 (d, J = 4.6 Hz, 1H) ppm. ^13^C NMR: (101 MHz, Chloroform-*d*) δ 160.3 (d, J = 248.4 Hz), 131.9, 130.4 (d, J = 8.4 Hz), 128.8, 128.6 (d, J = 3.4 Hz), 128.4, 128.0 (d, J = 12.9 Hz), 124.5 (d, J = 3.6 Hz), 122.4, 115.8 (d, J = 21.2 Hz), 87.8, 86.7, 59.6 ppm. ^19^F NMR: (282 MHz, Chloroform-*d*) δ-118.87 ppm.

#### (*R*)-1-(4-Fluorophenyl)-3-phenylprop-2-yn-1-ol (**25f**)

Product **25f**^63^ was obtained by silica column chromatography (PE:EtOAc = 10/1) as clear oil (107 mg, 95% yield, 91% ee). ^1^H NMR (400 MHz, Chloroform-*d*) δ 7.63 – 7.54 (m, 2H), 7.50 – 7.41 (m, 2H), 7.37 – 7.27 (m, 3H), 7.13 – 7.01 (m, 2H), 5.67 (d, J = 5.2 Hz, 1H), 2.32 (d, J = 5.6 Hz, 1H) ppm. ^13^C NMR (101 MHz, Chloroform-*d*) δ 162.8 (d, J = 246.7 Hz), 136.6 (d, J = 2.8 Hz), 131.9, 128.8 (d, J = 7.4 Hz), 128.7, 128.5, 122.4, 115.6 (d, J = 21.6 Hz), 88.8, 86.9, 64.4 ppm. ^19^F NMR: (282 MHz, Chloroform-*d*) δ-113.49 ppm.

#### (*R*)-1-(4-Chlorophenyl)-3-phenylprop-2-yn-1-ol (**25g**)

Product **25g**^63^ was obtained by silica column chromatography (PE:EtOAc = 8/1) as clear oil (113 mg, 94% yield). ^1^H NMR: (400 MHz, Chloroform-*d*) δ 7.53 – 7.51 (m, 2H), 7.48 – 7.42 (m, 2H), 7.38 – 7.27 (m, 5H), 5.64 (s, 1H), 2.87 (s, 1H) ppm. ^13^C NMR: (101 MHz, Chloroform-*d*) δ 139.2, 134.3, 131.9, 128.9, 128.5, 128.3, 122.3, 88.4, 87.1, 64.4 ppm.

#### (*R*)-1-(4-Bromophenyl)-3-phenylprop-2-yn-1-ol (**25h**)

Product **25h**^62^ was obtained by silica column chromatography (PE:EtOAc = 10/1) as yellow solid (128 mg, 90% yield). ^1^H NMR: (400 MHz, Chloroform-*d*) δ 7.55 – 7.40 (m, 6H), 7.38 – 7.25 (m, 3H), 5.63 (s, 1H), 2.95 (s, 1H) ppm. ^13^C NMR: (101 MHz, Chloroform-*d*) δ 139.7, 131.9, 131.8, 128.9, 128.6, 128.5, 122.5, 122.2, 88.4, 87.1, 64.5 ppm.

#### (*R*)-3-(1-Hydroxy-3-phenylprop-2-yn-1-yl)benzonitrile (**25i**)

Product **25i**^63^ was obtained by silica column chromatography (PE:EtOAc = 5/1) as white solid (106 mg, 91% yield). ^1^H NMR: (400 MHz, Chloroform-*d*) δ 7.90 (s, 1H), 7.85 – 7.83 (m, 1H), 7.61 – 7.59 (m, 1H), 7.52 – 7.41 (m, 3H), 7.38 – 7.28 (m, 3H), 5.71 (s, 1H), 2.90 (s, 1H) ppm. ^13^C NMR: (101 MHz, Chloroform-*d*) δ 142.3, 132.0, 131.9, 131.4, 130.4, 129.6, 129.1, 128.5, 121.9, 118.8, 112.5, 87.7, 87.5, 64.0 ppm.

#### (*R*)-4-(1-Hydroxy-3-phenylprop-2-yn-1-yl)benzonitrile (**25j**)

Product **25j**^63^ was obtained by silica column chromatography (PE:EtOAc = 5/1) as white solid (111 mg, 96% yield). ^1^H NMR: (400 MHz, Chloroform-*d*) δ 7.75 – 7.59 (m, 4H), 7.45 – 7.43 (m, 2H), 7.36 – 7.29 (m, 3H), 5.73 (d, J = 5.7 Hz, 1H), 3.46 – 2.15 (m, 1H) ppm. ^13^C NMR: (101 MHz, Chloroform-*d*) δ 145.7, 132.6, 131.9, 129.1, 128.3, 127.4, 121.9, 118.8, 112.1, 87.6, 87.6, 64.3 ppm.

#### (*R*)-1-(3-Nitrophenyl)-3-phenylprop-2-yn-1-ol (**25k**)

Product **25k**^62^ was obtained by silica column chromatography (PE:EtOAc = 10/1) as yellow solid (107 mg, 85% yield). ^1^H NMR: (400 MHz, Chloroform-*d*) δ 8.47 – 8.45 (m, 1H), 8.23 – 8.10 (m, 1H), 7.94 – 7.92 (m, 1H), 7.56 – 7.52 (m, 1H), 7.48 – 7.41 (m, 2H), 7.38 – 7.27 (m, 1H), 5.78 (s, 1H), 2.94 (s, 1H). ^13^C NMR: (101 MHz, Chloroform-*d*) δ 148.4, 142.7, 132.9, 131.9, 129.7, 129.2, 128.5, 123.4, 121.8, 87.7, 87.5, 64.0 ppm.

#### (*R*)-1-(Naphthalen-2-yl)-3-phenylprop-2-yn-1-ol (**25l**)

Product **25l**^62^ was obtained by silica column chromatography (PE:EtOAc = 10/1) as yellow solid (122 mg, 95% yield). ^1^H NMR: (400 MHz, Chloroform-*d*) δ 8.05 (s, 1H), 7.89 – 7.84 (m, 3H), 7.75 – 7.73 (m, 1H), 7.55 – 7.50 (m, 4H), 7.39 – 7.29 (m, 3H), 5.86 (d, J = 5.9 Hz, 1H), 2.82 (d, J = 5.8 Hz, 1H) ppm. ^13^C NMR: (101 MHz, Chloroform-*d*) δ 138.2, 133.4, 133.3, 132.0, 128.8, 128.8, 128.5, 128.4, 127.9, 126.5, 125.7, 124.9, 122.6, 89.0, 87.0, 65.3 ppm.

#### (*R*)-3-(2-Methoxyphenyl)-1-(naphthalen-2-yl)prop-2-yn-1-ol (**25m**)

Product **25m**^63^ was obtained by silica column chromatography (PE:EtOAc = 10/1) as yellow solid (116 mg, 81% yield). ^1^H NMR: (400 MHz, Chloroform-*d*) δ 8.14 (s, 1H), 7.93 – 7.76 (m, 4H), 7.58 – 7.44 (m, 3H), 7.34 – 7.26 (m, 1H), 6.93 (t, J = 7.5 Hz, 1H), 6.83 (d, J = 8.4 Hz, 1H), 5.97 (s, 1H), 3.85 (s, 1H), 3.82 (s, 3H) ppm. ^13^C NMR: (101 MHz, Chloroform-*d*) δ 160.3, 138.3, 133.8, 133.4, 130.2, 128.6, 128.4, 127.8, 126.3, 125.9, 125.1, 120.6, 111.8, 110.8, 93.2, 83.4, 65.4, 55.9 ppm.

#### (*R*)-3-(4-Methoxyphenyl)-1-(naphthalen-2-yl)prop-2-yn-1-ol (**25n**)

Product **25n**^63^ was obtained by silica column chromatography (PE:EtOAc = 10/1) as yellow solid (129 mg, 90% yield). ^1^H NMR: (400 MHz, Chloroform-*d*) δ 8.04 (s, 1H), 7.89 – 7.83 (m, 3H), 7.73 – 7.71 (m, 1H), 7.53 – 7.46 (m, 2H), 7.45 – 7.38 (m, 2H), 6.90 – 6.77 (m, 2H), 5.84 (s, 1H), 3.80 (s, 3H), 2.45 (s, 1H) ppm. ^13^C NMR: (101 MHz, Chloroform-*d*) δ 160.0, 138.3, 133.4, 1333, 128.7, 128.3, 127.8, 126.4, 125.6, 124.8, 114.6, 114.1, 87.5, 87.0, 65.4, 55.4 ppm.

#### (*R*)-3-(4-Chlorophenyl)-1-(naphthalen-2-yl)prop-2-yn-1-ol (**25o**)

Product **25o**^63^ was obtained by silica column chromatography (PE:EtOAc = 10/1) as white solid (134 mg, 92% yield). ^1^H NMR: (400 MHz, Chloroform-*d*) δ 8.02 (s, 1H), 7.92 – 7.81 (m, 3H), 7.71 – 7.69 (m, 1H), 7.53 – 7.48 (m, 2H), 7.43 – 7.37 (m, 2H), 7.32 – 7.26 (m, 2H), 5.84 (d, J = 5.9 Hz, 1H), 2.48 (d, J = 6.0 Hz, 1H) ppm. ^13^C NMR: (101 MHz, Chloroform-*d*) δ 137.8, 134.8, 133.4, 133.3, 133.1, 128.8, 128.3, 127.8, 126.5, 126.5, 125.6, 124.6, 121.0, 89.7, 85.9, 65.4 ppm.

#### (*R*)-3-(3-Chlorophenyl)-1-(naphthalen-2-yl)prop-2-yn-1-ol (**25p**)

Product **25p**^62^ was obtained by silica column chromatography (PE:EtOAc = 10/1) as white solid (132 mg, 91% yield). ^1^H NMR: (400 MHz, Chloroform-*d*) δ 8.01 (s, 1H), 7.89 – 7.83 (m, 3H), 7.71 – 7.68 (m, 1H), 7.54 – 7.45 (m, 3H), 7.37 – 7.34 (m, 1H), 7.33 – 7.30 (m, 1H), 7.25 – 7.21 (m, 1H), 6.09 – 5.29 (m, 1H), 2.88 – 2.33 (m, 1H) ppm. ^13^C NMR: (101 MHz, Chloroform-*d*) δ 137.7, 134.3, 133.4, 133.3, 131.8, 130.0, 129.7, 129.1, 128.8, 128.4, 127.9, 126.6, 126.5, 125.6, 124.6, 124.2, 90.0, 85.6, 65.3 ppm.

#### (*R*)-1-(Naphthalen-2-yl)-3-(4-(trifluoromethyl)phenyl)prop-2-yn-1-ol (**25q**)

Product **25q**^62^ was obtained by silica column chromatography (PE:EtOAc = 10/1) as white solid (153 mg, 94% yield). ^1^H NMR: (400 MHz, Chloroform-*d*) δ 8.03 (s, 1H), 7.94 – 7.78 (m, 3H), 7.71 – 7.69 (m, 1H), 7.58 – 7.55 (m, 4H), 7.53 – 7.48 (m, 2H), 5.86 (d, J = 5.8 Hz, 1H), 2.50 (d, J = 9.3 Hz, 1H) ppm. ^13^C NMR: (101 MHz, Chloroform-*d*) δ 137.6, 133.4, 133.3, 132.1, 130.48 (d, J = 32.7 Hz), 128.9, 128.3, 127.9, 126.6, 126.6, 126.3, 125.7, 125.4 (d, J = 3.5 Hz), 124.6, 122.6, 91.2, 85.6, 65.3 ppm. ^19^F NMR: (282 MHz, Chloroform-*d*) δ-62.73 ppm.

#### (*S*)-3-Phenyl-1-(thiophen-2-yl)prop-2-yn-1-ol (**25r**)

Product **25r**^65^ was obtained by silica column chromatography (PE:EtOAc = 10/1) as red solid (84 mg, 79% yield). ^1^H NMR: (400 MHz, Chloroform-*d*) δ 7.56 – 7.43 (m, 2H), 7.37 – 7.29 (m, 4H), 7.25 – 7.24 (m, 1H), 7.06 – 6.91 (m, 1H), 5.89 (d, J = 6.9 Hz, 1H), 2.74 (d, J = 6.9 Hz, 1H) ppm. ^13^C NMR: (101 MHz, Chloroform-*d*) δ 144.8, 131.9, 128.9, 128.5, 126.9, 126.3, 125.8, 122.2, 88.1, 86.2, 60.9 ppm.

#### (*R*,*E*)-1,5-Diphenylpent-1-en-4-yn-3-ol (**25s**)

Product **25s**^66^ was obtained by silica column chromatography (PE:EtOAc = 10/1) as light yellow solid (108 mg, 93% yield). ^1^H NMR: (400 MHz, Chloroform-*d*) δ 7.53 – 7.48 (m, 2H), 7.45 – 7.40 (m, 2H), 7.38 – 7.26 (m, 6H), 6.84 (d, J = 15.9 Hz, 1H), 6.44 – 6.38 (m, 1H), 5.29 (d, J = 5.9 Hz, 1H), 2.33 (s, 1H) ppm. ^13^C NMR: (101 MHz, Chloroform-*d*) δ 136.2, 132.2, 131.9, 128.8, 128.5, 128.3, 128.1, 127.0, 122.5, 88.0, 86.6, 63.6 ppm.

#### (*R*)-1-(3-Bromophenyl)-3-phenylprop-2-yn-1-ol (**25t**)

Product **25t**^67^ was obtained by silica column chromatography (PE:EtOAc = 10/1) as clear oil (131 mg, 92% yield). ^1^H NMR: (400 MHz, Chloroform-*d*) δ 7.76 (s, 1H), 7.58 – 7.50 (m, 1H), 7.51 – 7.42 (m, 3H), 7.37 – 7.21 (m, 4H), 5.65 (d, J = 4.6 Hz, 1H), 2.38 (d, J = 5.1 Hz, 1H) ppm. ^13^C NMR: (101 MHz, Chloroform-*d*) δ 142.9, 131.9, 131.6, 130.3, 129.9, 128.9, 128.5, 125.4, 122.8, 122.2, 88.1, 87.2, 64.5 ppm.

#### Experimental procedures for synthesis of (*S*)-**L14**, **L15** and **L16**

A flame-dried 25 mL, two-necked flask was equipped with vacuum/argon stopcock and a magnetic stirring bar. The flask was charged with toluene (5 mL) and PCl_3_ (175 μL, 2 mmol), and then cooled to 0^o^C. Another flame-dried, 10 mL flask was charged with bis [(*R*)-1-phenylethyl] amine **26** (228 μL, 2 mmol), toluene (5 mL), and Et_3_N (280 μL, 2 mmol). This mixture was added dropwise to the above mentioned PCl_3_ solution at 0^o^C. After the addition was complete, the reaction mixture was heated at 80^o^C for 6 h, and then cooled to -78^o^C. To this flask at -78^o^C, Et_3_N (560 μL, 4 mmol) was added dropwise and then a solution of **(*S*)-L2** (242 mg, 1 mmol) in toluene (5 mL) and THF (3 mL) was added slowly. The reaction mixture was concentrated in vacuo, and the residue was purified by silica gel column chromatography (eluent: PE:EtOAc = 50:1) to afford (*S*)-**L15** as a white foam (420 mg, 90% yield). (*S*)**-L14** was prepared as a white solid (410 mg, 83% yield) from (*S*)-**L1** according to the same procedure for the preparation of (*S*)-**L15**. (*S*)-**L16** was prepared as a colorless gum (371 mg, 71% yield) from (*S*)-**L3** according to the same procedure for the preparation of (*S*)-**L15**.

(11*aS*)-1,11-Dimethyl-*N*,*N*-bis((*R*)-1-phenylethyl)dibenzo[*d*,*f*][1,3,2]-dioxaphosphepin-6-amine ((*S*)-**L14**)

^1^H NMR: (400 MHz, Chloroform-*d*) δ 7.31 – 7.24 (m, 2H), 7.21 – 7.10 (m, 12H), 7.09 – 7.01 (m, 2H), 4.49 (dq, J = 14.0, 7.0 Hz, 2H), 2.24 (s, 3H), 2.19 (s, 3H), 1.71 (d, J = 7.1 Hz, 6H) ppm. ^13^C NMR: (101 MHz, Chloroform-*d*) δ 151.6, 151.3, 151.2, 143.28, 138.7, 138.2, 130.3, 130.2, 128.5, 128.1, 128.1, 128.0, 127.8, 126.9, 126.7, 125.6, 119.4, 119.4, 119.0, 52.2, 52.1, 22.2, 22.1, 20.1, 19.8 ppm. ^31^P NMR: (240 MHz, Chloroform-*d*) δ 141.84 ppm. HRMS (ESI-TOF) *m*/*z* calcd for [C_30_H_31_NO_2_P] [M+H]^+^ 468.2092, found 468.2015.

#### (11*aS*)-1,11-Diethyl-*N*,*N*-bis((*R*)-1-phenylethyl)dibenzo[*d*,*f*][1,3,2]dioxaphosphepin-6-amine ((*S*)-**L15**)

^1^H NMR: (400 MHz, Chloroform-*d*) δ 7.32 – 7.23 (m, 3H), 7.17 – 7.02 (m, 12H), 7.01 – 6.94 (m, 2H), 4.43 (dq, J = 14.1, 7.1 Hz, 2H), 2.63 – 2.33 (m, 4H), 1.74 – 1.65 (m, 6H), 0.92 (dt, J = 10.8, 7.5 Hz, 6H) ppm. ^13^C NMR: (101 MHz, Chloroform-*d*) δ 151.5, 151.2, 151.2, 144.7, 144.5, 143.3, 129.1, 128.8, 128.3, 128.0, 128.0, 127.8, 127.3, 126.6, 125.0, 123.8, 119.4, 119.4, 119.0, 52.1, 52.0, 26.7, 26.3, 22.1, 15.9, 15.4 ppm. ^31^P NMR: (240 MHz, Chloroform-*d*) δ 141.92 ppm. HRMS (ESI-TOF) *m*/*z* calcd for [C_32_H_35_NO_2_P] [M+H]^+^ 496.2405, found 496.2421.

#### (11*aS*)-1,11-Diisopropyl-*N*,*N*-bis((*R*)-1-phenylethyl)dibenzo[*d*,*f*][1,3,2]-dioxaphosphepin-6-amine ((*S*)-**L16**)

^1^H NMR: (400 MHz, Chloroform-*d*) δ 7.37 – 7.27 (m, 2H), 7.21 – 7.01 (m, 13H), 6.99 – 6.94 (m, 1H), 4.41 (dq, J = 14.1, 7.1 Hz, 2H), 2.92 (dp, J = 41.4, 6.8 Hz), 1.72 – 1.58 (m, 6H), 1.42 – 1.12 (m, 12H) ppm. ^13^C NMR: (101 MHz, Chloroform-*d*) δ 151.4, 151.0, 149.2, 148.9, 143.3, 129.0, 128.5, 128.0, 127.8, 126.6, 124.5, 123.6, 121.4, 120.0, 119.4, 119.1, 52.0, 31.6, 30.2, 30.0, 29.7, 26.2, 25.9, 22.1, 21.2, 21.0 ppm. ^31^P NMR: (240 MHz, Chloroform-*d*) δ 141.85 ppm. HRMS (ESI-TOF) *m*/*z* calcd for [C_34_H_39_NO_2_P] [M+H]^+^ 524.2718, found 524.2731.

#### Experimental procedure for synthesis of (*S*)-**L18**

A flame-dried 25 mL, two-necked flask was equipped with vacuum/argon stopcock and a magnetic stirring bar. The flask was charged with toluene (5 mL) and PCl_3_ (525 μL, 6 mmol), and then cooled to 0^o^C. Another flame-dried, 10 mL flask was charged with *N*-benzhydrylaniline **28** (518 mg, 2 mmol), toluene (5 mL), and Et_3_N (860 μL, 6.2 mmol). The mixture was added dropwise to the above mentioned PCl_3_ solution at 0^o^C. After the addition was complete, the reaction mixture was heated at 80^o^C for 6 h, and then cooled to -78^o^C. To this flask at -78^o^C, Et_3_N (1.7 mL, 12 mmol) was added dropwise and then a solution of (S)-**L2** (726 mg, 3 mmol) in toluene (10 mL) and THF (5 mL) was added slowly. The reaction mixture was concentrated in vacuo, and the residue was purified by silica gel column chromatography (eluent: PE:EtOAc = 50:1) to afford (*S*)-**L18** as a colorless gum (888 mg, 56% yield).

#### (11*aS*)-*N*-Benzhydryl-1,11-diethyl-*N*-phenyldibenzo[*d*,*f*][1,3,2]dioxaphosphepin-6-amine ((*S*)-**L18**)

^1^H NMR: (400 MHz, Chloroform-*d*) δ 7.40 – 7.28 (m, 10H), 7.28 – 7.21 (m, 3H), 7.15 – 7.05 (m, 2H), 6.97 (d, J = 7.7 Hz, 2H), 6.91 (d, J = 8.1 Hz, 2H), 5.50 (s, 1H), 2.29 (q, J = 7.6 Hz, 4H), 1.06 (t, J = 7.7 Hz, 6H) ppm. ^13^C NMR: (101 MHz, Chloroform-*d*) δ 153.9, 147.4, 145.1, 143.0, 130.5, 129.2, 128.8, 127.5, 127.4, 120.9, 118.7, 117.7, 113.6, 113.2, 63.1, 26.2, 14.7 ppm. ^31^P NMR: (240 MHz, Chloroform-*d*) δ 136.86 ppm. HRMS (ESI-TOF) *m*/*z* calcd for [C_35_H_33_NO_2_P] [M+H]^+^ 530.2249, found 530.2258.

#### Pd-catalyzed [4+2] cycloaddition of benzofuran-derived azadiene (**30**) with vinyl benzoxazinanone (**31**)

To a 10 mL vial charged with Pd_2_(dba)_3_·CHCl_3_ (5.2 mg, 5 mol%), chiral phosphoramidite ligand (*S*)**-L16** (5.3 mg, 10 mol%) azadiene **30** (45 mg, 0.12 mmol) and vinyl benzoxazinanone **31** (33 mg, 0.1 mmol), anhydrous acetonitrile (2 mL) was added at room temperature. The vial was then evacuated and back-filled five times with argon, and the mixture was allowed to stir at 0^o^C for 48 h. The reaction mixture was purified directly by silica gel chromatography (eluent: hexane:acetone = 20:1 to 10:1) to provide the desired chiral product **32** as a white solid (59 mg, 90% yield, >20:1 dr, 97% ee).

#### 4-Methyl-*N*-((*2S*,*2'S*,*4'S*,*E*)-2'-phenyl-1'-tosyl-4'-vinyl-1',4'-dihydro-2'*H*,3*H*-spiro[benzofuran-2,3'-quinolin]-3-ylidene)benzenesulfonamide (**32**)

**32**^52^**:**^1^H NMR (400 MHz, Chloroform-*d*) δ 8.41 (d, J = 8.1 Hz, 1H), 8.14 – 8.06 (m, 2H), 7.95 (d, J = 7.8 Hz, 1H), 7.62 – 7.54 (m, 2H), 7.52 – 7.44 (m, 1H), 7.30 (q, J = 8.3 Hz, 2H), 7.20 – 7.10 (m, 4H), 7.09 – 7.01 (m, 3H), 6.99 – 6.89 (m, 2H), 6.57 (d, J = 8.1 Hz, 2H), 6.44 (d, J = 8.4 Hz, 1H), 5.63 (s, 1H), 5.21 (dt, J = 16.6, 9.7 Hz, 1H), 5.11 (dd, J = 10.0, 2.1 Hz, 1H), 4.54 (d, J = 16.7 Hz, 1H), 2.60 (s, 3H), 2.25 (s, 3H), 2.03 (d, J = 9.5 Hz, 1H) ppm. ^13^C NMR (101 MHz, Chloroform-*d*) δ 180.1, 170.8, 144.0, 143.6, 139.2, 139.1, 136.3, 135.8, 134.3, 134.3, 130.4, 129.9, 129.3, 129.2, 128.2, 128.2, 127.8, 127.8, 127.4, 127.3, 127.2, 126.4, 122.5, 118.5, 112.0, 96.9, 70.0, 49.2, 21.8, 21.4 ppm.

#### General procedures for Pd-catalysed decarboxylation-cycloaddition of vinyl benzoxazinanone (**31**) with sulphur ylide (**33**)

In a flame-dried Schlenk tube underAr, Pd_2_(dba)_3_·CHCl_3_ (5.2 mg, 5 mol%) and phosphoramidites ligand (*S*)-**L18** (6.3 mg, 12 mol%) were mixed in dry chloroform, and the mixture was stirred at room temperature for 20 min, then cooled to -20^o^C, and vinyl benzoxazinanone **31** (0.1 mmol, 33 mg) and sulphur ylide **33** (0.15 mmol) were successively introduced. The resulting dark-green solution was stirred at this temperature until the reaction was completed (as indicated by thin-layer chromatography). After the solvent was evaporated under reduced pressure, the crude product was purified via silica gel flash chromatography (eluent: PE:EtOAc = 12:1) to give chiral 3-vinyl indoline **34**.

#### (4-Methoxyphenyl)((*2R*,*3R*)-1-tosyl-3-vinylindolin-2-yl)methanone (**34a**)

Product **34a**^53^ was obtained by silica column chromatography (PE:EtOAc = 12/1) as a white solid (43 mg, 99% yield, >20:1 dr, 88% ee). ^1^H NMR (400 MHz, Chloroform-*d*) δ 7.97 (d, J = 8.9 Hz, 2H), 7.73 (d, J = 8.3 Hz, 1H), 7.61 (d, J = 8.1 Hz, 1H), 7.27 – 7.22 (m, 3H), 6.99 (t, J = 7.3 Hz, 1H), 6.97 – 6.92 (m, 3H), 5.43 – 5.30 (m, 1H), 5.27 (d, J = 5.0 Hz, 1H), 5.03 – 4.88 (m, 2H), 3.86 (s, 3H), 3.77 (dd, J = 8.6, 5.0 Hz, 1H), 2.37 (s, 3H) ppm. ^13^C NMR (101 MHz, Chloroform-*d*) δ 193.0, 164.1, 144.4, 141.6, 137.0, 134.9, 131.5, 129.8, 128.9, 127.6, 127.1, 125.5, 124.2, 117.5, 115.2, 114.1, 70.9, 55.6, 50.7, 21.7 ppm.

#### (4-Chlorophenyl)((*2R*,*3R*)-1-tosyl-3-vinylindolin-2-yl)methanone (**34b**)

Product **34b**^53^ was obtained by silica column chromatography (PE:EtOAc = 12/1) as white solid (43 mg, 98% yield, >20:1 dr, 94% ee). ^1^H NMR (400 MHz, Chloroform-*d*) δ 7.93 (d, J = 8.7 Hz, 2H), 7.72 (d, J = 8.3 Hz, 2H), 7.61 (d, J = 8.1 Hz, 1H), 7.44 (d, J = 8.7 Hz, 1H), 7.30 – 7.22 (m, 3H), 7.05 – 6.93 (m, 2H), 5.44 – 5.32 (m, 1H), 5.20 (d, J = 5.3 Hz, 1H), 5.08 – 4.87 (m, 2H), 3.79 (dd, J = 8.5, 5.4 Hz, 1H), 2.38 (s, 3H) ppm. ^13^C NMR (101 MHz, Chloroform-*d*) δ 193.5, 144.7, 141.4, 140.3, 136.6, 134.5, 132.6, 131.1, 130.5, 129.9, 129.2, 129.1, 127.6, 125.6, 124.4, 117.9, 115.2, 71.3, 50.4, 21.7 ppm.

#### General procedures for synthesis of (*S*)-**39**, **40** and **41**

(*S*)-**36** was prepared according to a slightly modified procedure for the preparation of (*S*)-**21.** To a 100 mL three-neck round-bottom flask which contained (*S*)-**16** (20 mmol, 8.6 g), PdCl_2_(PPh_3_)_2_ (15 mol%, 2.1 g) and MeB(OH)_2_ (140 mmol, 8.4 g) was added DME (50 mL). After adding an aqueous (15 mL) solution of Na_2_CO_3_ (140 mmol, 14.8 g) via a syringe at room temperature under argon atmosphere, the mixture was refluxed for 24 h. The resulting solution was cooled to room temperature and extracted with EtOAc, and the combined organic layer was washed with water, brine, dried over Na_2_SO_4_, filtered and concentrated. The residue was purified by flash column chromatography on silica gel (eluent: PE:EtOAc = 25:1) to remove most of the catalyst and impurities, and the crude product (*S*)-**36** (84% yield) was directly used in the next step. (*S*)-**37** and (*S*)-**38** were prepared from 3,4,5-(F)_3_-C_6_H_2_B(OH)_2_ and 4-CF_3_-C_6_H_4_B(OH)_2_, respectively, according to the same procedure for the preparation of (*S*)-**21.** After quenching, the residue was purified by flash column chromatography on silica gel (eluent: PE:EtOAc = 25:1) to remove most of the catalyst and impurities, and the crude product (*S*)-**37** (87% yield) and (*S*)-**38** (85% yield) was directly used in the next step. (*S*)-**39∼41** were prepared according to a slightly modified procedure for the preparation of (*S*)-**L11.** To a flamed-dried 100 mL flask which contained (*S*)-**36** (5 mmol) was charged dry DCM. The solution was cooled to 0^o^C before adding a DCM (15 mL) solution of BBr_3_ (30 mmol, 2.9 mL) dropwise. After the addition completed, the reaction mixture was allowed to warm to room temperature and stirred for 7 h. Saturated aqueous NH_4_Cl solution was added to quench the reaction. The mixture was extracted with DCM, and the combined organic layer was washed with water, brine, dried over Na_2_SO_4_, filtered and concentrated. The crude product was purified by column chromatography on silica gel (eluent: PE:EtOAc = 25:1 to 5:1) to afford **(*S*)-39** (90% yield) as gum. (*S*)-**40** and (*S*)-**41** were prepared from (*S*)-**37** and (*S*)-**38**, respectively, according to the same procedure for the preparation of (*S*)-**39.** After quenching, the residue was subjected to flash column chromatography (eluent: PE:EtOAc = 25:1) to remove most of the catalyst and impurities, and the crude product (*S*)-**40** (88% yield) and (*S*)-**41** (89% yield) was directly used in the next step.

#### (*S*)-6,6'-Diethyl-5,5'-dimethyl-[1,1'-biphenyl]-2,2'-diol ((*S*)-**39**)

^1^H NMR: (400 MHz, Chloroform-*d*) δ 7.15 (d, J = 8.3 Hz, 2H), 6.83 (d, J = 8.3 Hz, 2H), 4.53 (s, 2H), 2.38 (dq, J = 13.4, 7.6 Hz, 2H), 2.32 (s, 6H), 2.23 (dq, J = 13.3, 7.6 Hz, 2H), 0.93 (t, J = 7.6 Hz, 6H) ppm. ^13^C NMR: (101 MHz, Chloroform-*d*) δ 152.1, 143.0, 132.2, 128.8, 119.5, 113.0, 24.3, 19.1, 13.5 ppm.

#### General procedures for synthesis of (*S*)-**42**∼**45**

To a solution of **(*S*)-39** (3.63 g, 15 mmol) in DCM (50 mL) was added successively morpholine (7.8 mL, 90 mmol) and I_2_ (9.5 g, 37.5 mmol) at room temperature. The mixture was stirred for 10 h. After that, 2 M HCl (50 mL) was added. The aqueous layer was extracted with CH_2_Cl_2_, and the combined organic layer was washed with saturated Na_2_S_2_O_3_ solution and brine, and then dried over Na_2_SO_4_ and concentrated under reduced pressure. The crude product was purified by recrystallize in PE:DCM (50:1) to afford **(*S*)-42** (90% yield) as white solid. (*S*)-**43,** (*S*)-**44** and (*S*)-**45** were prepared from (*S*)-**L11**, (*S*)-**40** and (*S*)-**41**, respectively, according to the same procedure for the preparation of (*S*)-**42**.

#### (*S*)-6,6'-Diethyl-3,3'-diiodo-5,5'-dimethyl-[1,1'-biphenyl]-2,2'-diol ((*S*)-**42**)

^1^H NMR: (400 MHz, Chloroform-*d*) δ 7.59 (s, 2H), 4.95 (s, 2H), 2.39 – 2.30 (m, 2H), 2.29 (s, 6H), 2.24 – 2.12 (m, 2H), 0.92 (t, J = 7.6 Hz) ppm. ^13^C NMR: (101 MHz, Chloroform-*d*) δ 150.8, 143.5, 140.3, 131.0, 121.5, 81.1, 24.2, 18.8, 13.3 ppm.

#### (*S*)-2',2''-Diethyl-5',5''-diiodo-[1,1':3',1'':3'',1'''-quaterphenyl]-4',6''-diol ((*S*)-**43**)

^1^H NMR: (400 MHz, Chloroform-*d*) δ 7.66 (s, 2H), 7.48 – 7.30 (m, 10H), 5.23 (s, 2H), 2.43 (dq, J = 15.0, 7.5 Hz, 2H), 2.24 (dq, J = 14.9, 7.5 Hz, 2H), 0.74 (t, J = 7.5 Hz, 6H) ppm. ^13^C NMR: (101 MHz, Chloroform-*d*) δ 151.9, 143.2, 140.6, 140.4, 137.7, 129.4, 128.3, 127.2, 121.7, 81.4, 24.4, 14.3 ppm.

#### (*S*)-2',2''-Diethyl-3,3''',4,4''',5,5'''-hexafluoro-5',5''-diiodo-[1,1':3',1'':3'',1'''-quaterphenyl]-4',6''-diol ((*S*)-**44**)

^1^H NMR: (400 MHz, Chloroform-*d*) δ 7.59 (s, 2H), 7.07 – 6.83 (m, 4H), 5.27 (s, 2H), 2.37 (dq, J = 15.0, 7.5 Hz, 2H), 2.19 (dq, J = 14.8, 7.5 Hz, 2H), 0.75 (t, J = 7.5 Hz, 6H) ppm. ^13^C NMR: (101 MHz, Chloroform-*d*) δ 152.6, 152.2 (dd, J = 9.6, 4.0 Hz), 149.7 (dd, J = 9.9, 4.1 Hz), 143.0, 140.0, 136.27 (q, J = 7.5 Hz), 134.4, 122.0, 116.98 – 108.43 (m), 82.0, 24.2, 14.3 ppm. ^19^F NMR: (282 MHz, Chloroform-*d*) δ-134.00 (dd, J = 21.1, 8.1 Hz, 4F), -161.77 – -162.01 (m, 2F) ppm.

#### (*S*)-2',2''-Diethyl-5',5''-diiodo-4,4'''-bis(trifluoromethyl)-[1,1':3',1'':3'',1'''-quaterphenyl]-4',6''-diol ((*S*)-**45**)

^1^H NMR: (400 MHz, Chloroform-*d*) δ 7.74 – 7.56 (m, 6H), 7.50 – 7.35 (m, 4H), 5.27 (s, 2H), 2.42 – 2.33 (m, 2H), 2.22 (dq, J = 14.8, 7.4 Hz, 2H), 0.80 – 0.65 (m, 6H) ppm. ^13^C NMR: (101 MHz, Chloroform-*d*) δ 152.4, 144.2, 143.7, 143.0, 140.1, 137.1, 136.1, 136.1, 129.8, 129.7, 129.4, 125.3 (d, J = 3.3 Hz), 124.5, 124.4, 124.4, 124.3 (d, J = 271.9 Hz), 121.9, 81.9, 24.3, 14.3 ppm. ^19^F NMR: (282 MHz, Chloroform-*d*) δ-62.29 ppm.

#### General procedures for synthesis of (*S*)-**46**∼**49**

At 0^o^C underAr, to a suspension of NaH (60% dispersion in mineral oil, 3.5 equivs.) in anhydrous THF (20 mL) was added a solution of (*S*)-**42** (2.6 mmol, 1 equiv.) in anhydrous THF (10 mL) slowly. The reaction mixture was allowed to warm to room temperature and stirred for 2 h after MOMCl (3.5 equivs.) was added in portions at 0^o^C. The mixture was stirred for additional 10hat room temperature. Subsequently, the reaction mixture was cooled to 0^o^C and quenched by dropwise addition of a saturated aqueous NH_4_Cl solution (20 mL) and H_2_O (10 mL). The resulting mixture was extracted with EtOAc. The combined organic layers were washed with brine, dreied over Na_2_SO_4,_ and concentrated. The crude (*S*)**-46** was directly used in the next step. (*S*)-**49** was prepared from (*S*)-**45** according to the same procedure for the preparation of (*S*)-**46**. The crude (*S*)**-49** was directly used in the next step. (*S*)**-47** and (*S*)**-48** were prepared from (*S*)**-43** and (*S*)**-44**, respectively, according to the similar procedure for the preparation of (*S*)-**46**, but after quenched reaction, the crude product was purified by column chromatography on silica gel (eluent: PE:EtOAc = 50:1) to afford (*S*)**-47** and (*S*)**-48.**

#### (*S*)-2',2''-Diethyl-5',5''-diiodo-4',6''-bis(methoxymethoxy)-1,1':3',1'':3'',1'''-quaterphenyl ((*S*)-**47**)

^1^H NMR: (400 MHz, Chloroform-*d*) δ 7.69 (s, 2H), 7.44 – 7.34 (m, 6H), 7.33 – 7.28 (m, 4H), 5.10 – 4.90 (m, 4H), 3.04 (s, 6H), 2.43 (dh, J = 21.3, 7.4 Hz, 4H), 0.69 (t, J = 7.5 Hz, 6H) ppm. ^13^C NMR: (101 MHz, Chloroform-*d*) δ 154.9, 143.3, 140.9, 140.6, 140.6, 132.7, 129.1, 128.3, 127.2, 99.5, 88.7, 56.5, 24.8, 13.5 ppm.

#### (*S*)-2',2''-Diethyl-3,3''',4,4''',5,5'''-hexafluoro-5',5''-diiodo-4',6''-bis(methoxymethoxy)-1,1':3',1'':3'',1'''-quaterphenyl ((*S*)-**48**)

^1^H NMR: (400 MHz, Chloroform-*d*) δ 7.65 (s, 2H), 6.94 (t, J = 6.1 Hz, 4H), 4.99 (d, J = 13.4 Hz, 4H), 3.00 (s, 6H), 2.36 (dq, J = 13.5, 7.2, 6.4 Hz, 4H), 0.71 (t, J = 6.8 Hz, 6H) ppm. ^13^C NMR: (101 MHz, Chloroform-*d*) δ 156.0, 152.3 (d, J = 10.1 Hz), 149.7 (dd, J = 9.5, 3.9 Hz), 143.0, 140.4, 137.5, 136.5 (q, J = 6.7, 5.4 Hz), 132.7, 114.6 – 112.0 (m), 100.0, 88.9, 56.5, 24.6, 13.6 ppm. ^19^F NMR: (282 MHz, Chloroform-*d*) δ-133.84 (dd, J = 20.4, 7.6 Hz, 4F),-161.73 (t, J = 20.5 Hz, 2F) ppm.

#### General procedures for synthesis of (*S*)-**50**∼**53**

To a 100 mL three-neck round-bottom flask which contained (*S*)-**46** (1.5 mmol), Pd_2_(dba)_3_ (3 mol%), SPhos (6 mol%), K_3_PO_4_ (9 mmol) and 3,5-(CF_3_)_2_-C_6_H_3_B(OH)_2_ (6 mmol) was added toluene (10 mL). Under argon atmosphere, the mixture was refluxed for 24 h. The reaction mixture was cooled to room temperature, H_2_O was added to the mixture, and then extracted with EtOAc. The combined organic layer was washed with water, brine, dried over Na_2_SO_4_, filtered and concentrated. The residue was purified by a column chromatography (eluent: PE:EtOAc = 50:1) to afford the desired product (*S*)-**50** in 80% yield. (*S*)-**51** and (*S*)-**52** was prepared from (*S*)-**47** and (*S*)-**48**, respectively, according to the same procedure for the preparation of (*S*)-**50** (eluent for chromatography: PE:EtOAc = 50:1) in 75%∼76% yield. (*S*)-**53** was prepared from (*S*)-**49** according to the same procedure for the preparation of (*S*)-**50**. The crude (*S*)-**53** (81% yield) was directly used in the next step.

#### (*S*)-4',6''-Diethyl-2',2''-bis(methoxymethoxy)-5',5''-dimethyl-3,3''',5,5'''-tetrakis(trifluoromethyl)-1,1':3',1'':3'',1'''-quaterphenyl ((*S*)-**50**)

^1^H NMR: (400 MHz, Chloroform-*d*) δ 8.02 (s, 4H), 7.83 (s, 2H), 7.19 (s, 2H), 4.53 – 4.29 (m, 4H), 2.73 (s, 6H), 2.50 (q, J = 7.5 Hz, 4H), 2.44 (s, 6H), 1.10 (t, J = 7.5 Hz, 6H) ppm. ^13^C NMR: (101 MHz, Chloroform-*d*) δ 150.88, 144.03, 141.55, 133.03, 132.17, 131.99, 131.60 (q, J = 33.1 Hz), 129.55, 123.5 (q, J = 272.6 Hz), 120.6 (d, J = 6.7 Hz), 98.76, 56.10, 25.17, 19.47, 12.97 ppm. ^19^F NMR: (282 MHz, Chloroform-*d*) δ-62.72 ppm.

#### (*S*)-4',6''-Diethyl-2',2''-bis(methoxymethoxy)-5',5''-diphenyl-3,3''',5,5'''-tetrakis(trifluoromethyl)-1,1':3',1'':3'',1'''-quaterphenyl ((*S*)-**51**)

^1^H NMR: (400 MHz, Chloroform-*d*) δ 8.10 (s, 4H), 7.87 (s, 2H), 7.46 (dq, J = 11.7, 6.5, 5.1 Hz, 10H), 7.30 (s, 2H), 4.65 – 4.50 (m, 4H), 2.90 (s, 6H), 2.68 (dhept, J = 14.1, 7.3 Hz, 4H), 0.86 (t, J = 7.4 Hz, 6H) ppm. ^13^C NMR: (101 MHz, Chloroform-*d*) δ 152.19, 143.79, 141.59, 141.15, 139.46, 132.39, 132.27, 131.77 (d, J = 33.2 Hz), 129.59, 129.27, 128.45, 127.31, 123.47 (q, J = 272.6 Hz), 120.80 (d, J = 6.6 Hz), 99.03, 56.35, 25.09, 13.77, 1.13 ppm. ^19^F NMR: (282 MHz, Chloroform-*d*) δ-62.69 ppm.

#### (*S*)-5',5''-Bis(3,5-bis(trifluoromethyl)phenyl)-2',2''-diethyl-3,3''',4,4''',5,5'''-hexafluoro-4',6''-bis(methoxymethoxy)-1,1':3',1'':3'',1'''-quaterphenyl ((*S*)-**52**)

^1^H NMR: (400 MHz, Chloroform-*d*) δ 8.02 (s, 4H), 7.87 (s, 2H), 7.22 (s, 2H), 7.14 – 6.92 (m, 4H), 4.59 – 4.35 (m, 4H), 2.84 (s, 6H), 2.68 – 2.50 (m, 4H), 0.86 (t, J = 7.5 Hz, 6H) ppm. ^13^C NMR: (101 MHz, Chloroform-*d*) δ 153.0, 152.4 (d, J = 8.2 Hz), 149.8 (d, J = 7.3 Hz), 143.5, 140.5, 136.4, 132.4, 132.2, 131.8, 130.1, 129.4, 123.3 (d, J = 272.8 Hz), 121.2 (d, J = 9.8 Hz), 113.95 – 113.32 (m), 99.2, 56.4, 24.9, 13.8 ppm. ^19^F NMR: (282 MHz, Chloroform-*d*) δ-62.74, -133.80 (dd, J = 20.7, 7.7 Hz, 4F),-161.83 (tt, J = 21.3, 6.3 Hz, 2F) ppm.

#### General procedures for synthesis of (*S*)-**54**∼**57**

To a 50 mL round-bottom flask which contained **(*S*)-50** (0.5 mmol) in 1,4-dioxane (10 mL) was added conc. HCl (0.5 mL), the mixture was stirred at 70^o^C for 10 h. On cooling, the mixture was extracted with DCM. The combined organic layer was dried over anhydrous Na_2_SO_4_, filtered and concentrated to dryness. The residue was purified by a column chromatography (eluent: PE:EtOAc = 50:1) to afford the desired product (*S*)-**54** in 71% yield. (*S*)-**55** was prepared from (*S*)-**51** according to the same procedure for the preparation of (*S*)-**54**. The crude (*S*)**-55** (65% yield) was directly used in the next step. (*S*)**-56** was prepared from (*S*)**-52** according to the same procedure for the preparation of (*S*)**-54**. (*S*)**-57** was prepared from (*S*)**-53** according to the same procedure for the preparation of (*S*)-**54**. The crude (*S*)**-57** (72% yield) was directly used in the next step.

#### (*S*)-4',6''-Diethyl-5',5''-dimethyl-3,3''',5,5'''-tetrakis(trifluoromethyl)-[1,1':3',1'':3'',1'''-quaterphenyl]-2',2''-diol ((*S*)-**54**)

^1^H NMR: (400 MHz, Chloroform-*d*) δ 8.09 (s, 4H), 7.83 (s, 2H), 7.32 (s, 2H), 4.88 (s, 2H), 2.54 – 2.44 (m, 2H), 2.43 (s, 6H), 2.35 (dq, J = 14.0, 7.2 Hz, 2H), 1.03 (t, J = 7.5 Hz, 6H) ppm. ^13^C NMR: (101 MHz, Chloroform-*d*) δ 148.9, 144.3, 139.9, 133.4, 131.5 (q, J = 32.9 Hz), 130.2, 129.4, 123.6 (d, J = 272.7 Hz), 123.3, 120.7 (d, J = 3.3 Hz), 120.1, 24.6, 19.1, 13.6 ppm. ^19^F NMR: (282 MHz, Chloroform-*d*) δ-62.690 ppm. HRMS (ESI-TOF) *m*/*z* calcd for [C_34_H_27_F_12_O_2_] [M+H]^+^ 695.1819, found 695.1828.

#### (*S*)-4',6''-Diethyl-3,3''',5,5'''-tetrakis(trifluoromethyl)-5',5''-bis(3,4,5-trifluorophenyl)-[1,1':3',1'':3'',1'''-quaterphenyl]-2',2''-diol ((*S*)-**56**)

^1^H NMR: (400 MHz, Chloroform-*d*) δ 8.10 (s, 4H), 7.88 (s, 2H), 7.37 (s, 2H), 7.11 – 6.93 (m, 4H), 5.20 (s, 2H), 2.54 (dq, J = 15.0, 7.5 Hz, 2H), 2.36 (dq, J = 14.9, 7.4 Hz, 2H), 0.88 (t, J = 7.5 Hz, 6H) ppm. ^13^C NMR: (101 MHz, Chloroform-*d*) δ 152.35 (dd, J = 10.0, 3.8 Hz), 150.9, 149.84 (dd, J = 9.7, 3.9 Hz), 143.8, 139.43 (dt, J = 252.9, 15.0 Hz), 138.7, 136.52 (q, J = 7.3, 6.7 Hz), 134.1, 133.5, 131.84 (q, ^2^J_CF_ = 33.4 Hz), 129.5, 123.40 (q, ^1^J_CF_ = 272.9 Hz), 124.1, 121.63 – 121.02 (m), 120.2, 115.01 – 112.00 (m), 24.6, 14.7 ppm. ^19^F NMR: (282 MHz, Chloroform-*d*) δ-62.7, -133.59 (dd, J = 20.5, 7.7 Hz, 4F),-161.55 (tt, J = 21.4, 6.4 Hz, 2F) ppm. HRMS (ESI-TOF) *m*/*z* calcd for [C_44_H_25_F_18_O_2_] [M+H]^+^ 927.1567, found 927.1571.

#### General procedures for synthesis of (*S*)-**CPA-2**∼ **CPA-5**

To a solution of biphenol (*S*)-54 (1.8 mmol) in pyridine (10 mL) was slowly added phosphoryl chloride (3 equiv.) at room temperature and the mixture was heated to reflux for 3 h. After the reaction mixture was allowed to cool to room temperature, distilled water (0.9 mL) was added, and then the mixture was heated to 95^o^C for 30 min and cooled again to room temperature. Pyridine was removed in vacuo, and 6 M HCl was added to the mixture, and stirred at room temperature for 0.5 h. The mixture was extracted with DCM, and the combined organic extract was washed with 6 M HCl solution 3 times, and dried over anhydrous Na_2_SO_4_, and concentrated in vacuo. The crude residue was purified by column chromatography (eluent: DCM:MeOH = 4:1) to give the desired compound (*S*)-**CPA-2**. (*S*)-CPA-**3∼CPA-5** was prepared from (*S*)-**55∼57** according to the same procedure for the preparation of (*S*)-**CPA-2.**

#### (11*aS*)-4,8-Bis(3,5-bis(trifluoromethyl)phenyl)-1,11-diethyl-6-hydroxy-2,10-dimethyldibenzo[d,f][1,3,2]dioxaphosphepine 6-oxide ((*S*)-**CPA-2**)

^1^H NMR: (400 MHz, Chloroform-*d*) δ 8.01 (s, 4H), 7.61 (s, 2H), 7.19 (s, 2H), 2.80 – 2.49 (m, 4H), 2.46 (s, 6H), 0.85 (s, 6H) ppm. ^13^C NMR: (101 MHz, Chloroform-*d*) δ 144.8, 144.7, 143.6, 139.6, 133.4, 132.2, 130.9 (q, J = 33.1 Hz), 129.8, 128.7, 127.7, 123.5 (q, J = 272.7 Hz), 120.6, 23.9, 19.4, 13.9 ppm. ^19^F NMR: (282 MHz, Chloroform-*d*) δ-63.046 ppm. ^31^P NMR: (240 MHz, Chloroform-*d*) δ 0.969 ppm. HRMS (ESI-TOF) *m*/*z* calcd for [C_34_H_26_F_12_O_4_P] [M+H]^+^ 757.1377, found 757.1389.

#### (11*aS*)-4,8-Bis(3,5-bis(trifluoromethyl)phenyl)-1,11-diethyl-6-hydroxy-2,10-diphenyldibenzo[*d*,*f*][1,3,2]dioxaphosphepine 6-oxide ((*S*)-**CPA-3**)

^1^H NMR: (400 MHz, Chloroform-*d*) δ 8.13 (s, 4H), 7.84 (s, 2H), 7.50 – 7.43 (m, 4H), 7.43 – 7.36 (m, 8H), 5.21 (s, 2H), 2.57 (dq, J = 14.9, 7.5 Hz, 2H), 2.38 (dq, J = 14.8, 7.5 Hz, 2H), 0.84 (t, J = 7.5 Hz, 6H) ppm. ^13^C NMR: (101 MHz, Chloroform-*d*) δ 150.2, 144.0 140.9, 139.4, 137.1, 133.6, 131.6 (q, J = 33.2 Hz), 129.5, 129.5, 129.4, 128.5, 127.4, 123.5 (q, J = 272.6 Hz), 123.5, 121.0 (d, J = 6.5 Hz), 120.1, 24.7, 14.6 ppm. ^19^F NMR: (282 MHz, Chloroform-*d*) δ-62.679 ppm. ^31^P NMR: (240 MHz, Chloroform-*d*) δ 0.97 ppm. HRMS (ESI-TOF) *m*/*z* calcd for [C_44_H_28_F_12_O_4_P] [M-H]^+^ 879.1528, found 879.1473.

#### (11*aS*)-4,8-Bis(3,5-bis(trifluoromethyl)phenyl)-1,11-diethyl-6-hydroxy-2,10-bis(3,4,5-trifluorophenyl)dibenzo[d,f][1,3,2]dioxaphosphepine 6-oxide ((*S*)-**CPA-4**)

^1^H NMR: (400 MHz, Chloroform-*d*) δ 8.12 (s, 4H), 7.82 (s, 2H), 7.30 (s, 2H), 7.04 (t, J = 7.0 Hz, 4H), 2.72 (dq, J = 14.6, 7.3 Hz, 2H), 2.62 (dq, J = 14.6, 7.3 Hz, 2H), 0.73 (t, J = 7.4 Hz, 6H) ppm. ^13^C NMR: (101 MHz, Chloroform-*d*) δ 152.32 (d, J = 8.9 Hz), 149.83 (dd, J = 9.7, 3.7 Hz), 146.97 (d, J = 9.0 Hz), 143.0, 140.75 (t, J = 14.9 Hz), 138.9, 137.2, 136.69 (q, J = 7.4 Hz), 132.1, 131.52 (q, J = 33.5 Hz), 129.8, 129.21 (d, J = 2.7 Hz), 128.7, 123.42 (q, J = 272.5 Hz), 121.1, 115.10 – 112.44 (m), 20.3, 15.1 ppm. ^19^F NMR: (282 MHz, Chloroform-*d*) δ-62.84, -133.41 – -133.87 (m, 4F),-161.47 (t, J = 20.2 Hz, 2F) ppm. ^31^P NMR: (240 MHz, Chloroform-*d*) δ-0.320 ppm. HRMS (ESI-TOF) *m*/*z* calcd for [C_44_H_22_F_18_O_4_P] [M-H]^+^ 987.0963, found 987.0944.

#### (11*aS*)-4,8-Bis(3,5-bis(trifluoromethyl)phenyl)-1,11-diethyl-6-hydroxy-2,10-bis(4-(trifluoromethyl)phenyl)dibenzo[*d*,*f*][1,3,2]dioxaphosphepine 6-oxide ((*S*)-**CPA-5**)

^1^H NMR: (400 MHz, Chloroform-*d*) δ 8.11 (s, 4H), 7.86 (s, 2H), 7.78 – 7.69 (m, 4H), 7.57 – 7.48 (m, 4H), 7.38 (s, 2H), 5.23 (s, 2H), 2.55 (dq, J = 15.0, 7.5 Hz, 2H), 2.38 (dq, J = 14.9, 7.5 Hz, 2H), 0.85 (t, J = 7.5 Hz, 6H) ppm. ^13^C NMR: (101 MHz, Chloroform-*d*) δ 150.6, 144.4, 143.8, 139.0, 135.6, 133.5, 131.8 (q, J = 33.0 Hz), 130.0, 129.8, 129.7, 129.5, 125.6, 125.5, 123.9, 123.4 (d, J = 272.9 Hz), 121.2 (d, J = 3.1 Hz), 120.2, 24.6, 14.7 ppm. ^19^F NMR: (282 MHz, Chloroform-*d*) δ-62.397, -62.702 ppm. ^31^P NMR: (240 MHz, Chloroform-*d*) δ 1.046 ppm. HRMS (ESI-TOF) *m*/*z* calcd for [C_46_H_26_F_18_O_4_P] [M-H]^+^ 1015.1276, found 1015.1269.

#### General procedure for synthesis of (*S*)-**CPA-1**

(*S*)**-CPA-1** was prepared from (*S*)-**43** according to the same procedures for the preparation of (*S*)-**CPA-2**∼**CPA-5** (eluent for chromatography: DCM/MeOH = 4:1) in 81% yield as a white solid

#### (11*aS*)-1,11-Diethyl-6-hydroxy-4,8-diiodo-2,10-diphenyldibenzo[d,f][1,3,2]dioxaphosphepine 6-oxide ((*S*)-**CPA-1**)

^1^H NMR: (400 MHz, Chloroform-*d*) δ 8.82 (s, 1H), 7.76 (s, 2H), 7.46 – 7.35 (m, 6H), 7.34 – 7.29 (m, 4H), 2.70 (dq, J = 14.7, 7.4 Hz, 2H), 2.52 (dq, J = 14.7, 7.4 Hz, 2H), 0.55 (t, J = 7.4 Hz, 6H) ppm. ^13^C NMR: (101 MHz, Chloroform-*d*) δ 148.1, 148.0, 143.3, 142.0, 141.3, 140.1, 129.3, 128.4, 127.6, 127.3, 85.9, 24.4, 14.9 ppm. ^31^P NMR: (240 MHz, Chloroform-*d*) δ 1.624 ppm. HRMS (ESI-TOF) *m*/*z* calcd for [C_28_H_24_I_2_O_4_P] [M+H]^+^ 708.9502, found 708.9497.

#### Chiral phosphoric acid-catalyzed asymmetric synthesis of 1,1'-spirobiindane-7,7'-diol (SPINOL) derivative (**60**)

Under argon atmosphere, **59** (0.1 mmol), (*S*)-**CPA-4** (10 mol%) and 3 mL of anhydrous chloroform were added to a 10 mL of oven-dried pressure Schlenk tube with a magnetic stirring bar. Then the reaction proceeded in the sealed tube at 120^o^C (the temperature of oil bath) for 120 h. After completion of the reaction, the solvent was evaporated, and the residue was purified by flash chromatography eluted with PE:EtOAc = 10:1 to afford the product **60** (11 mg, 26% yield, 98% ee) as a white solid.

#### (*S*)-4,4'-Dibromo-2,2',3,3'-tetrahydro-1,1'-spirobi[indene]-7,7'-diol (**60**)

**60**^57^: ^1^H NMR (400 MHz, Chloroform-*d*) δ 7.29 (d, J = 8.6 Hz, 2H), 6.57 (d, J = 8.5 Hz, 2H), 4.59 (s, 2H), 3.15 – 2.91 (m, 4H), 2.49 – 2.04 (m, 4H) ppm. ^13^C NMR (101 MHz, Chloroform-*d*) δ 152.0, 145.5, 132.6, 132.3, 116.7, 111.1, 60.4, 36.8, 32.8 ppm.

#### Chiral phosphoric acid-catalyzed asymmetric [4+3] cyclization of *in situ* generated *o*-quinonemethides from 2-indolylmethanol (**61**) with *o*-hydroxybenzylalcohol (**62**)

To the mixture of 2-indolylmethanols **61** (0.12 mmol), 2-(hydroxy(phenyl)methyl)phenol **62** (0.1 mmol), chiral phosphoric acid (*S*)-**CPA-4** (0.01 mmol) and MgSO_4_ (100 mg) was added DCE (2 mL), and the mixture was stirred at r.t. for 24 h. After the completion of the reaction indicated by TLC, the reaction mixture was filtered to remove MgSO_4_ and the solid powder was washed with ethyl acetate. The resultant solution was concentrated under the reduced pressure to give the residue, which was purified through preparative thin layer chromatography to afford pure products **63** (43 mg, 92% yield, 93% ee).

#### (*R*)-6,6,12-Triphenyl-7,12-dihydro-6*H*-benzo[6,7]oxepino[3,4-b]indole (**63**)

**63**^58^: ^1^H NMR (400 MHz, Chloroform-*d*) δ 7.75 – 7.66 (m, 1H), 7.51 (s, 1H), 7.45 – 7.39 (m, 1H), 7.38 – 7.34 (m, 2H), 7.33 – 7.28 (m, 3H), 7.27 – 7.13 (m, 12H), 7.06 – 6.94 (m, 3H), 6.83 (td, J = 7.8, 1.5 Hz, 1H), 6.28 (d, J = 7.8 Hz, 1H), 5.56 (s, 3H) ppm. ^13^C NMR (101 MHz, Chloroform-*d*) δ 153.5, 145.0, 145.0, 140.9, 139.8, 135.7, 135.5, 130.5, 129.4, 128.7, 128.4, 128.2, 127.8, 127.7, 127.6, 127.4, 126.1, 126.0, 124.4, 122.5, 120.1, 118.8, 113.9, 110.9, 87.4, 45.3 ppm.

## Data Availability

• All data reported in this paper will be shared by the lead contact upon request. The crystallographic, catalysts and catalysis are provided in Supplemental Information as referenced in the main text. All original crystal structures have been deposited at CCDC and are publicly available as of the date of publication. CCDC numbers are listed in the key resources table. • This paper does not report original code. • Any additional information required to reanalyze the data reported in this paper is available from the lead contact upon request.

## Footnotes

Supplemental information can be found online at https://doi.org/10.1016/j.isci.2023.106344.
