# Supplementary material for: Development of diverse adjustable axially chiral biphenyl ligands and catalysts
Source: iScience. 2023 Mar 7;26(4):106344. doi: 10.1016/j.isci.2023.106344 (PMC10040738; doi:10.1016/j.isci.2023.106344)
Supplement: Document S1, Figures S1–S262 [file mmc1.pdf]

iScience, Volume *26*

Supplemental information

Development of diverse adjustable axially chiral biphenyl ligands and catalysts

Jiyang Jie, Haijun Yang, Yufen Zhao, and Hua Fu

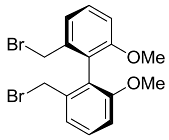

(*S*)-**3**

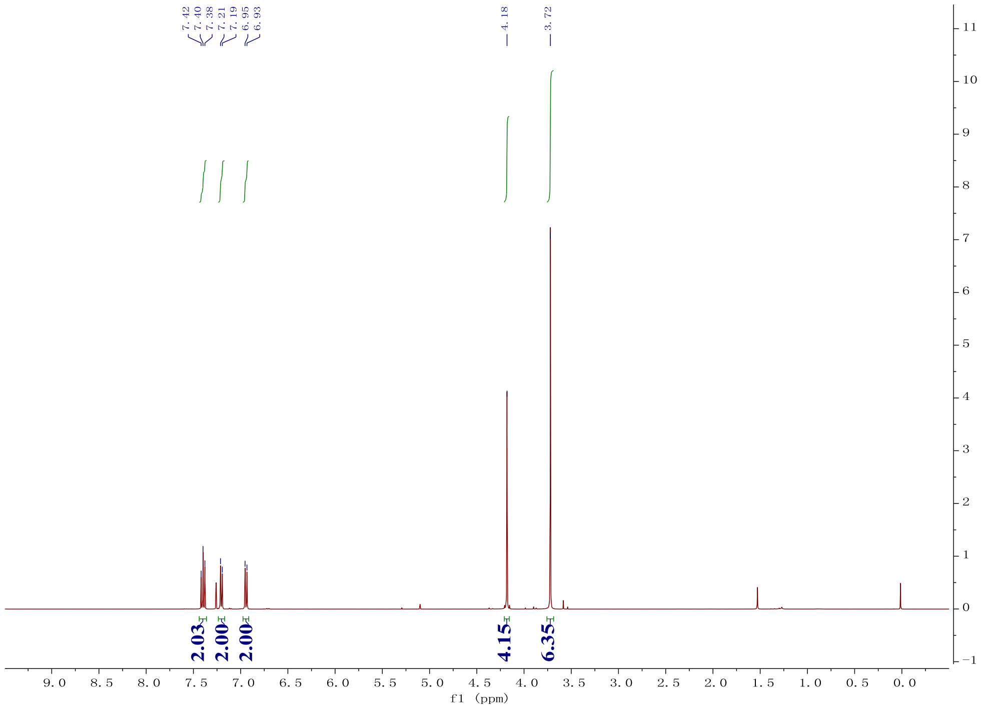

Figure S1. ^{1}H NMR spectrum of (*S*)-3, Related to Figure 2a

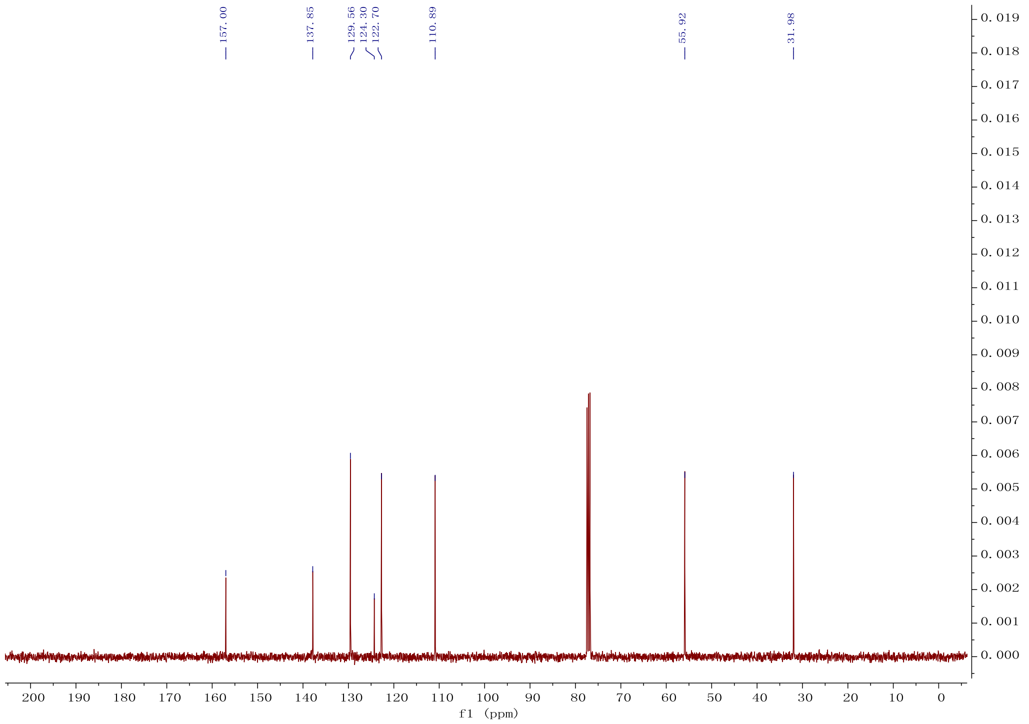

Figure S2. ^{13}C NMR spectrum of (*S*)-3, Related to Figure 2a

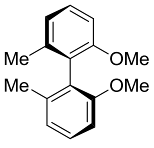

(*S*)-**4**

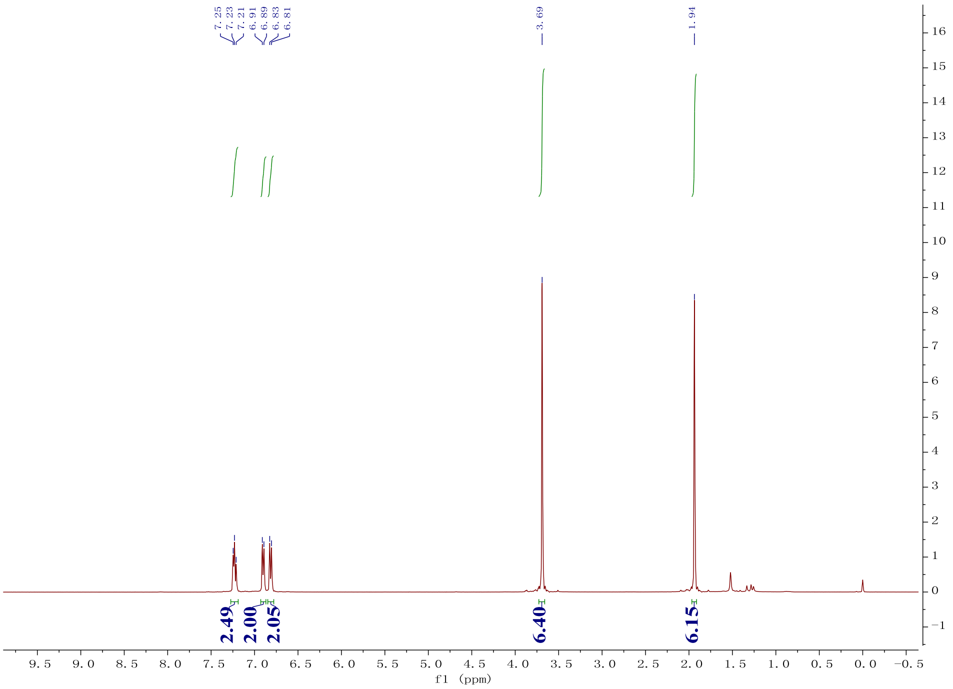

Figure S3. ^{1}H NMR spectrum of (*S*)-4, Related to Figure 2a

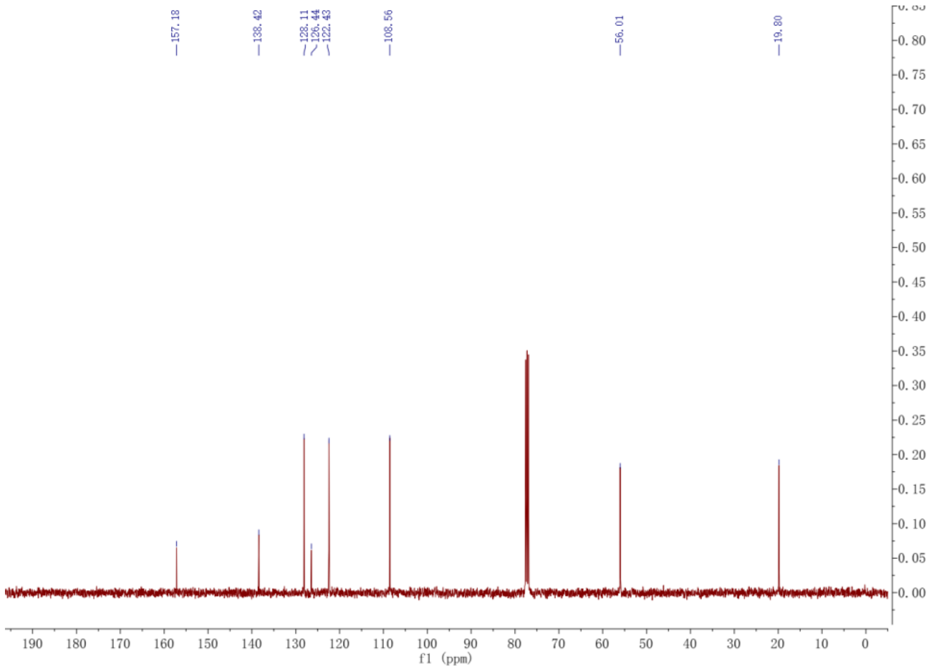

Figure S4. ^{13}C NMR spectrum of (*S*)-4, Related to Figure 2a

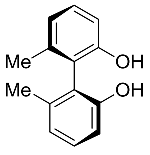

(*S*)-**L1**

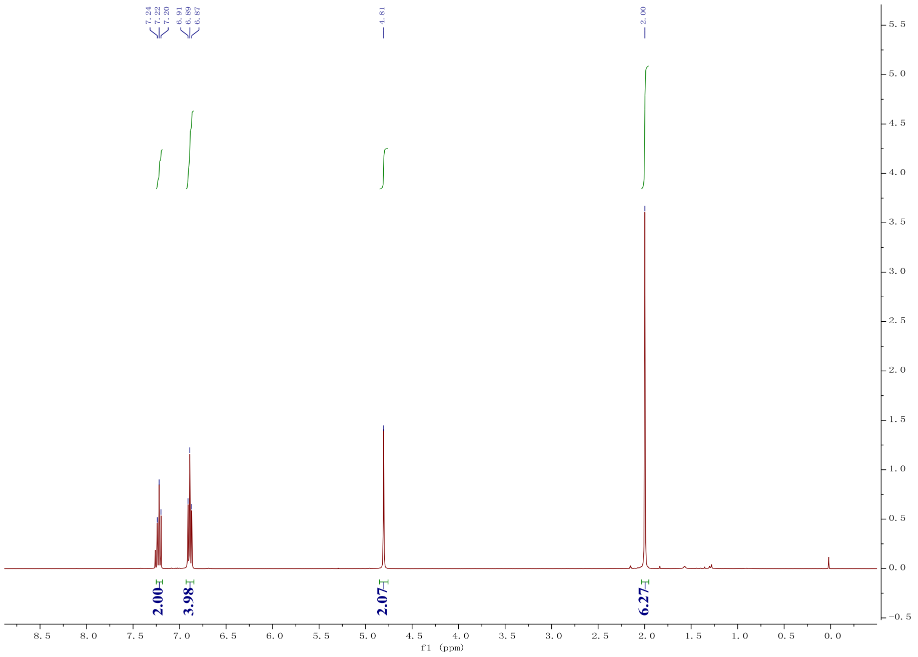

Figure S5. ¹H NMR spectrum of (*S*)-L1, Related to Figure 2a

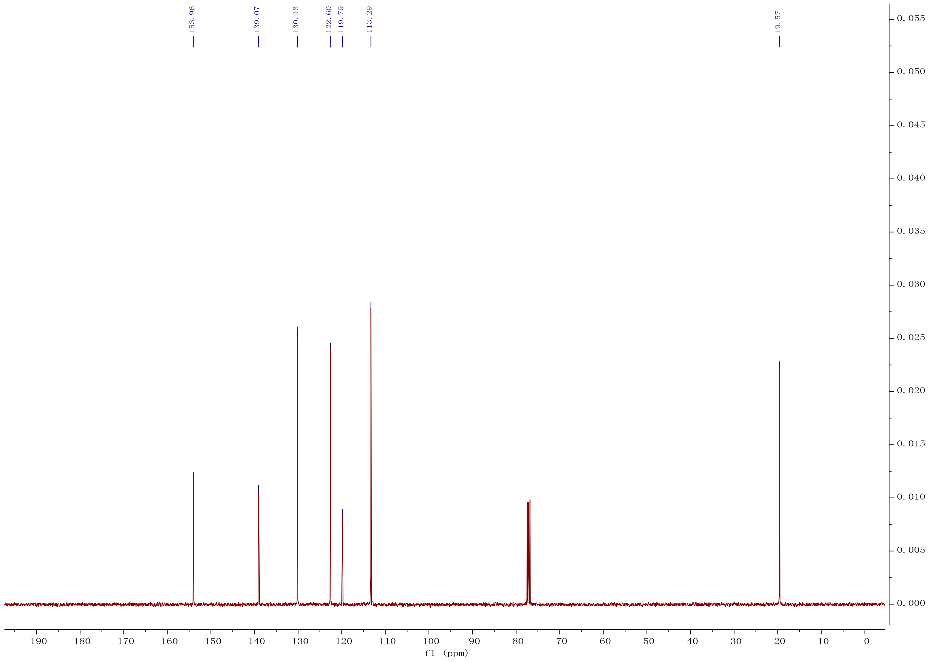

Figure S6. ¹³C NMR spectrum of (*S*)-L1, Related to Figure 2a

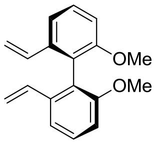

(*S*)-**5**

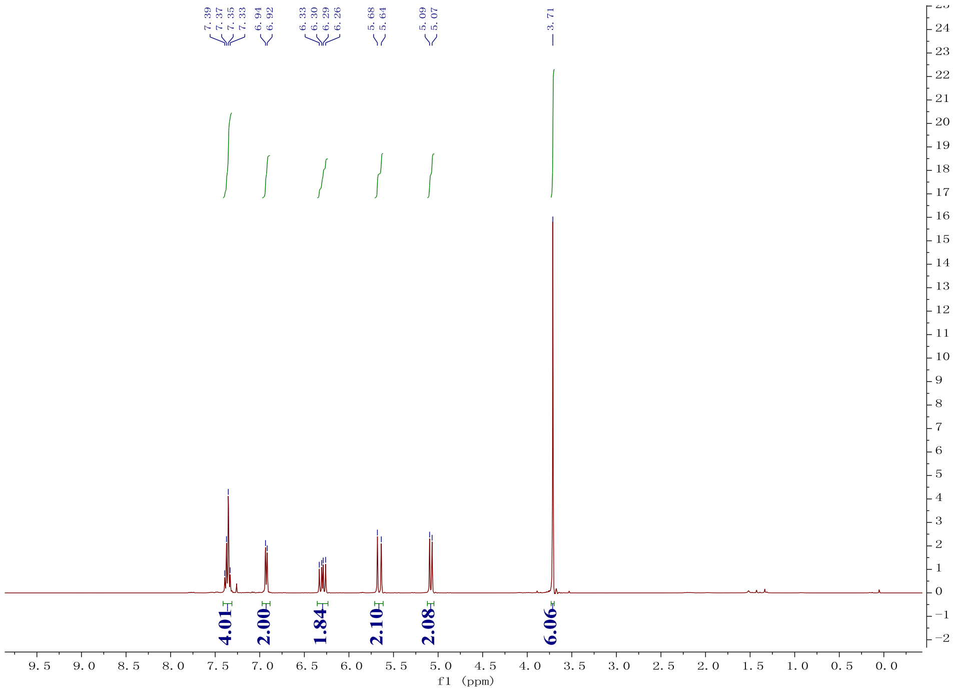

Figure S7. ^{1}H NMR spectrum of (*S*)-5, Related to Figure 2b

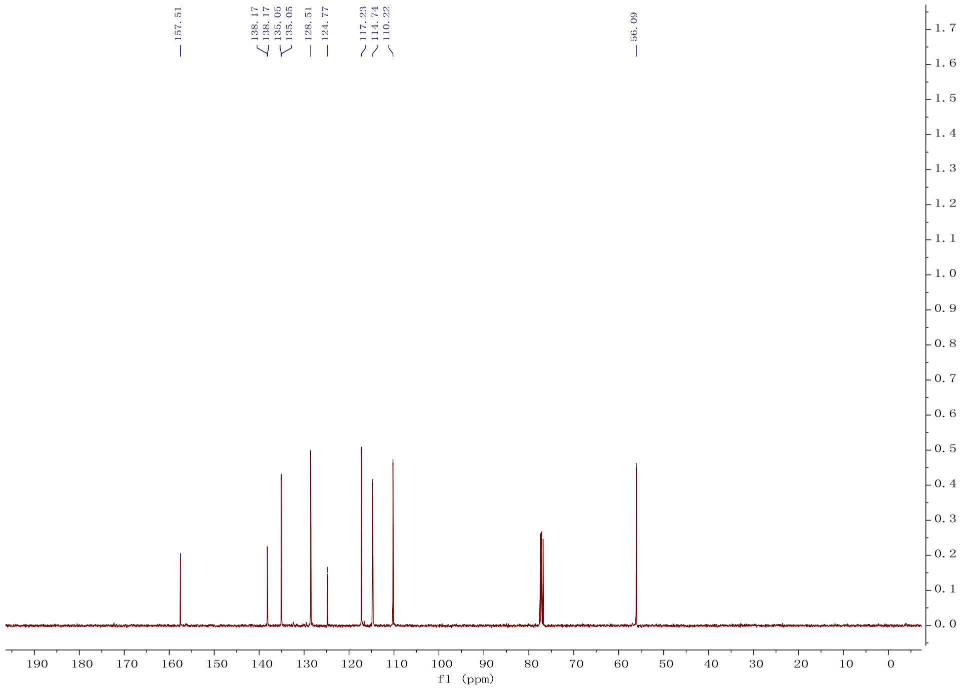

Figure S8. ^{13}C NMR spectrum of (*S*)-5, Related to Figure 2b

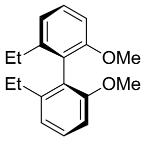

(*S*)-**6**

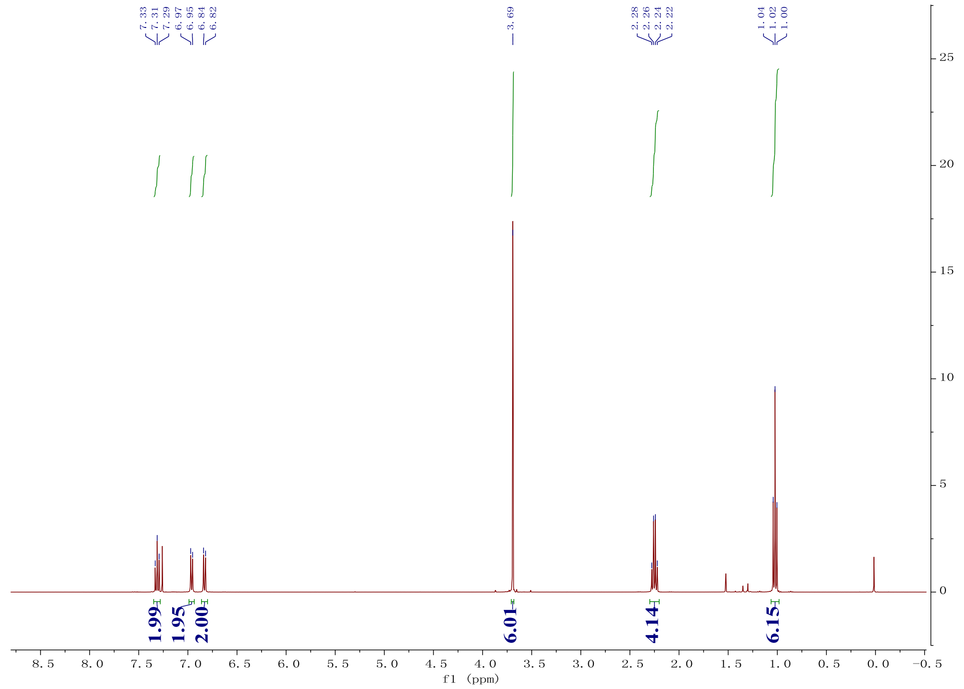

Figure S9. ^{1}H NMR spectrum of (*S*)-6, Related to Figure 2b

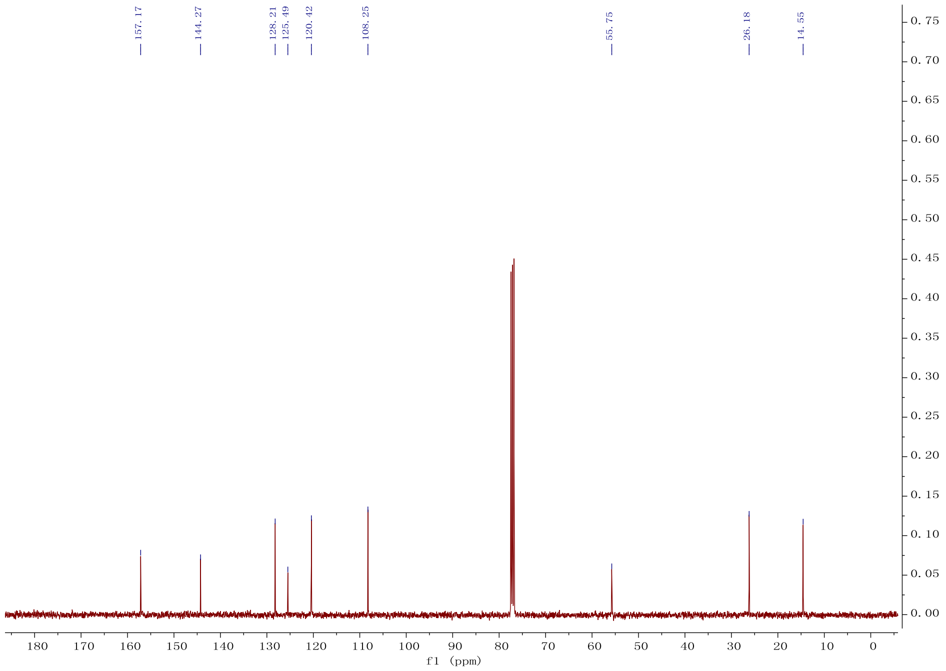

Figure S10. ^{13}C NMR spectrum of (*S*)-6, Related to Figure 2b

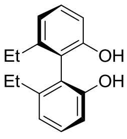

(*S*)-**L2**

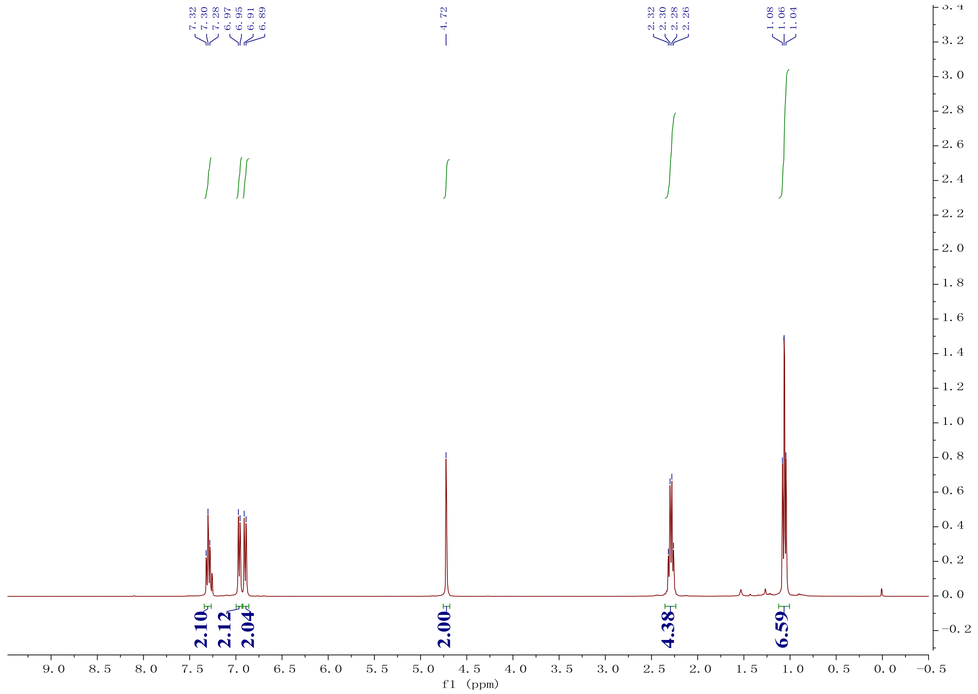

Figure S11. ¹H NMR spectrum of (*S*)-L2, Related to Figure 2b

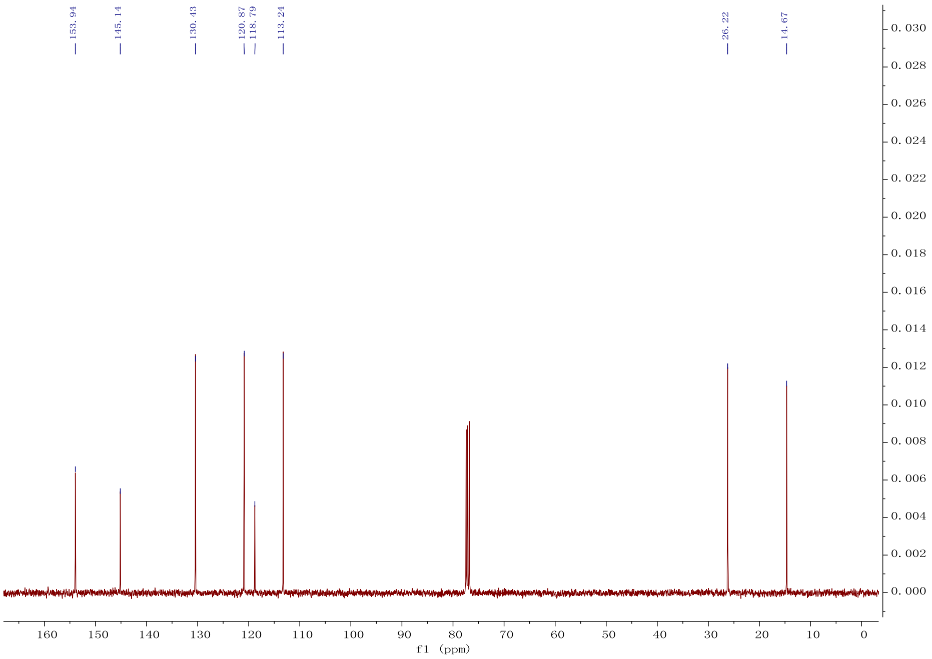

Figure S12. ¹³C NMR spectrum of (*S*)-L2, Related to Figure 2b

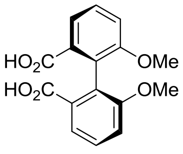

(*S*)-**7**

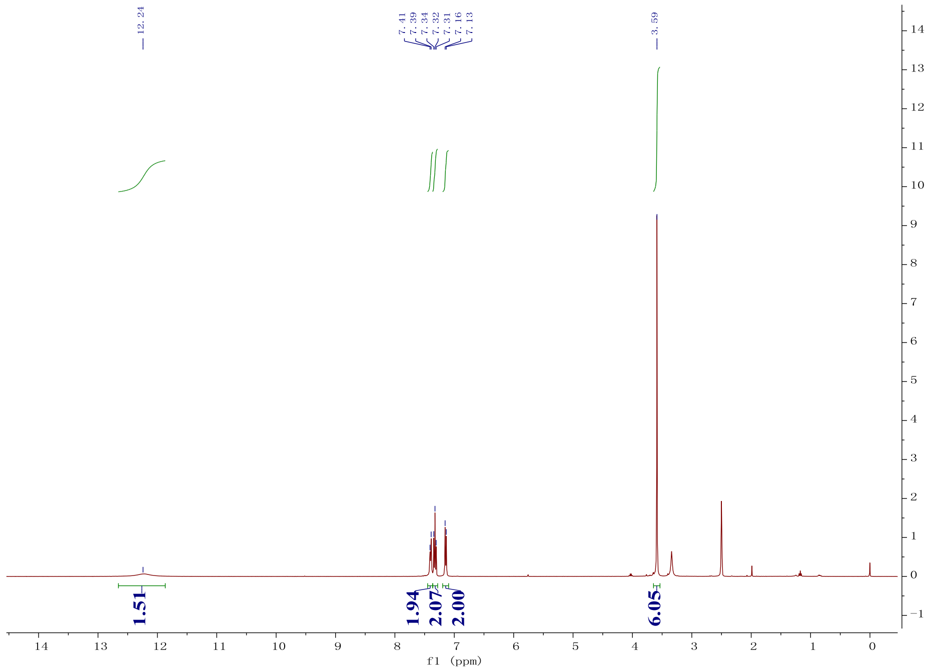

Figure S13. ^{1}H NMR spectrum of (*S*)-7, Related to Figure 2c

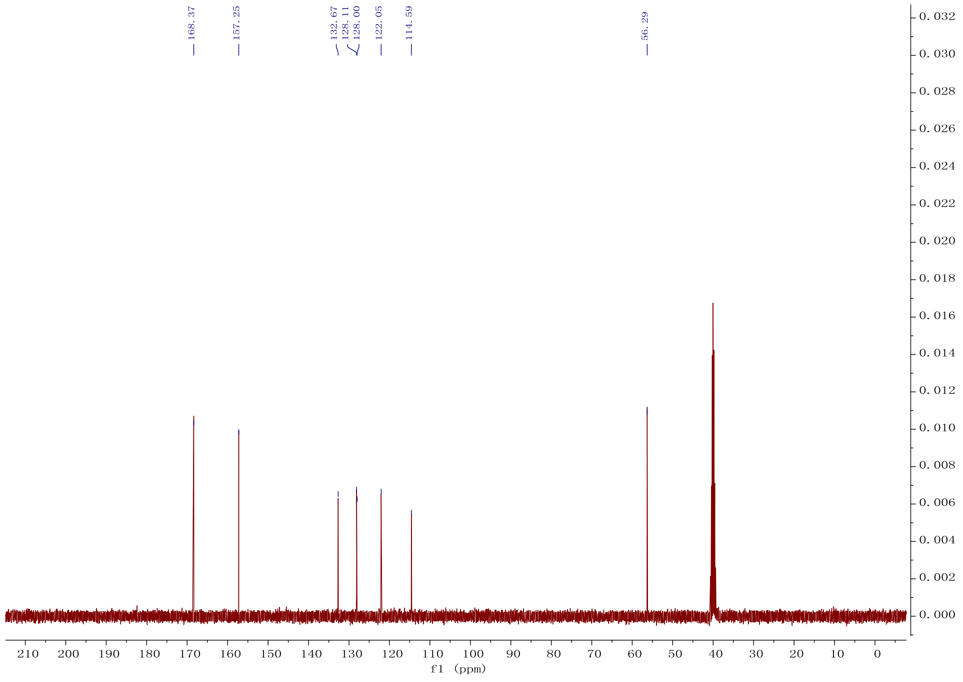

Figure S14. ^{14}C NMR spectrum of (*S*)-7, Related to Figure 2c

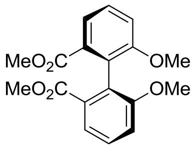

(*S*)-**8**

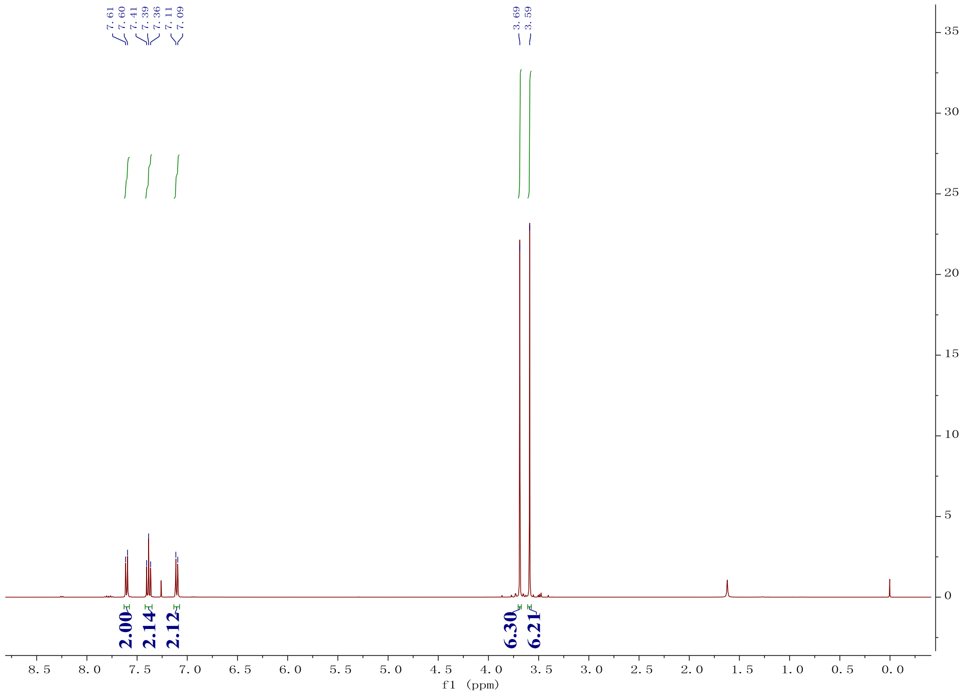

Figure S15. ^{1}H NMR spectrum of (*S*)-8, Related to Figure 2c

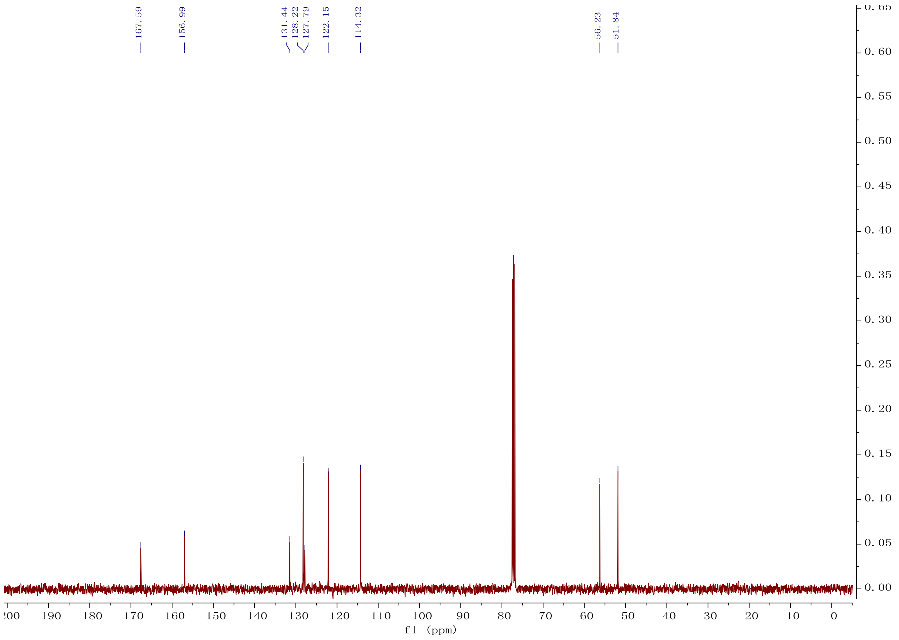

Figure S16. ^{13}C NMR spectrum of (*S*)-8, Related to Figure 2c

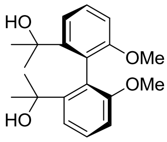

(*S*)-**9**

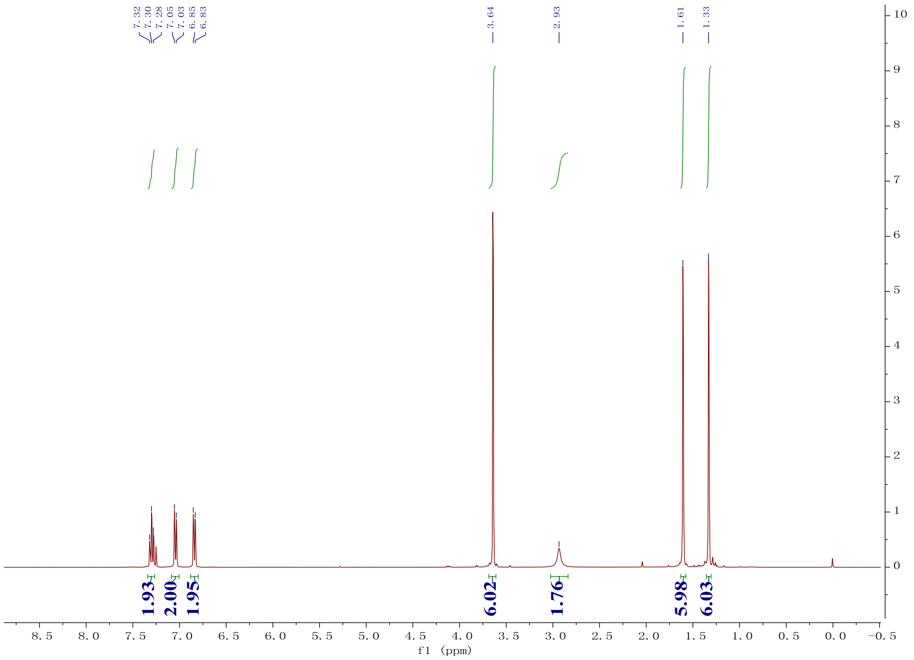

Figure S17. ^{1}H NMR spectrum of (*S*)-9, Related to Figure 2c

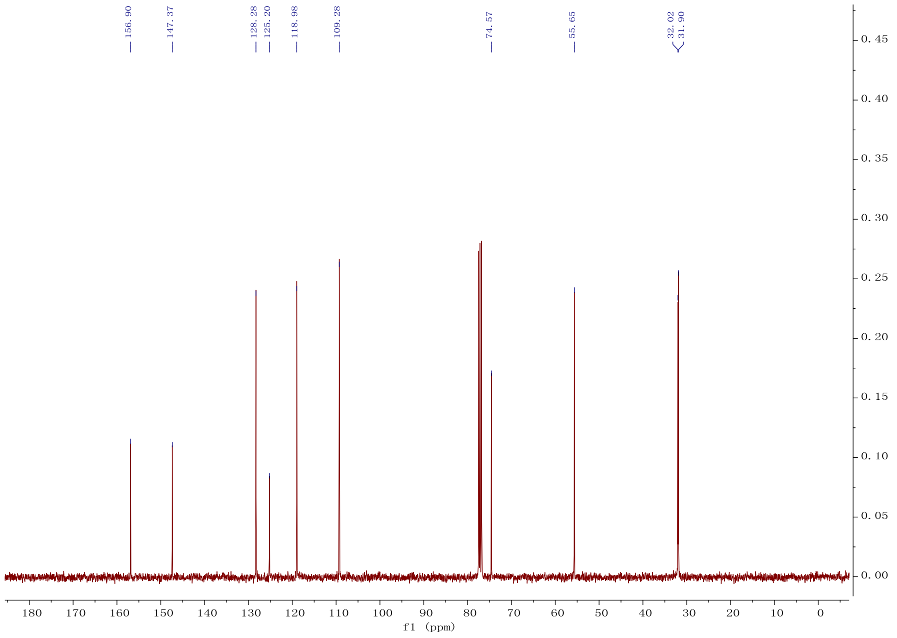

Figure S18. ^{13}C NMR spectrum of (*S*)-9, Related to Figure 2c

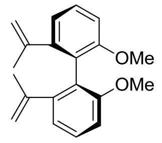

(*S*)-**10**

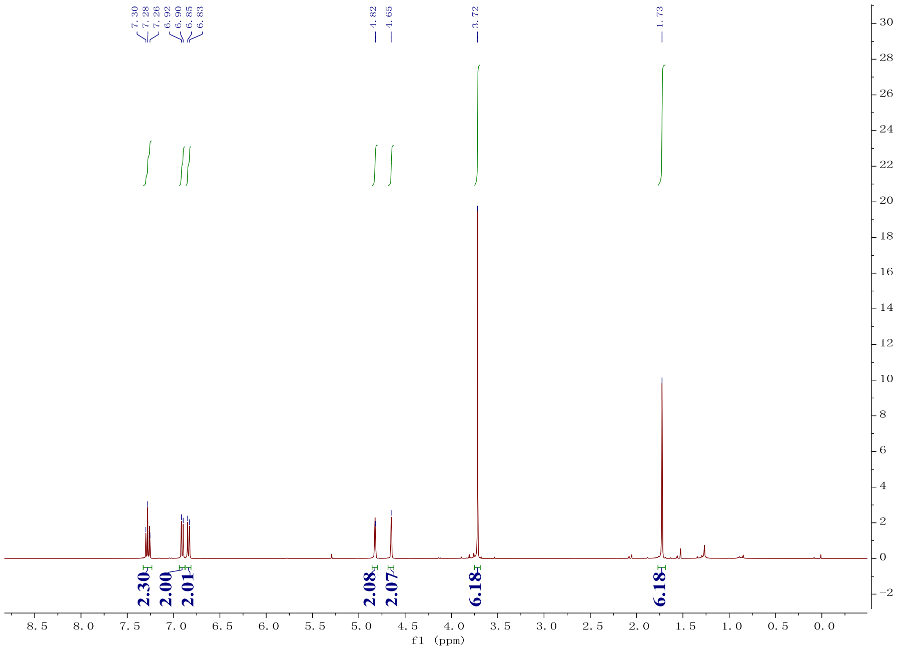

Figure S19. ^{1}H NMR spectrum of (*S*)-10, Related to Figure 2c

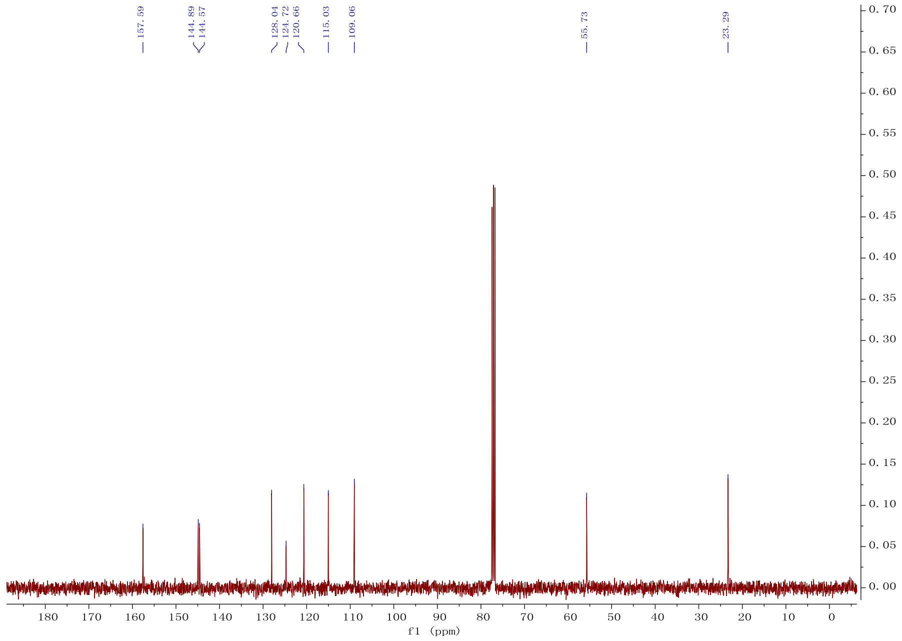

Figure S20. ^{13}C NMR spectrum of (*S*)-10, Related to Figure 2c

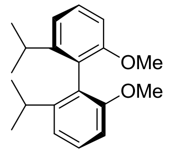

(*S*)-**11**

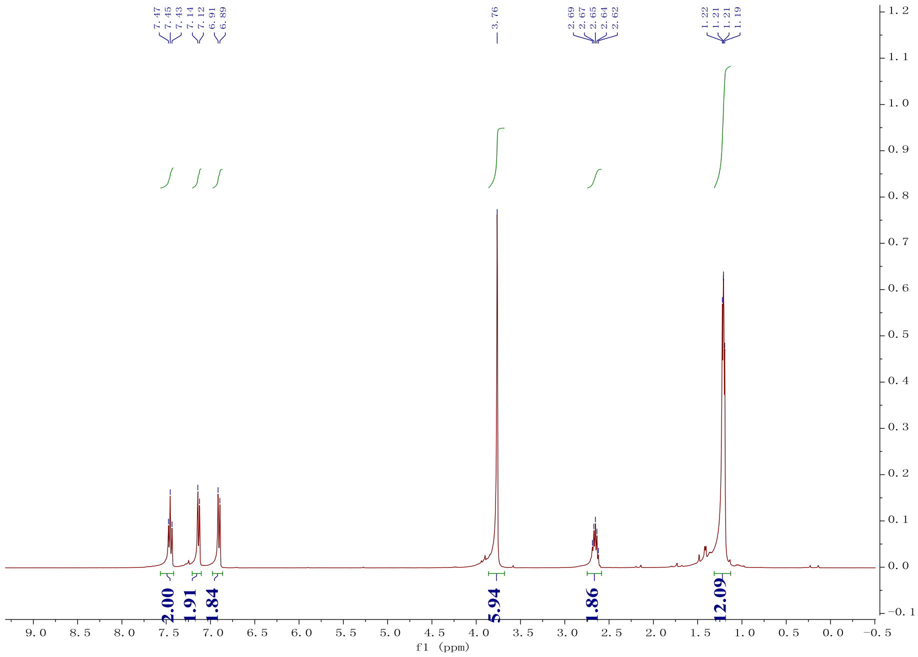

Figure S21. ^{1}H NMR spectrum of (*S*)-11, Related to Figure 2c

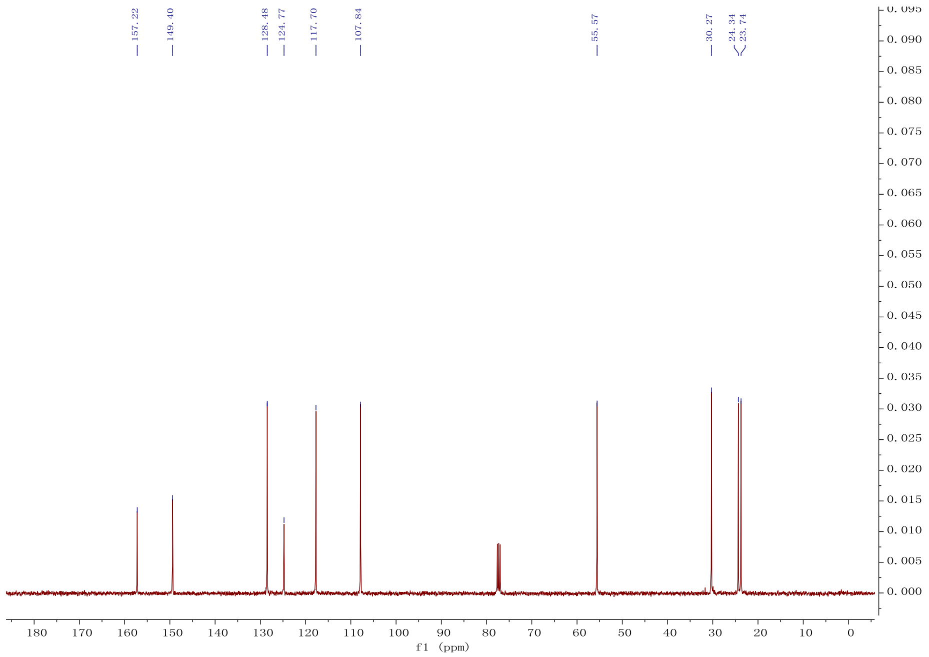

Figure S22. ^{13}C NMR spectrum of (*S*)-11, Related to Figure 2c

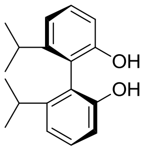

(*S*)-**L3**

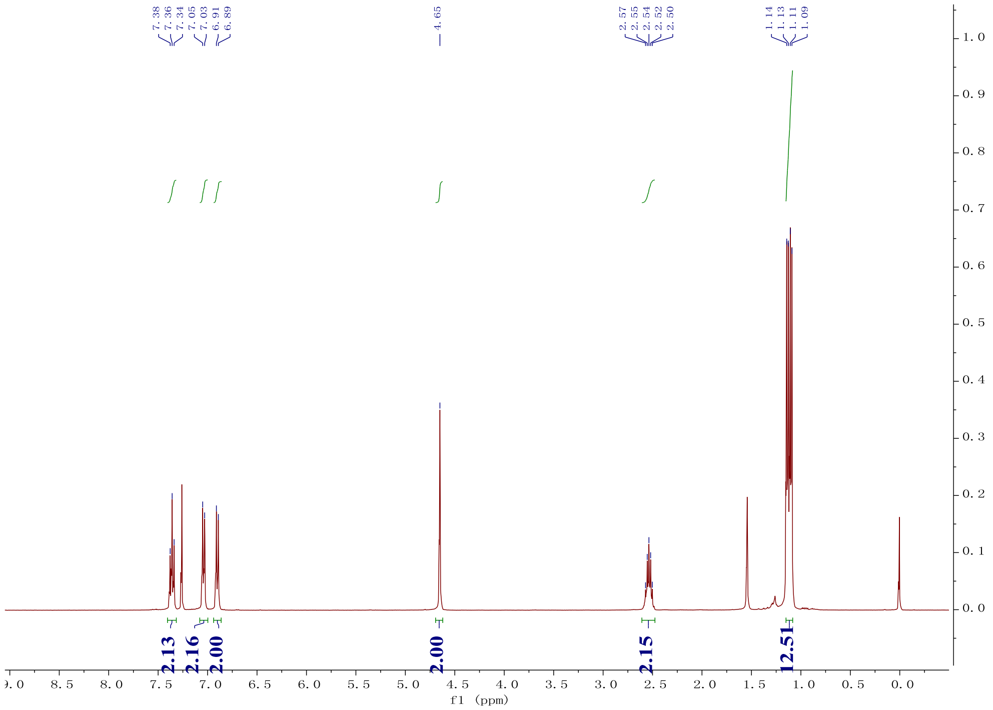

Figure S23. ^{1}H NMR spectrum of (*S*)-L3, Related to Figure 2c

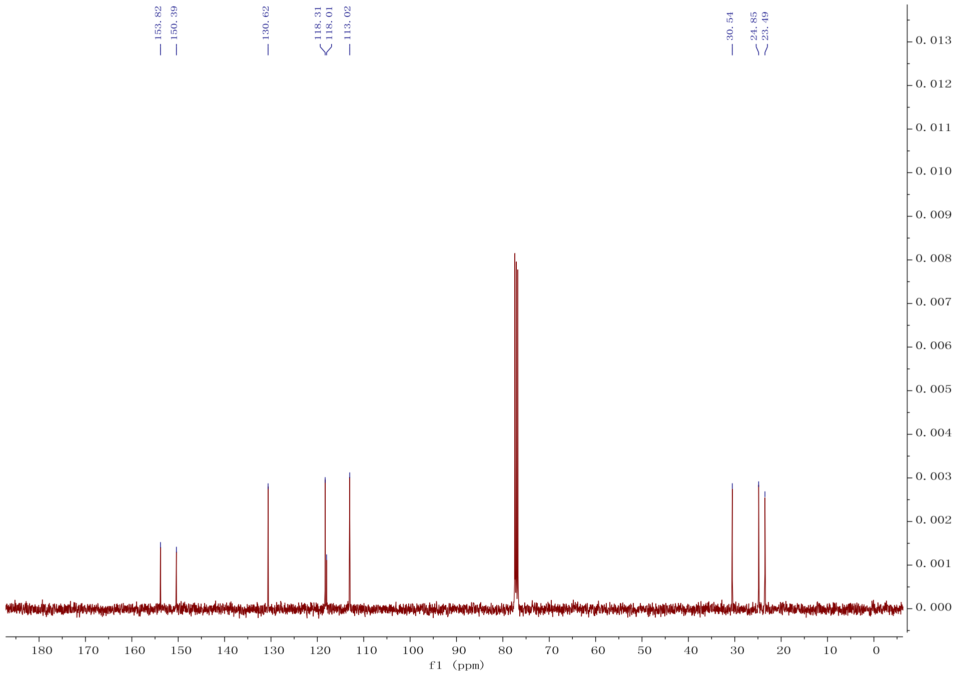

Figure S24. ^{13}C NMR spectrum of (*S*)-L3, Related to Figure 2c

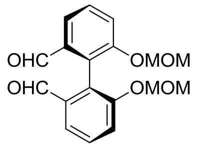

(*S*)-**13**

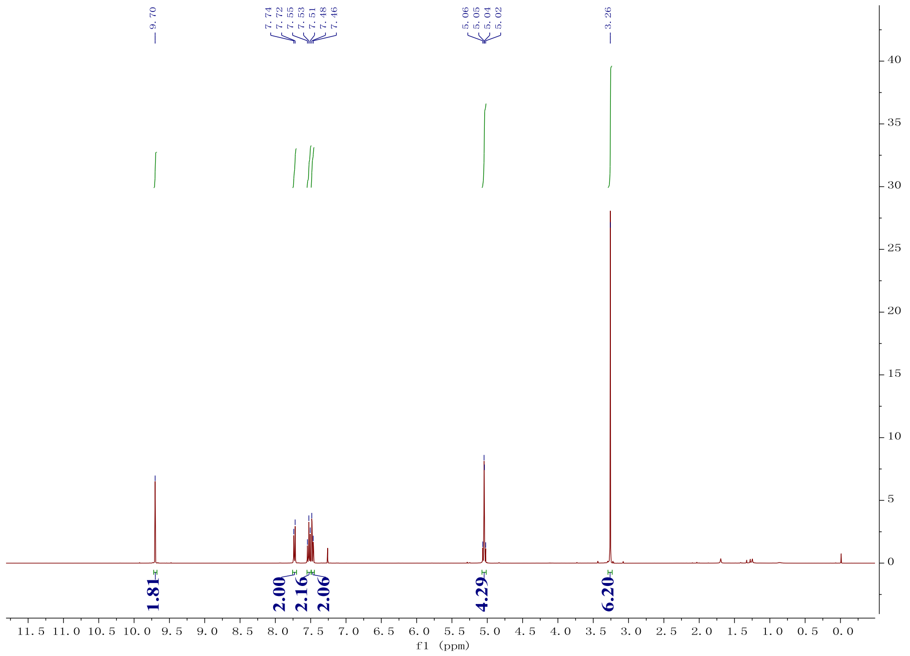

Figure S25. ¹H NMR spectrum of (*S*)-13, Related to Figure 2d

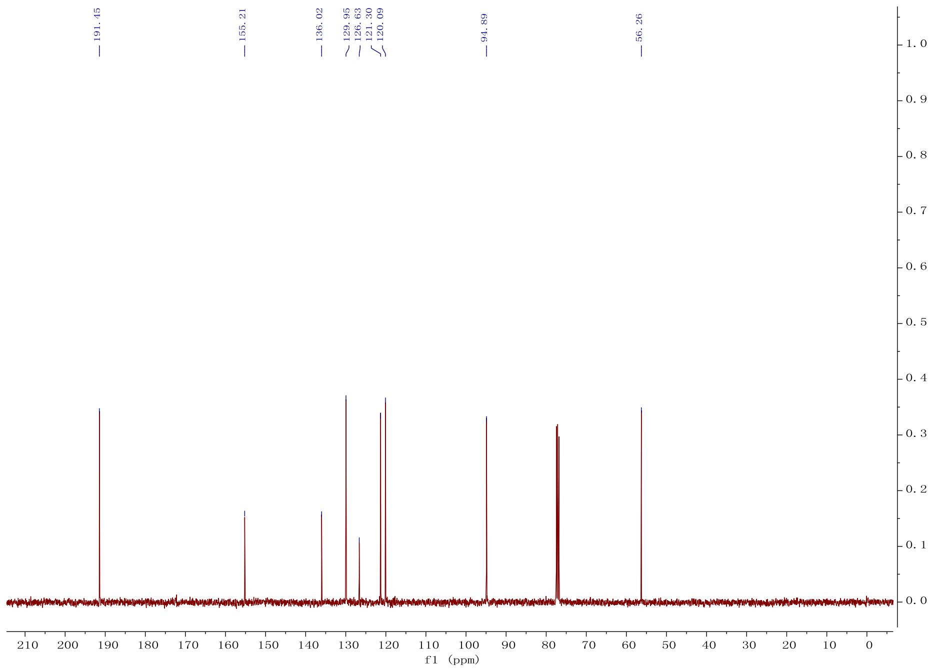

Figure S26. ¹³C NMR spectrum of (*S*)-13, Related to Figure 2d

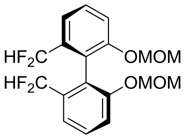

(*S*)-**14**

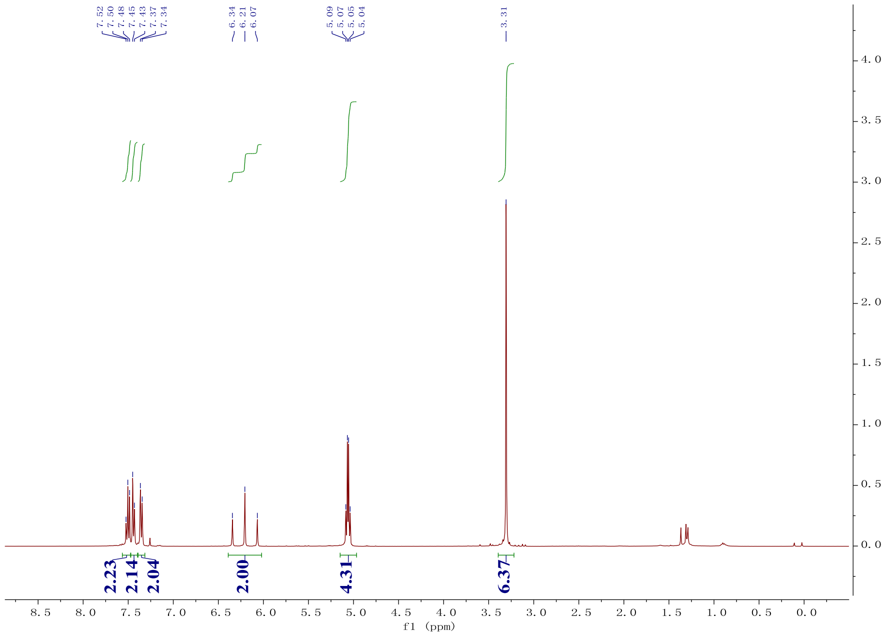

Figure S27. ^{1}H NMR spectrum of (*S*)-14, Related to Figure 2d

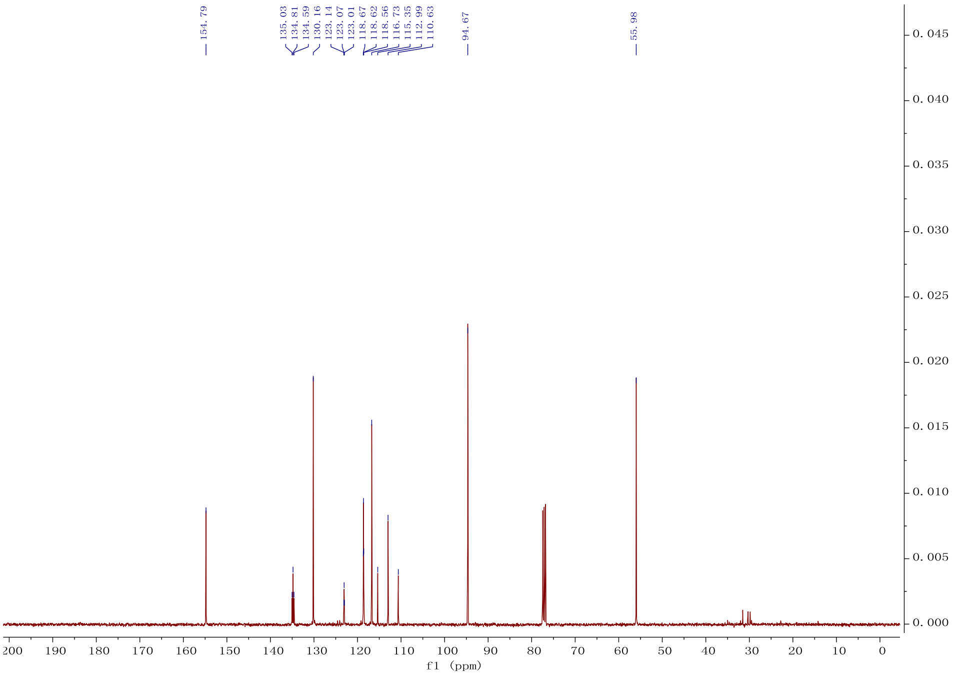

Figure S28. ^{13}C NMR spectrum of (*S*)-14, Related to Figure 2d

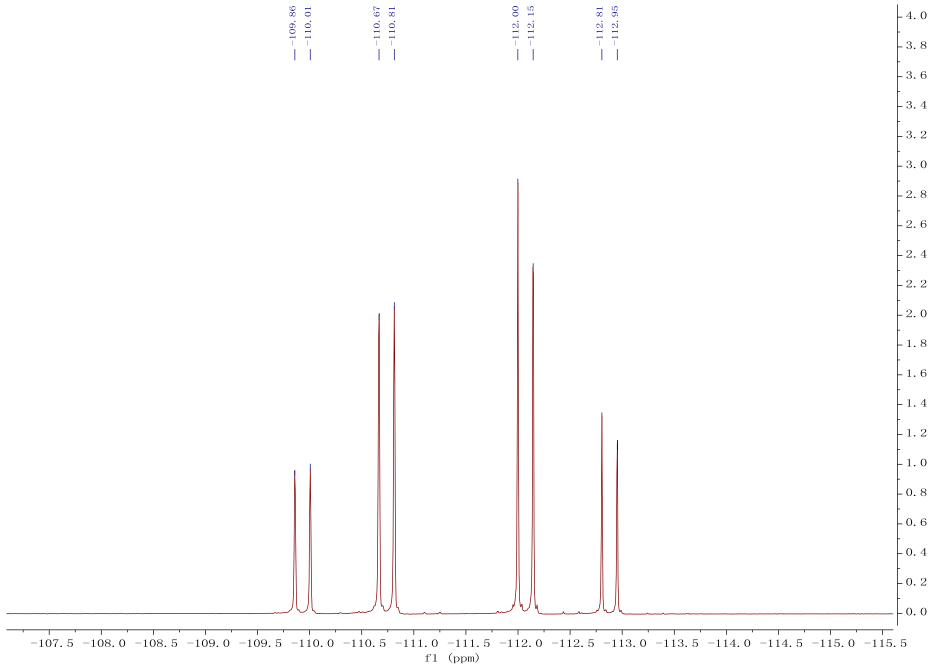

Figure S29. ^{19}**F NMR spectrum of (*S*)-14, Related to Figure 2d**

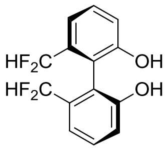

(*S*)-**L4**

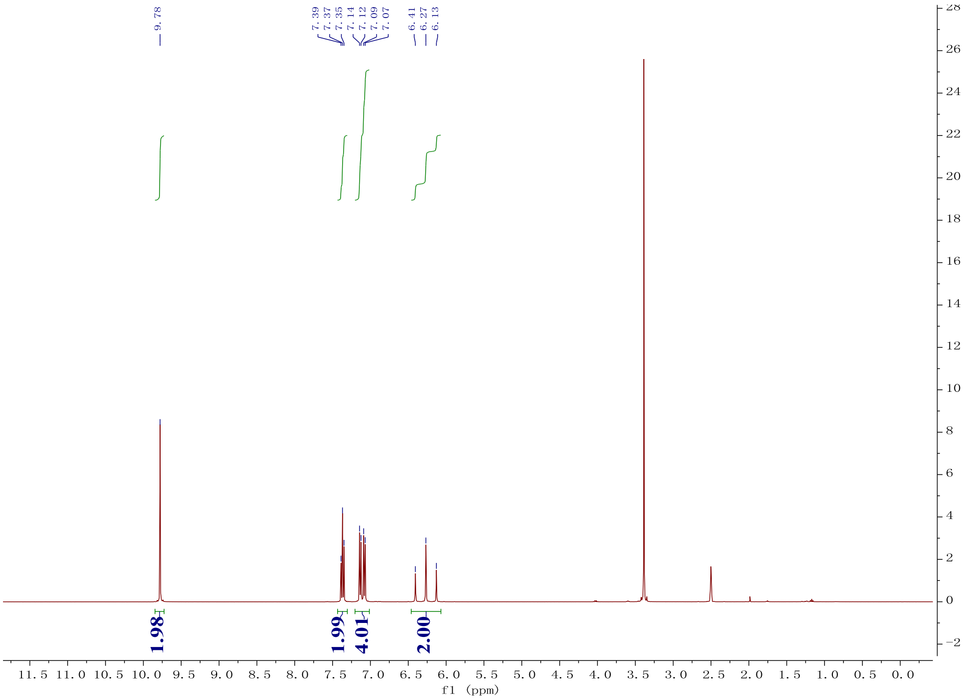

Figure S30. ^{1}**H NMR spectrum of (*S*)-L4, Related to Figure 2d**

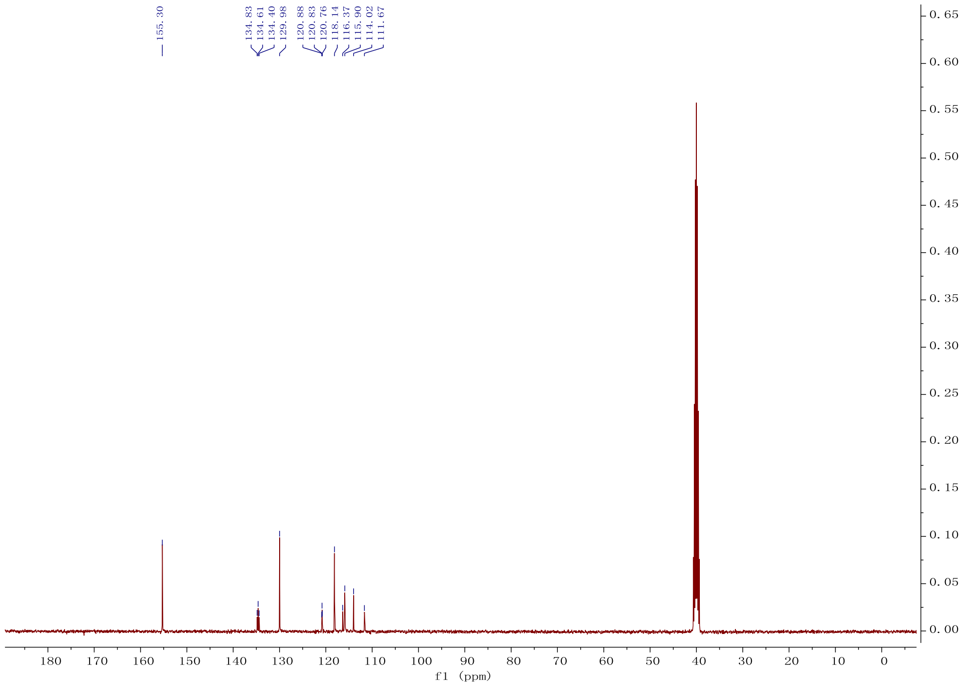

Figure S31. ^{13}C NMR spectrum of (*S*)-L4, Related to Figure 2d

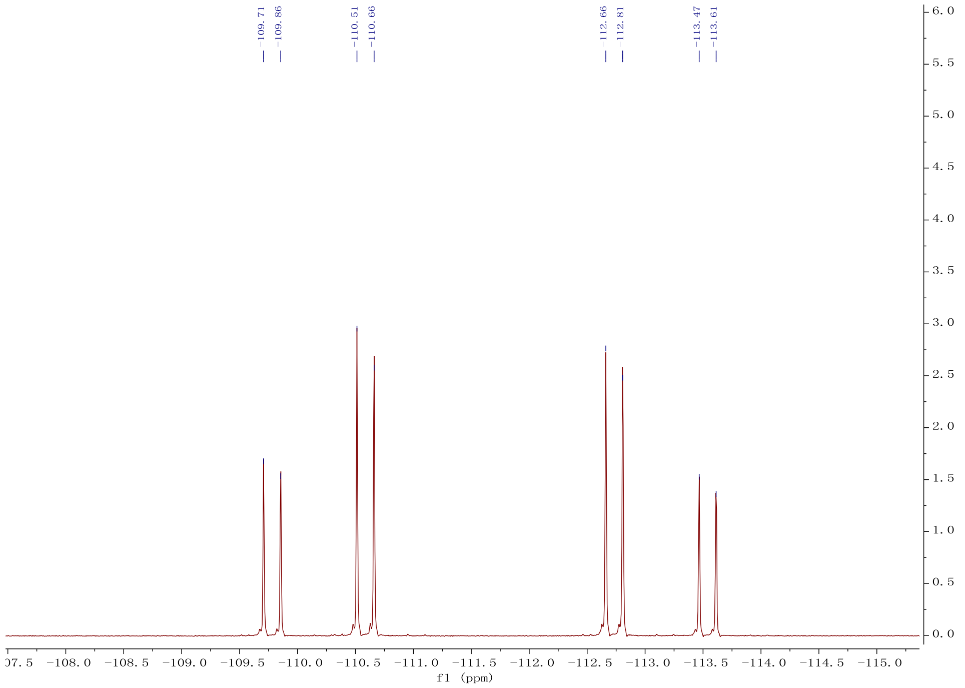

Figure S32. ^{19}F NMR spectrum of (*S*)-L4, Related to Figure 2d

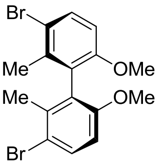

(*S*)-**15**

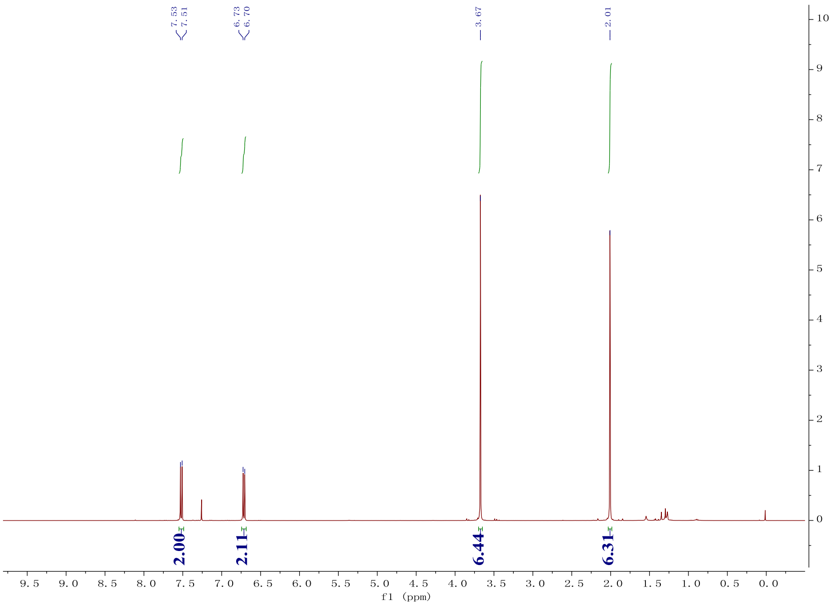

Figure S33. ^{1}H NMR spectrum of (*S*)-15, Related to Figure 3a

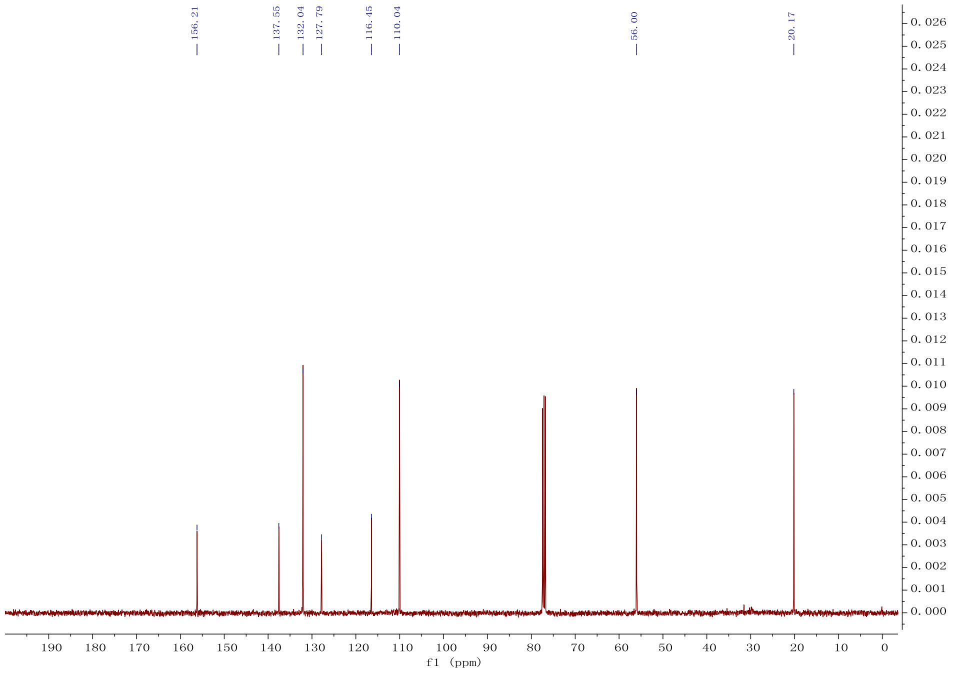

Figure S34. ^{13}C NMR spectrum of (*S*)-15, Related to Figure 3a

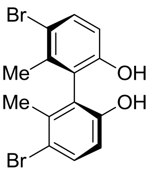

(*S*)-**L5**

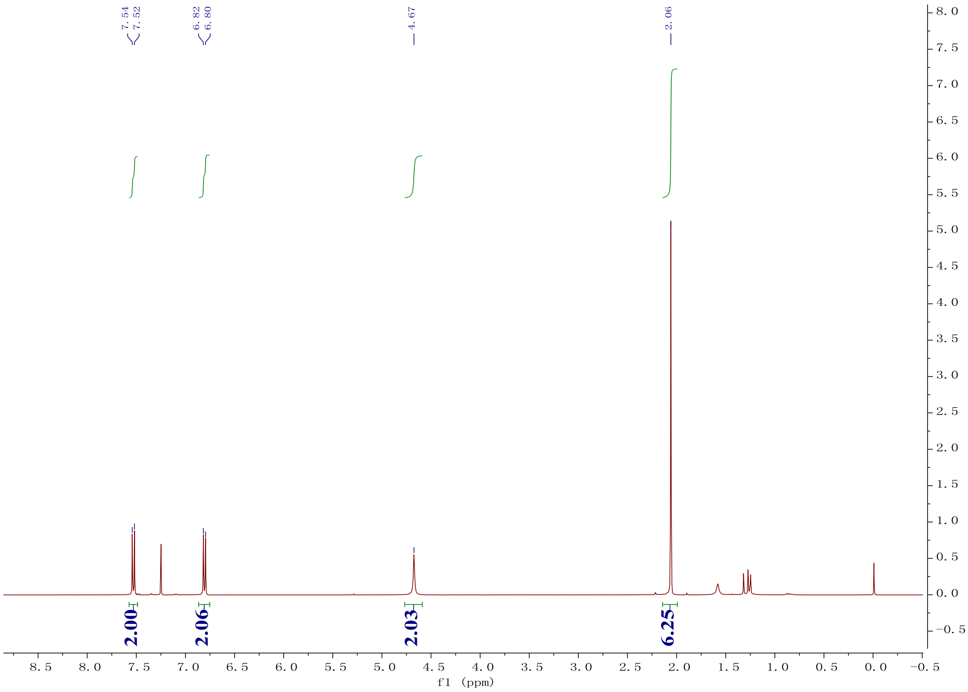

Figure S35. ^{1}H NMR spectrum of (*S*)-L5, Related to Figure 3a

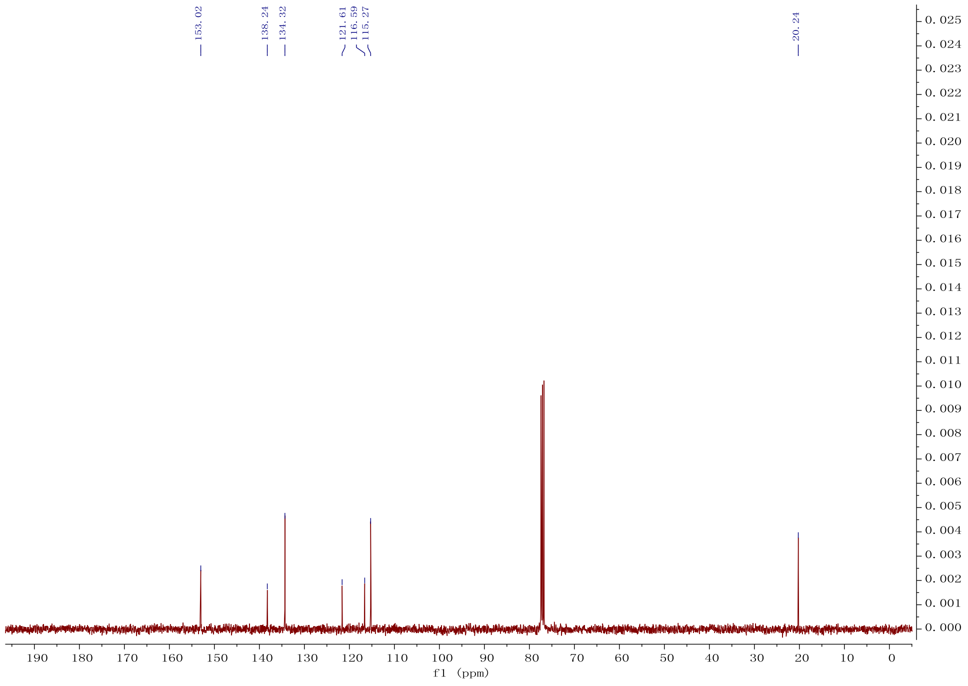

Figure S36. ^{13}C NMR spectrum of (*S*)-L5, Related to Figure 3a

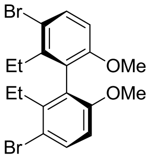

(*S*)-**16**

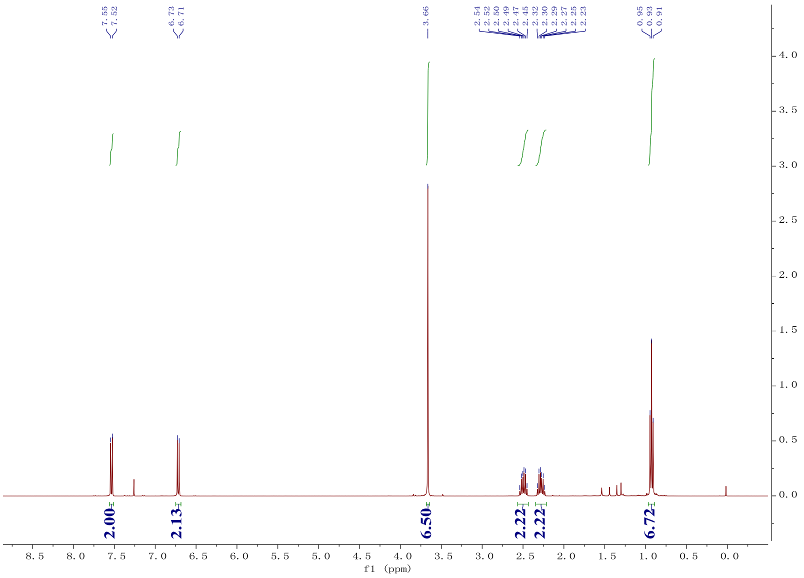

Figure S37. ^{1}H NMR spectrum of (*S*)-16, Related to Figure 3b

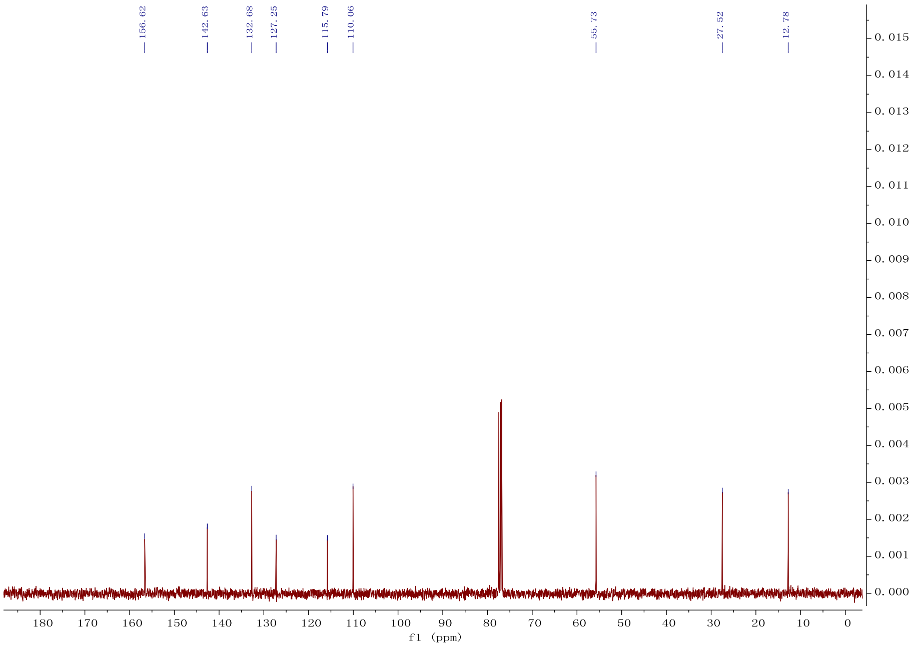

Figure S38. ^{13}C NMR spectrum of (*S*)-16, Related to Figure 3b

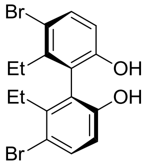

(*S*)-**L6**

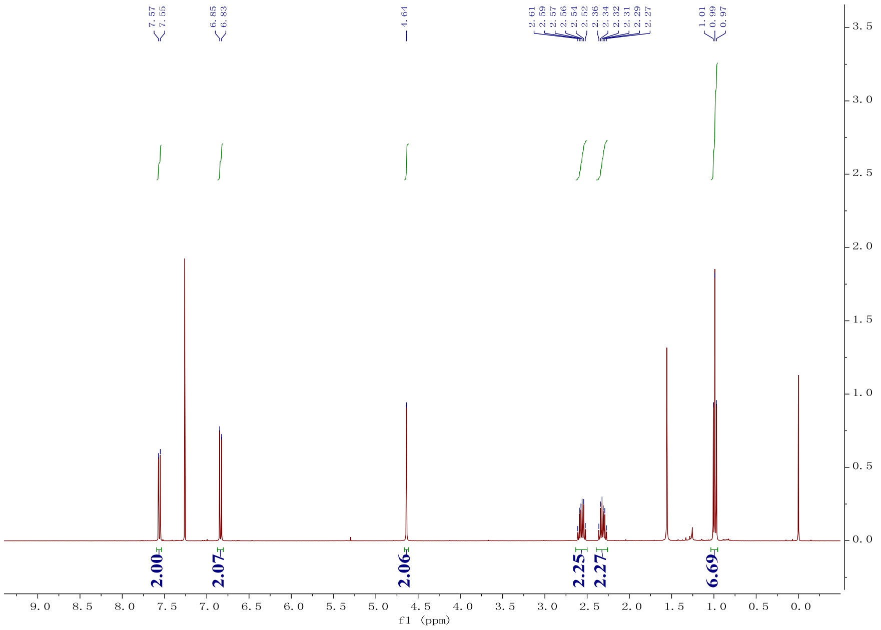

Figure S39. ^{1}H NMR spectrum of (*S*)-L6, Related to Figure 3b

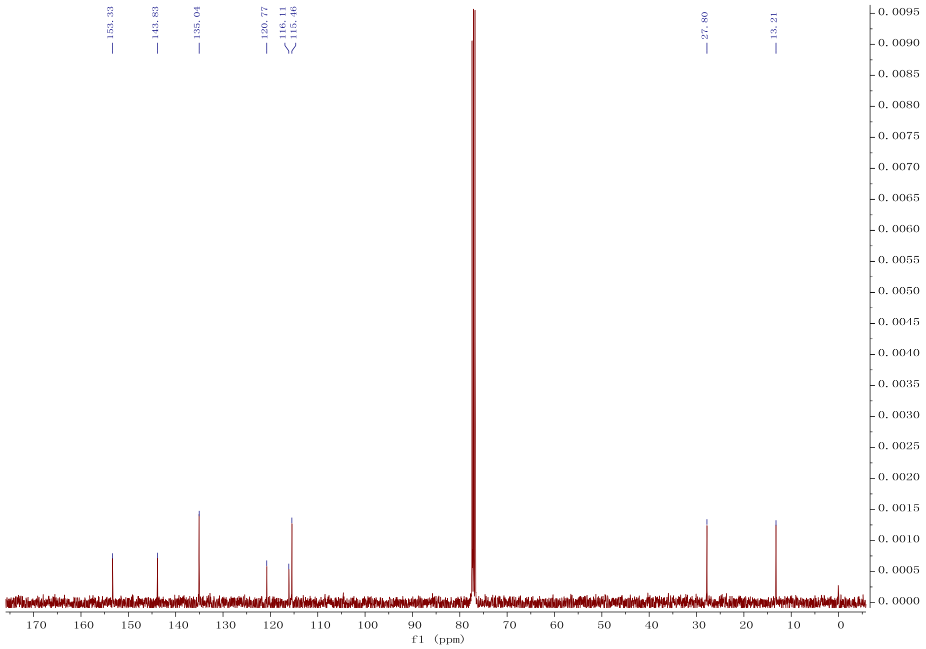

Figure S40. ^{13}C NMR spectrum of (*S*)-L6, Related to Figure 3b

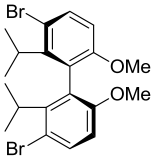

(*S*)-**17**

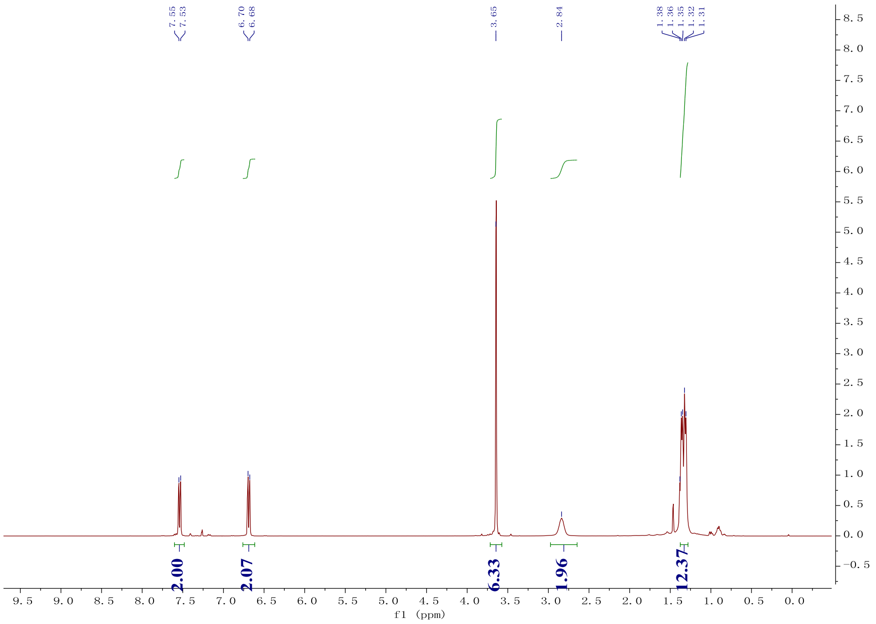

Figure S41. ^{1}H NMR spectrum of (*S*)-17, Related to Figure 3c

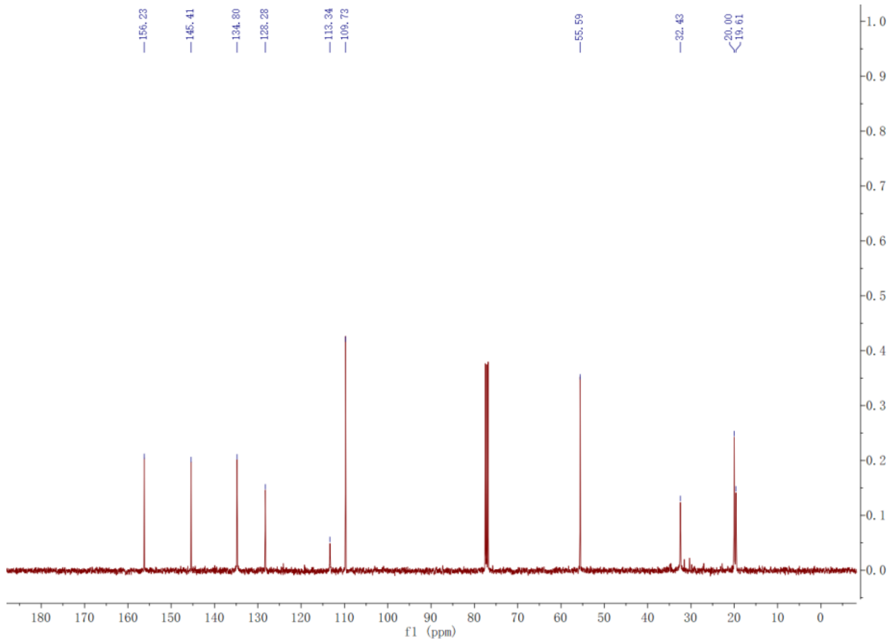

Figure S42. ^{13}C NMR spectrum of (*S*)-17, Related to Figure 3c

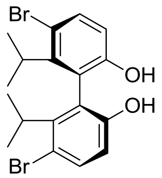

(*S*)-**L7**

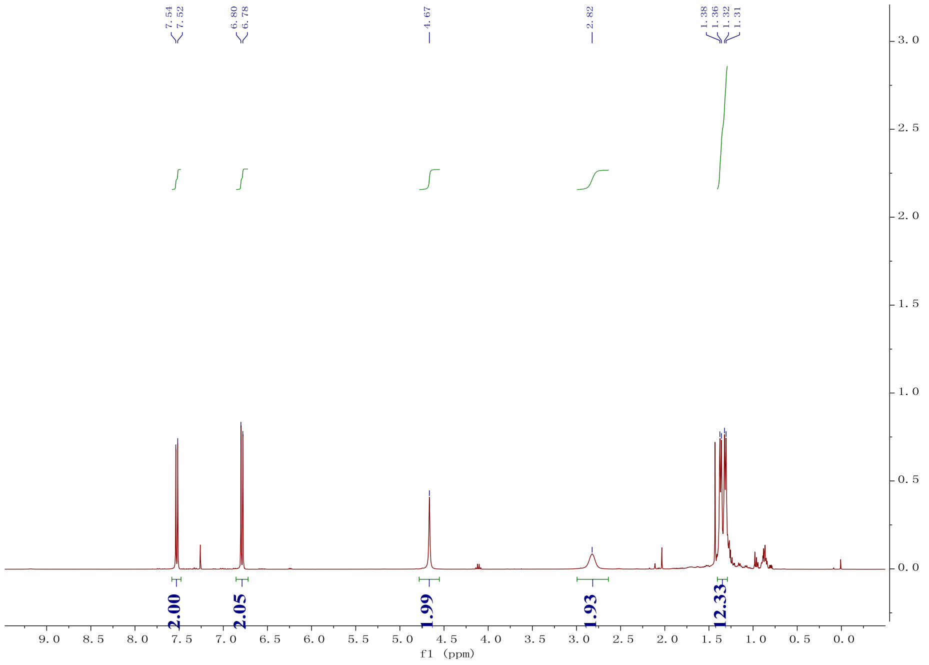

Figure S43. ^{1}H NMR spectrum of (*S*)-L7, Related to Figure 3c

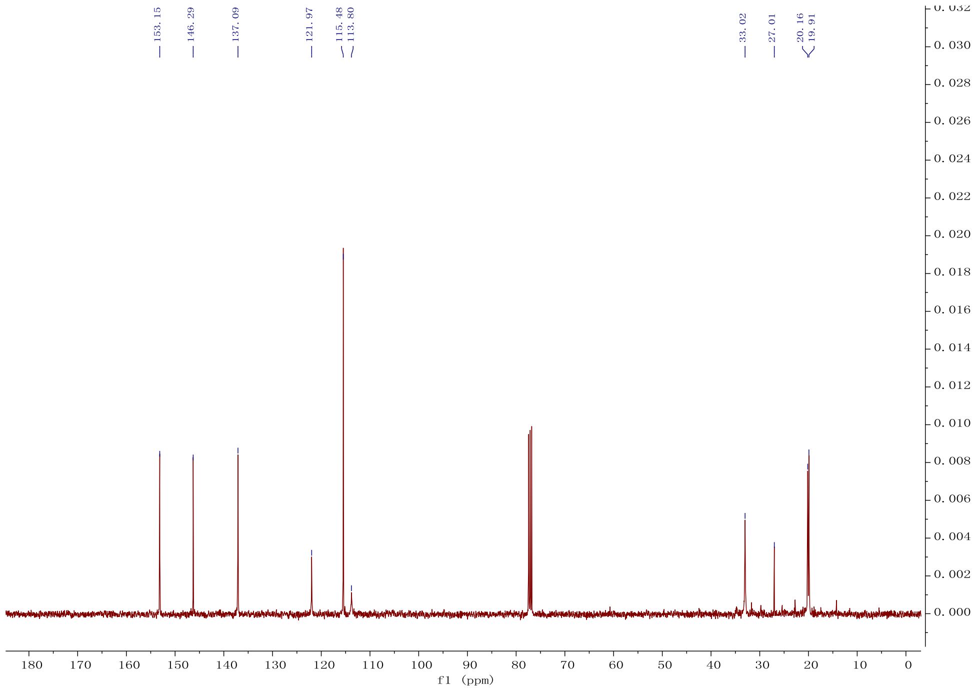

Figure S44. ^{13}C NMR spectrum of (*S*)-L7, Related to Figure 3c

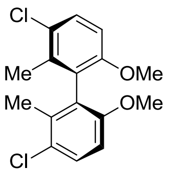

(*S*)-**18**

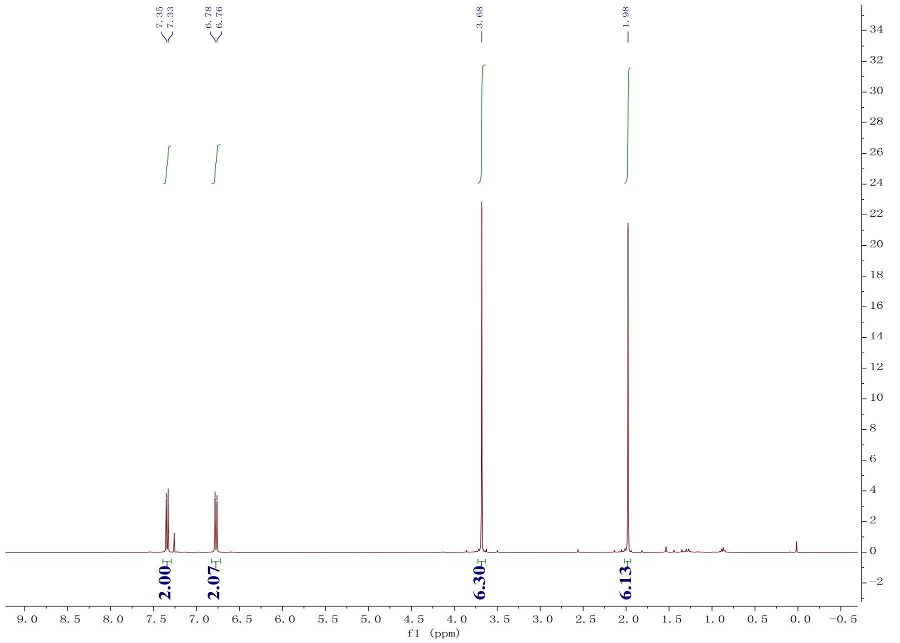

Figure S45. ^{1}H NMR spectrum of (*S*)-18, Related to Figure 3d

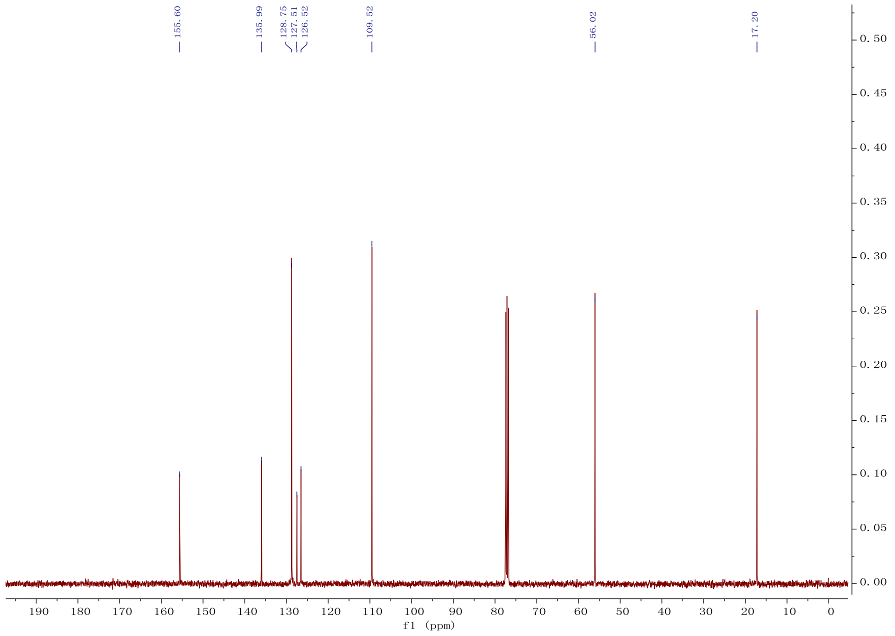

Figure S46. ^{13}C NMR spectrum of (*S*)-18, Related to Figure 3d

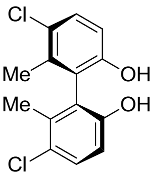

(S)-**L8**

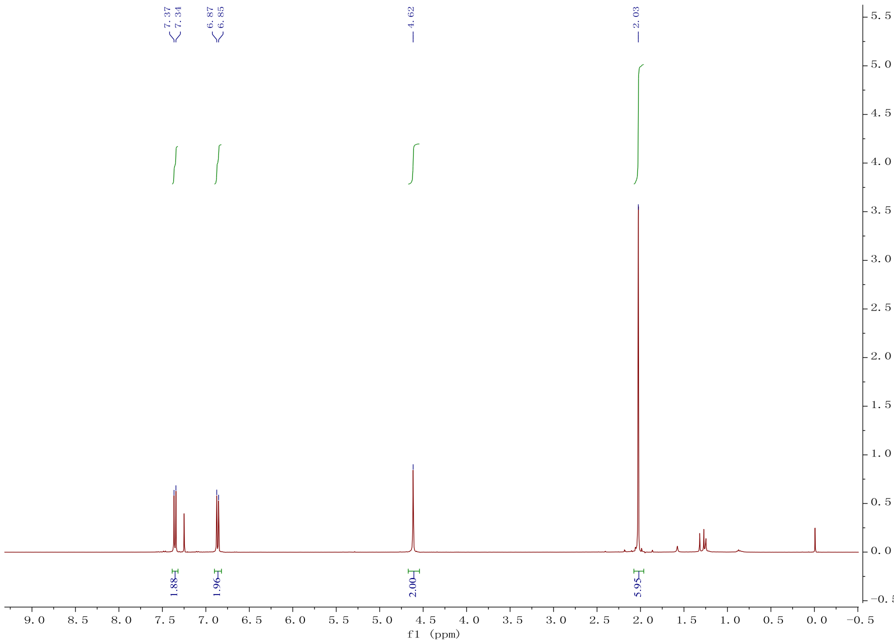

Figure S47. ¹H NMR spectrum of (*S*)-L8, Related to Figure 3d

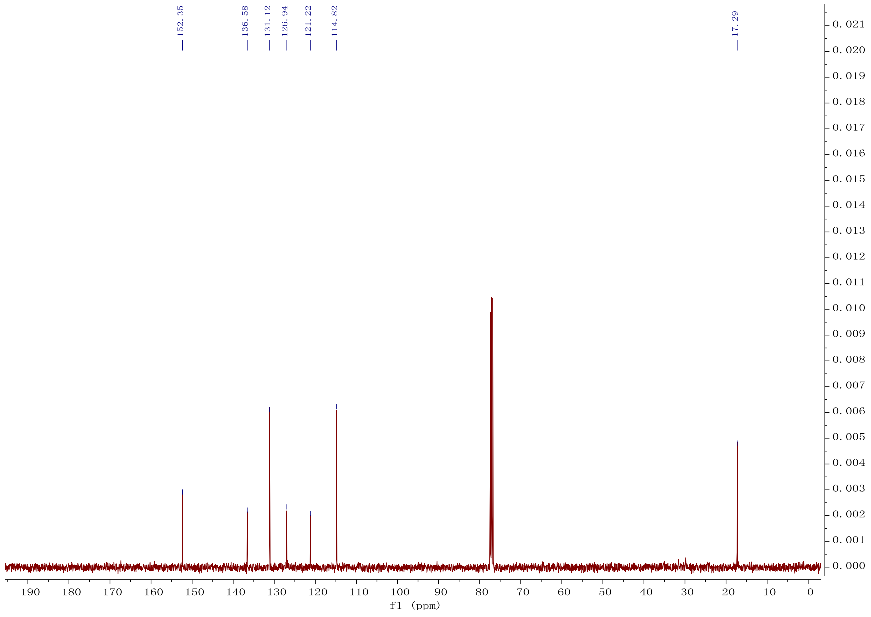

Figure S48. ¹³C NMR spectrum of (*S*)-L8, Related to Figure 3d

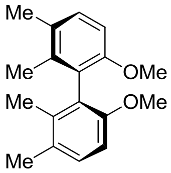

(*S*)-**19**

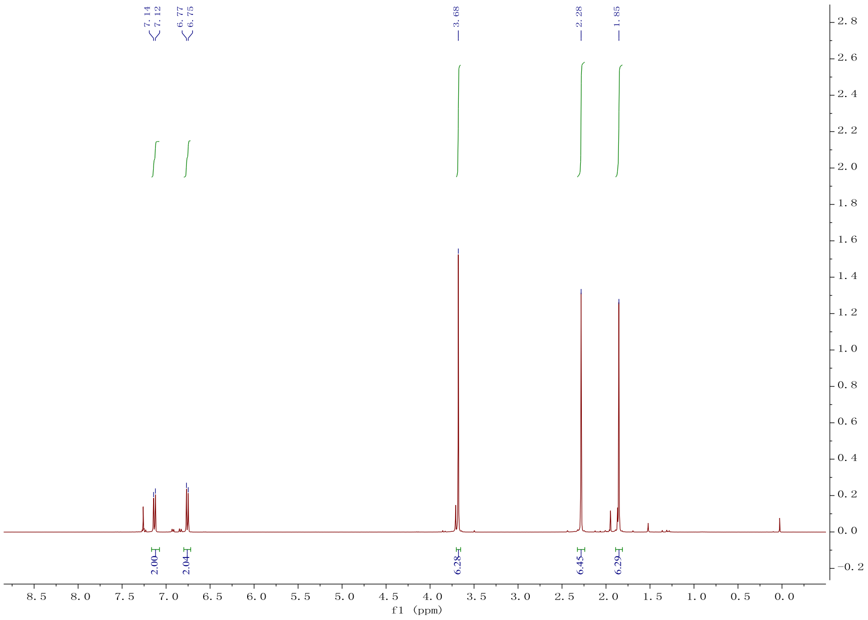

Figure S49. ^{1}H NMR spectrum of (*S*)-19, Related to Figure 4a

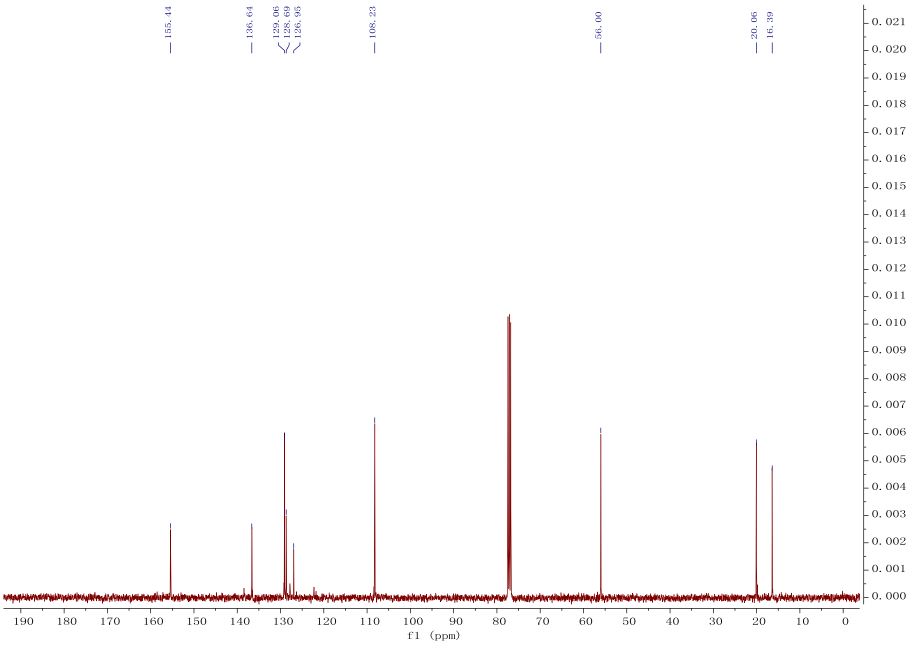

Figure S50. ^{13}C NMR spectrum of (*S*)-19, Related to Figure 4a

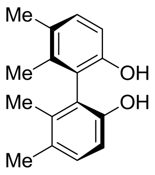

(*S*)-**L9**

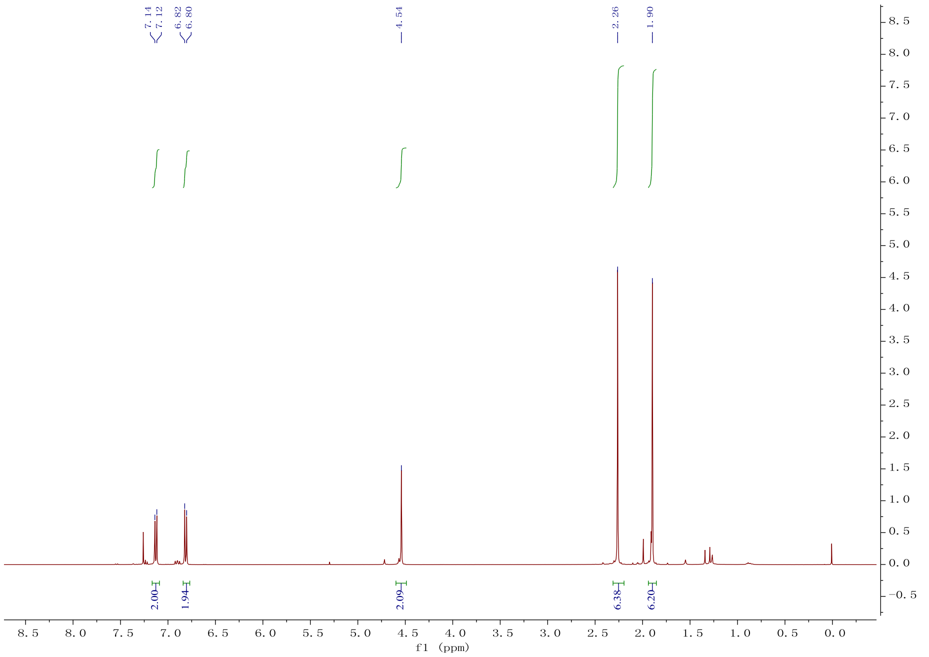

Figure S51. ^{1}H NMR spectrum of (*S*)-L9, Related to Figure 4a

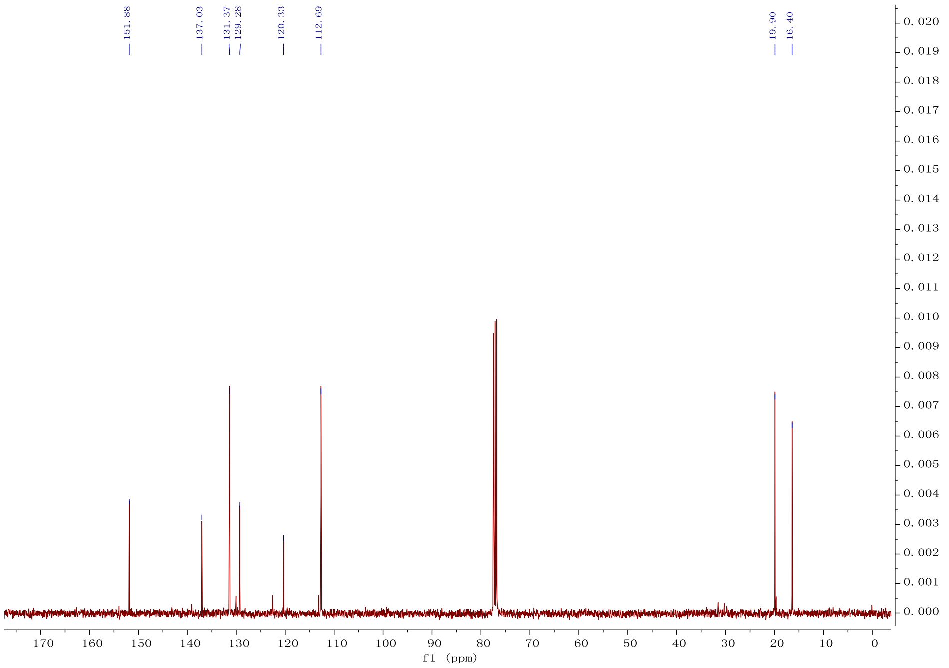

Figure S52. ^{13}C NMR spectrum of (*S*)-L9, Related to Figure 4a

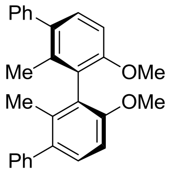

(S)-**20**

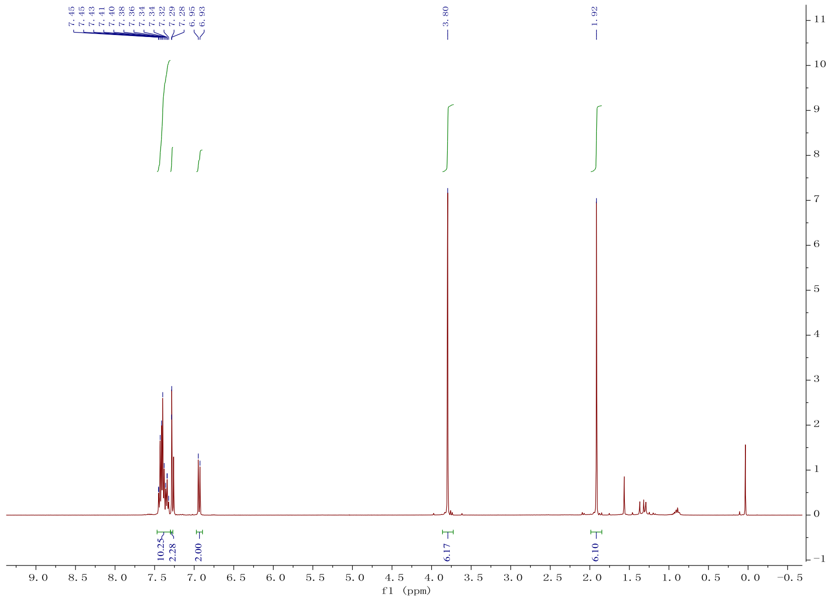

Figure S53. ^{1}H NMR spectrum of (*S*)-20, Related to Figure 4b

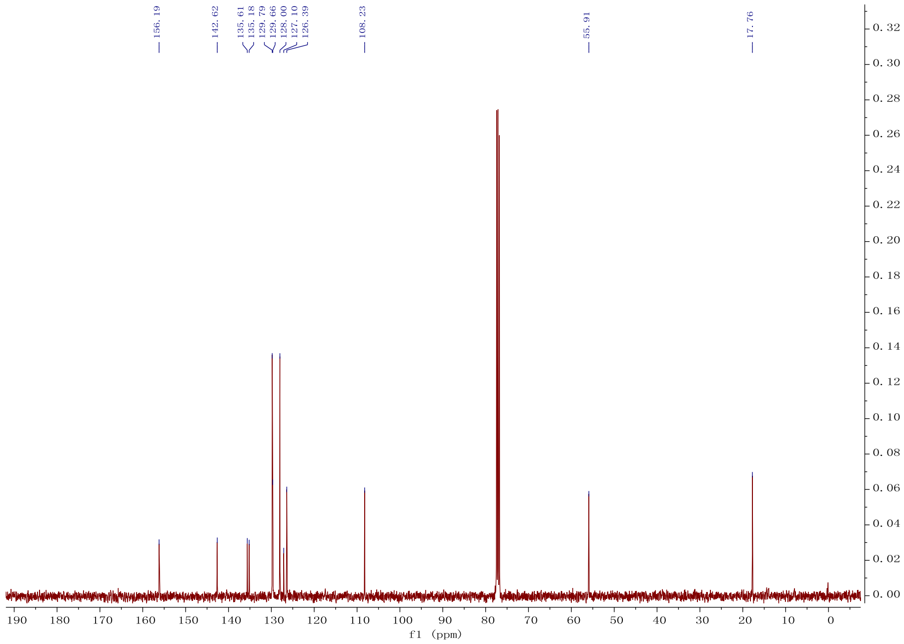

Figure S54. ^{13}C NMR spectrum of (*S*)-20, Related to Figure 4b

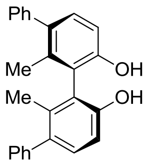

(S)-**L10**

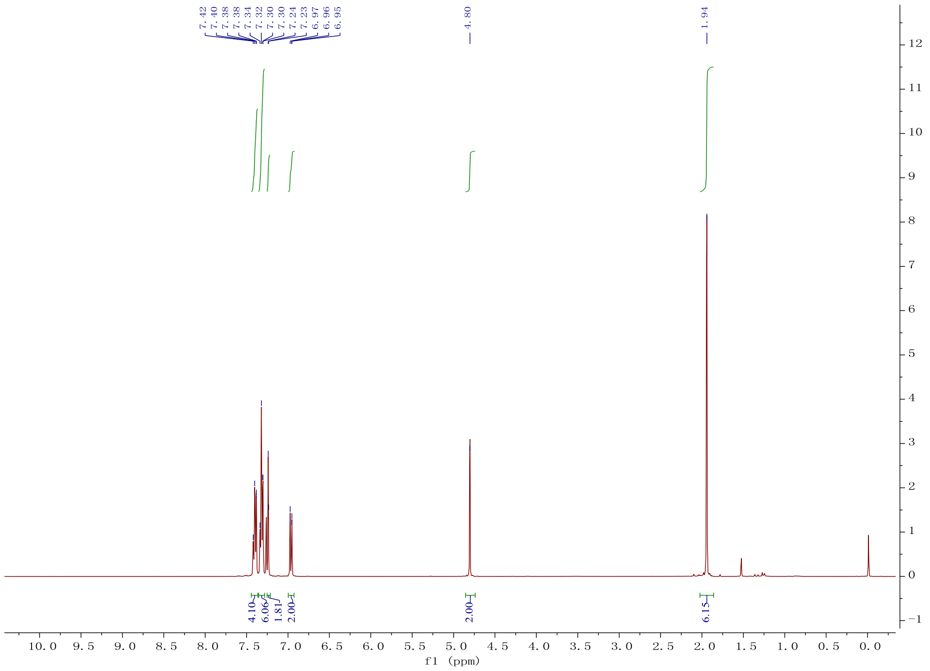

Figure S55. ^{1}H NMR spectrum of (*S*)-L10, Related to Figure 4b

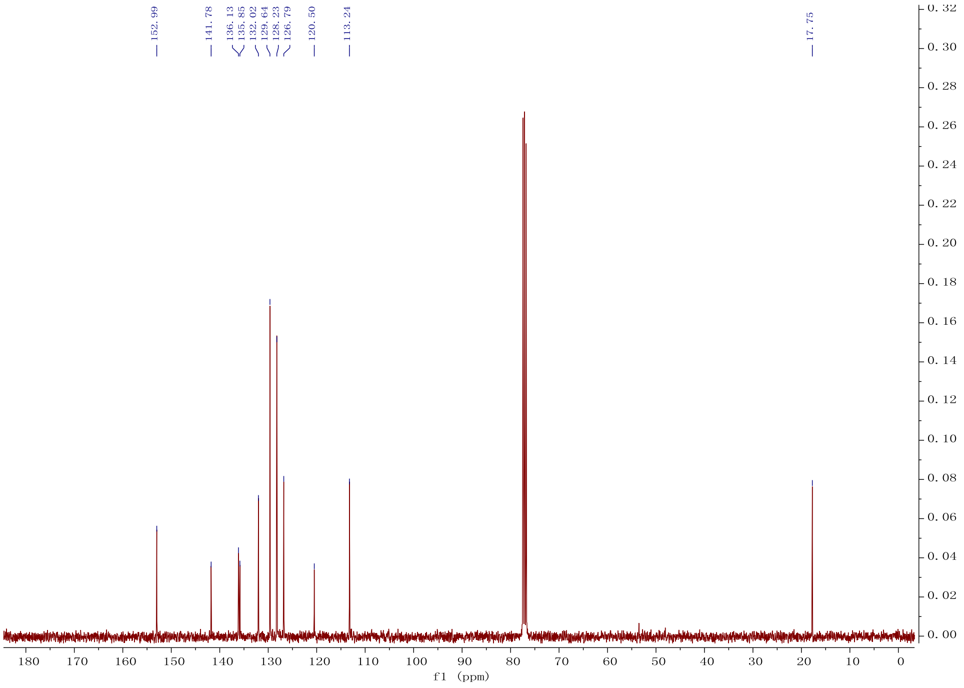

Figure S56. ^{13}C NMR spectrum of (*S*)-L10, Related to Figure 4b

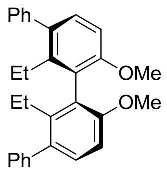

(*S*)-**21**

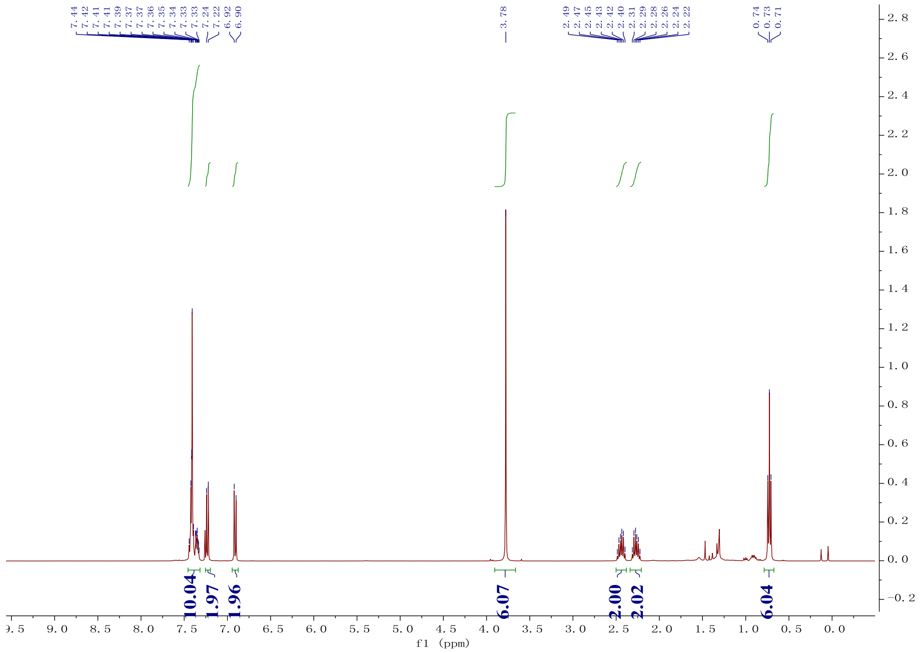

Figure S57. ^{1}H NMR spectrum of (*S*)-21, Related to Figure 4c

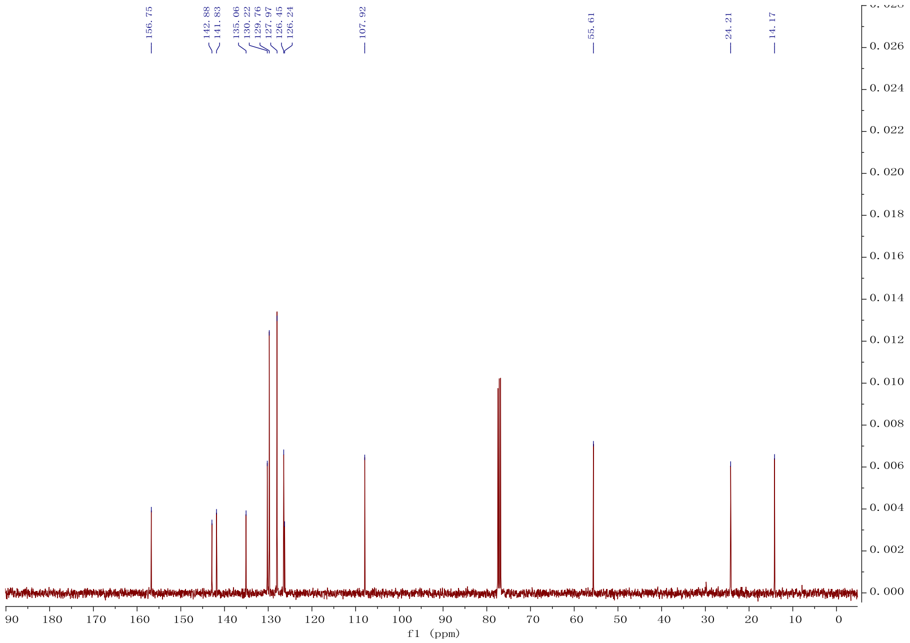

Figure S58. ^{13}C NMR spectrum of (*S*)-21, Related to Figure 4c

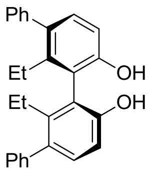

(*S*)-**L11**

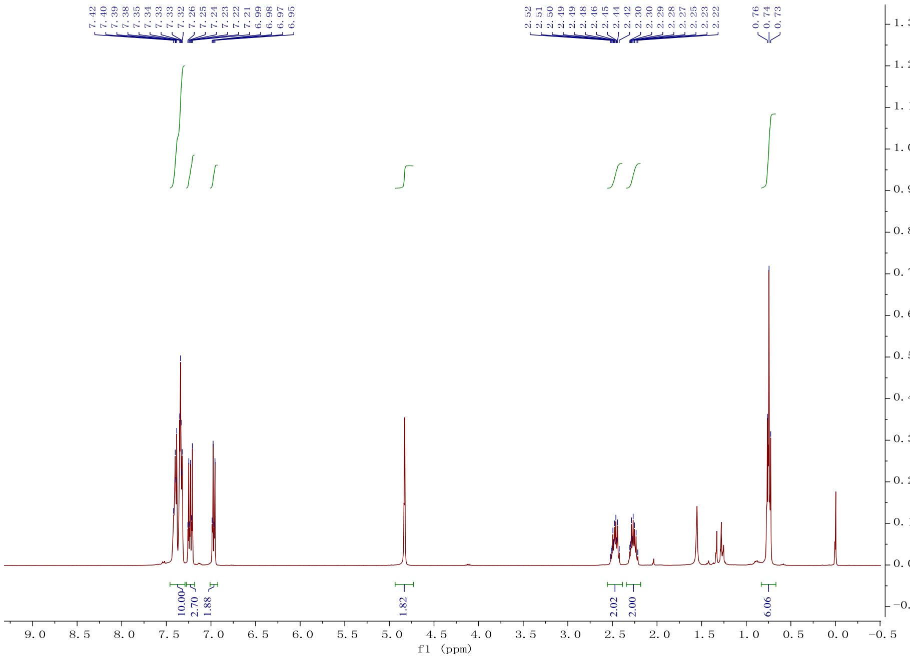

Figure S59. ^{1}H NMR spectrum of (*S*)-L11, Related to Figure 4c

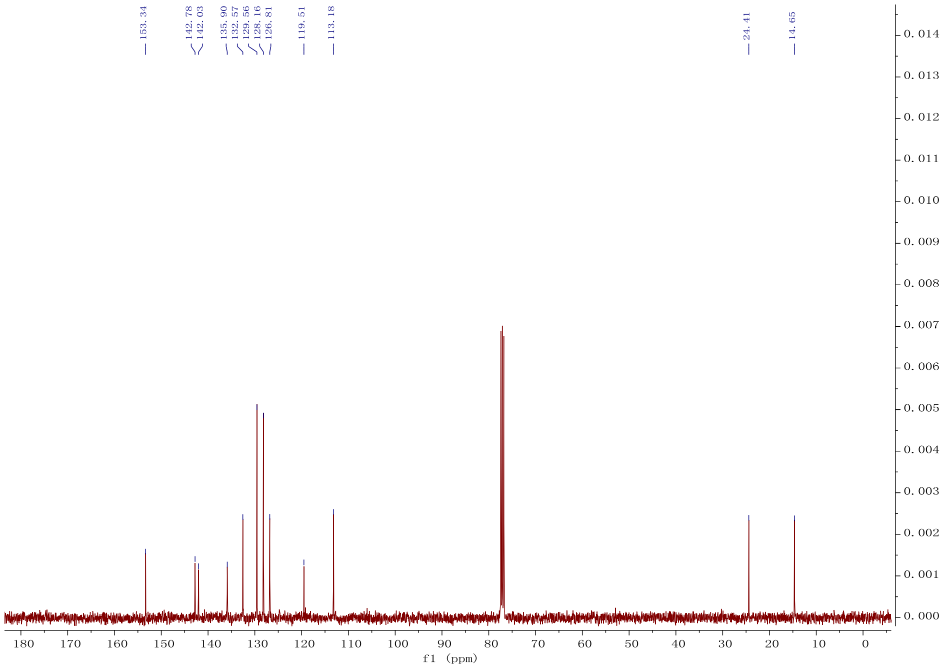

Figure S60. ^{13}C NMR spectrum of (*S*)-L11, Related to Figure 4c

X-Ray crystallographic data for (*S*)-L1

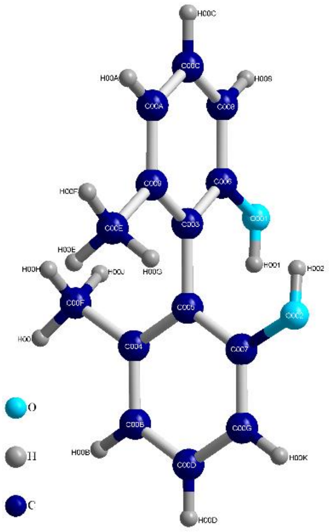

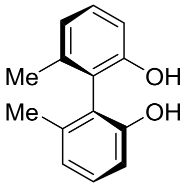

(*S*)-**L1**

Table S1. Crystal data and structure refinement for (*S*)-L1 (CCDC 2209953).

Related to STAR methods.

Identification code	**(*S*)-L1**
Empirical formula	$C_{14}H_{14}O_2$
Formula weight	214.10
Temperature/K	150.00
Crystal system	orthorhombic
Space group	$P2_12_12_1$
a/Å	7.4035(4)
b/Å	7.5182(4)
c/Å	20.2558(12)
α/°	90
β/°	90
γ/°	90
Volume/Å^3	1127.46(11)
Z	1
ρ_{calc}g/cm^3	0.043
μ/mm^{-1}	0.004
F(000)	15.0
Crystal size/mm^3	0.21 × 0.14 × 0.11
Radiation	MoKα (λ = 0.71073)
2Θ range for data collection/°	4.022 to 56.71
Index ranges	$-9 \le h \le 9, -10 \le k \le 10, -25 \le l \le 27$
Reflections collected	9669

Independent reflections	2786 [R_{int} = 0.0485, R_{sigma} = 0.0507]
Data/restraints/parameters	2786/0/149
Goodness-of-fit on F^2	1.095
Final R indexes [I>=2σ (I)]	R_1 = 0.0447, wR_2 = 0.0887
Final R indexes [all data]	R_1 = 0.0742, wR_2 = 0.1037
Largest diff. peak/hole / e Å^{-3}	0.23/-0.21
Flack parameter	0.5(9)

Table S2. Fractional Atomic Coordinates (×10^4) and Equivalent Isotropic Displacement Parameters (Å^2×10^3) for (*S*)-L1. U_{eq} is defined as 1/3 of the trace of the orthogonalised U_{IJ} tensor. Related to STAR methods.

Atom	*x*	*y*	*z*	U(eq)
O001	3315(2)	2907(2)	5177.6(9)	32.7(5)
O002	357(3)	608(2)	3757.1(11)	39.5(5)
C003	1958(3)	3933(3)	4160.3(12)	24.1(5)
C004	4751(4)	2869(3)	3577.0(12)	27.7(6)
C005	2965(3)	2561(3)	3776.0(12)	25.2(5)
C006	2175(3)	4048(3)	4840.8(13)	24.5(5)
C007	2123(4)	959(4)	3601.8(13)	29.3(6)
C008	1271(3)	5309(3)	5215.9(14)	28.3(6)
C009	788(3)	5140(3)	3844.8(13)	26.1(6)
C00A	-112(4)	6407(3)	4223.2(14)	29.9(6)
C00B	5657(4)	1572(4)	3217.0(13)	30.9(6)
C00C	118(4)	6484(3)	4899.0(14)	31.0(6)
C00D	4817(4)	-11(4)	3052.9(13)	34.1(6)
C00E	528(4)	5058(4)	3105.0(13)	34.8(6)
C00F	5666(4)	4597(4)	3744.3(16)	36.3(7)
C00G	3042(4)	-323(4)	3244.3(14)	34.6(6)

Table S3. Anisotropic Displacement Parameters (Å^2×10^3) for (*S*)-L1. The Anisotropic displacement factor exponent takes the form: -2π^2[h^{2}a*^{2}U$_{11}$+2hka*b*U$_{12}$+…]. Related to STAR methods.

Atom	U_{11}	U_{22}	U_{33}	U_{23}	U_{13}	U_{12}
O001	33.1(11)	34.7(10)	30.3(11)	-1.5(8)	-3.6(9)	9.4(9)
O002	31.7(10)	32.9(10)	53.8(14)	-10.4(9)	10.5(10)	-9.7(9)
C003	20.3(11)	22.5(12)	29.5(14)	-0.9(10)	1.9(10)	-3.3(11)

C004	27.0(13)	31.5(12)	24.6(13)	0.3(11)	0.6(11)	-2.3(12)
C005	24.9(12)	27.2(13)	23.5(13)	0.4(10)	-0.8(11)	1.5(11)
C006	21.5(12)	23.1(11)	28.9(14)	0.6(11)	-2.2(11)	-0.2(10)
C007	27.1(13)	30.0(13)	30.9(15)	-0.9(11)	2.9(11)	-1.4(12)
C008	29.0(13)	27.7(12)	28.2(14)	-3.5(11)	1.0(11)	-1.9(12)
C009	22.6(12)	24.0(12)	31.7(15)	3.5(11)	1.0(10)	-3.1(11)
C00A	26.5(13)	23.5(12)	39.7(16)	2.3(11)	-2.1(12)	0.4(11)
C00B	27.3(14)	38.7(14)	26.8(15)	0.0(11)	3.3(11)	4.3(12)
C00C	29.3(13)	24.0(12)	39.7(17)	-5.7(11)	2.0(13)	1.0(11)
C00D	40.0(16)	36.4(15)	25.8(14)	-4.6(12)	2.2(12)	7.3(14)
C00E	34.4(14)	38.2(15)	31.7(15)	5.6(12)	-2.3(12)	1.5(13)
C00F	26.2(13)	38.2(14)	44.5(17)	-5.2(13)	4.7(12)	-5.3(13)
C00G	39.5(16)	28.8(13)	35.6(16)	-6.6(12)	0.6(13)	-0.6(13)

Table S4. Bond Lengths for (*S*)-L1. Related to STAR methods.

Atom	Atom	Length/Å	Atom	Atom	Length/Å
O001	C006	1.383(3)	C006	C008	1.387(3)
O002	C007	1.370(3)	C007	C00G	1.385(4)
C003	C005	1.492(3)	C008	C00C	1.386(4)
C003	C006	1.390(3)	C009	C00A	1.392(4)
C003	C009	1.408(3)	C009	C00E	1.512(4)
C004	C005	1.402(4)	C00A	C00C	1.381(4)
C004	C00B	1.390(4)	C00B	C00D	1.383(4)
C004	C00F	1.504(4)	C00D	C00G	1.390(4)
C005	C007	1.402(4)			

Table S5. Bond Angles for (*S*)-L1. Related to STAR methods.

Atom	Atom	Atom	Angle/°	Atom	Atom	Atom	Angle/°
C006	C003	C005	120.2(2)	O002	C007	C005	122.1(2)
C006	C003	C009	118.8(2)	O002	C007	C00G	117.1(2)
C009	C003	C005	121.1(2)	C00G	C007	C005	120.7(2)
C005	C004	C00F	120.2(2)	C00C	C008	C006	118.6(2)
C00B	C004	C005	119.4(2)	C003	C009	C00E	120.1(2)
C00B	C004	C00F	120.5(2)	C00A	C009	C003	119.0(2)

C004	C005	C003	120.5(2)	C00A	C009	C00E	120.8(2)
C007	C005	C003	120.2(2)	C00C	C00A	C009	121.0(2)
C007	C005	C004	119.3(2)	C00D	C00B	C004	120.9(2)
O001	C006	C003	121.4(2)	C00A	C00C	C008	120.6(2)
O001	C006	C008	116.6(2)	C00B	C00D	C00G	120.2(3)
C008	C006	C003	122.0(2)	C007	C00G	C00D	119.6(3)

Table S6. Torsion Angles for (*S*)-L1. Related to STAR methods.

A	B	C	D	Angle/°	A	B	C	D	Angle/°
O001	C006	C008	C00C	179.8(2)	C006	C003	C005	C007	97.8(3)
O002	C007	C00G	C00D	177.7(3)	C006	C003	C009	C00A	0.0(3)
C003	C005	C007	O002	1.8(4)	C006	C003	C009	C00E	179.7(2)
C003	C005	C007	C00G	179.9(2)	C006	C008	C00C	C00A	-0.5(4)
C003	C006	C008	C00C	0.0(4)	C009	C003	C005	C004	96.4(3)
C003	C009	C00A	C00C	-0.5(4)	C009	C003	C005	C007	-82.7(3)
C004	C005	C007	O002	-177.2(2)	C009	C003	C006	O001	-179.5(2)
C004	C005	C007	C00G	0.8(4)	C009	C003	C006	C008	0.2(4)
C004	C00B	C00D	C00G	0.3(4)	C009	C00A	C00C	C008	0.7(4)
C005	C003	C006	O001	0.0(3)	C00B	C004	C005	C003	-179.7(2)
C005	C003	C006	C008	179.7(2)	C00B	C004	C005	C007	-0.7(4)
C005	C003	C009	C00A	-179.5(2)	C00B	C00D	C00G	C007	-0.2(4)
C005	C003	C009	C00E	0.2(3)	C00E	C009	C00A	C00C	179.9(2)
C005	C004	C00B	C00D	0.1(4)	C00F	C004	C005	C003	-0.6(4)
C005	C007	C00G	C00D	-0.4(4)	C00F	C004	C005	C007	178.5(2)
C006	C003	C005	C004	-83.1(3)	C00F	C004	C00B	C00D	-179.1(2)

Table S7. Hydrogen Atom Coordinates (Å×10^4) and Isotropic Displacement Parameters (Å^2×10^3) for (*S*)-L1. Related to STAR methods.

**Atom**	***x***	***y***	***z***	**U(eq)**
H001	3762.9	2171.17	4912	49
H002	10.2	1316.1	4051.88	59
H008	1437.9	5365.47	5680.46	34
H00A	-896.84	7231.22	4013.11	36
H00B	6867.76	1775.63	3081.86	37

H00C	-518.75	7349.84	5148.88	37
H00D	5455.43	-887.65	2808.69	41
H00E	1702.72	5166.61	2885.51	52
H00F	-259.22	6034.56	2964.24	52
H00G	-27.09	3919.05	2985.74	52
H00H	4924.46	5590.81	3587.37	54
H00I	6852.74	4641.14	3530.57	54
H00J	5816.78	4685.94	4223.79	54
H00K	2464.31	-1408.77	3130.55	42

X-Ray crystallographic data for (*S*)-L3

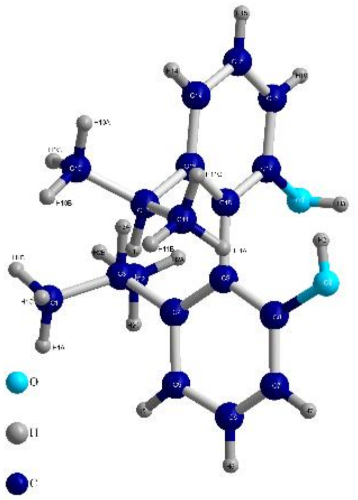

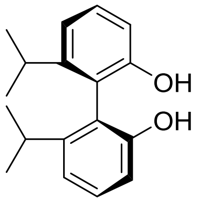

(*S*)-**L3**

Table S8. Crystal data and structure refinement for (*S*)-L3 (CCDC2209954).

Related to STAR methods.

Identification code	**(*S*)-L3**
Empirical formula	$C_{18}H_{22}O_2$
Formula weight	270.35
Temperature/K	202.00
Crystal system	orthorhombic
Space group	$P2_12_12_1$
a/Å	9.3963(12)
b/Å	9.7309(13)
c/Å	17.463(3)
α/°	90
β/°	90
γ/°	90
Volume/Å^3	1596.7(4)
Z	4
ρ_{calc}g/cm^3	1.125
μ/mm^{-1}	0.072
F(000)	584.0
Crystal size/mm^3	0.22 × 0.16 × 0.13
Radiation	MoKα (λ = 0.71073)
2Θ range for data collection/°	4.666 to 56.544
Index ranges	-10 ≤ h ≤ 12, -11 ≤ k ≤ 12, -23 ≤ l ≤ 23
Reflections collected	13925

Independent reflections	3893 [R_{int} = 0.0470, R_{sigma} = 0.0408]
Data/restraints/parameters	3893/33/177
Goodness-of-fit on F^2	1.095
Final R indexes [I>=2σ (I)]	R_1 = 0.1008, wR_2 = 0.2835
Final R indexes [all data]	R_1 = 0.1522, wR_2 = 0.3293
Largest diff. peak/hole / e $Å^{-3}$	0.53/-0.33
Flack parameter	-3(7)

Table S9. Fractional Atomic Coordinates (×10^4) and Equivalent Isotropic Displacement Parameters ($Å^2$×10^3) for (*S*)-L3. U_{eq} is defined as 1/3 of of the trace of the orthogonalised U_{IJ} tensor. Related to STAR methods.

**Atom**	***x***	***y***	***z***	**U(eq)**
O2	7346(6)	-5170(9)	-2250(4)	116(2)
O3	10585(7)	-7171(7)	-2512(3)	107.1(19)
C13	9386(3)	-4619(4)	-3906(2)	58.6(13)
C18	9355(3)	-5749(4)	-3420(2)	60.3(14)
C17	10538(4)	-6065(4)	-2975(2)	78.0(19)
C16	11751(3)	-5251(5)	-3017(2)	88(2)
C15	11782(4)	-4121(5)	-3503(3)	81(2)
C14	10599(4)	-3805(3)	-3948(2)	73.2(16)
C9	8046(6)	-6613(7)	-3326(3)	66.0(16)
C12	8093(7)	-4256(7)	-4392(3)	67.6(15)
C4	7759(6)	-7704(8)	-3796(5)	83(2)
C8	7095(7)	-6260(9)	-2740(5)	88(2)
C3	8767(9)	-8143(9)	-4422(5)	91(2)
C7	5847(10)	-6979(9)	-2634(5)	97(2)
C10	8468(10)	-3847(9)	-5203(5)	103(3)
C5	6496(10)	-8479(9)	-3666(6)	104(3)
C6	5587(12)	-7995(10)	-3059(5)	107(3)
C11	7157(10)	-3200(11)	-4014(6)	115(3)
C1	8041(18)	-8279(16)	-5201(7)	172(6)
C2	9595(18)	-9448(14)	-4208(11)	214(8)

Table S10. Anisotropic Displacement Parameters (Å^2×10^3) for (*S*)-L3. The Anisotropic displacement factor exponent takes the form: -2π^2[h^{2}a*^{2}U$_{11}$+2hka*b*U$_{12}$+…]. Related to STAR methods.

Atom	U11	U22	U33	U23	U13	U12
O2	76(4)	183(6)	88(3)	4(4)	20(3)	26(4)
O3	98(3)	120(3)	103(3)	40(2)	-4(2)	6(2)
C13	56(3)	63(3)	56(3)	-9(2)	-1(2)	-2(3)
C18	44(2)	86(4)	51(3)	6(3)	2(2)	3(3)
C17	52(3)	122(5)	61(3)	21(4)	2(3)	2(4)
C16	51(3)	152(7)	62(3)	-3(4)	-6(3)	-1(4)
C15	62(4)	102(5)	79(4)	-24(4)	3(3)	-20(4)
C14	65(4)	69(3)	86(4)	-9(3)	-1(3)	-8(3)
C9	43(3)	90(4)	65(3)	24(3)	2(2)	4(3)
C12	61(3)	68(3)	74(3)	2(3)	-10(3)	-1(3)
C4	50(3)	86(4)	114(6)	38(4)	-1(4)	-7(3)
C8	41(3)	131(6)	92(5)	40(5)	11(3)	14(4)
C3	91(2)	90(2)	93(2)	-0.4(13)	0.6(13)	-1.4(13)
C7	95(3)	99(3)	98(3)	4.5(13)	1.5(13)	2.0(13)
C10	99(6)	118(6)	91(5)	29(5)	-10(4)	6(5)
C5	103(3)	103(3)	106(3)	2.3(13)	-0.7(13)	-1.8(13)
C6	104(3)	108(3)	109(3)	4.5(13)	0.2(13)	-0.2(13)
C11	99(6)	136(7)	110(6)	3(6)	-15(5)	46(6)
C1	160(11)	222(14)	134(9)	-73(10)	-17(8)	-25(11)
C2	204(15)	149(11)	290(20)	48(12)	96(15)	89(11)

Table S11. Bond Lengths for (*S*)-L3. Related to STAR methods.

Atom	Atom	Length/Å	Atom	Atom	Length/Å
O2	C8	1.383(11)	C9	C8	1.402(10)
O3	C17	1.346(6)	C12	C10	1.512(11)
C13	C18	1.3900	C12	C11	1.506(11)
C13	C14	1.3900	C4	C3	1.509(11)
C13	C12	1.524(6)	C4	C5	1.424(11)
C18	C17	1.3900	C8	C7	1.378(11)
C18	C9	1.499(7)	C3	C1	1.527(15)
C17	C16	1.3900	C3	C2	1.534(15)
C16	C15	1.3900	C7	C6	1.260(12)

C15	C14	1.3900	C5	C6	1.440(13)
C9	C4	1.368(10)			

Table S12. Bond Angles for (*S*)-L3. Related to STAR methods.

**Atom**	**Atom**	**Atom**	**Angle/°**	**Atom**	**Atom**	**Atom**	**Angle/°**
C18	C13	C14	120.0	C10	C12	C13	113.4(6)
C18	C13	C12	120.5(3)	C11	C12	C13	112.3(5)
C14	C13	C12	119.5(3)	C11	C12	C10	111.6(7)
C13	C18	C17	120.0	C9	C4	C3	122.0(6)
C13	C18	C9	121.8(3)	C9	C4	C5	118.6(8)
C17	C18	C9	118.1(3)	C5	C4	C3	119.3(8)
O3	C17	C18	122.6(4)	O2	C8	C9	122.0(6)
O3	C17	C16	117.4(4)	C7	C8	O2	116.9(7)
C16	C17	C18	120.0	C7	C8	C9	121.0(9)
C15	C16	C17	120.0	C4	C3	C1	112.9(9)
C16	C15	C14	120.0	C4	C3	C2	112.1(9)
C15	C14	C13	120.0	C1	C3	C2	111.8(11)
C4	C9	C18	122.1(5)	C6	C7	C8	119.0(10)
C4	C9	C8	120.1(7)	C4	C5	C6	116.0(9)
C8	C9	C18	117.7(6)	C7	C6	C5	125.1(11)

Table S13. Torsion Angles for (*S*)-L3. Related to STAR methods.

**A**	**B**	**C**	**D**	**Angle/°**	**A**	**B**	**C**	**D**	**Angle/°**
O2	C8	C7	C6	-178.8(8)	C14	C13	C12	C10	42.9(7)
O3	C17	C16	C15	177.9(5)	C14	C13	C12	C11	-84.8(7)
C13	C18	C17	O3	-177.8(5)	C9	C18	C17	O3	5.2(6)
C13	C18	C17	C16	0.0	C9	C18	C17	C16	-177.0(4)
C13	C18	C9	C4	87.7(6)	C9	C4	C3	C1	-128.1(9)
C13	C18	C9	C8	-91.6(5)	C9	C4	C3	C2	104.5(11)
C18	C13	C14	C15	0.0	C9	C4	C5	C6	2.6(11)
C18	C13	C12	C10	-137.6(5)	C9	C8	C7	C6	2.3(12)
C18	C13	C12	C11	94.8(7)	C12	C13	C18	C17	-179.5(4)
C18	C17	C16	C15	0.0	C12	C13	C18	C9	-2.7(5)
C18	C9	C4	C3	2.3(9)	C12	C13	C14	C15	179.5(4)

C18	C9	C4	C5	179.7(6)	C4	C9	C8	O2	179.7(6)
C18	C9	C8	O2	-1.0(9)	C4	C9	C8	C7	-1.4(10)
C18	C9	C8	C7	177.9(6)	C4	C5	C6	C7	-1.8(13)
C17	C18	C9	C4	-95.4(6)	C8	C9	C4	C3	-178.5(6)
C17	C18	C9	C8	85.3(6)	C8	C9	C4	C5	-1.1(9)
C17	C16	C15	C14	0.0	C8	C7	C6	C5	-0.6(14)
C16	C15	C14	C13	0.0	C3	C4	C5	C6	-179.9(7)
C14	C13	C18	C17	0.0	C5	C4	C3	C1	54.5(11)
C14	C13	C18	C9	176.8(4)	C5	C4	C3	C2	-72.9(12)

Table S14. Hydrogen Atom Coordinates (Å×10^4) and Isotropic Displacement Parameters (Å^2×10^3) for (S)-L3. Related to STAR methods.

**Atom**	***x***	***y***	***z***	**U(eq)**
H2	8121.73	-4794.13	-2365.95	173
H3	10461.9	-6921.86	-2056.46	161
H16	12558.8	-5467.51	-2712.54	106
H15	12610.87	-3565.12	-3531.68	97
H14	10620.61	-3032.95	-4280.53	88
H12	7511.58	-5113.42	-4432.42	81
H3A	9487.41	-7392.48	-4474.44	109
H7	5192.19	-6712.56	-2247.5	116
H10A	9014.29	-2989.64	-5195.31	154
H10B	7592.63	-3713.21	-5498.79	154
H10C	9039.48	-4574.38	-5439.91	154
H5	6267.14	-9267.08	-3962.13	124
H6	4724.66	-8479.6	-2972.68	129
H11A	6825.36	-3553.21	-3519.24	172
H11B	6336.22	-3006.42	-4342.47	172
H11C	7700.48	-2352.7	-3932.26	172
H1A	7373.45	-9052.47	-5188.44	258
H1B	8761.16	-8441.55	-5596.35	258
H1C	7522.71	-7430.41	-5316.52	258
H2A	10128.18	-9288.11	-3734.5	321
H2B	10257.68	-9679.72	-4621.82	321
H2C	8926.11	-10208.74	-4132.36	321

X-Ray crystallographic data for (*S*)-L5.

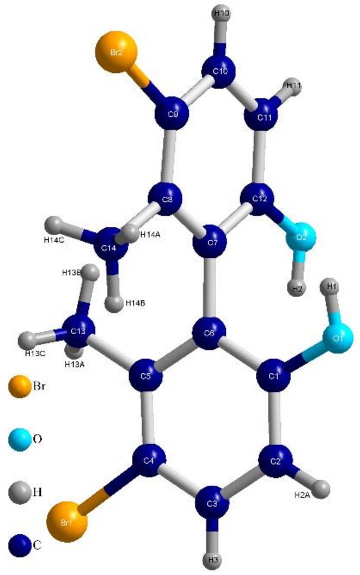

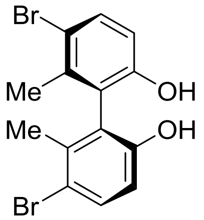

(*S*)-**L5**

Table S15. Crystal data and structure refinement for (*S*)-L5 (CCDC 2209951).
Related to STAR methods.

Identification code	**(*S*)-L5**
Empirical formula	$C_{14}H_{12}Br_2O_2$
Formula weight	372.06
Temperature/K	170.0
Crystal system	orthorhombic
Space group	$P2_12_12_1$
a/Å	7.4976(2)
b/Å	15.9674(4)
c/Å	22.3329(7)
α/°	90
β/°	90
γ/°	90
Volume/Å^3	2673.63(13)
Z	8
ρ_{calc}g/cm^3	1.849
μ/mm^{-1}	6.054
F(000)	1456.0
Crystal size/mm^3	0.48 × 0.13 × 0.1
Radiation	MoKα (λ = 0.71073)
2Θ range for data collection/°	4.452 to 54.58
Index ranges	$-9 \le h \le 9$, $-20 \le k \le 20$, $-28 \le l \le 28$
Reflections collected	39035

Independent reflections	5975 [R_{int} = 0.0406, R_{sigma} = 0.0345]
Data/restraints/parameters	5975/1/335
Goodness-of-fit on F^2	1.024
Final R indexes [I>=2σ (I)]	R_1 = 0.0201, wR_2 = 0.0432
Final R indexes [all data]	R_1 = 0.0244, wR_2 = 0.0447
Largest diff. peak/hole / e Å^{-3}	0.25/-0.34
Flack parameter	0.008(4)

Table S16. Fractional Atomic Coordinates (×10^4) and Equivalent Isotropic Displacement Parameters (Å^2 ×10^3) for (S)-L5. U_{eq} is defined as 1/3 of of the trace of the orthogonalised U_{IJ} tensor. Related to STAR methods.

**Atom**	***x***	***y***	***z***	**U(eq)**
Br1	13141.7(5)	3926.7(2)	1010.0(2)	37.62(9)
Br2	4350.2(4)	7347.3(2)	1569.5(2)	33.73(9)
O1	6108(3)	3828.2(13)	2300.1(11)	31.9(5)
O2	8818(3)	5148.6(13)	3170.7(9)	23.7(5)
C1	7706(3)	3903.2(19)	2015.1(13)	21.5(6)
C2	8449(4)	3165.4(18)	1799.3(13)	25.0(6)
C3	10045(4)	3190.7(19)	1490.8(13)	24.5(6)
C4	10908(4)	3948.1(19)	1417.2(12)	22.1(6)
C5	10230(4)	4699.7(18)	1638.4(12)	20.1(6)
C6	8575(4)	4670.3(17)	1938.5(12)	17.6(6)
C7	7708(3)	5449.9(17)	2174.7(12)	17.3(6)
C8	6687(4)	5972.1(17)	1798.4(12)	20.3(6)
C9	5833(4)	6659.2(18)	2052.5(13)	22.2(6)
C10	6006(4)	6855.7(18)	2658.2(13)	23.1(6)
C11	7022(4)	6345.5(17)	3023.0(13)	21.4(6)
C12	7851(3)	5641.7(17)	2783.1(12)	17.6(6)
C13	11213(4)	5515.6(19)	1568.2(15)	28.6(7)
C14	6541(4)	5786(2)	1139.1(12)	28.8(7)

Table S17. Anisotropic Displacement Parameters (Å^2 ×10^3) for (*S*)-L5. The Anisotropic displacement factor exponent takes the form: -2π^2[h^{2}a*^{2}U$_{11}$+2hka*b*U$_{12}$+…]. Related to STAR methods.

**Atom**	**U_{11}**	**U_{22}**	**U_{33}**	**U_{23}**	**U_{13}**	**U_{12}**

Br1	34.10(17)	35.31(19)	43.5(2)	-1.01(16)	20.36(15)	5.26(16)
Br2	33.05(16)	23.04(16)	45.1(2)	3.96(14)	-14.33(14)	5.55(14)
O1	27.9(11)	18.8(11)	48.9(14)	-3.2(11)	16.5(10)	-4.3(9)
O2	27.7(11)	22.2(11)	21.2(11)	-1.8(8)	-3.8(8)	5.4(9)
C1	19.2(13)	22.9(15)	22.3(15)	0.7(13)	0.0(11)	-2.2(12)
C2	28.9(15)	15.6(14)	30.5(17)	-2.5(12)	0.9(13)	-3.7(13)
C3	30.7(15)	20.7(15)	22.2(15)	-4.8(12)	0.1(12)	4.4(13)
C4	23.4(14)	24.9(15)	17.9(14)	-0.3(12)	3.5(11)	4.5(13)
C5	22.2(14)	19.4(14)	18.8(14)	2.9(11)	0.4(11)	0.6(11)
C6	20.6(13)	16.4(13)	16.0(14)	-0.3(11)	-1.8(11)	1.2(11)
C7	15.6(12)	14.6(13)	21.9(15)	-1.4(11)	2.0(10)	-2.9(11)
C8	18.4(12)	18.8(14)	23.6(15)	0.7(11)	-1.5(11)	-1.6(12)
C9	18.0(13)	17.5(14)	31.2(17)	1.7(12)	-4.2(12)	-0.6(12)
C10	21.5(14)	16.5(14)	31.2(17)	-3.2(12)	2.8(12)	1.2(11)
C11	23.0(14)	18.4(14)	22.9(15)	-3.1(12)	0.6(12)	-1.3(12)
C12	15.6(12)	17.1(14)	20.0(14)	1.9(11)	-0.3(10)	-1.8(11)
C13	27.5(15)	21.6(15)	36.9(19)	0.7(14)	9.4(13)	-4.7(13)
C14	37.9(17)	27.5(16)	21.0(16)	-0.1(12)	-5.8(13)	-1.1(14)

Table S18. Bond Lengths for (*S*)-L5. Related to STAR methods.

**Atom**	**Atom**	**Length/Å**	**Atom**	**Atom**	**Length/Å**
Br1	C4	1.906(3)	C5	C6	1.411(4)
Br2	C9	1.899(3)	C5	C13	1.505(4)
O1	C1	1.362(3)	C6	C7	1.500(4)
O2	C12	1.377(3)	C7	C8	1.410(4)
C1	C2	1.389(4)	C7	C12	1.397(4)
C1	C6	1.398(4)	C8	C9	1.391(4)
C2	C3	1.382(4)	C8	C14	1.506(4)
C3	C4	1.381(4)	C9	C10	1.395(4)
C4	C5	1.394(4)	C10	C11	1.381(4)
			C11	C12	1.391(4)

Table S19. Bond Angles for (*S*)-L5. Related to STAR methods.

Atom	Atom	Atom	Angle/°	Atom	Atom	Atom	Angle/°
O1	C1	C2	116.1(3)	C8	C7	C6	121.1(2)
O1	C1	C6	123.0(3)	C12	C7	C6	119.4(2)
C2	C1	C6	120.9(2)	C12	C7	C8	119.4(3)
C3	C2	C1	119.7(3)	C7	C8	C14	120.3(3)
C4	C3	C2	119.4(3)	C9	C8	C7	118.2(3)
C3	C4	Br1	116.9(2)	C9	C8	C14	121.4(3)
C3	C4	C5	122.7(2)	C8	C9	Br2	119.6(2)
C5	C4	Br1	120.3(2)	C8	C9	C10	122.0(3)
C4	C5	C6	117.4(3)	C10	C9	Br2	118.4(2)
C4	C5	C13	122.0(3)	C11	C10	C9	119.4(3)
C6	C5	C13	120.6(3)	C10	C11	C12	119.7(3)
C1	C6	C5	119.8(3)	O2	C12	C7	121.8(2)
C1	C6	C7	118.8(2)	O2	C12	C11	117.1(2)
C5	C6	C7	121.4(2)	C11	C12	C7	121.2(3)

Table S20. Torsion Angles for (S)-L5. Related to STAR methods.

A	B	C	D	Angle/°	A	B	C	D	Angle/°
Br1	C4	C5	C6	-179.62(19)	C6	C1	C2	C3	-1.7(4)
Br1	C4	C5	C13	-0.1(4)	C6	C7	C8	C9	176.3(2)
Br2	C9	C10	C11	177.5(2)	C6	C7	C8	C14	-3.9(4)
O1	C1	C2	C3	178.3(3)	C6	C7	C12	O2	2.2(4)
O1	C1	C6	C5	180.0(3)	C6	C7	C12	C11	-178.1(2)
O1	C1	C6	C7	-0.6(4)	C7	C8	C9	Br2	-176.9(2)
C1	C2	C3	C4	2.0(4)	C7	C8	C9	C10	2.0(4)
C1	C6	C7	C8	-96.8(3)	C8	C7	C12	O2	179.4(2)
C1	C6	C7	C12	80.4(3)	C8	C7	C12	C11	-0.9(4)
C2	C1	C6	C5	0.0(4)	C8	C9	C10	C11	-1.4(4)
C2	C1	C6	C7	179.4(3)	C9	C10	C11	C12	-0.3(4)
C2	C3	C4	Br1	178.0(2)	C10	C11	C12	O2	-178.8(3)
C2	C3	C4	C5	-0.5(4)	C10	C11	C12	C7	1.5(4)
C3	C4	C5	C6	-1.2(4)	C12	C7	C8	C9	-0.8(4)
C3	C4	C5	C13	178.3(3)	C12	C7	C8	C14	178.9(3)
C4	C5	C6	C1	1.4(4)	C13	C5	C6	C1	-178.1(3)
C4	C5	C6	C7	-178.0(3)	C13	C5	C6	C7	2.5(4)

C5	C6	C7	C8	82.6(3)	C14	C8	C9	Br2	3.3(4)
C5	C6	C7	C12	-100.2(3)	C14	C8	C9	C10	-177.8(3)

Table S21. Hydrogen Atom Coordinates (Å×10^4) and Isotropic Displacement Parameters (Å^2×10^3) for (*S*)-L5. Related to STAR methods.

**Atom**	***x***	***y***	***z***	**U(eq)**
H1	5714.78	4306.52	2384.53	48
H2	9218.35	4730.85	2985.81	36
H2A	7861.59	2645.86	1863.58	30
H3	10544.9	2691.8	1330.8	29
H10	5429.87	7335.97	2818.34	28
H11	7155.13	6474.54	3435.81	26
H3A	4150(40)	5343(18)	3048(14)	32
H13A	12428.25	5455.49	1724.3	43
H13B	10589.09	5955.45	1791.21	43
H13C	11263.18	5667.22	1143.28	43
H14A	5287.86	5691.25	1034.32	43
H14B	7237.1	5283.13	1044.77	43
H14C	7003.65	6261.16	909.21	43

X-Ray crystallographic data for (*S*)-L8

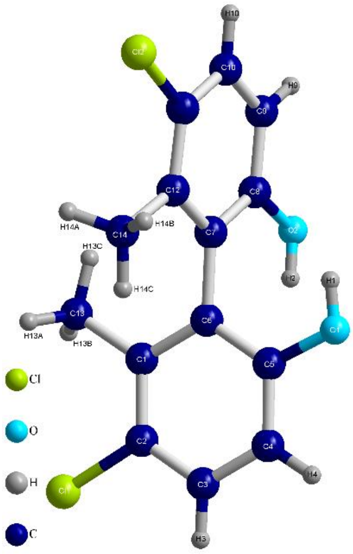

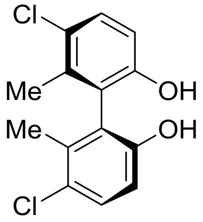

(*S*)-**L8**

Table S22. Crystal data and structure refinement for (*S*)-L8 (CCDC 2209952).
Related to STAR methods.

Identification code	**(*S*)-L8**
Empirical formula	$C_{14}H_{12}Cl_2O_2$
Formula weight	283.14
Temperature/K	170.0
Crystal system	orthorhombic
Space group	$P2_12_12_1$
a/Å	7.3584(3)
b/Å	15.7010(8)
c/Å	22.2426(12)
α/°	90
β/°	90
γ/°	90
Volume/Å^3	2569.8(2)
Z	8
ρ_{calc}g/cm^3	1.464
μ/mm^{-1}	0.495
F(000)	1168.0
Crystal size/mm^3	0.48 × 0.1 × 0.08
Radiation	MoKα (λ = 0.71073)
2Θ range for data collection/°	5.188 to 54.402
Index ranges	$-9 \leq h \leq 9$, $-20 \leq k \leq 20$, $-28 \leq l \leq 28$
Reflections collected	38785

Independent reflections	5733 [R_{int} = 0.0769, R_{sigma} = 0.0454]
Data/restraints/parameters	5733/1/335
Goodness-of-fit on F^2	1.028
Final R indexes [I>=2σ (I)]	R_1 = 0.0388, wR_2 = 0.0776
Final R indexes [all data]	R_1 = 0.0581, wR_2 = 0.0861
Largest diff. peak/hole / e Å^{-3}	0.23/-0.23
Flack parameter	-0.09(3)

Table S23. Fractional Atomic Coordinates (×10^4) and Equivalent Isotropic Displacement Parameters (Å^2×10^3) for (*S*)-L8. U_{eq} is defined as 1/3 of of the trace of the orthogonalised U_{IJ} tensor. Related to STAR methods.

Atom	*x*	*y*	*z*	U(eq)
Cl1	1862.9(13)	4032.9(6)	9007.6(5)	40.1(3)
Cl2	10546.6(12)	7377.7(6)	8394.0(4)	35.0(2)
O1	8838(3)	3840.5(15)	7740.6(13)	34.0(6)
O2	6158(3)	5167.1(15)	6837.8(11)	26.5(5)
C1	4647(5)	4770(2)	8385.7(15)	23.0(7)
C2	3940(5)	4017(2)	8623.8(15)	24.4(7)
C3	4807(5)	3241(2)	8566.8(16)	26.7(8)
C4	6429(5)	3194(2)	8255.7(16)	26.6(8)
C5	7203(4)	3935(2)	8026.0(15)	22.8(7)
C6	6332(4)	4720.2(19)	8086.4(14)	19.0(7)
C7	7248(4)	5499.8(19)	7838.0(15)	18.3(7)
C8	7136(4)	5680.9(19)	7224.7(15)	20.3(7)
C9	8006(4)	6376.8(19)	6977.9(15)	21.6(7)
C10	9040(4)	6901(2)	7341.9(16)	24.6(8)
C11	9186(5)	6720(2)	7949.2(16)	23.8(7)
C12	8286(4)	6032(2)	8212.7(14)	21.4(7)
C13	3652(5)	5602(2)	8438.3(18)	30.7(8)
C14	8411(5)	5874(2)	8878.0(15)	31.1(8)

Table S24. Anisotropic Displacement Parameters (Å^2×10^3) for (*S*)-L8. The Anisotropic displacement factor exponent takes the form: -$2\pi^2[h^2a^{*2}U_{11}+2hka^*b^*U_{12}+\ldots]$. Related to STAR methods.

Atom	U_{11}	U_{22}	U_{33}	U_{23}	U_{13}	U_{12}
Cl1	35.0(5)	38.8(5)	46.6(6)	0.6(5)	21.3(5)	-5.5(5)

Cl2	32.6(5)	25.5(4)	46.8(6)	-5.9(4)	-13.4(4)	-5.1(4)
O1	30.8(14)	21.6(13)	49.7(17)	4.3(12)	15.4(12)	8.6(11)
O2	29.8(13)	24.6(12)	25.2(13)	1.5(10)	-3.9(11)	-5.5(10)
C1	27.1(18)	20.6(16)	21.4(18)	-3.0(14)	0.1(15)	-0.7(14)
C2	25.8(18)	28.6(18)	18.8(18)	0.0(15)	4.4(13)	-4.8(15)
C3	32(2)	21.9(17)	26.6(19)	5.7(14)	-0.7(15)	-3.9(15)
C4	32(2)	17.2(16)	30(2)	3.0(14)	1.0(16)	2.3(15)
C5	21.8(17)	22.7(17)	24.0(18)	0.7(14)	2.9(14)	4.0(14)
C6	20.9(16)	18.0(15)	18.0(17)	0.5(13)	-0.5(13)	-0.6(13)
C7	15.7(15)	17.4(15)	21.9(17)	1.9(13)	0.7(13)	3.2(12)
C8	17.9(16)	18.9(16)	24.1(18)	-1.4(13)	-1.6(14)	1.7(13)
C9	20.6(17)	20.5(16)	23.7(18)	4.3(14)	1.3(14)	1.5(14)
C10	22.3(17)	16.9(16)	35(2)	2.6(15)	3.9(15)	-0.1(13)
C11	21.1(17)	18.2(16)	32(2)	-6.2(14)	-3.7(15)	2.2(13)
C12	20.9(16)	21.3(16)	21.8(17)	-0.8(14)	-1.8(13)	5.2(14)
C13	31(2)	24.4(18)	37(2)	-2.9(16)	9.0(17)	3.7(16)
C14	35(2)	32.1(19)	26.3(19)	-1.6(16)	-5.8(16)	-0.7(17)

Table S25. Bond Lengths for (*S*)-L8. Related to STAR methods.

Atom	Atom	Length/Å	Atom	Atom	Length/Å
Cl1	C2	1.751(3)	C5	C6	1.396(4)
Cl2	C11	1.746(3)	C6	C7	1.503(4)
O1	C5	1.368(4)	C7	C8	1.396(4)
O2	C8	1.382(4)	C7	C12	1.405(4)
C1	C2	1.395(5)	C8	C9	1.380(4)
C1	C6	1.410(5)	C9	C10	1.382(5)
C1	C13	1.503(4)	C10	C11	1.384(5)
C2	C3	1.382(5)	C11	C12	1.397(5)
C3	C4	1.381(5)	C12	C14	1.503(4)
C4	C5	1.393(5)			

Table S26. Bond Angles for (*S*)-L8. Related to STAR methods.

Atom	Atom	Atom	Angle/˚	Atom	Atom	Atom	Angle/˚
C2	C1	C6	117.4(3)	C8	C7	C6	119.9(3)

C2	C1	C13	121.7(3)	C8	C7	C12	119.4(3)
C6	C1	C13	120.9(3)	C12	C7	C6	120.6(3)
C1	C2	Cl1	119.9(3)	O2	C8	C7	121.4(3)
C3	C2	Cl1	117.4(3)	C9	C8	O2	117.1(3)
C3	C2	C1	122.7(3)	C9	C8	C7	121.5(3)
C4	C3	C2	119.5(3)	C8	C9	C10	119.6(3)
C3	C4	C5	119.5(3)	C9	C10	C11	119.5(3)
O1	C5	C4	116.1(3)	C10	C11	Cl2	118.4(3)
O1	C5	C6	122.9(3)	C10	C11	C12	122.1(3)
C4	C5	C6	121.0(3)	C12	C11	Cl2	119.5(3)
C1	C6	C7	121.6(3)	C7	C12	C14	121.3(3)
C5	C6	C1	119.8(3)	C11	C12	C7	117.9(3)
C5	C6	C7	118.6(3)	C11	C12	C14	120.7(3)

Table S27. Torsion Angles for (*S*)-L8. Related to STAR methods.

A	B	C	D	Angle/°	A	B	C	D	Angle/°
Cl1	C2	C3	C4	178.5(3)	C6	C1	C2	C3	-1.2(5)
Cl2	C11	C12	C7	-178.0(2)	C6	C7	C8	O2	1.6(5)
Cl2	C11	C12	C14	2.6(4)	C6	C7	C8	C9	-178.0(3)
O1	C5	C6	C1	179.9(3)	C6	C7	C12	C11	176.5(3)
O1	C5	C6	C7	-1.3(5)	C6	C7	C12	C14	-4.2(5)
O2	C8	C9	C10	-178.4(3)	C7	C8	C9	C10	1.1(5)
C1	C2	C3	C4	-0.9(5)	C8	C7	C12	C11	-0.8(5)
C1	C6	C7	C8	-100.7(4)	C8	C7	C12	C14	178.6(3)
C1	C6	C7	C12	82.0(4)	C8	C9	C10	C11	-0.1(5)
C2	C1	C6	C5	1.5(5)	C9	C10	C11	Cl2	178.4(2)
C2	C1	C6	C7	-177.3(3)	C9	C10	C11	C12	-1.5(5)
C2	C3	C4	C5	2.7(5)	C10	C11	C12	C7	1.9(5)
C3	C4	C5	O1	177.9(3)	C10	C11	C12	C14	-177.5(3)
C3	C4	C5	C6	-2.3(5)	C12	C7	C8	O2	178.9(3)
C4	C5	C6	C1	0.2(5)	C12	C7	C8	C9	-0.7(5)
C4	C5	C6	C7	179.0(3)	C13	C1	C2	Cl1	-1.2(5)
C5	C6	C7	C8	80.5(4)	C13	C1	C2	C3	178.2(3)
C5	C6	C7	C12	-96.8(4)	C13	C1	C6	C5	-177.9(3)
C6	C1	C2	Cl1	179.4(2)	C13	C1	C6	C7	3.3(5)
					C6	C1	C2	C3	-1.2(5)

Table S28. Hydrogen Atom Coordinates (Å×10^4) and Isotropic Displacement Parameters (Å^2×10^3) for (*S*)-L8. Related to STAR methods.

**Atom**	***x***	***y***	***z***	**U(eq)**
H1	9202.16	4317.48	7619.31	51
H2	5814.59	4728	7021.26	40
H3	4292.19	2743.33	8740.3	32
H4	7011.46	2659.61	8198.96	32
H9	7895.57	6494.89	6560.66	26
H10	9646.01	7381.08	7176.63	30
H13A	3594.07	5772.34	8861.76	46
H13B	2416.84	5537.06	8280.03	46
H13C	4296.17	6039.62	8207.45	46
H14A	7927.23	6367.18	9095.43	47
H14B	9685.27	5785.85	8990.5	47
H14C	7703.53	5366.16	8981.95	47

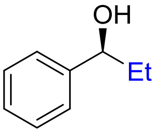

(*S*)-**23a**

HPLC: enantiomeric excess of **23a** (ee = 94%) was determined by high-performance liquid chromatography (HPLC) using a chiral stationary phase (OD-H column, flow rate = 1.0 mL/ min, eluent: hexane/ isopropanol = 96/4, 254 nm absorbance), retention times: minor enantiomer (t_R = 9.31 min), major enantiomer (t_R = 10.12 min).

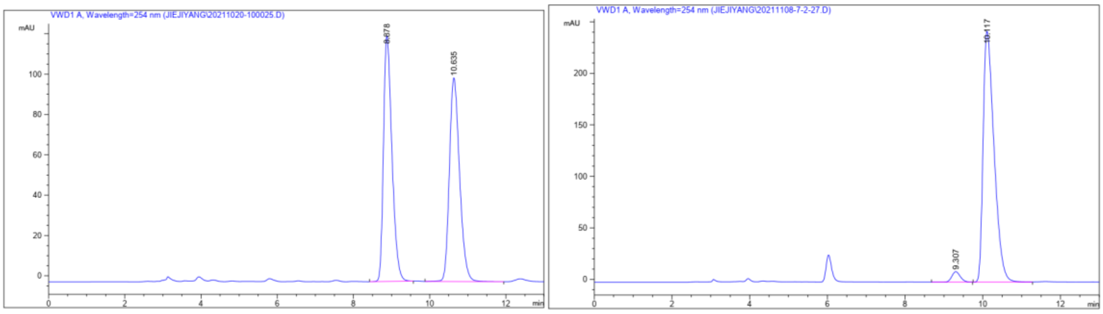

Figure S61. HPLC traces of 23a, Related to Figure 6

Signal 1: VWD1 A, Wavelength=254 nm

Peak #	RetTime [min]	Type	Width [min]	Area mAU *s	Height [mAU]	Area %
1	8.878	BB	0.2358	1866.36633	121.65910	49.9159
2	10.635	BB	0.2864	1872.65271	100.89375	50.0841
Totals :				3739.01904	222.55285	

Signal 1: VWD1 A, Wavelength=254 nm

Peak #	RetTime [min]	Type	Width [min]	Area mAU *s	Height [mAU]	Area %
1	9.307	BB	0.2412	153.24931	10.09301	3.1239
2	10.117	BV	0.3045	4752.51221	242.33174	96.8761
Totals :				4905.76152	252.42475	

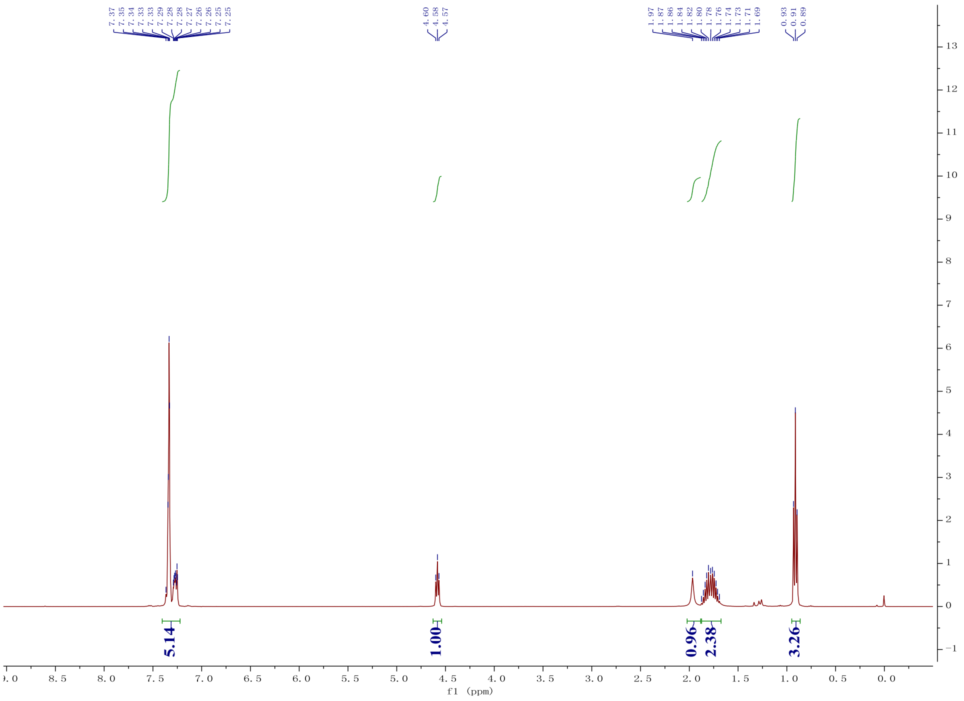

Figure S62. ^{1}H NMR spectrum of 23a, Related to Figure 6

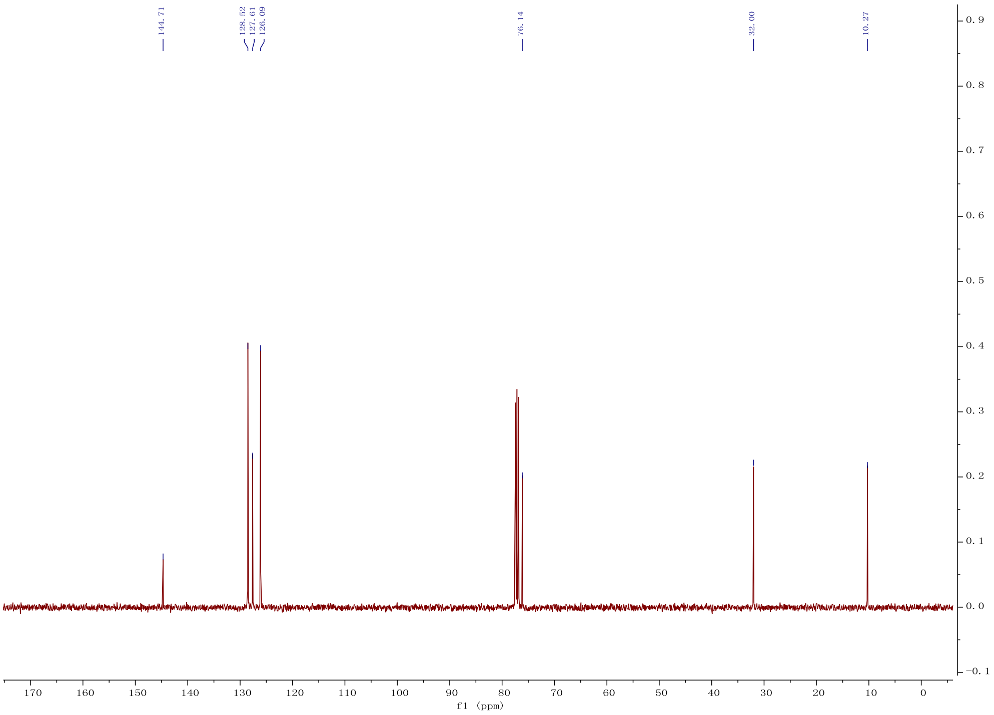

Figure S63. ^{13}C NMR spectrum of 23a, Related to Figure 6

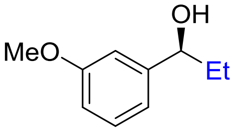

(*S*)-**23b**

HPLC: enantiomeric excess of **23b** (ee = 95%) was determined by high-performance liquid chromatography (HPLC) using a chiral stationary phase (IA column, flow rate = 1.0 mL/ min, eluent: hexane/ isopropanol = 98/2, 254 nm absorbance), retention times: minor enantiomer (t_R = 29.60 min), major enantiomer (t_R = 31.62 min).

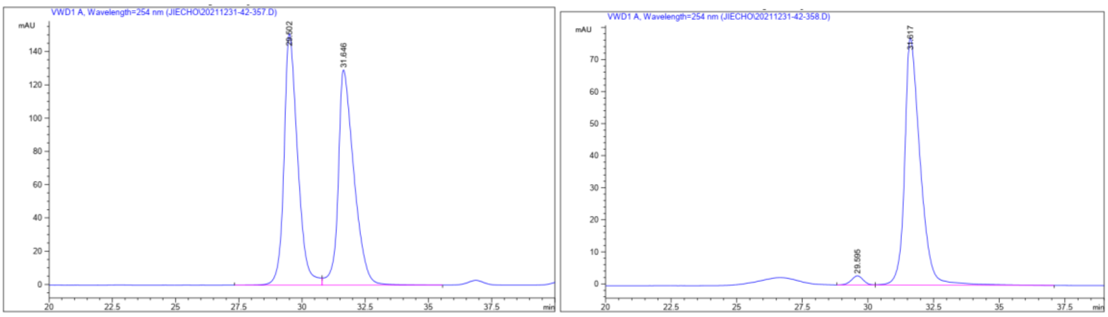

Figure S64. HPLC traces of 23b, Related to Figure 6

Signal 1: VWD1 A, Wavelength=254 nm

Peak #	RetTime [min]	Type	Width [min]	Area mAU *s	Height [mAU]	Area %
1	29.502	BV	0.5637	5682.47852	151.19569	50.3548
2	31.646	VB	0.6422	5602.40283	129.33150	49.6452
Totals :				1.12849e4	280.52719	

Signal 1: VWD1 A, Wavelength=254 nm

Peak #	RetTime [min]	Type	Width [min]	Area mAU *s	Height [mAU]	Area %
1	29.595	BV	0.4871	90.23805	2.85154	2.7595
2	31.617	VB	0.6103	3179.88525	77.16721	97.2405
Totals :				3270.12331	80.01875	

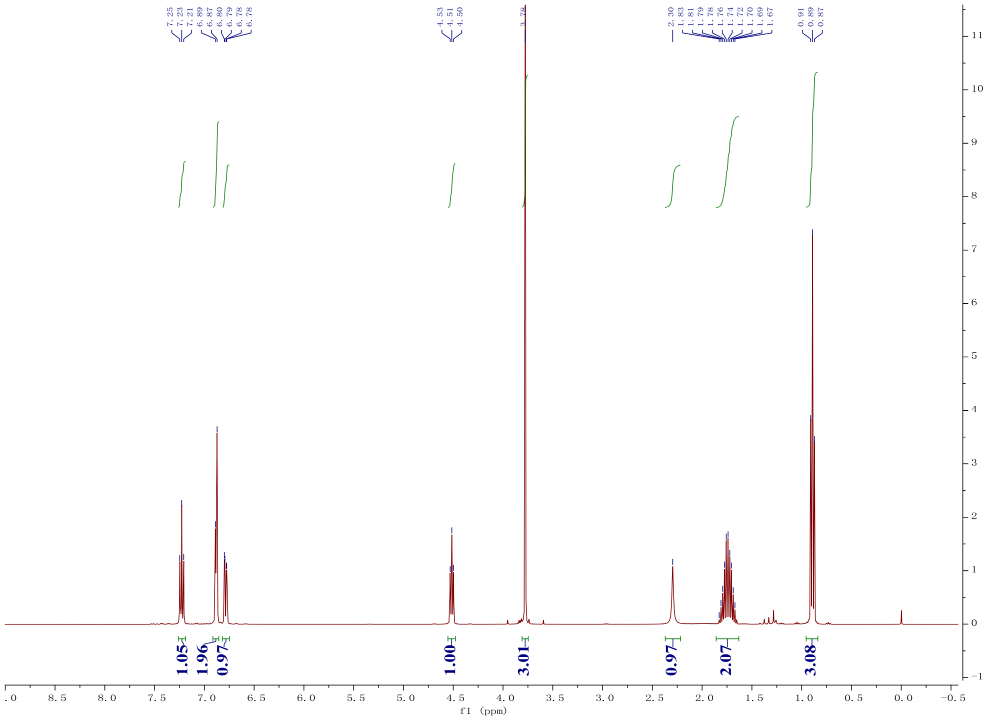

Figure S65. ^{1}H NMR spectrum of 23b, Related to Figure 6

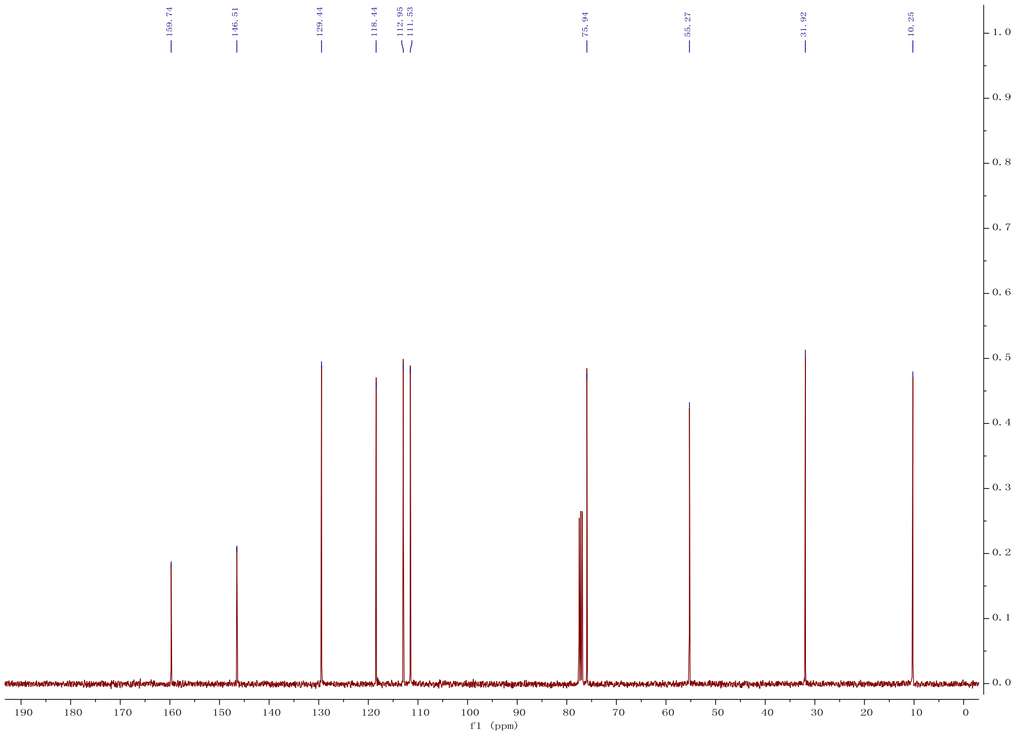

Figure S66. ^{13}C NMR spectrum of 23b, Related to Figure 6

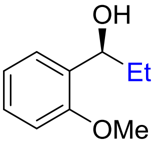

(*S*)-**23c**

HPLC: enantiomeric excess of **23c** (ee = 86%) was determined by high-performance liquid chromatography (HPLC) using a chiral stationary phase (OD-H column, flow rate = 1.0 mL/ min, eluent: hexane/ isopropanol = 96/4, 254 nm absorbance), retention times: minor enantiomer (t_R = 11.65 min), major enantiomer (t_R = 10.33 min).

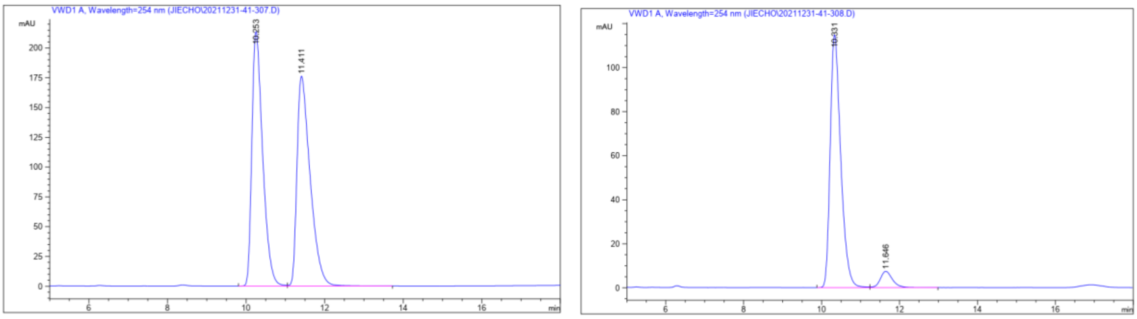

Figure S67. HPLC traces of 23c, Related to Figure 6

Signal 1: VWD1 A, Wavelength=254 nm

Peak #	RetTime [min]	Type	Width [min]	Area mAU *s	Height [mAU]	Area %
1	10.253	BV	0.2892	4064.65259	213.42894	49.7819
2	11.411	VB	0.3501	4100.27100	176.19601	50.2181
Totals :				8164.92358	389.62495	

Signal 1: VWD1 A, Wavelength=254 nm

Peak #	RetTime [min]	Type	Width [min]	Area mAU *s	Height [mAU]	Area %
1	10.331	BV	0.2873	2151.73853	114.69680	93.1483
2	11.646	VB	0.3258	158.27441	7.38256	6.8517
Totals :				2310.01294	122.07936	

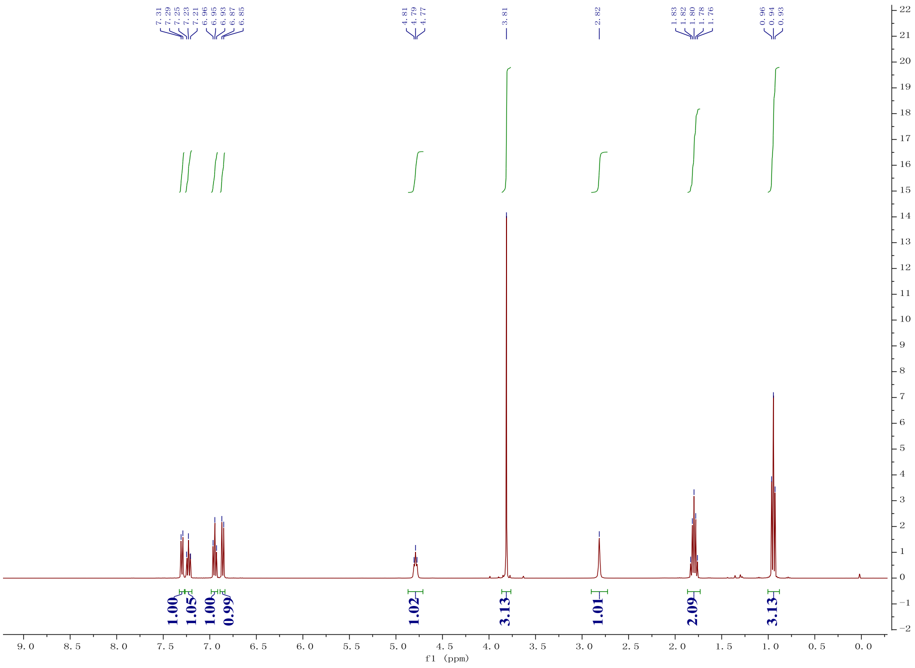

Figure S68. ^{1}H NMR spectrum of 23c, Related to Figure 6

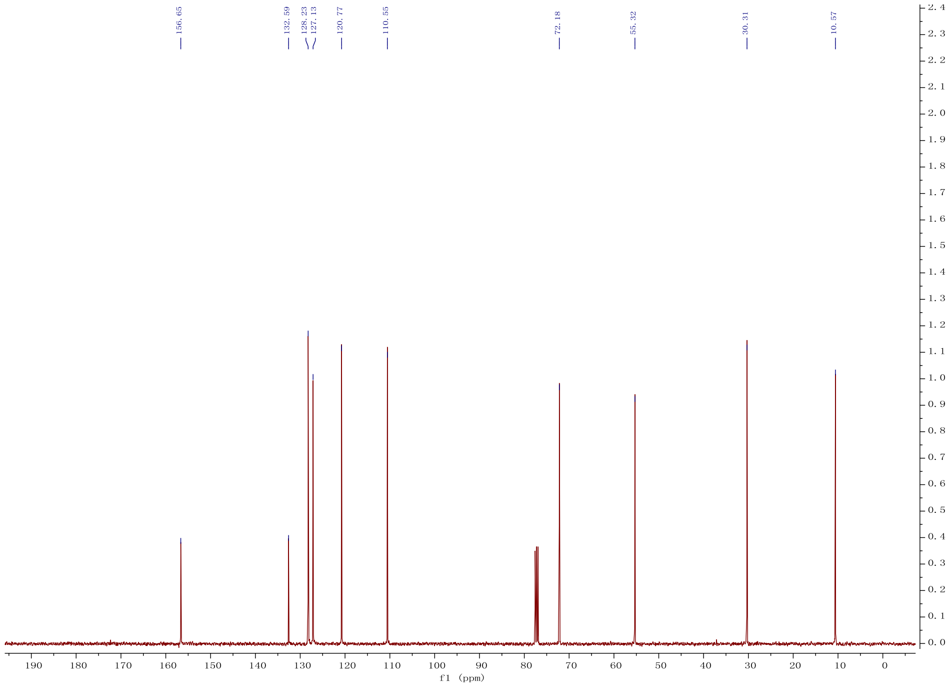

Figure S69. ^{13}C NMR spectrum of 23c, Related to Figure 6

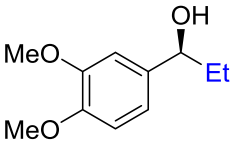

(*S*)-**23d**

HPLC: enantiomeric excess of **23d** (ee = 80%) was determined by high-performance liquid chromatography (HPLC) using a chiral stationary phase (AY-H column, flow rate = 1.0 mL/ min, eluent: hexane/ isopropanol = 96/4, 254 nm absorbance), retention times: minor enantiomer (t_R = 10.30 min), major enantiomer (t_R = 7.79 min).

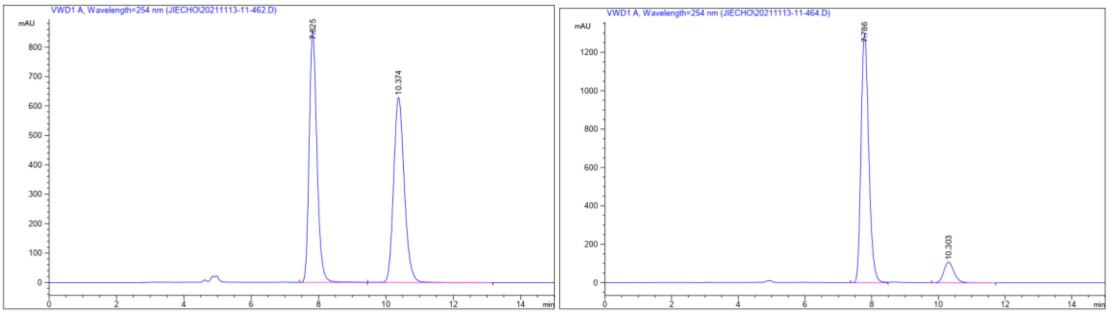

Figure S70. HPLC traces of 23d, Related to Figure 6

Signal 1: VWD1 A, Wavelength=254 nm

Peak #	RetTime [min]	Type	Width [min]	Area mAU *s	Height [mAU]	Area %
1	7.825	VB	0.2480	1.36569e4	852.56360	49.7152
2	10.374	BB	0.3408	1.38134e4	628.68689	50.2848
Totals :				2.74703e4	1481.25049	

Signal 1: VWD1 A, Wavelength=254 nm

Peak #	RetTime [min]	Type	Width [min]	Area mAU *s	Height [mAU]	Area %
1	7.786	BV	0.2524	2.09024e4	1294.66821	90.1116
2	10.303	VB	0.3271	2293.72559	107.70407	9.8884
Totals :				2.31961e4	1402.37228	

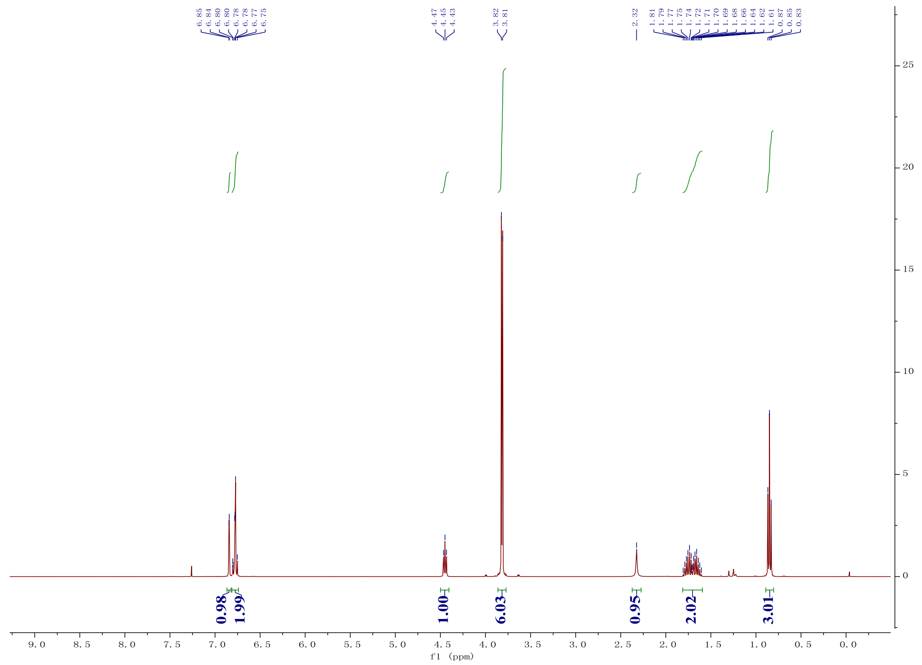

Figure S71. ^{1}H NMR spectrum of 23d, Related to Figure 6

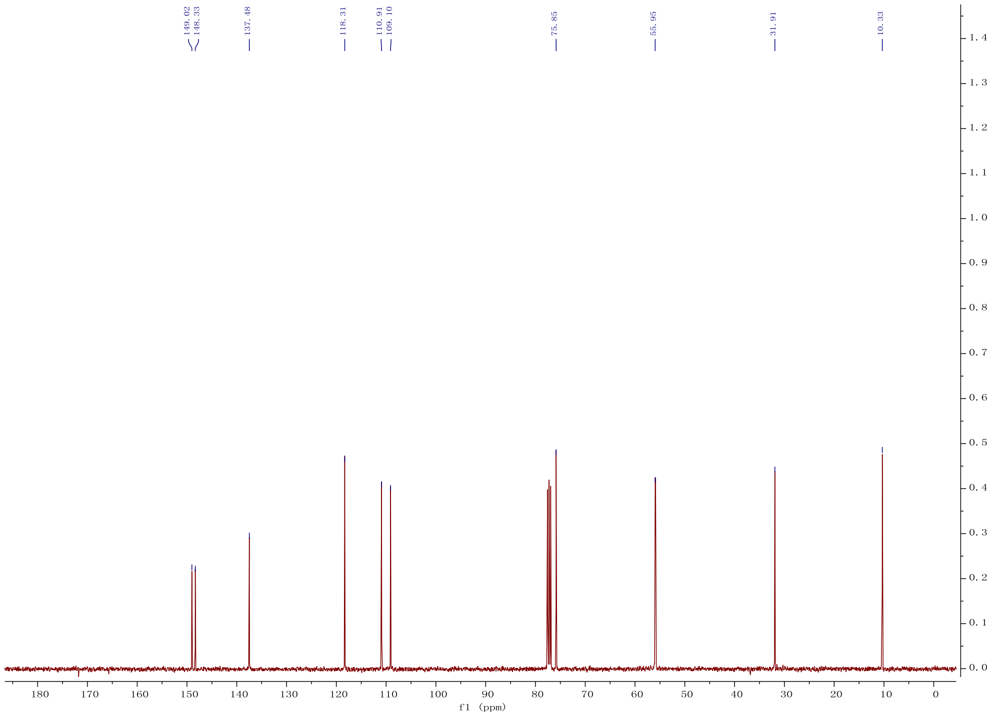

Figure S72. ^{13}C NMR spectrum of 23d, Related to Figure 6

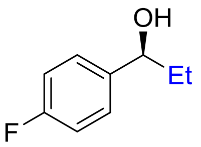

(*S*)-**23e**

HPLC: enantiomeric excess of **23e** (ee = 93%) was determined by high-performance liquid chromatography (HPLC) using a chiral stationary phase (IF column, flow rate = 1.0 mL/ min, eluent: hexane/ isopropanol = 98/2, 254 nm absorbance), retention times: minor enantiomer (t_R = 12.21 min), major enantiomer (t_R = 13.24 min).

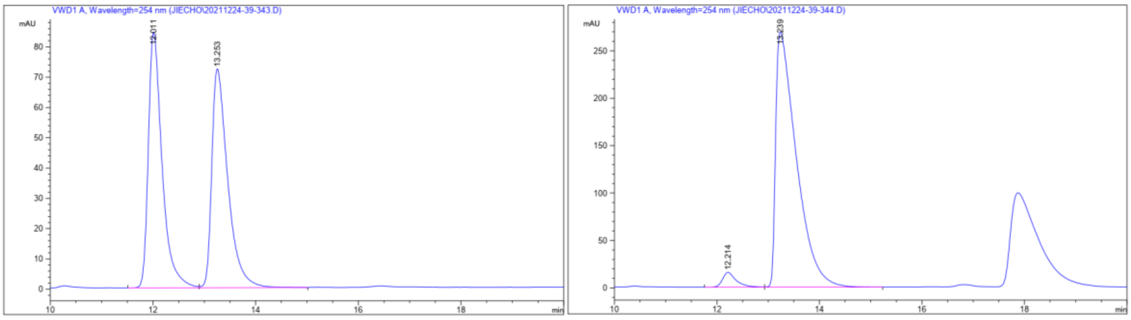

Figure S73. HPLC traces of 23e, Related to Figure 6

Signal 1: VWD1 A, Wavelength=254 nm

Peak #	RetTime [min]	Type	Width [min]	Area mAU *s	Height [mAU]	Area %
1	12.011	BV	0.2807	1568.10938	84.43968	50.1649
2	13.253	VB	0.3245	1557.79871	72.21828	49.8351
Totals :				3125.90808	156.65797	

Signal 1: VWD1 A, Wavelength=254 nm

Peak #	RetTime [min]	Type	Width [min]	Area mAU *s	Height [mAU]	Area %
1	12.214	BV	0.2731	292.28391	15.65972	3.7398
2	13.239	VB	0.4132	7523.26270	269.36960	96.2602
Totals :				7815.54660	285.02932	

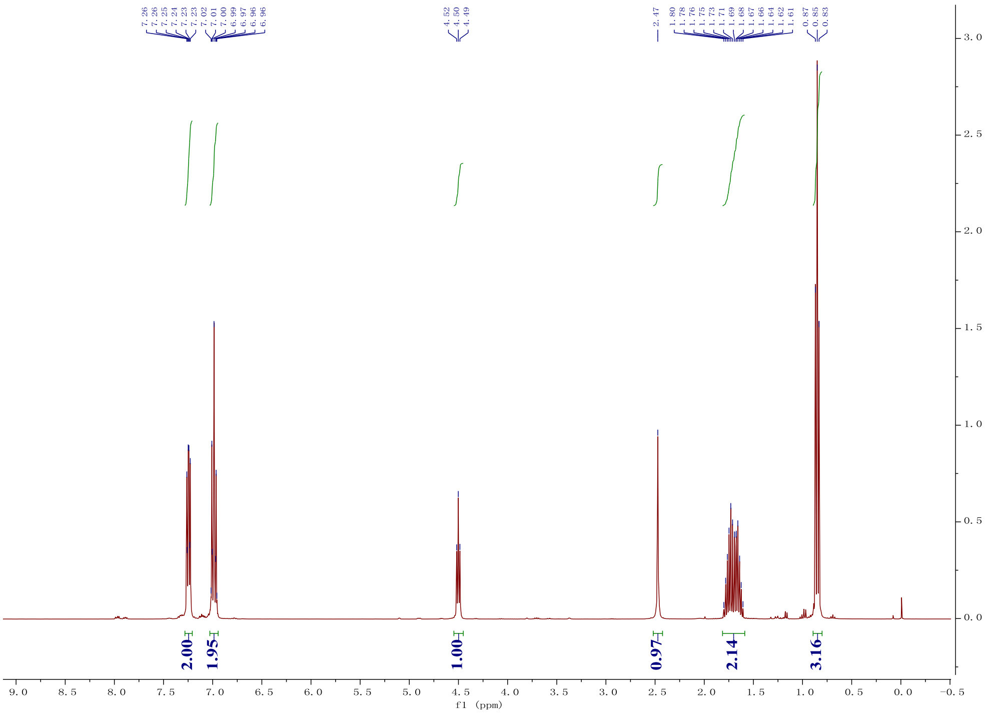

Figure S74. ^{1}H NMR spectrum of 23e, Related to Figure 6

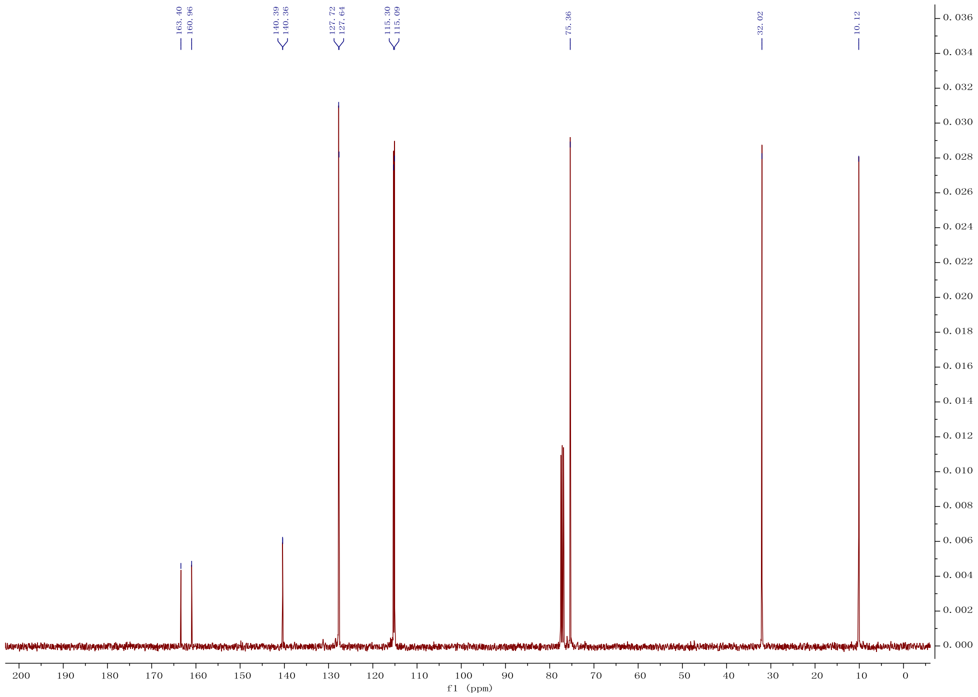

Figure S75. ^{13}C NMR spectrum of 23e, Related to Figure 6

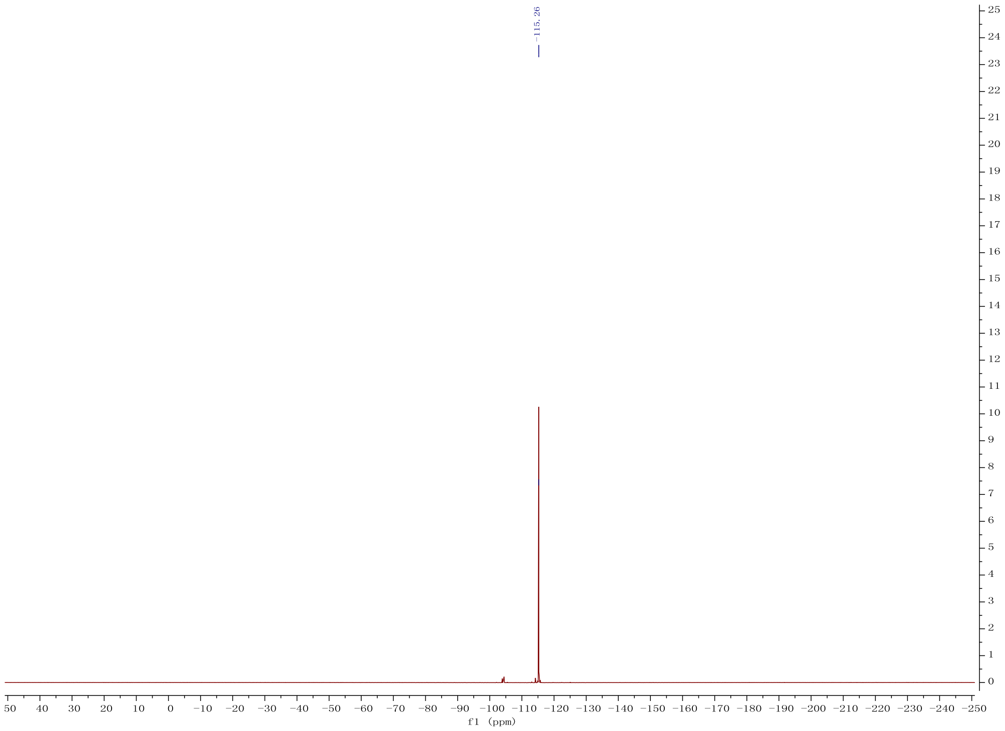

Figure S76. ^{19}F NMR spectrum of 23e, Related to Figure 6

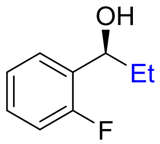

(*S*)-**23f**

HPLC: enantiomeric excess of **23f** (ee = 84%) was determined by high-performance liquid chromatography (HPLC) using a chiral stationary phase (ID column, flow rate = 1.0 mL/ min, eluent: hexane/ isopropanol = 95/5, 254 nm absorbance), retention times: minor enantiomer (t_R = 5.52 min), major enantiomer (t_R = 5.99 min).

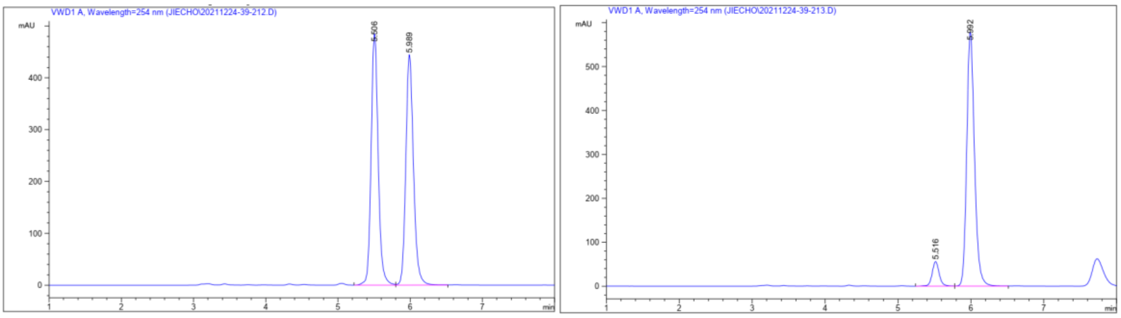

Figure S77. HPLC traces of 23f, Related to Figure 6

Signal 1: VWD1 A, Wavelength=254 nm

Peak #	RetTime [min]	Type	Width [min]	Area mAU *s	Height [mAU]	Area %
1	5.506	BV	0.1052	3284.44238	485.35776	50.0407
2	5.989	VV	0.1139	3279.10132	443.54474	49.9593
Totals :				6563.54370	928.90250	

Signal 1: VWD1 A, Wavelength=254 nm

Peak #	RetTime [min]	Type	Width [min]	Area mAU *s	Height [mAU]	Area %
1	5.516	BB	0.1026	371.73157	55.68695	8.0033
2	5.992	BV	0.1139	4273.00488	577.97748	91.9967
Totals :				4644.73645	633.66442	

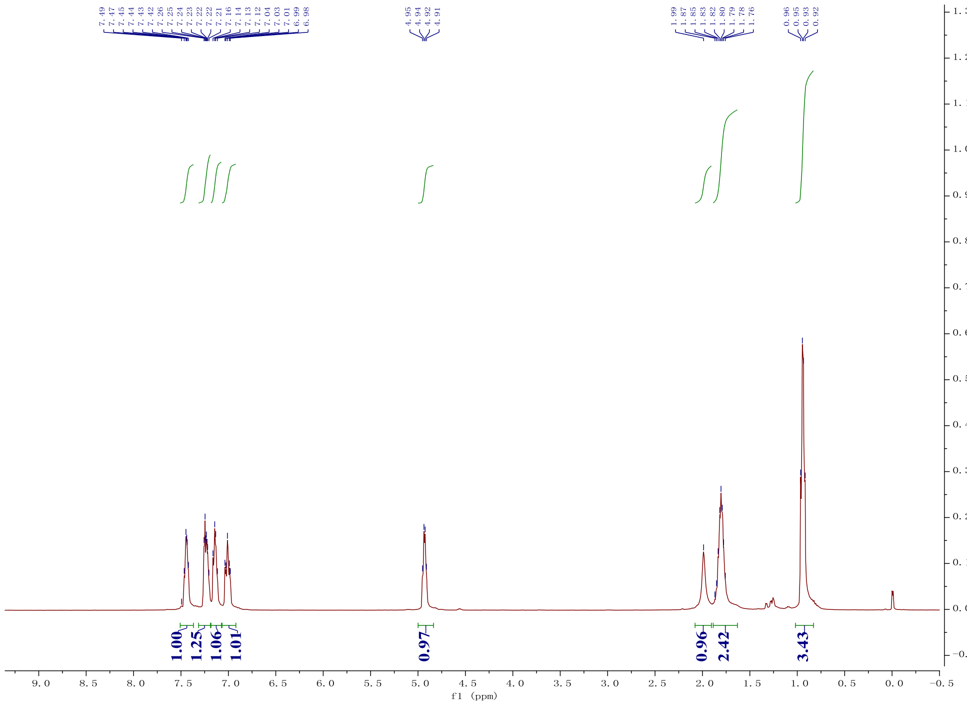

Figure S78. ^{1}H NMR spectrum of 23f, Related to Figure 6

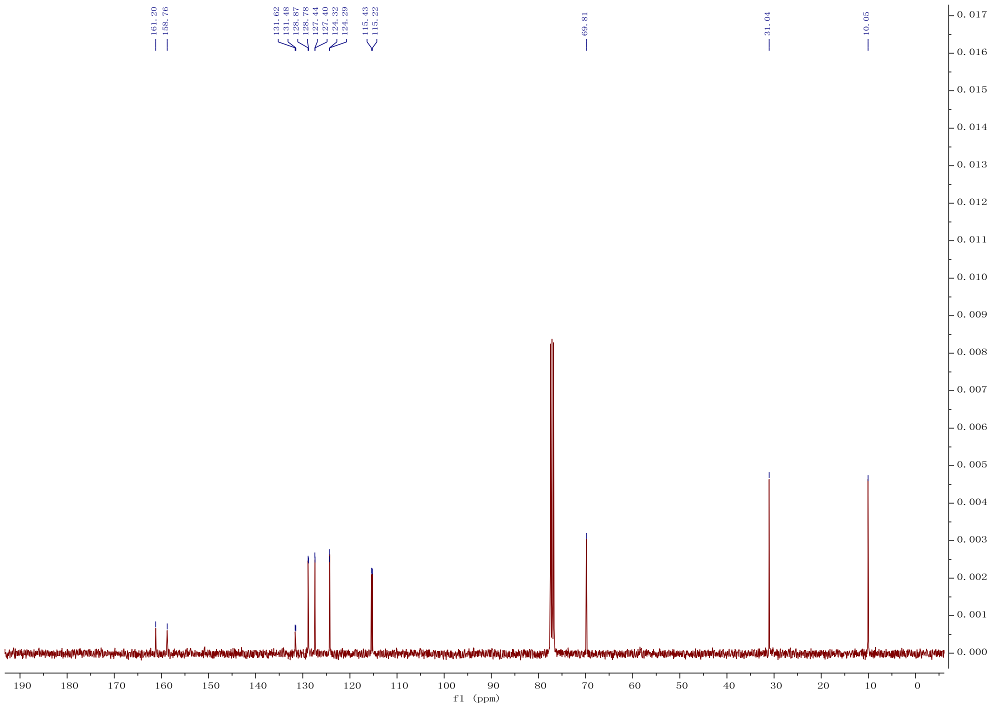

Figure S79. ^{13}C NMR spectrum of 23f, Related to Figure 6

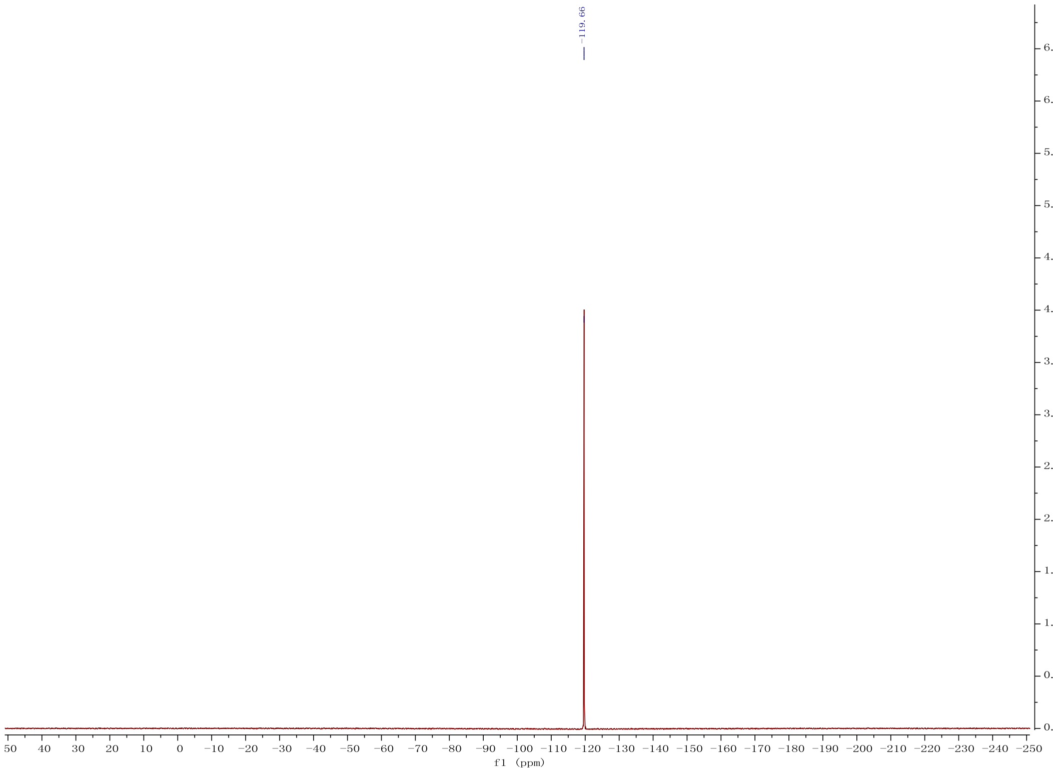

Figure S80. ^{19}F NMR spectrum of 23f, Related to Figure 6

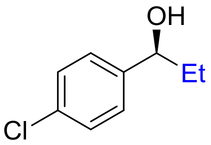

(*S*)-**23g**

HPLC: enantiomeric excess of **23g** (ee = 93%) was determined by high-performance liquid chromatography (HPLC) using a chiral stationary phase (OD-H column, flow rate = 1.0 mL/ min, eluent: hexane/ isopropanol = 98/2, 254 nm absorbance), retention times: minor enantiomer (t_R = 15.99 min), major enantiomer (t_R = 14.69 min).

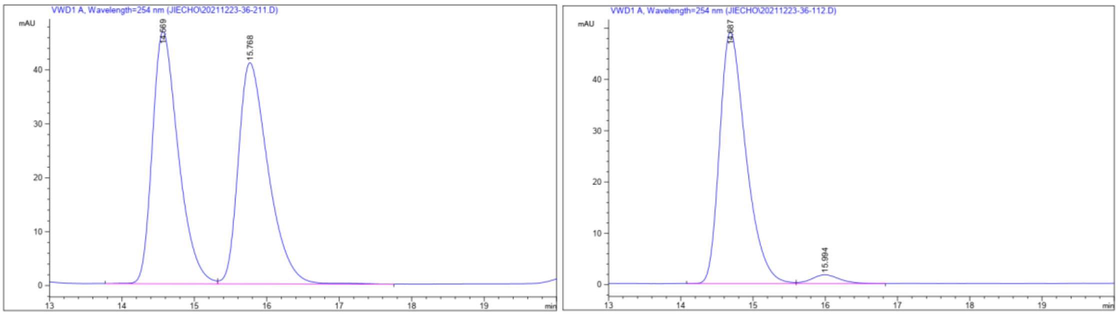

Figure S81. HPLC traces of 23g, Related to Figure 6

Signal 1: VWD1 A, Wavelength=254 nm

Peak #	RetTime [min]	Type	Width [min]	Area mAU *s	Height [mAU]	Area %
1	14.569	BV	0.3803	1162.74707	46.77419	49.7023
2	15.768	VB	0.4366	1176.67529	41.01486	50.2977
Totals :				2339.42236	87.78905	

Signal 1: VWD1 A, Wavelength=254 nm

Peak #	RetTime [min]	Type	Width [min]	Area mAU *s	Height [mAU]	Area %
1	14.687	BV	0.3834	1244.83862	49.05046	96.4407
2	15.994	VB	0.4162	45.94239	1.70532	3.5593
Totals :				1290.78101	50.75578	

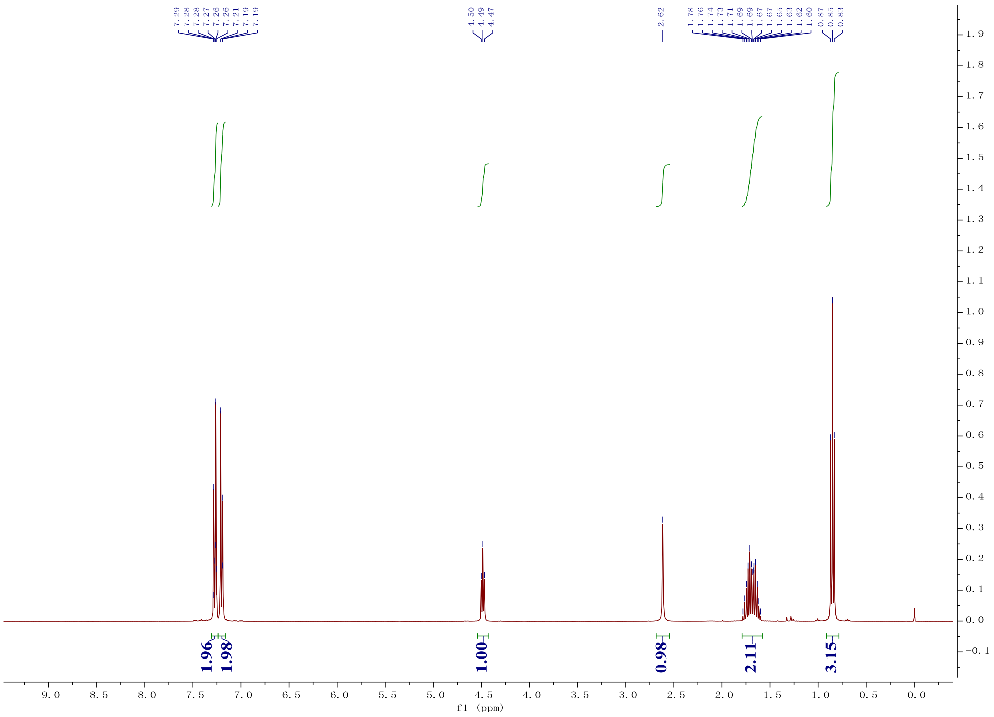

Figure S82. ^{1}H NMR spectrum of 23g, Related to Figure 6

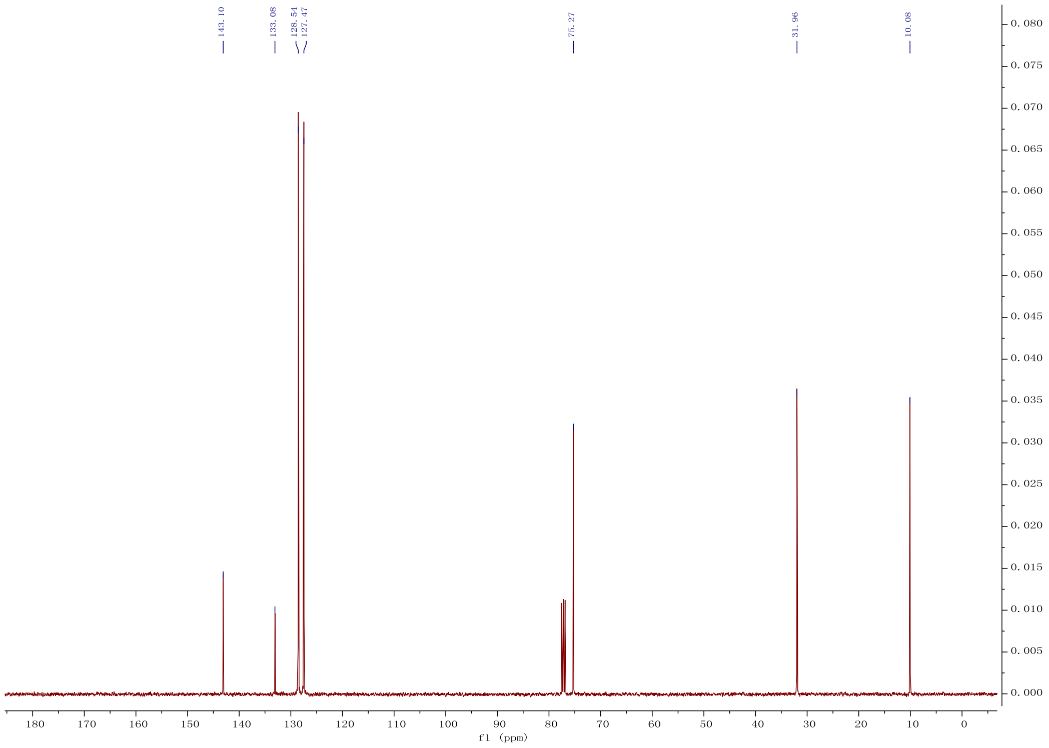

Figure S83. ^{13}C NMR spectrum of 23g, Related to Figure 6

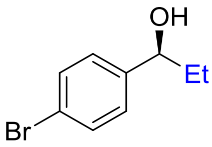

(*S*)-**23h**

ppm.

HPLC: enantiomeric excess of **23h** (ee = 92%) was determined by high-performance liquid chromatography (HPLC) using a chiral stationary phase (OD-H column, flow rate = 0.6 mL/ min, eluent: hexane/ isopropanol = 98/2, 254 nm absorbance), retention times: minor enantiomer (t_R = 28.27 min), major enantiomer (t_R = 25.80 min).

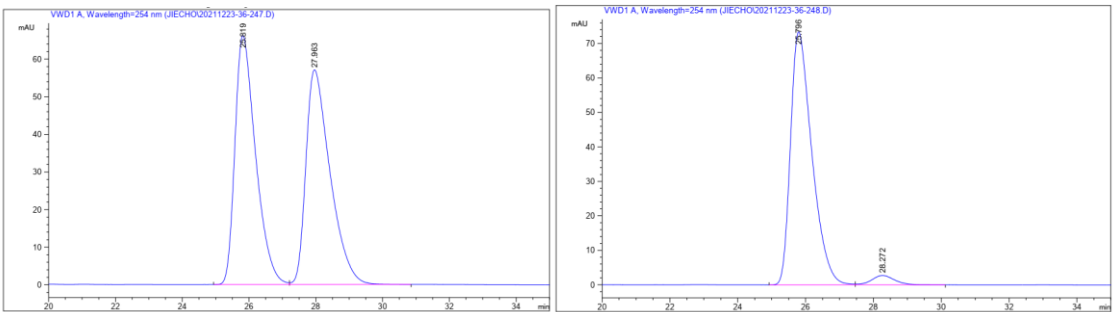

Figure S84. HPLC traces of 23h, Related to Figure 6

Signal 1: VWD1 A, Wavelength=254 nm

Peak #	RetTime [min]	Type	Width [min]	Area mAU *s	Height [mAU]	Area %
1	25.819	BV	0.6535	2847.46582	66.16389	49.7623
2	27.963	VB	0.7510	2874.66357	57.04161	50.2377
Totals :				5722.12939	123.20550	

Signal 1: VWD1 A, Wavelength=254 nm

Peak #	RetTime [min]	Type	Width [min]	Area mAU *s	Height [mAU]	Area %
1	25.796	BV	0.6619	3211.51709	73.39992	96.1000
2	28.272	VB	0.7019	130.33121	2.74767	3.9000
Totals :				3341.84830	76.14759	

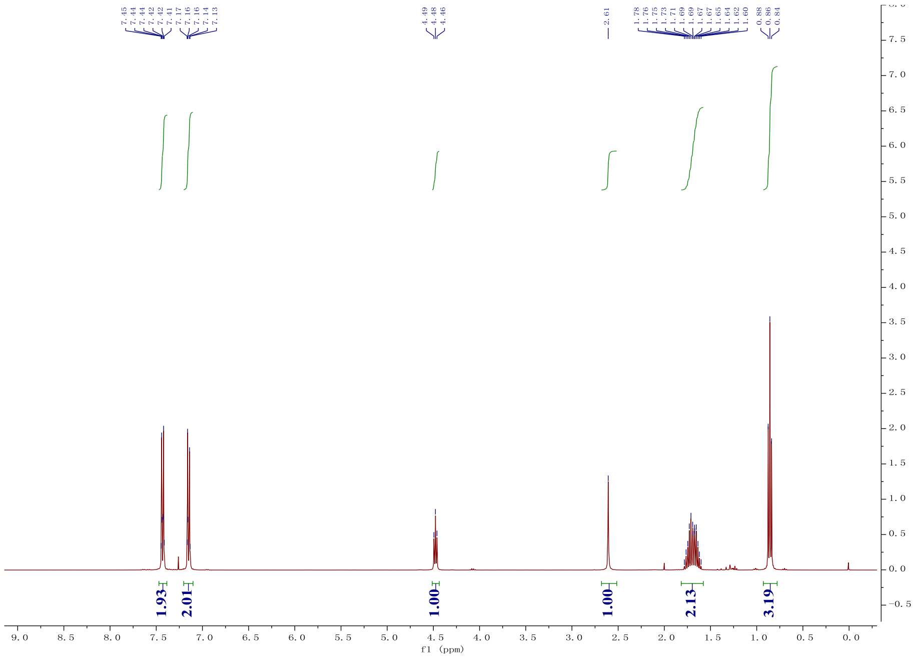

Figure S85. ^{1}H NMR spectrum of 23h, Related to Figure 6

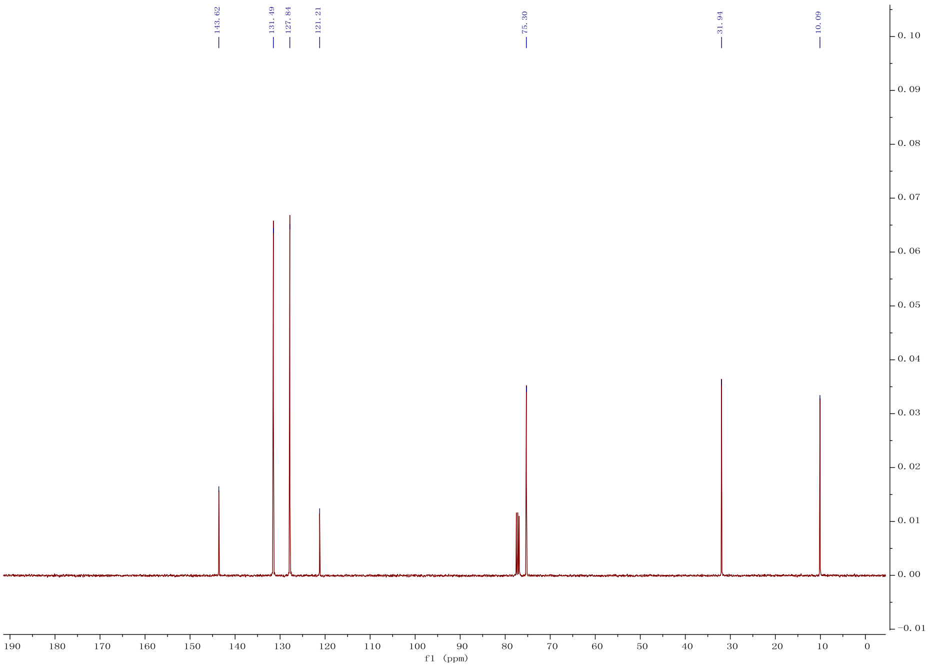

Figure S86. ^{13}C NMR spectrum of 23h, Related to Figure 6

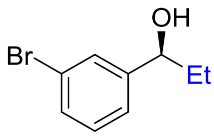

(*S*)-**23i**

HPLC: enantiomeric excess of **23i** (ee = 93%) was determined by high-performance liquid chromatography (HPLC) using a chiral stationary phase (OD-H column, flow rate = 1.0 mL/ min, eluent: hexane/ isopropanol = 96/4, 254 nm absorbance), retention times: minor enantiomer (t_R = 10.34 min), major enantiomer (t_R = 9.00 min).

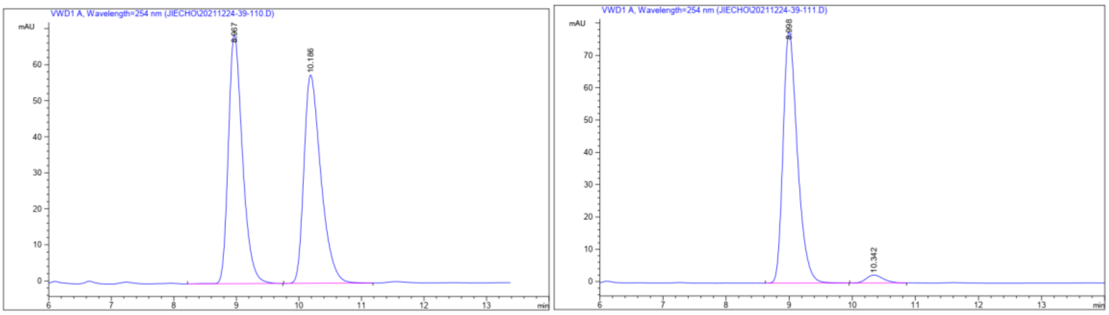

Figure S87. HPLC traces of 23i, Related to Figure 6

Signal 1: VWD1 A, Wavelength=254 nm

Peak #	RetTime [min]	Type	Width [min]	Area mAU *s	Height [mAU]	Area %
1	8.967	BB	0.2385	1084.90857	69.09742	49.8614
2	10.186	BV	0.2871	1090.94128	57.81048	50.1386
Totals :				2175.84985	126.90790	

Signal 1: VWD1 A, Wavelength=254 nm

Peak #	RetTime [min]	Type	Width [min]	Area mAU *s	Height [mAU]	Area %
1	8.998	BB	0.2413	1230.96338	77.83236	96.3938
2	10.342	BB	0.2907	46.05227	2.46574	3.6062
Totals :				1277.01565	80.29809	

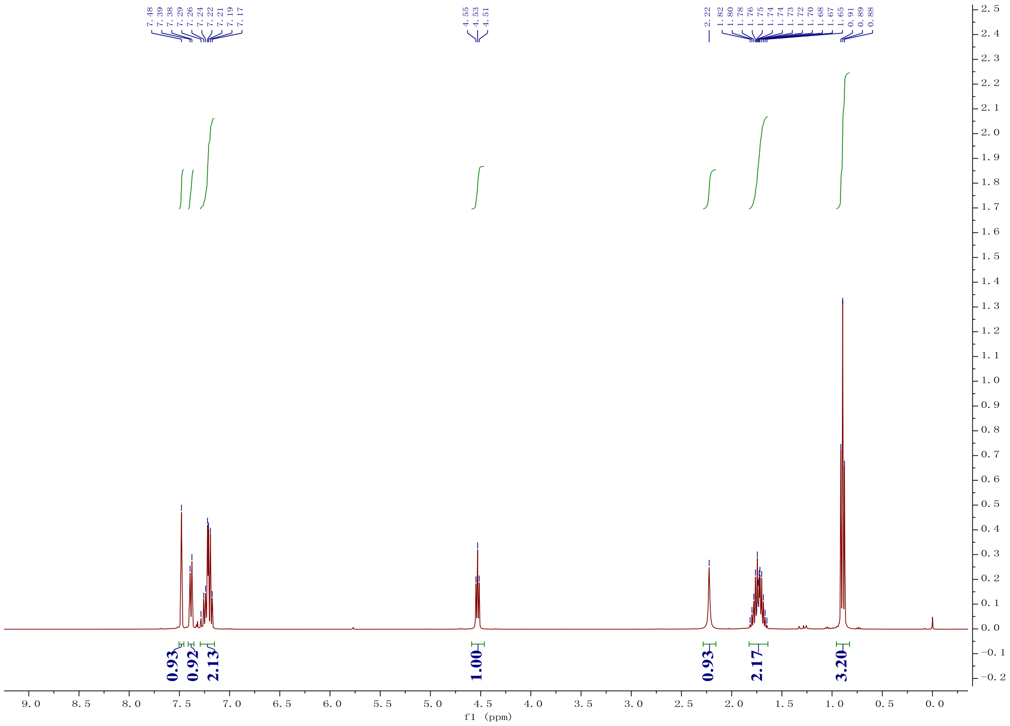

Figure S88. ^{1}H NMR spectrum of 23i, Related to Figure 6

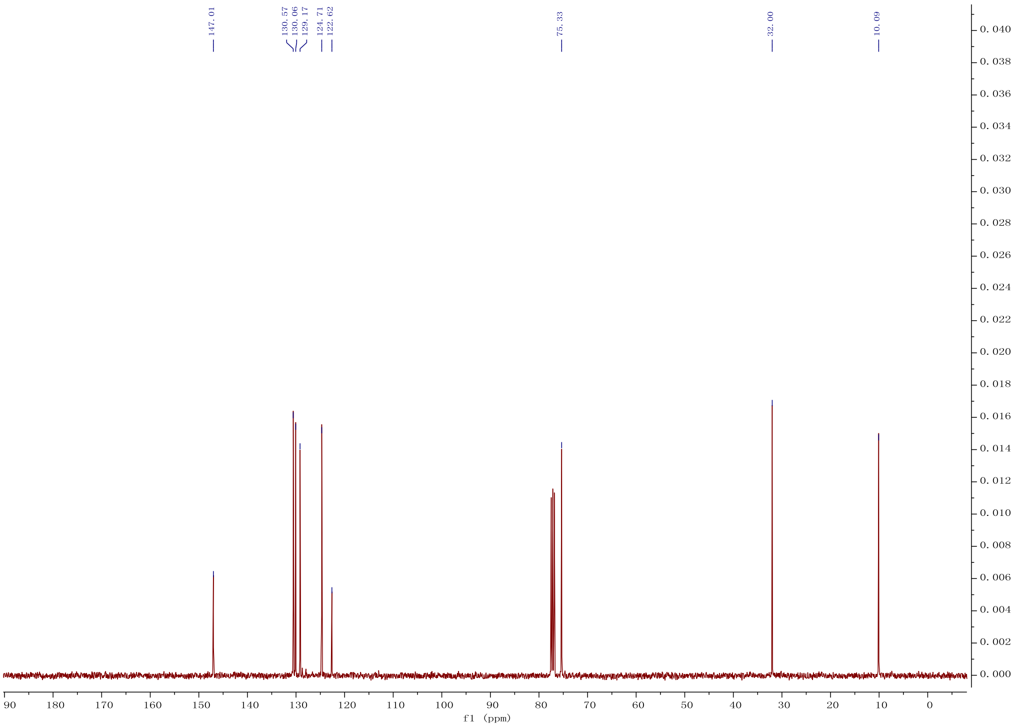

Figure S89. ^{13}C NMR spectrum of 23i, Related to Figure 6

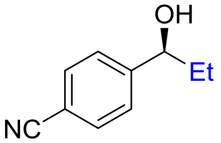

(S)-**23j**

HPLC: enantiomeric excess of **23j** (ee = 93%) was determined by high-performance liquid chromatography (HPLC) using a chiral stationary phase (ID column, flow rate = 0.8 mL/ min, eluent: hexane/ isopropanol = 97/3, 254 nm absorbance), retention times: minor enantiomer (t_R = 26.09 min), major enantiomer (t_R = 26.96 min).

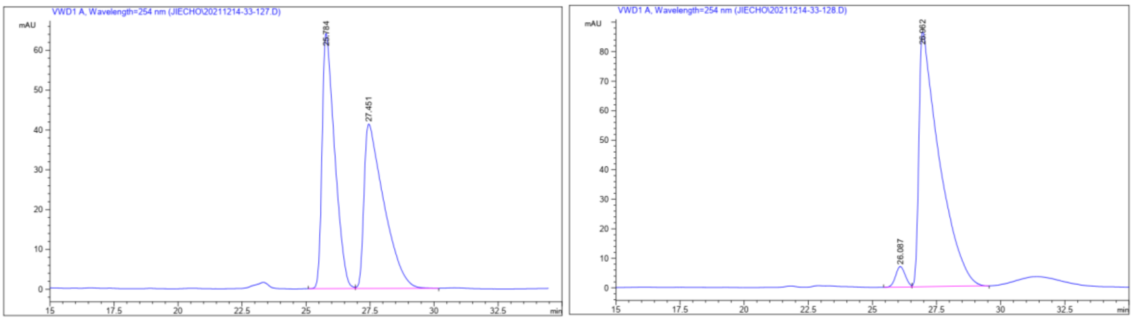

Figure S90. HPLC traces of 23j, Related to Figure 6

Signal 1: VWD1 A, Wavelength=254 nm

Peak #	RetTime [min]	Type	Width [min]	Area mAU *s	Height [mAU]	Area %
1	25.784	BV	0.5054	2192.26880	64.08096	49.5427
2	27.451	VB	0.7547	2232.73560	41.28281	50.4573
Totals :				4425.00439	105.36377	

Signal 1: VWD1 A, Wavelength=254 nm

Peak #	RetTime [min]	Type	Width [min]	Area mAU *s	Height [mAU]	Area %
1	26.087	BV	0.4112	181.42531	6.94129	3.6485
2	26.962	VB	0.7365	4791.22168	86.29939	96.3515
Totals :				4972.64699	93.24067	

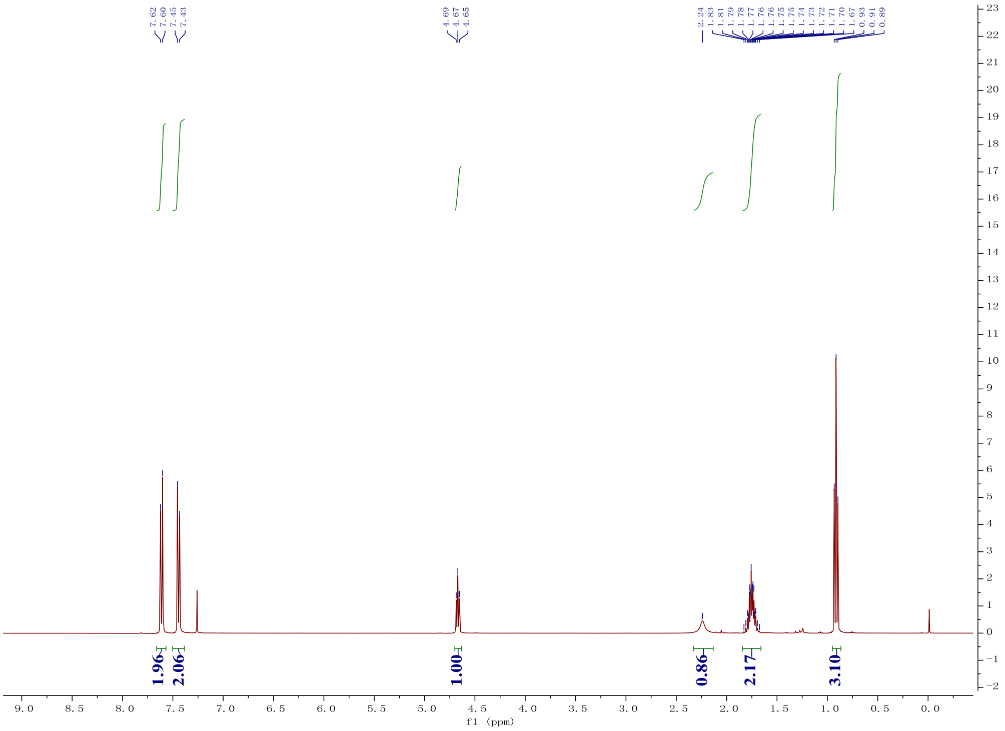

Figure S91. ^{1}H NMR spectrum of 23j, Related to Figure 6

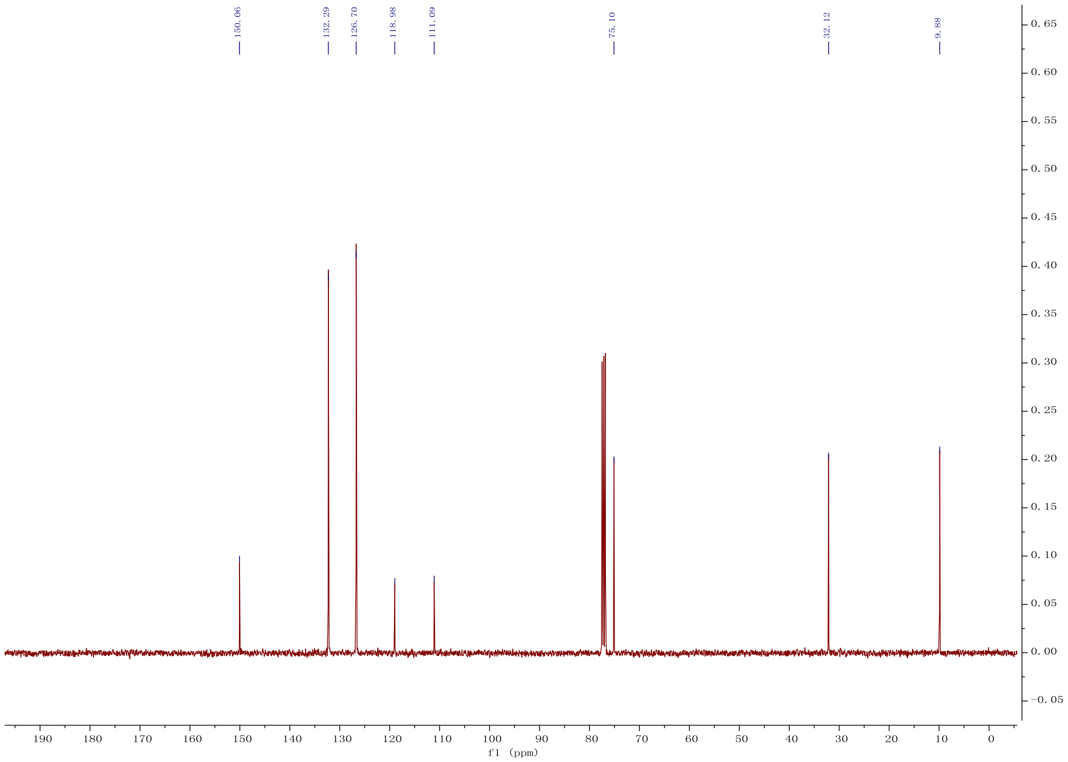

Figure S92. ^{13}C NMR spectrum of 23j, Related to Figure 6

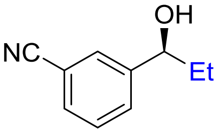

(*S*)-**23k**

HPLC: enantiomeric excess of **23k** (ee = 94%) was determined by high-performance liquid chromatography (HPLC) using a chiral stationary phase (ID column, flow rate = 1.0 mL/ min, eluent: hexane/ isopropanol = 95/5, 254 nm absorbance), retention times: minor enantiomer (t_R = 18.35 min), major enantiomer (t_R = 20.50 min).

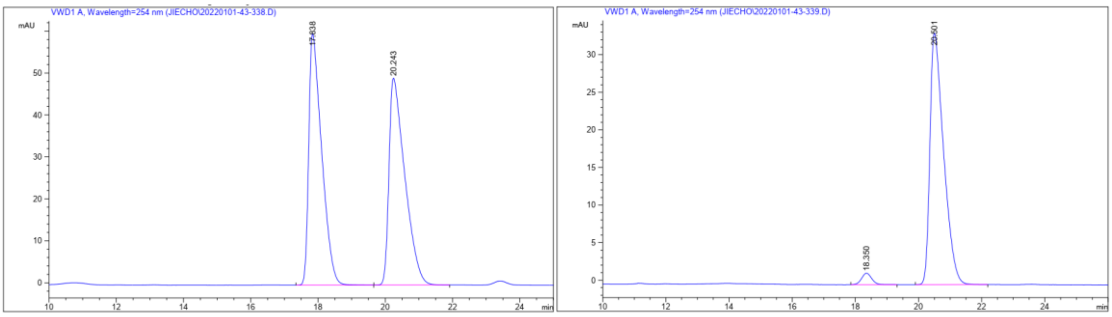

Figure S93. HPLC traces of 23k, Related to Figure 6

Signal 1: VWD1 A, Wavelength=254 nm

Peak #	RetTime [min]	Type	Width [min]	Area mAU *s	Height [mAU]	Area %
1	17.838	BB	0.3952	1597.24792	59.95994	49.8406
2	20.243	BB	0.4735	1607.46155	49.30806	50.1594
Totals :				3204.70947	109.26800	

Signal 1: VWD1 A, Wavelength=254 nm

Peak #	RetTime [min]	Type	Width [min]	Area mAU *s	Height [mAU]	Area %
1	18.350	BB	0.3425	33.89666	1.51526	3.2602
2	20.501	BB	0.4452	1005.79858	33.47583	96.7398
Totals :				1039.69524	34.99108	

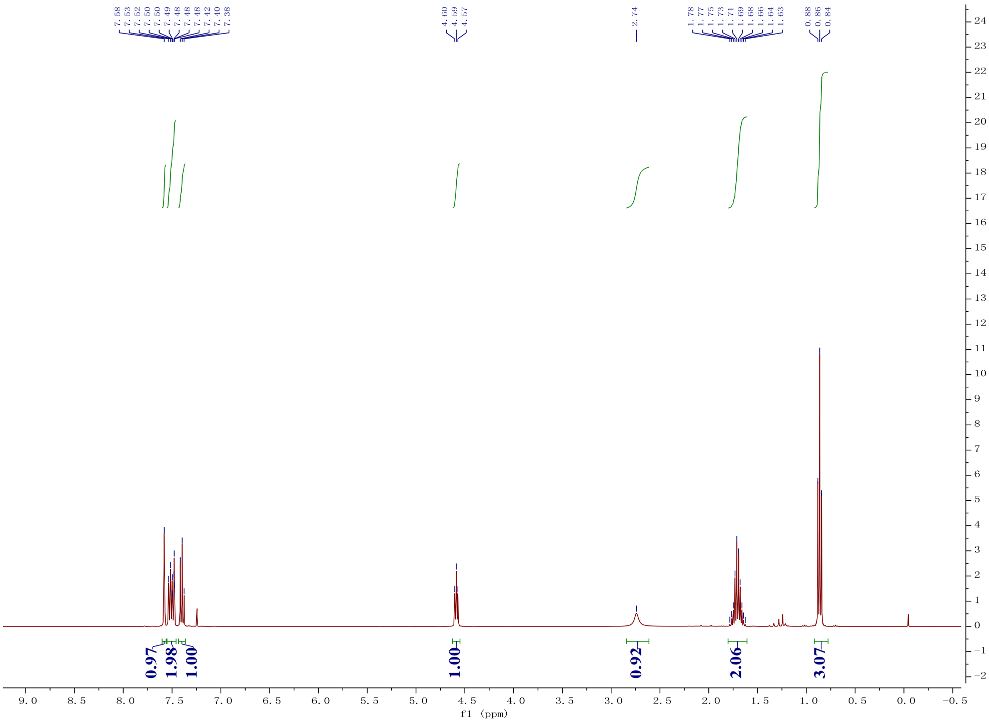

Figure S94. ^{1}H NMR spectrum of 23k, Related to Figure 6

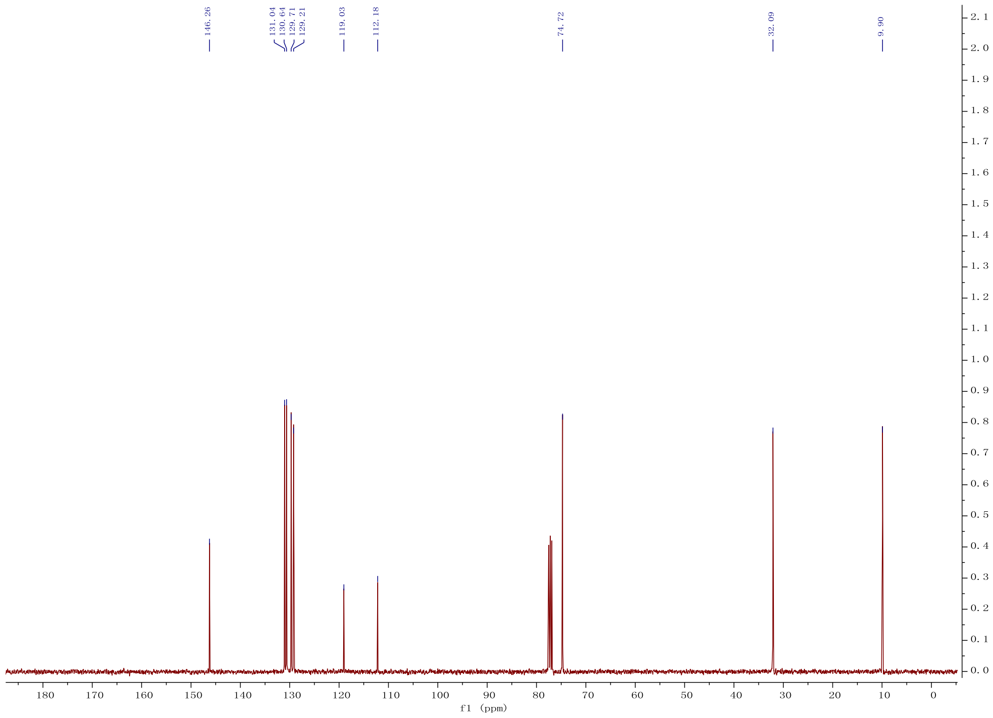

Figure S95. ^{13}C NMR spectrum of 23k, Related to Figure 6

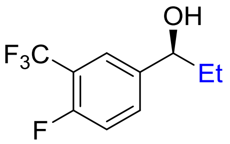

(*S*)-**23l**

HPLC: enantiomeric excess of **23l** (ee = 88%) was determined by high-performance liquid chromatography (HPLC) using a chiral stationary phase (AY-H column, flow rate = 0.8 mL/ min, eluent: hexane/ isopropanol = 96/4, 254 nm absorbance), retention times: minor enantiomer (t_R = 5.07 min), major enantiomer (t_R = 5.65 min).

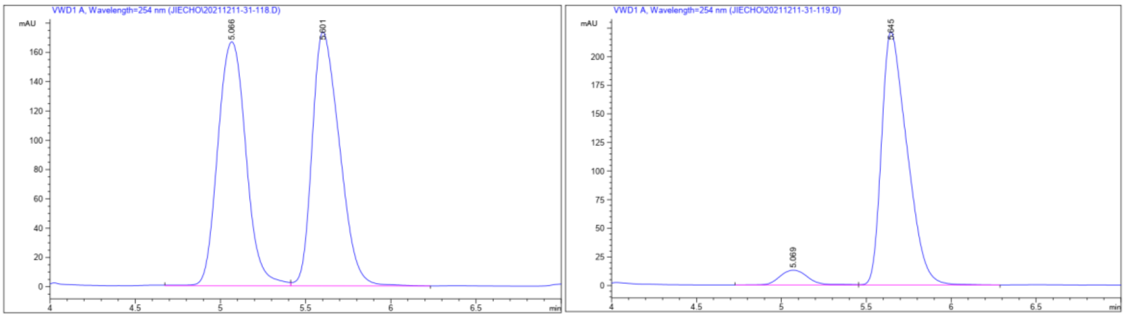

Figure S96. HPLC traces of 23l, Related to Figure 6

Signal 1: VWD1 A, Wavelength=254 nm

Peak #	RetTime [min]	Type	Width [min]	Area mAU *s	Height [mAU]	Area %
1	5.066	VV	0.1840	1917.62793	166.39598	49.8524
2	5.601	VB	0.1794	1928.98376	173.27333	50.1476
Totals :				3846.61169	339.66931	

Signal 1: VWD1 A, Wavelength=254 nm

Peak #	RetTime [min]	Type	Width [min]	Area mAU *s	Height [mAU]	Area %
1	5.069	BV	0.1831	149.24313	12.89415	6.0837
2	5.645	VB	0.1564	2303.91870	221.38692	93.9163
Totals :				2453.16183	234.28106	

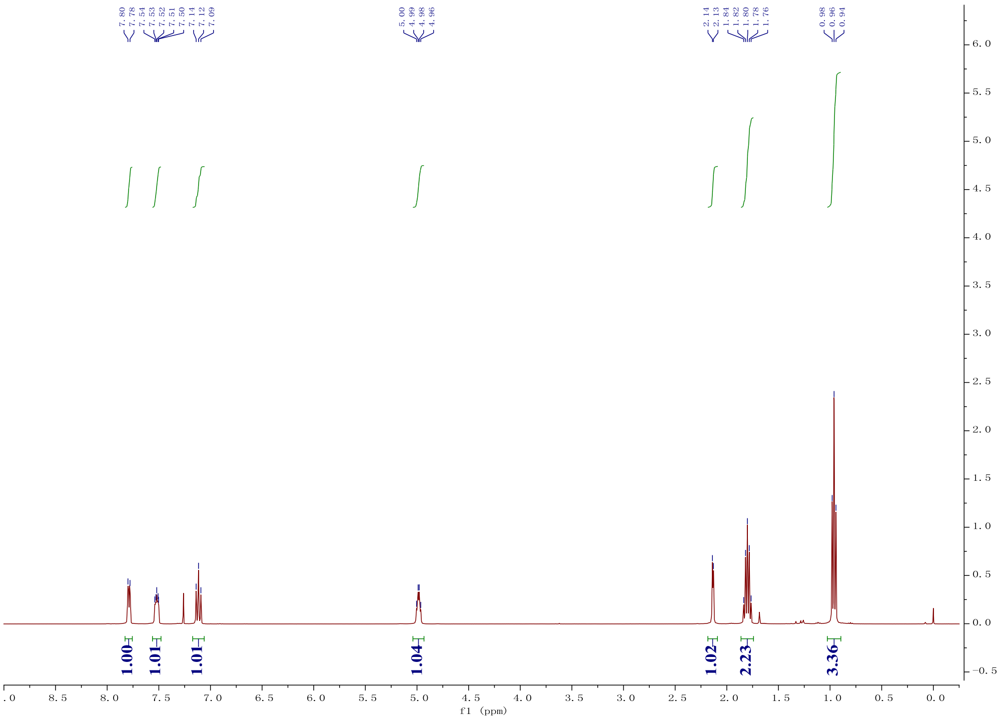

Figure S97. ^{1}H NMR spectrum of 23l, Related to Figure 6

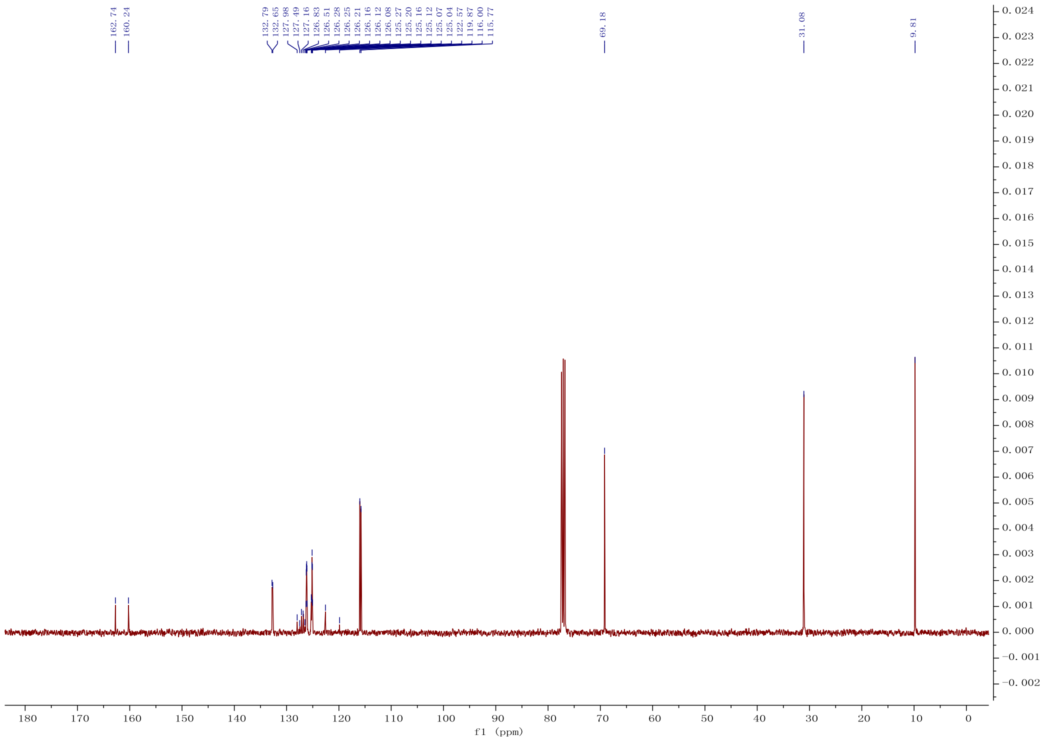

Figure S98. ^{13}C NMR spectrum of 23l, Related to Figure 6

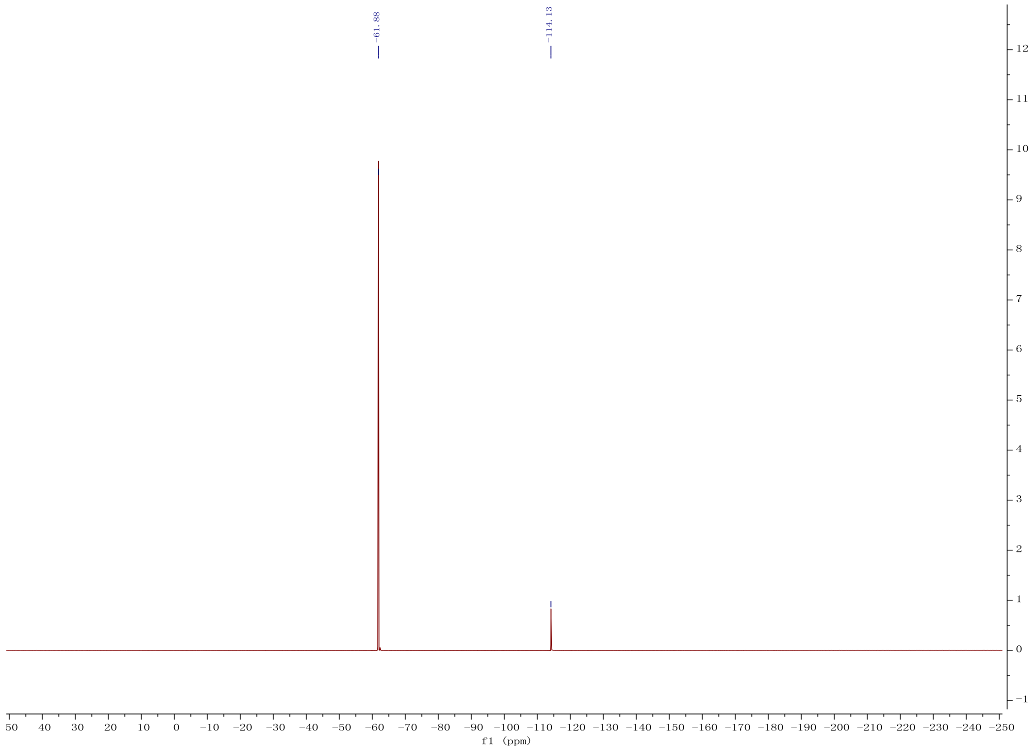

Figure S99. ^{19}F NMR spectrum of 23l, Related to Figure 6

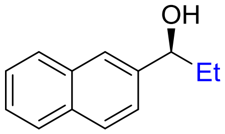

(*S*)-**23m**

HPLC: enantiomeric excess of **23m** (ee = 92%) was determined by high-performance liquid chromatography (HPLC) using a chiral stationary phase (OD-H column, flow rate = 1.0 mL/ min, eluent: hexane/ isopropanol = 96/4, 254 nm absorbance), retention times: minor enantiomer (t_R = 20.77 min), major enantiomer (t_R = 20.16 min).

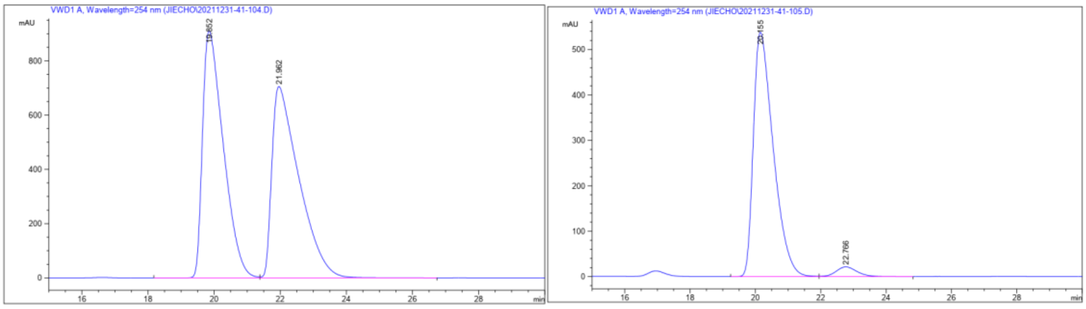

Figure S100. HPLC traces of 23m, Related to Figure 6

Signal 1: VWD1 A, Wavelength=254 nm

Peak #	RetTime [min]	Type	Width [min]	Area mAU *s	Height [mAU]	Area %
1	19.852	BV	0.6537	3.96573e4	910.57642	49.7872
2	21.962	VB	0.8509	3.99962e4	705.28143	50.2128
Totals :				7.96535e4	1615.85785	

Signal 1: VWD1 A, Wavelength=254 nm

Peak #	RetTime [min]	Type	Width [min]	Area mAU *s	Height [mAU]	Area %
1	20.155	BV	0.6384	2.26676e4	538.21783	95.9634
2	22.766	VB	0.6834	953.49115	21.37689	4.0366
Totals :				2.36211e4	559.59473	

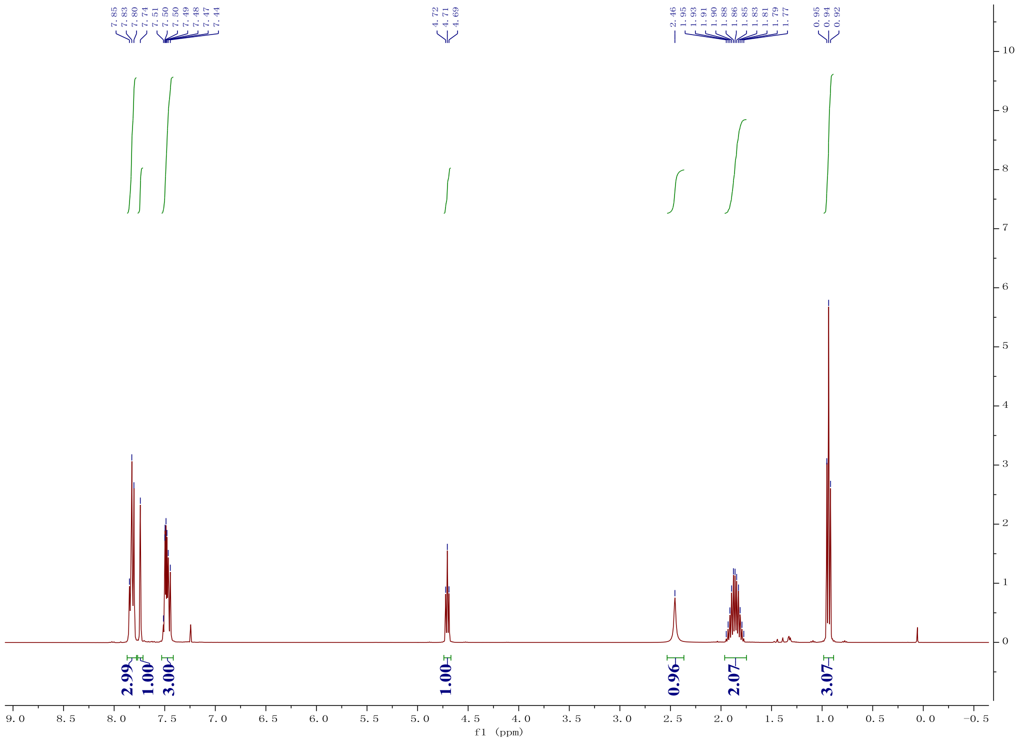

Figure S101. ^{1}H NMR spectrum of 23m, Related to Figure 6

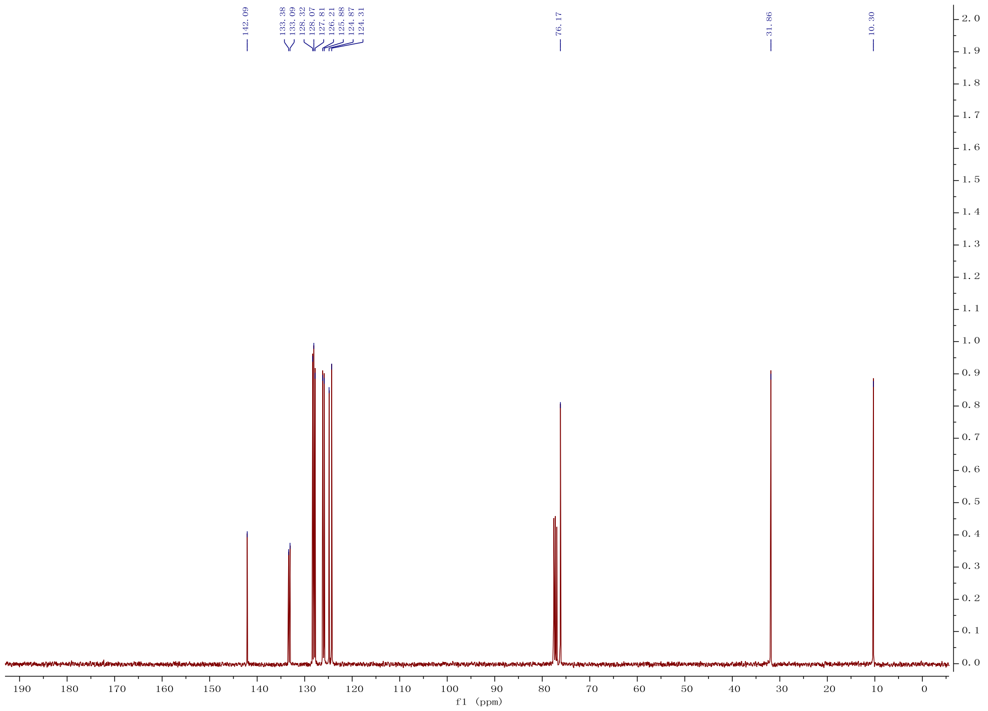

Figure S102. ^{13}C NMR spectrum of 23m, Related to Figure 6

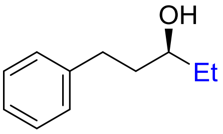

(*S*)-**23n**

HPLC: enantiomeric excess of **23n** (ee = 87%) was determined by high-performance liquid chromatography (HPLC) using a chiral stationary phase (AD-H column, flow rate = 1.0 mL/ min, eluent: hexane/ isopropanol = 96/4, 254 nm absorbance), retention times: minor enantiomer (t_R = 10.61 min), major enantiomer (t_R = 8.90 min).

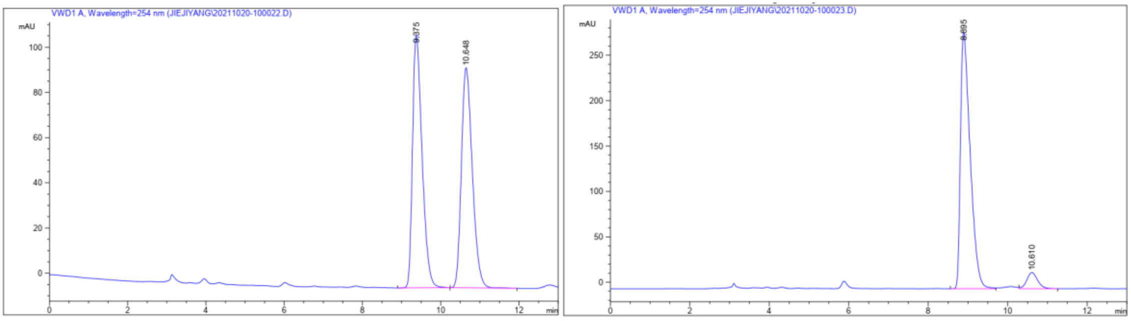

Figure S103. HPLC traces of 23n, Related to Figure 6

Signal 1: VWD1 A, Wavelength=254 nm

Peak #	RetTime [min]	Type	Width [min]	Area mAU *s	Height [mAU]	Area %
1	9.375	BB	0.2584	1893.41748	111.96207	50.0655
2	10.648	BB	0.2990	1888.46130	97.40170	49.9345
Totals :				3781.87878	209.36378	

Signal 1: VWD1 A, Wavelength=254 nm

Peak #	RetTime [min]	Type	Width [min]	Area mAU *s	Height [mAU]	Area %
1	8.895	BV	0.2559	4784.49609	282.38409	93.4917
2	10.610	VB	0.2893	333.06454	17.71025	6.5083
Totals :				5117.56064	300.09435	

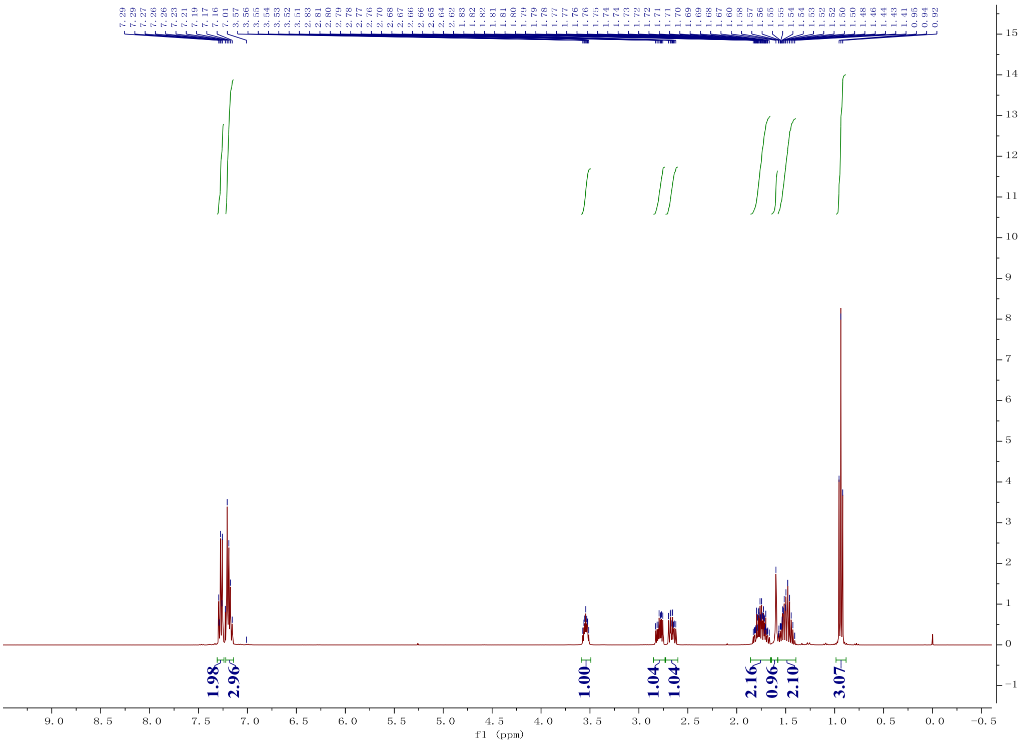

Figure S104. ^{1}H NMR spectrum of 23n, Related to Figure 6

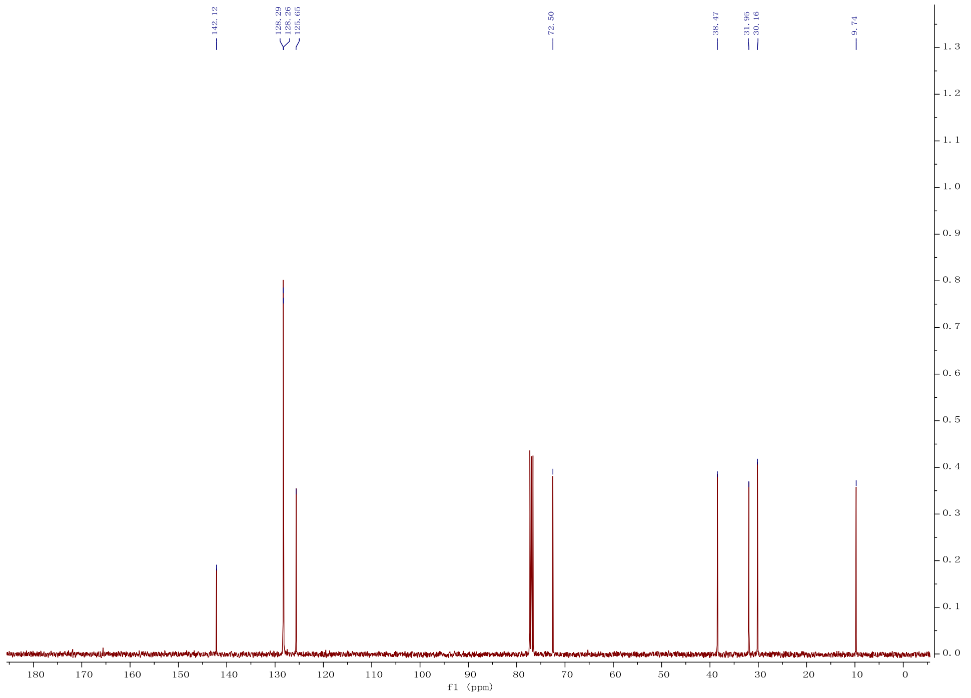

Figure S105. ^{13}C NMR spectrum of 23n, Related to Figure 6

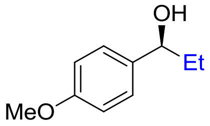

(*S*)-**23o**

HPLC: enantiomeric excess of **23o** (ee = 91%) was determined by high-performance liquid chromatography (HPLC) using a chiral stationary phase (OD-H column, flow rate = 0.8 mL/ min, eluent: hexane/ isopropanol = 96/4, 254 nm absorbance), retention times: minor enantiomer (t_R = 14.35 min), major enantiomer (t_R = 12.43 min).

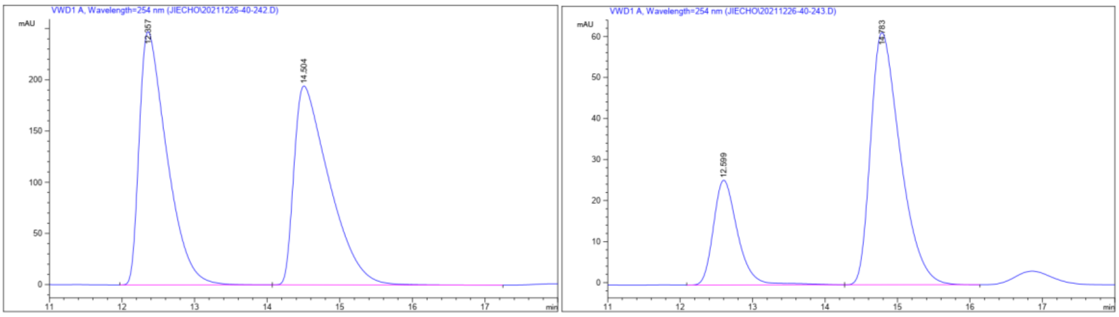

Figure S106. HPLC traces of 23o, Related to Figure 6

Signal 1: VWD1 A, Wavelength=254 nm

Peak #	RetTime [min]	Type	Width [min]	Area mAU *s	Height [mAU]	Area %
1	12.357	BB	0.3953	6523.72754	247.18260	49.9560
2	14.504	BB	0.4975	6535.22266	194.14665	50.0440
Totals :				1.30590e4	441.32925	

Signal 1: VWD1 A, Wavelength=254 nm

Peak #	RetTime [min]	Type	Width [min]	Area mAU *s	Height [mAU]	Area %
1	12.599	BB	0.3539	596.50818	25.54988	25.4368
2	14.783	BB	0.4310	1748.55542	61.44253	74.5632
Totals :				2345.06360	86.99242	

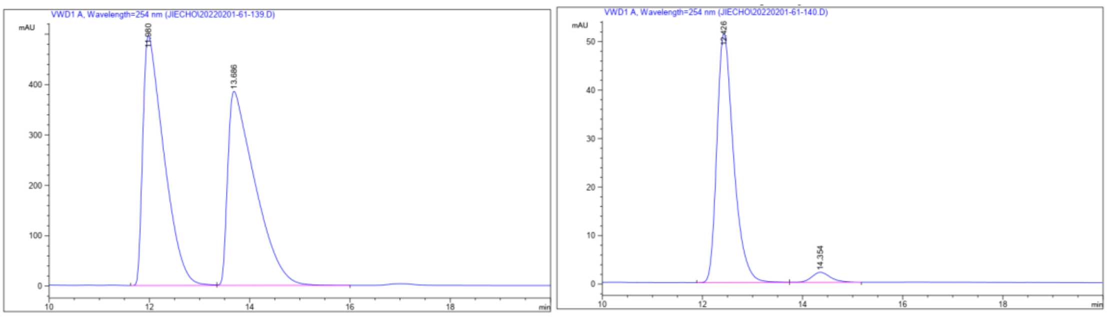

Figure S107. HPLC traces of 23o, Related to Figure 6

Signal 1: VWD1 A, Wavelength=254 nm

Peak #	RetTime [min]	Type	Width [min]	Area mAU *s	Height [mAU]	Area %
1	11.980	BV	0.4335	1.44558e4	495.59543	49.8360
2	13.686	VB	0.5370	1.45510e4	385.00458	50.1640
Totals :				2.90068e4	880.60001	

Signal 1: VWD1 A, Wavelength=254 nm

Peak #	RetTime [min]	Type	Width [min]	Area mAU *s	Height [mAU]	Area %
1	12.426	BV	0.3520	1191.13025	51.37113	95.6451
2	14.354	VB	0.4006	54.23489	2.05844	4.3549
Totals :				1245.36514	53.42957	

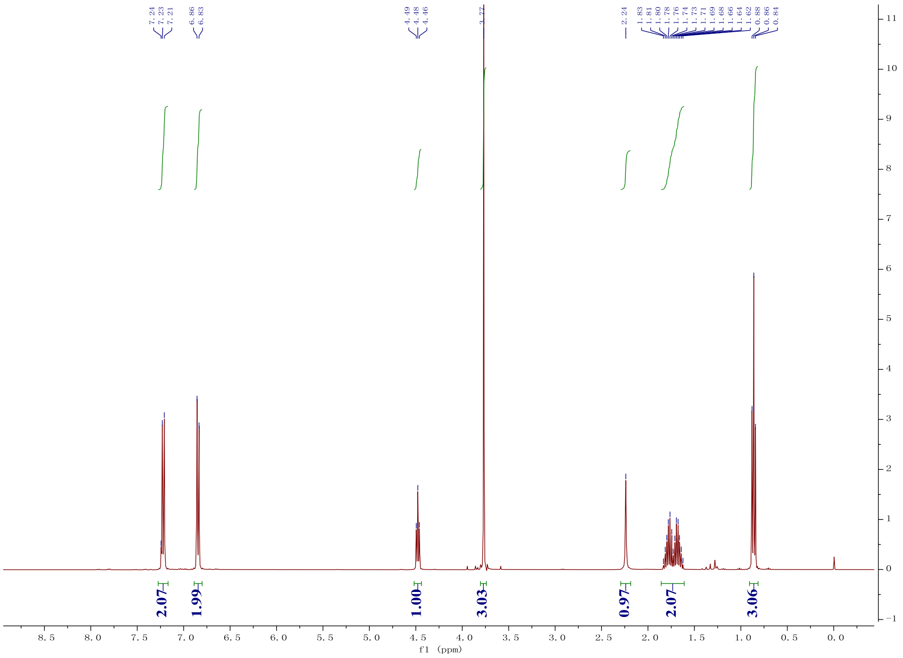

Figure S108. ¹H NMR spectrum of 23o, Related to Figure 6

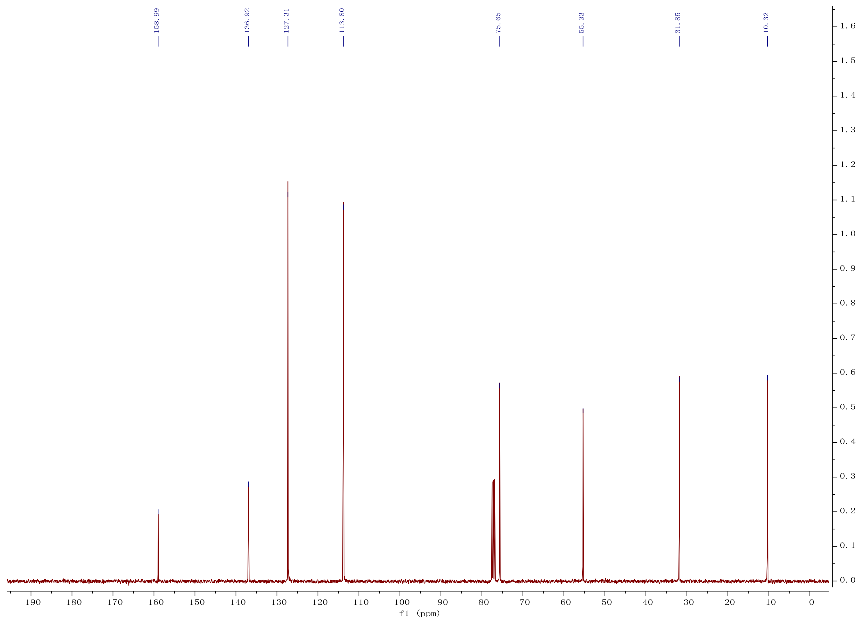

Figure S109. ^{13}C NMR spectrum of 23o, Related to Figure 6

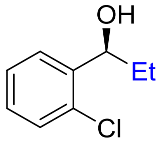

(*S*)-**23p**

HPLC: enantiomeric excess of **23p** (ee = 81%) was determined by high-performance liquid chromatography (HPLC) using a chiral stationary phase (ID column, flow rate = 1.0 mL/ min, eluent: hexane/ isopropanol = 96/4, 254 nm absorbance), retention times: minor enantiomer (t_R = 6.33 min), major enantiomer (t_R = 5.84 min).

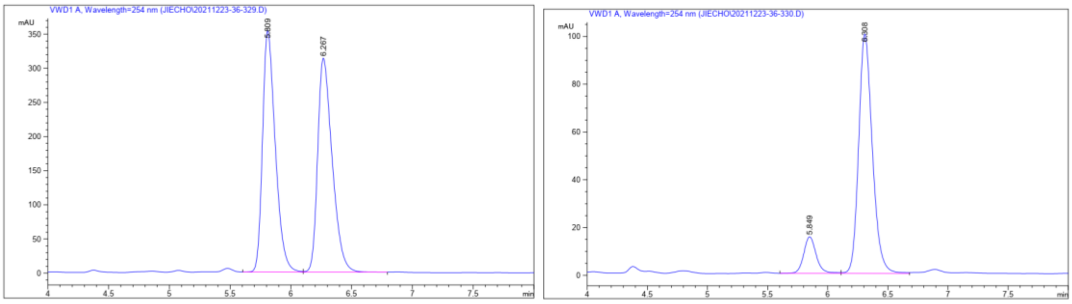

Figure S110. HPLC traces of 23p, Related to Figure 6

Signal 1: VWD1 A, Wavelength=254 nm

Peak #	RetTime [min]	Type	Width [min]	Area mAU *s	Height [mAU]	Area %
1	5.809	BV	0.1115	2587.18652	353.87332	49.8832
2	6.267	VB	0.1273	2599.29907	313.45938	50.1168
Totals :				5186.48560	667.33270	

Signal 1: VWD1 A, Wavelength=254 nm

Peak #	RetTime [min]	Type	Width [min]	Area mAU *s	Height [mAU]	Area %
1	5.849	BV	0.1105	110.34198	15.26464	12.2372
2	6.308	VV	0.1214	791.35034	100.08033	87.7628
Totals :				901.69232	115.34497	

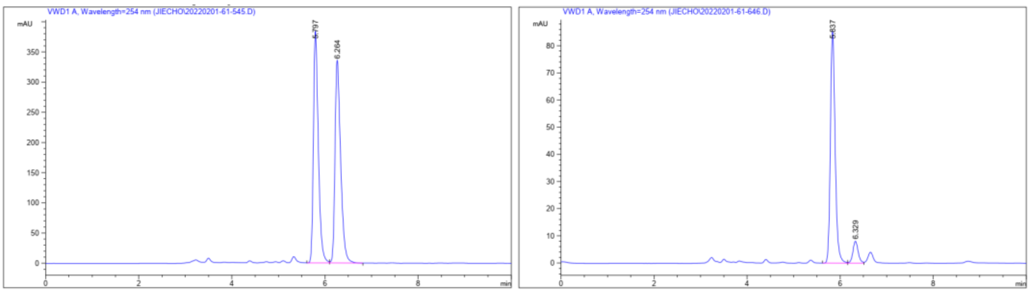

Figure S111. HPLC traces of 23p, Related to Figure 6

Signal 1: VWD1 A, Wavelength=254 nm

Peak #	RetTime [min]	Type	Width [min]	Area mAU *s	Height [mAU]	Area %
1	5.797	BV	0.1125	2827.79541	382.25641	49.8274
2	6.264	VB	0.1311	2847.38965	335.25888	50.1726
Totals :				5675.18506	717.51529	

Signal 1: VWD1 A, Wavelength=254 nm

Peak #	RetTime [min]	Type	Width [min]	Area mAU *s	Height [mAU]	Area %
1	5.837	BV	0.1093	608.19171	85.36367	90.4145
2	6.329	VV	0.1221	64.47861	8.09137	9.5855
Totals :				672.67033	93.45504	

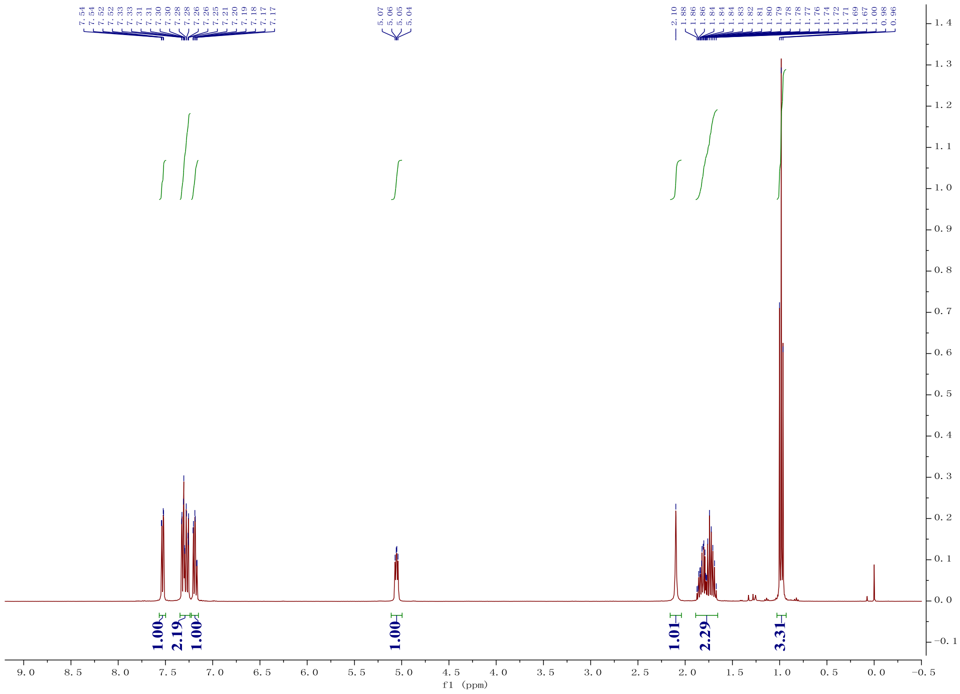

Figure S112. ^{1}H NMR spectrum of 23p, Related to Figure 6

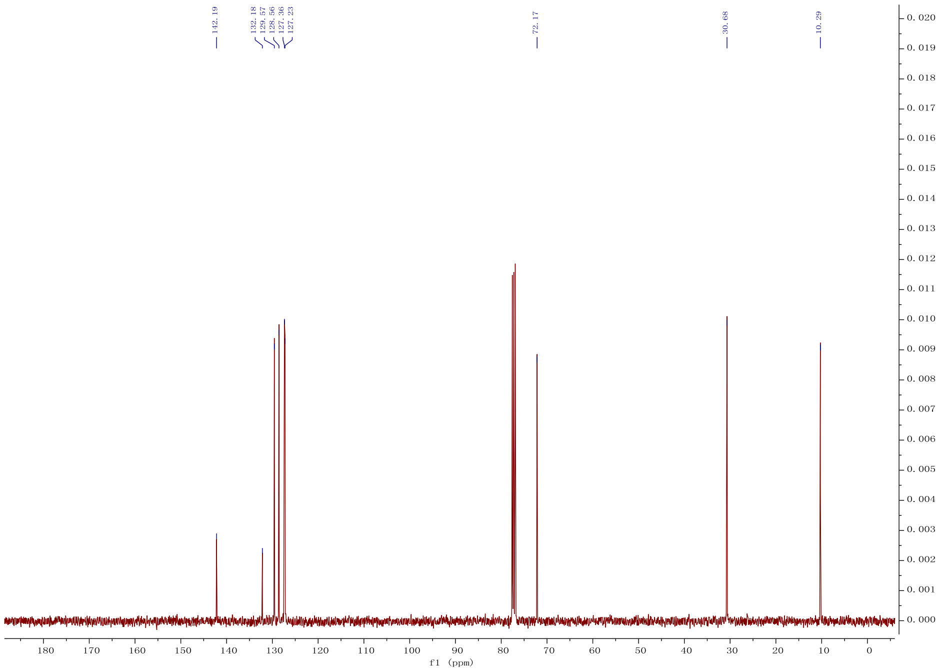

Figure S113. ^{13}C NMR spectrum of 23p, Related to Figure 6

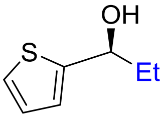

(*S*)-**23q**

HPLC: enantiomeric excess of **23q** (ee = 76%) was determined by high-performance liquid chromatography (HPLC) using a chiral stationary phase (ID column, flow rate = 0.8 mL/ min, eluent: hexane/ isopropanol = 98.5/1.5, 254 nm absorbance), retention times: minor enantiomer (t_R = 19.66 min), major enantiomer (t_R = 17.71 min).

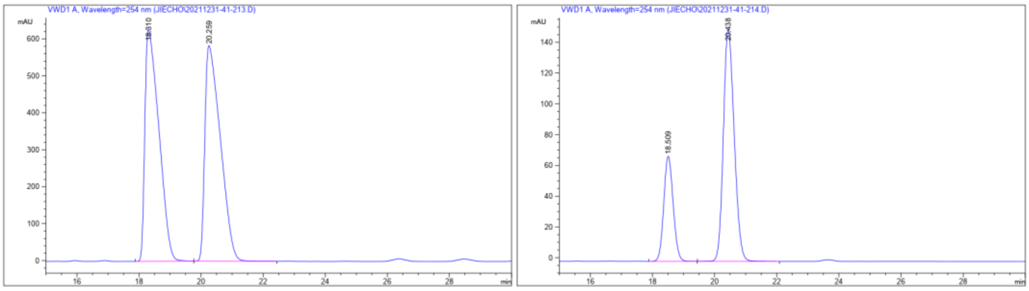

Figure S114. HPLC traces of 23q, Related to Figure 6

Signal 1: VWD1 A, Wavelength=254 nm

Peak #	RetTime [min]	Type	Width [min]	Area mAU *s	Height [mAU]	Area %
1	18.310	BB	0.5152	1.95771e4	627.77942	49.2278
2	20.259	BV	0.5616	2.01913e4	582.64435	50.7722
Totals :				3.97684e4	1210.42377	

Signal 1: VWD1 A, Wavelength=254 nm

Peak #	RetTime [min]	Type	Width [min]	Area mAU *s	Height [mAU]	Area %
1	18.509	BB	0.3265	1434.36047	68.31806	28.1964
2	20.438	BB	0.3757	3652.67505	150.85057	71.8036
Totals :				5087.03552	219.16863	

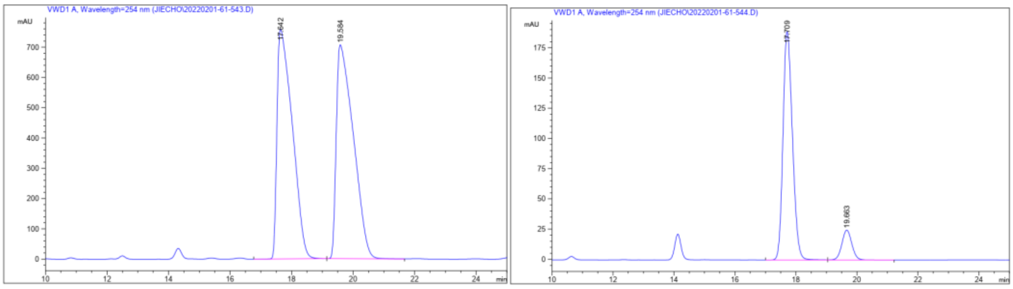

Figure S115. HPLC traces of 23q, Related to Figure 6

Signal 1: VWD1 A, Wavelength=254 nm

Peak #	RetTime [min]	Type	Width [min]	Area mAU *s	Height [mAU]	Area %
1	17.642	BB	0.5868	2.60694e4	759.45654	49.0072
2	19.584	BV	0.6458	2.71256e4	706.72418	50.9928
Totals :				5.31950e4	1466.18073	

Signal 1: VWD1 A, Wavelength=254 nm

Peak #	RetTime [min]	Type	Width [min]	Area mAU *s	Height [mAU]	Area %
1	17.709	BV	0.3344	4042.76880	188.71382	87.8339
2	19.663	VB	0.3516	559.97510	24.72568	12.1661
Totals :				4602.74390	213.43950	

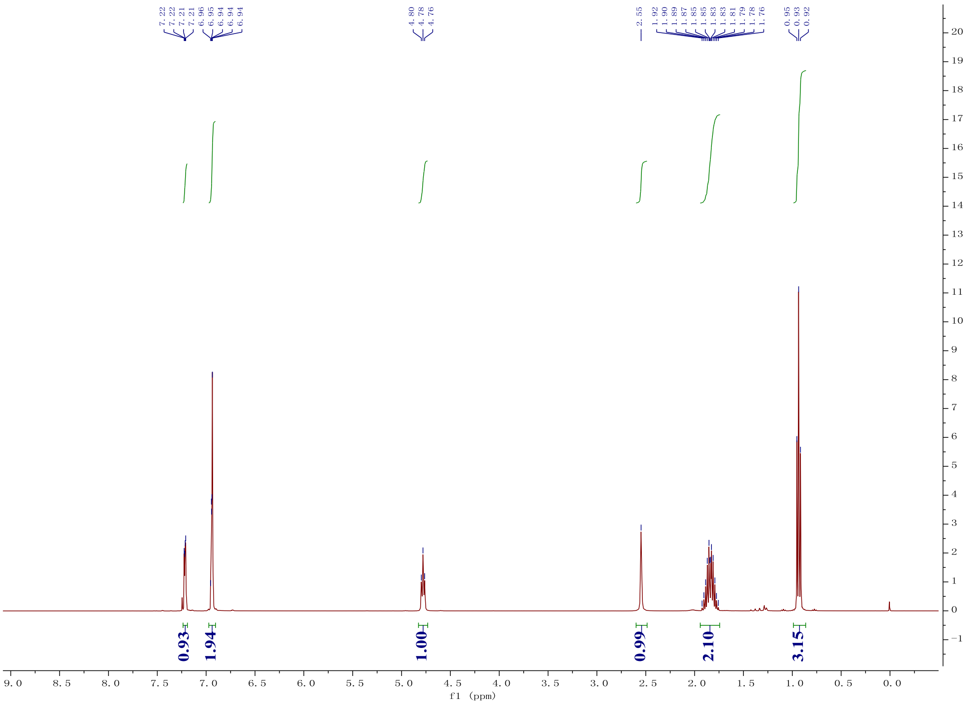

Figure S116. ^{1}H NMR spectrum of 23q, Related to Figure 6

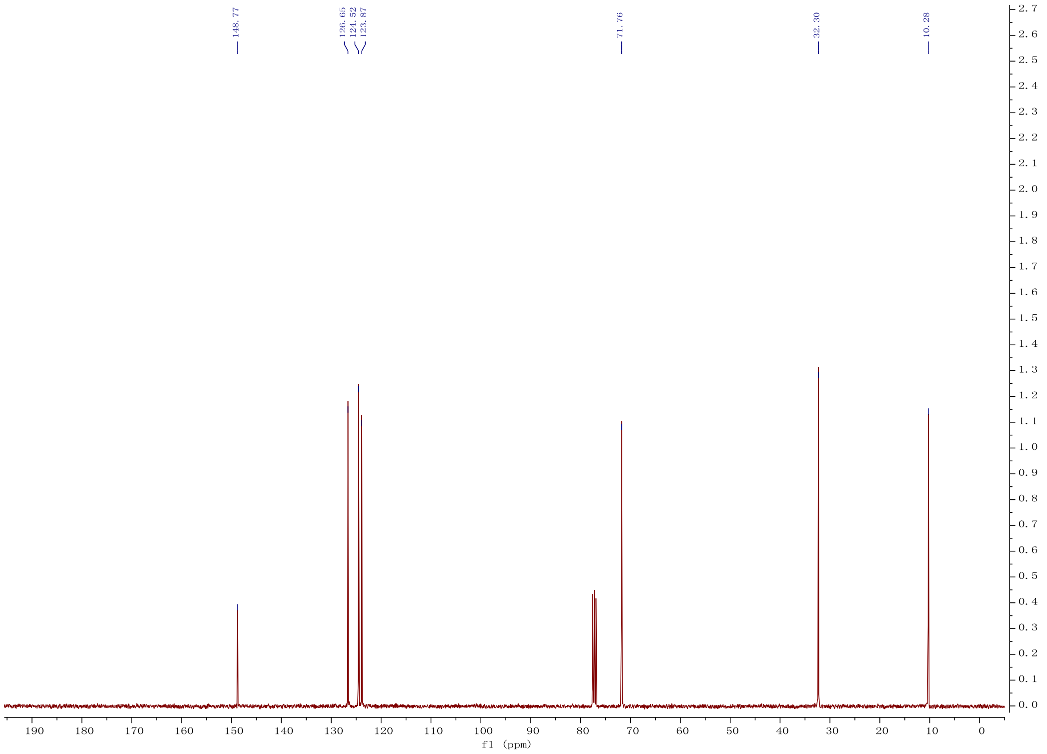

Figure S117. ^{13}C NMR spectrum of 23q, Related to Figure 6

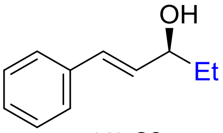

(*S*)-**23r**

HPLC: enantiomeric excess of **23r** (ee = 88%) was determined by high-performance liquid chromatography (HPLC) using a chiral stationary phase (AY-H column, flow rate = 0.8 mL/ min, eluent: hexane/ isopropanol = 98/2, 254 nm absorbance), retention times: minor enantiomer (t_R = 19.61 min), major enantiomer (t_R = 17.21 min).

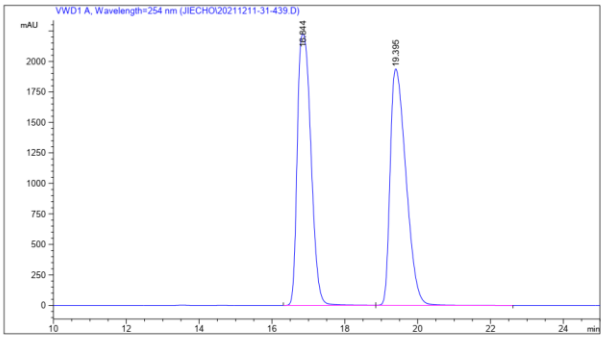

Figure S118. HPLC traces of 23r, Related to Figure 6

Signal 1: VWD1 A, Wavelength=254 nm

Peak #	RetTime [min]	Type	Width [min]	Area mAU *s	Height [mAU]	Area %
1	16.844	BB	0.4130	5.76424e4	2224.63599	49.7700
2	19.395	BB	0.4729	5.81753e4	1935.73657	50.2300

Totals : 1.15818e5 4160.37256

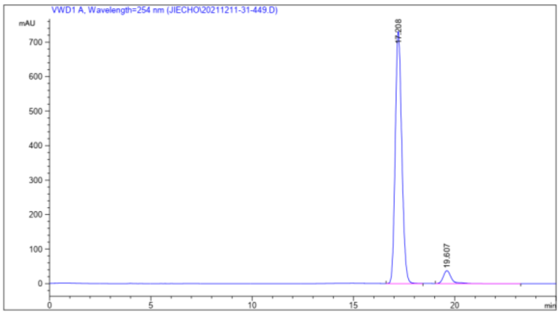

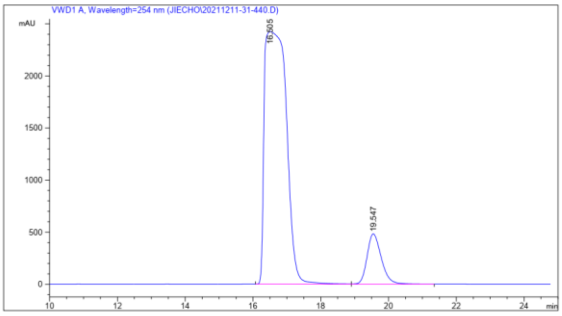

Figure S119. HPLC traces of 23r, Related to Figure 6

Signal 1: VWD1 A, Wavelength=254 nm

Peak #	RetTime [min]	Type	Width [min]	Area mAU *s	Height [mAU]	Area %
1	17.208	VV	0.3492	1.64049e4	730.96429	94.0016
2	19.607	VB	0.4244	1046.82532	37.53172	5.9984
Totals :				1.74517e4	768.49601	

Signal 1: VWD1 A, Wavelength=254 nm

Peak #	RetTime [min]	Type	Width [min]	Area mAU *s	Height [mAU]	Area %
1	16.505	BV	0.7337	1.09530e5	2425.09644	88.5893
2	19.547	VV	0.4486	1.41079e4	482.91675	11.4107
Totals :				1.23638e5	2908.01318	

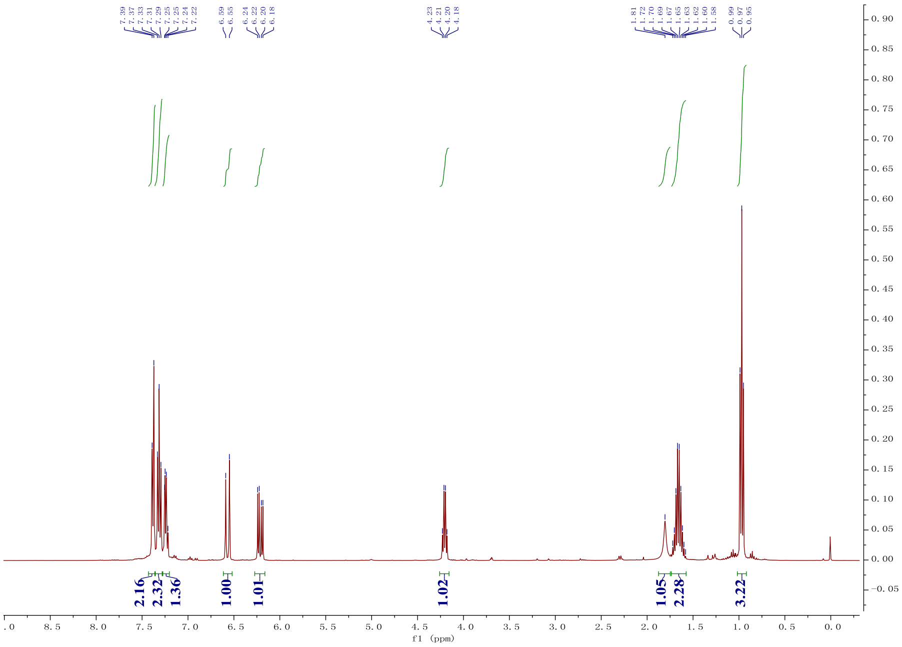

Figure S120. ^{1}H NMR spectrum of 23r, Related to Figure 6

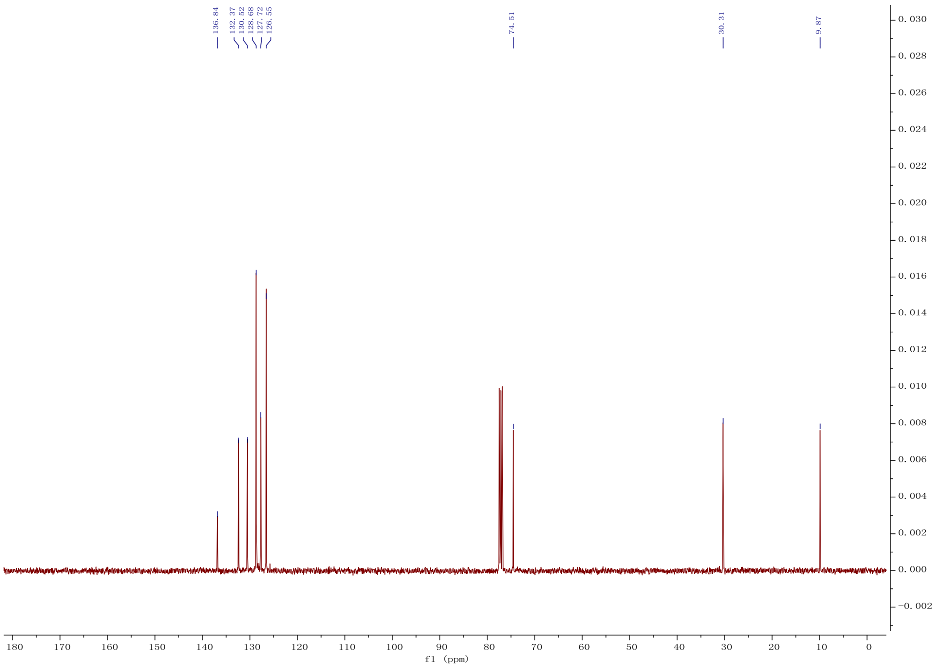

Figure S121. ^{13}C NMR spectrum of 23r, Related to Figure 6

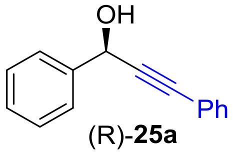

(R)-**25a**

HPLC: enantiomeric excess of **25a** (ee = 93%) was determined by high-performance liquid chromatography (HPLC) using a chiral stationary phase (OD-H column, flow rate = 1.0 mL/ min, eluent: hexane/ isopropanol = 80/20, 254 nm absorbance), retention times: major enantiomer (t_R = 13.84 min), minor enantiomer (t_R = 21.85 min).

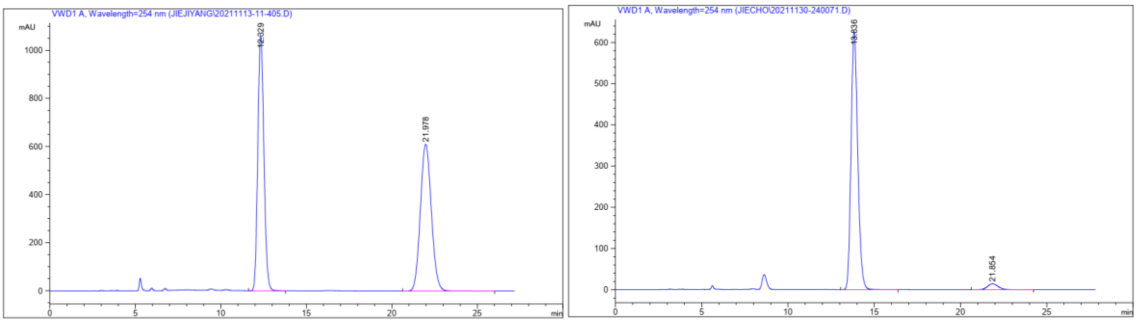

Figure S122. HPLC traces of 25a, Related to Figure 7

Signal 1: VWD1 A, Wavelength=254 nm

Peak #	RetTime [min]	Type	Width [min]	Area mAU *s	Height [mAU]	Area %
1	12.329	BV	0.3884	2.63350e4	1061.53503	49.0916
2	21.978	BB	0.6961	2.73097e4	611.08081	50.9084
Totals :				5.36447e4	1672.61584	

Signal 1: VWD1 A, Wavelength=254 nm

Peak #	RetTime [min]	Type	Width [min]	Area mAU *s	Height [mAU]	Area %
1	13.836	BV	0.4208	1.69148e4	624.58704	96.3982
2	21.854	BB	0.6826	631.99573	14.35209	3.6018
Totals :				1.75468e4	638.93913	

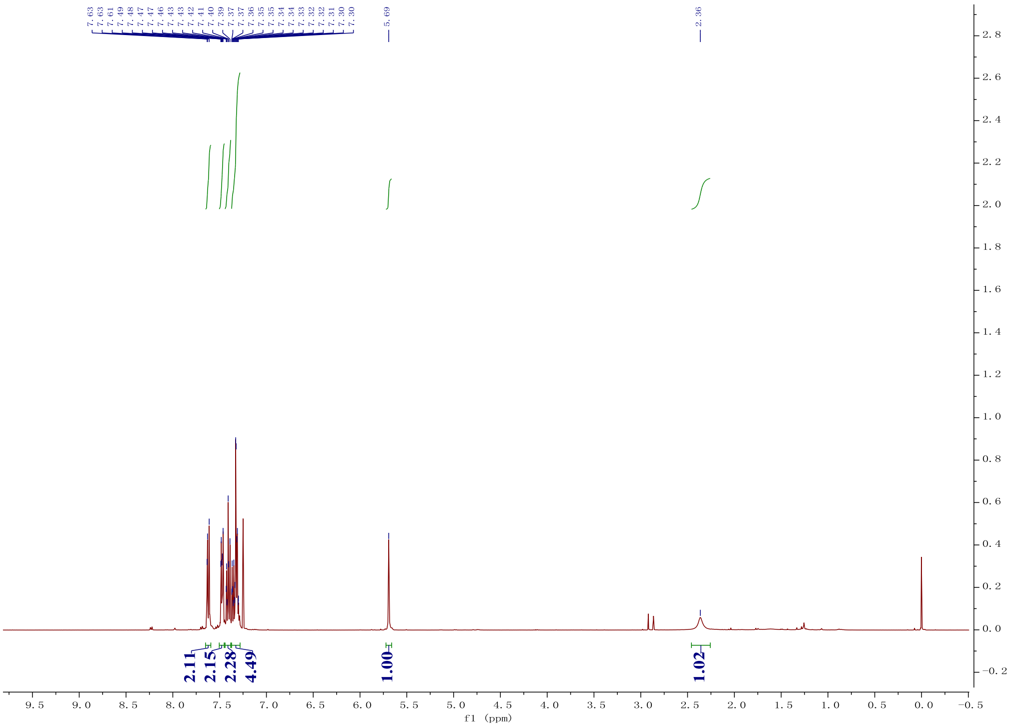

Figure S123. ^{1}H NMR spectrum of 25a, Related to Figure 7

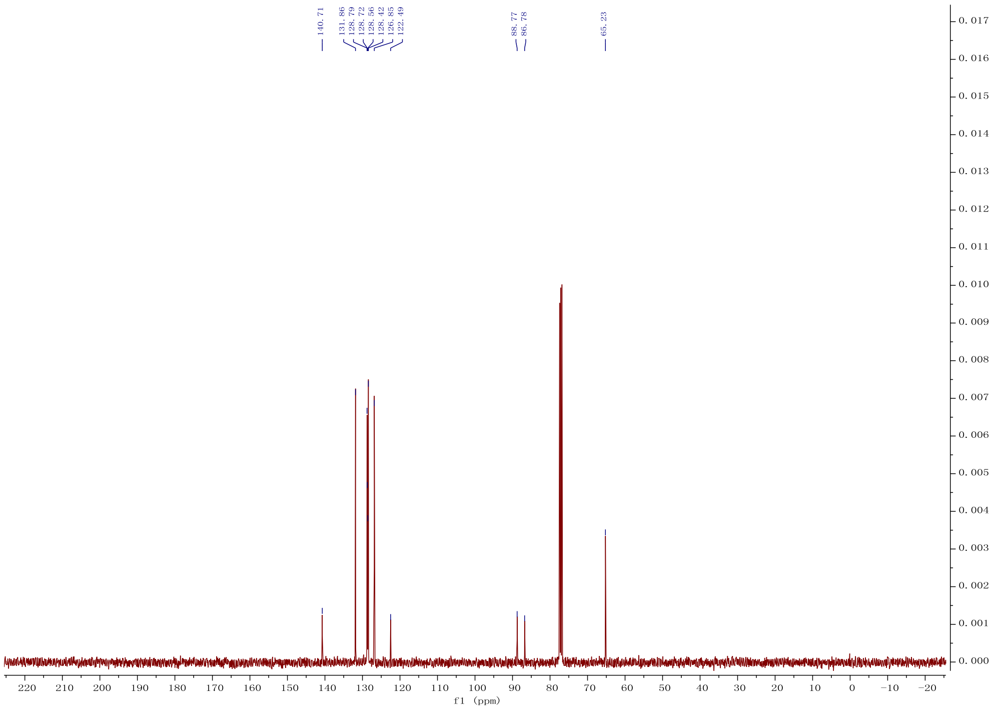

Figure S124. ^{13}C NMR spectrum of 25a, Related to Figure 7

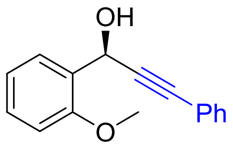

(R)-**25b**

HPLC: enantiomeric excess of **25b** (ee = 90%) was determined by high-performance liquid chromatography (HPLC) using a chiral stationary phase (OD-H column, flow rate = 1.0 mL/ min, eluent: hexane/ isopropanol = 90/10, 254 nm absorbance), retention times: major enantiomer (t_R = 15.79 min), minor enantiomer (t_R = 19.34 min).

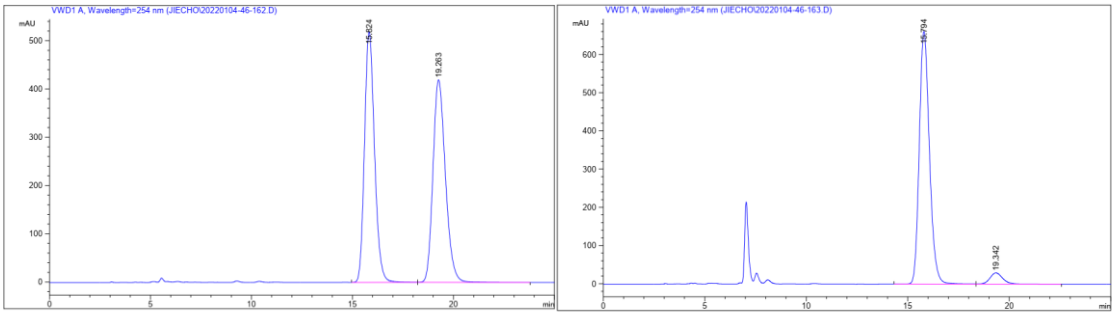

Figure S125. HPLC traces of 25b, Related to Figure 7

Signal 1: VWD1 A, Wavelength=254 nm

Peak #	RetTime [min]	Type	Width [min]	Area mAU *s	Height [mAU]	Area %
1	15.824	VB	0.5327	1.78817e4	519.42993	49.8989
2	19.263	BB	0.6624	1.79542e4	419.46136	50.1011
Totals :				3.58360e4	938.89130	

Signal 1: VWD1 A, Wavelength=254 nm

Peak #	RetTime [min]	Type	Width [min]	Area mAU *s	Height [mAU]	Area %
1	15.794	BV	0.5353	2.29046e4	661.06927	94.7783
2	19.342	VB	0.6659	1261.90564	29.11008	5.2217
Totals :				2.41665e4	690.17935	

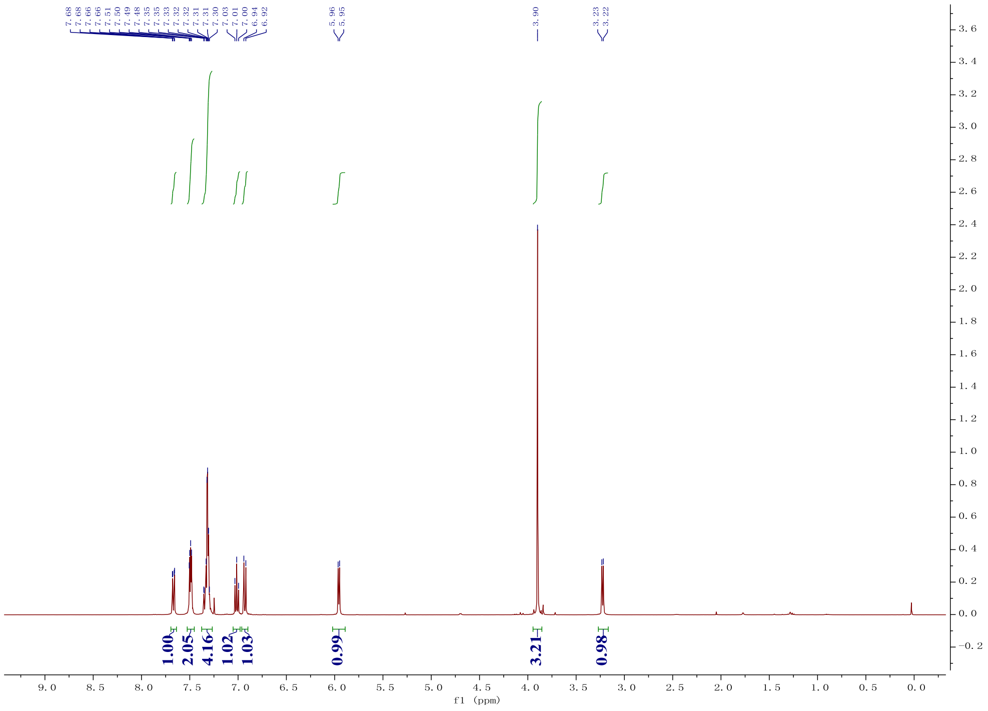

Figure S126. ^{1}H NMR spectrum of 25b, Related to Figure 7

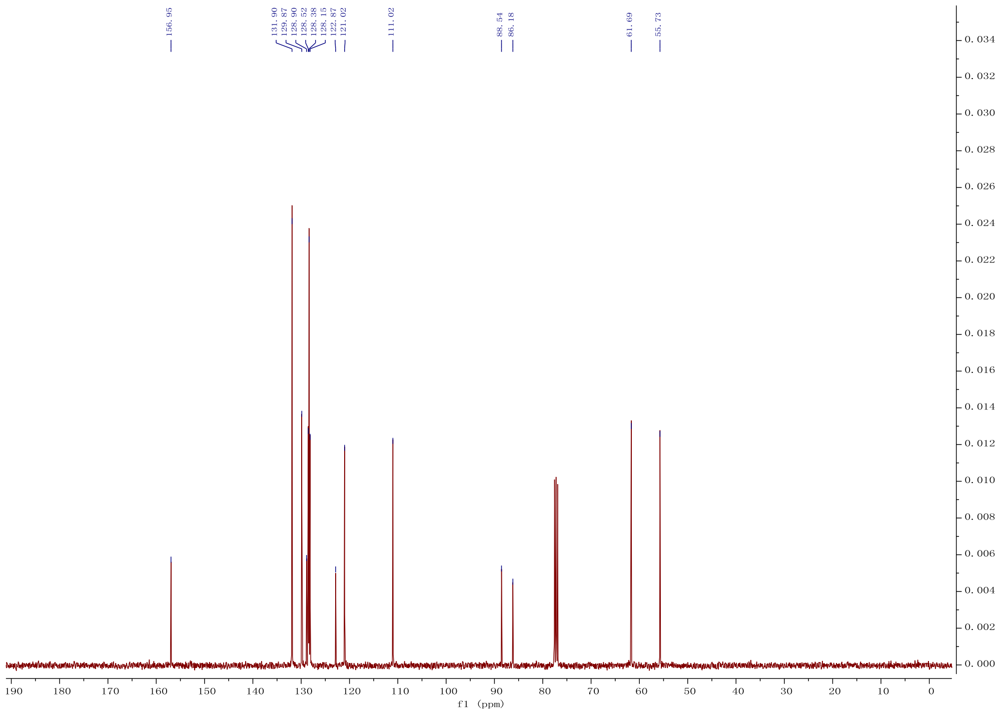

Figure S127. ^{13}C NMR spectrum of 25b, Related to Figure 7

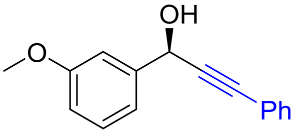

(R)-**25c**

HPLC: enantiomeric excess of **25c** (ee = 97%) was determined by high-performance liquid chromatography (HPLC) using a chiral stationary phase (OD-H column, flow rate = 1.0 mL/ min, eluent: hexane/ isopropanol = 80/20, 254 nm absorbance), retention times: major enantiomer (t_R = 9.89 min), minor enantiomer (t_R = 12.83 min).

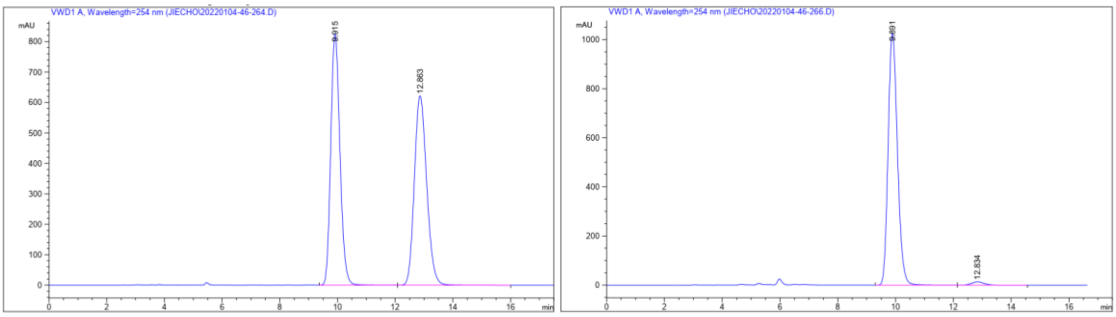

Figure S128. HPLC traces of 25c, Related to Figure 7

Signal 1: VWD1 A, Wavelength=254 nm

Peak #	RetTime [min]	Type	Width [min]	Area mAU *s	Height [mAU]	Area %
1	9.915	VB	0.3434	1.82422e4	826.58545	49.6864
2	12.863	BB	0.4628	1.84725e4	622.26093	50.3136
Totals :				3.67147e4	1448.84637	

Signal 1: VWD1 A, Wavelength=254 nm

Peak #	RetTime [min]	Type	Width [min]	Area mAU *s	Height [mAU]	Area %
1	9.891	BV	0.3488	2.30078e4	1026.60767	98.3365
2	12.834	VB	0.4582	389.20621	13.17548	1.6635
Totals :				2.33970e4	1039.78314	

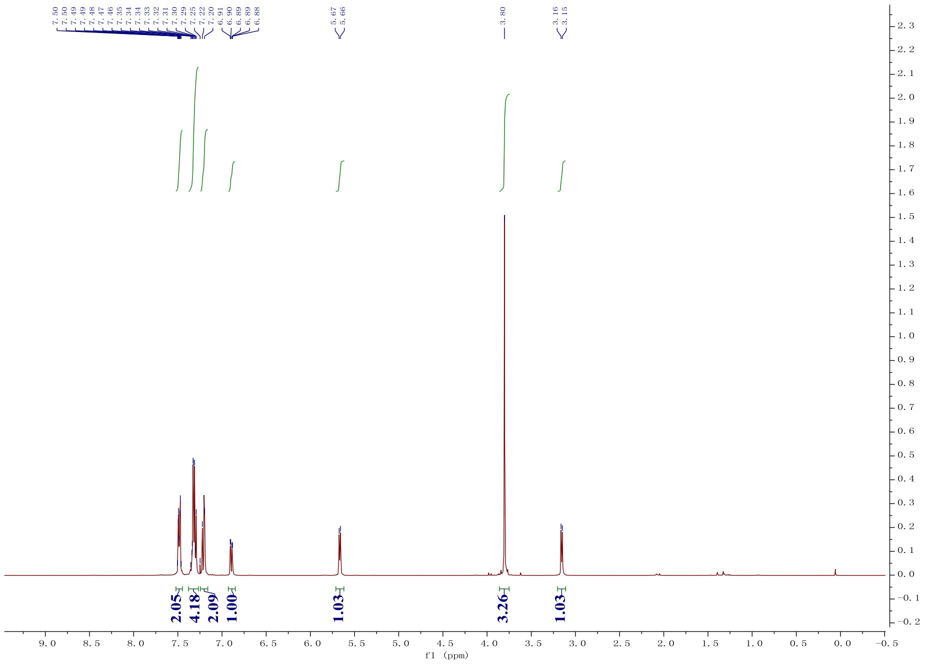

Figure S129. ^{1}H NMR spectrum of 25c, Related to Figure 7

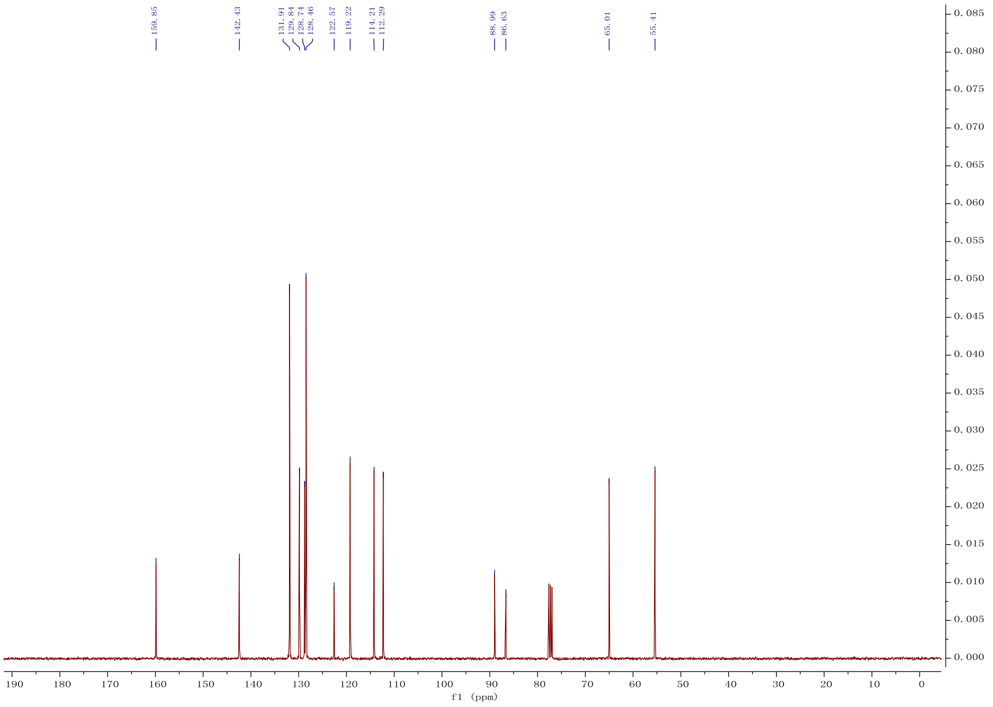

Figure S130. ^{13}C NMR spectrum of 25c, Related to Figure 7

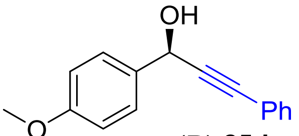

(R)-**25d**

HPLC: enantiomeric excess of **25d** (ee = 94%) was determined by high-performance liquid chromatography (HPLC) using a chiral stationary phase (OD-H column, flow rate = 1.0 mL/ min, eluent: hexane/ isopropanol = 80/20, 254 nm absorbance), retention times: major enantiomer (t_R = 9.15 min), minor enantiomer (t_R = 13.86 min).

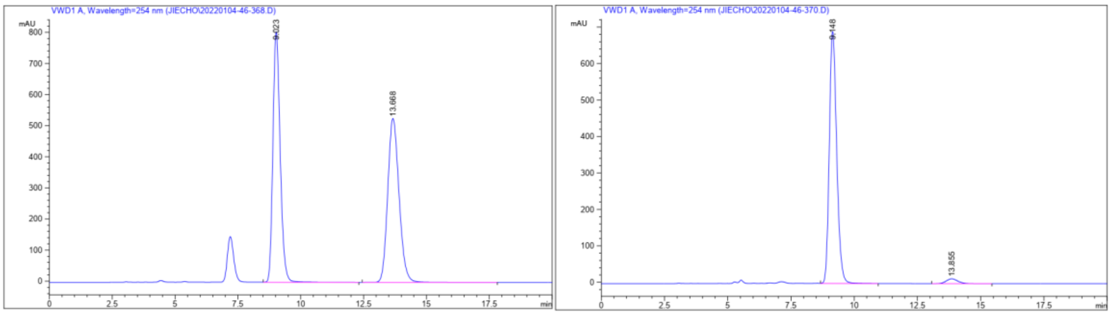

Figure S131. HPLC traces of 25d, Related to Figure 7

Signal 1: VWD1 A, Wavelength=254 nm

Peak #	RetTime [min]	Type	Width [min]	Area mAU *s	Height [mAU]	Area %
1	9.023	VB	0.3114	1.62527e4	804.63605	49.7403
2	13.668	BB	0.4832	1.64224e4	526.60986	50.2597
Totals :				3.26751e4	1331.24591	

Signal 1: VWD1 A, Wavelength=254 nm

Peak #	RetTime [min]	Type	Width [min]	Area mAU *s	Height [mAU]	Area %
1	9.148	VB	0.3165	1.40883e4	690.93719	97.1437
2	13.855	BB	0.4899	414.22870	13.04365	2.8563
Totals :				1.45025e4	703.98085	

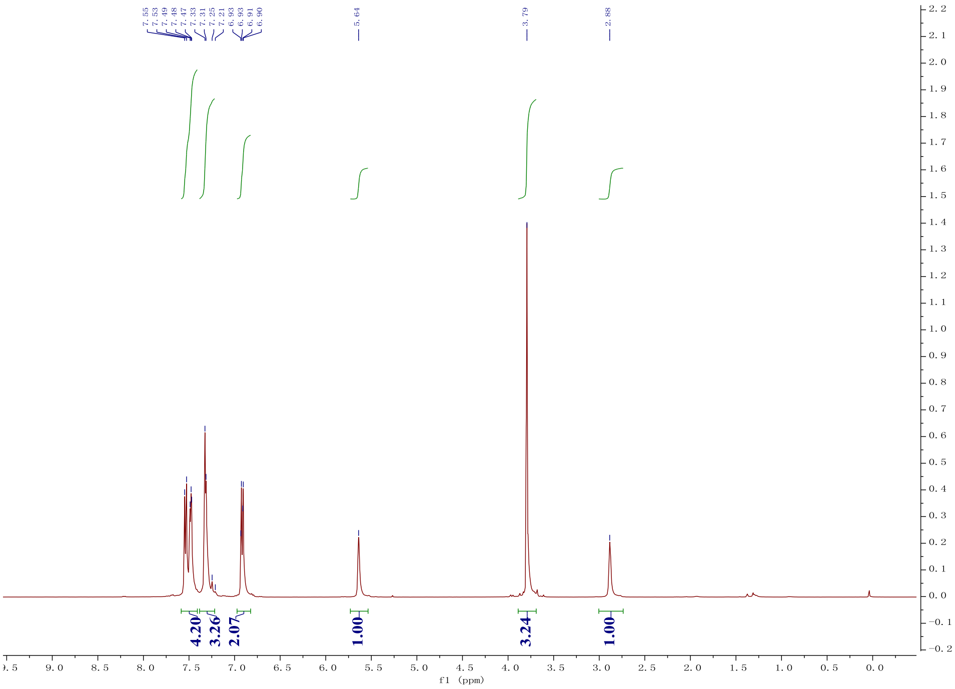

Figure S132. ^{1}H NMR spectrum of 25d, Related to Figure 7

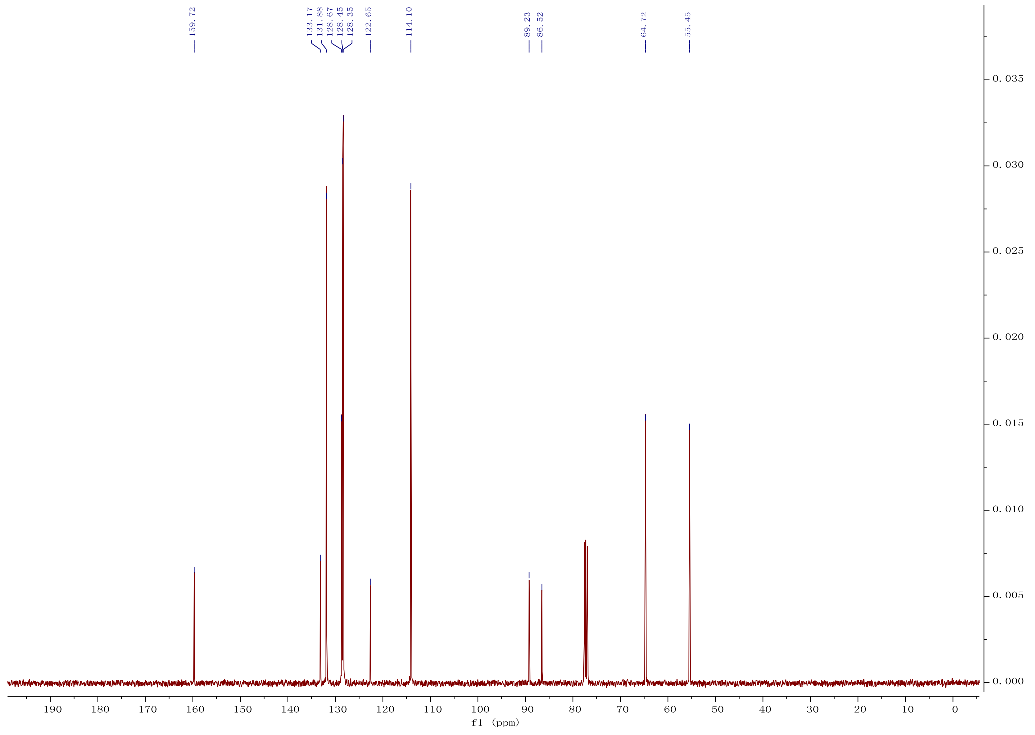

Figure S133. ^{13}C NMR spectrum of 25d, Related to Figure 7

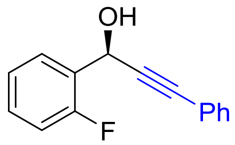

(R)-**25e**

HPLC: enantiomeric excess of **25e** (ee = 89%) was determined by high-performance liquid chromatography (HPLC) using a chiral stationary phase (OD-H column, flow rate = 1.0 mL/ min, eluent: hexane/ isopropanol = 80/20, 254 nm absorbance), retention times: major enantiomer (t_R = 5.87 min), minor enantiomer (t_R = 6.92 min).

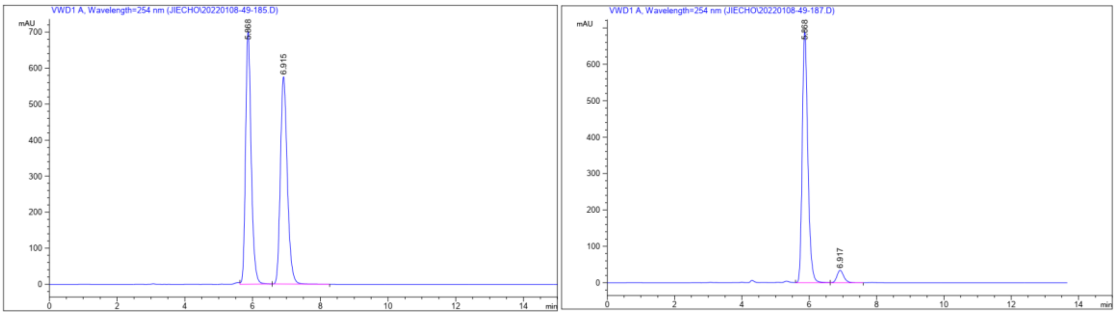

Figure S134. HPLC traces of 25e, Related to Figure 7

Signal 1: VWD1 A, Wavelength=254 nm

Peak #	RetTime [min]	Type	Width [min]	Area mAU *s	Height [mAU]	Area %
1	5.868	VB	0.1774	8125.37500	701.20819	49.8797
2	6.915	BB	0.2179	8164.55908	575.71576	50.1203
Totals :				1.62899e4	1276.92395	

Signal 1: VWD1 A, Wavelength=254 nm

Peak #	RetTime [min]	Type	Width [min]	Area mAU *s	Height [mAU]	Area %
1	5.868	VV	0.1770	7957.76904	688.66541	94.2775
2	6.917	VB	0.2171	483.02676	34.22313	5.7225
Totals :				8440.79581	722.88854	

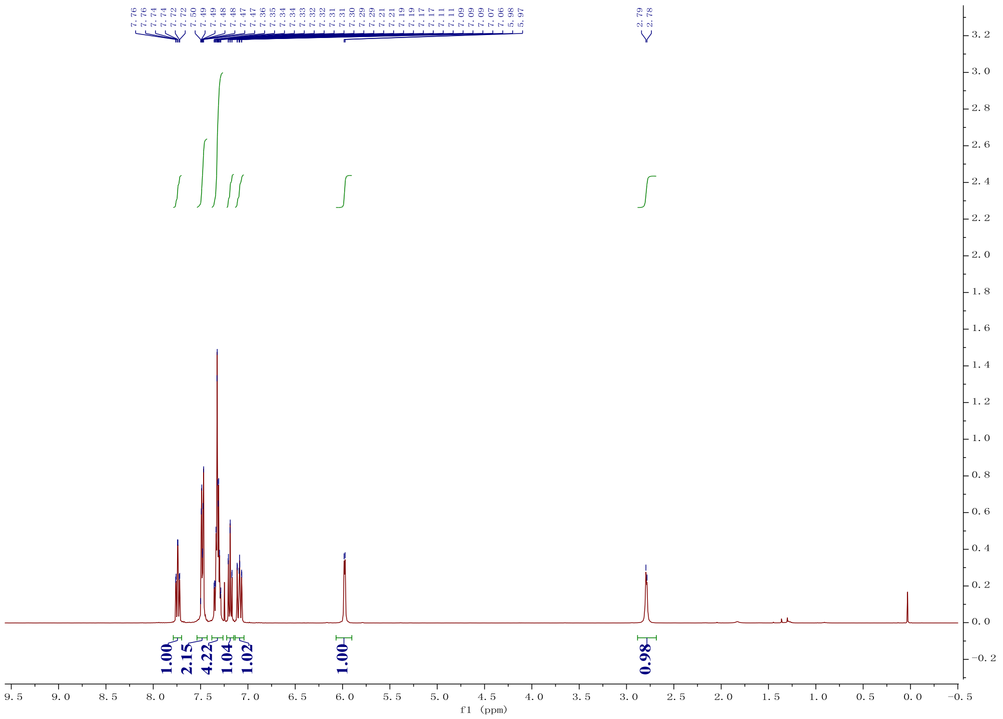

Figure S135. ^{1}H NMR spectrum of 25e, Related to Figure 7

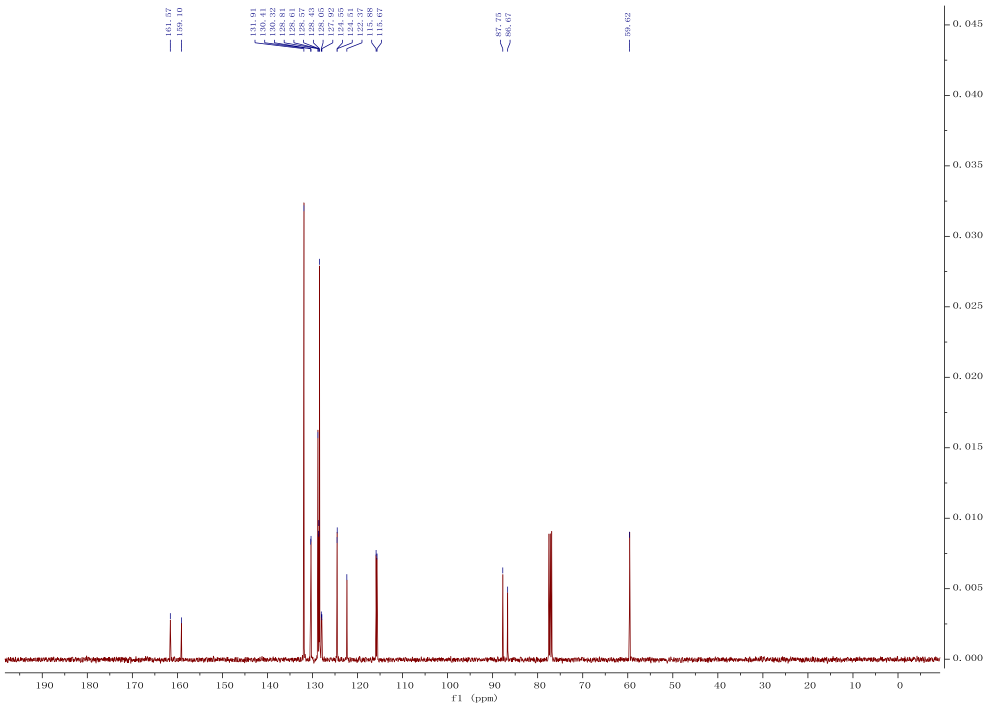

Figure S136. ^{13}C NMR spectrum of 25e, Related to Figure 7

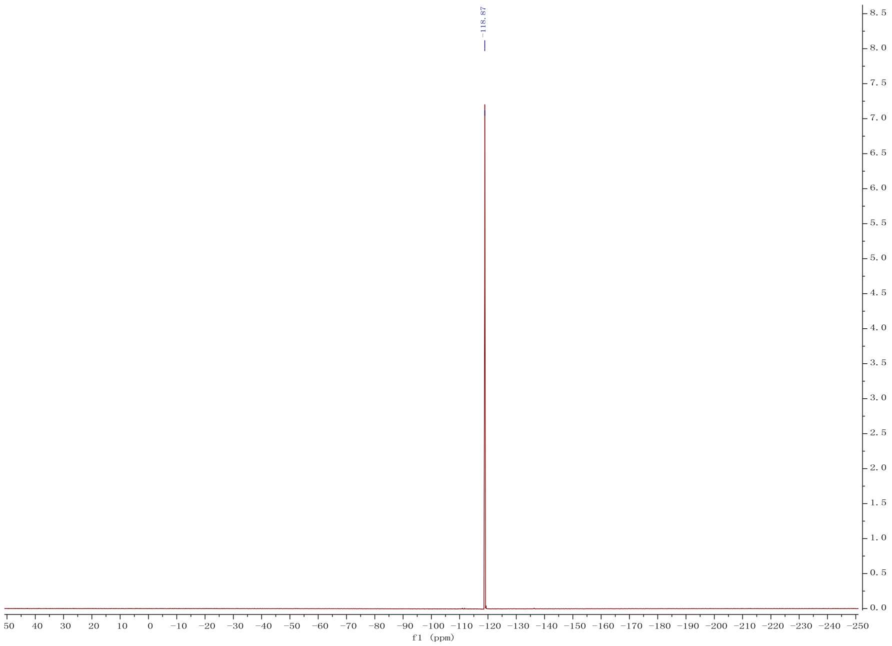

Figure S137. ^{19}F NMR spectrum of 25e, Related to Figure 7

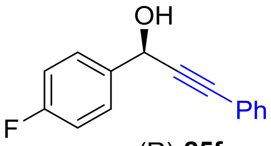

(R)-**25f**

HPLC: enantiomeric excess of **25f** (ee = 91%) was determined by high-performance liquid chromatography (HPLC) using a chiral stationary phase (OD-H column, flow rate = 1.0 mL/ min, eluent: hexane/ isopropanol = 80/20, 254 nm absorbance), retention times: minor enantiomer (t_R = 11.74 min), major enantiomer (t_R = 6.04 min).

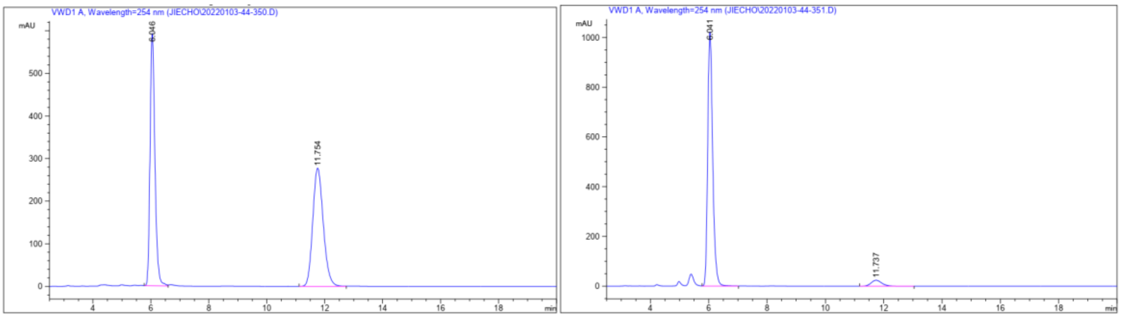

Figure S138. HPLC traces of 25f, Related to Figure 7

Signal 1: VWD1 A, Wavelength=254 nm

Peak #	RetTime [min]	Type	Width [min]	Area mAU *s	Height [mAU]	Area %
1	6.046	BV	0.1794	6883.90234	591.75671	49.6278
2	11.754	BV	0.3900	6987.16455	277.34381	50.3722

Totals : 1.38711e4 869.10052

Signal 1: VWD1 A, Wavelength=254 nm

Peak #	RetTime [min]	Type	Width [min]	Area mAU *s	Height [mAU]	Area %
1	6.041	VV	0.1826	1.21773e4	1022.45477	95.3368
2	11.737	BB	0.3834	595.62238	23.94164	4.6632

Totals : 1.27729e4 1046.39641

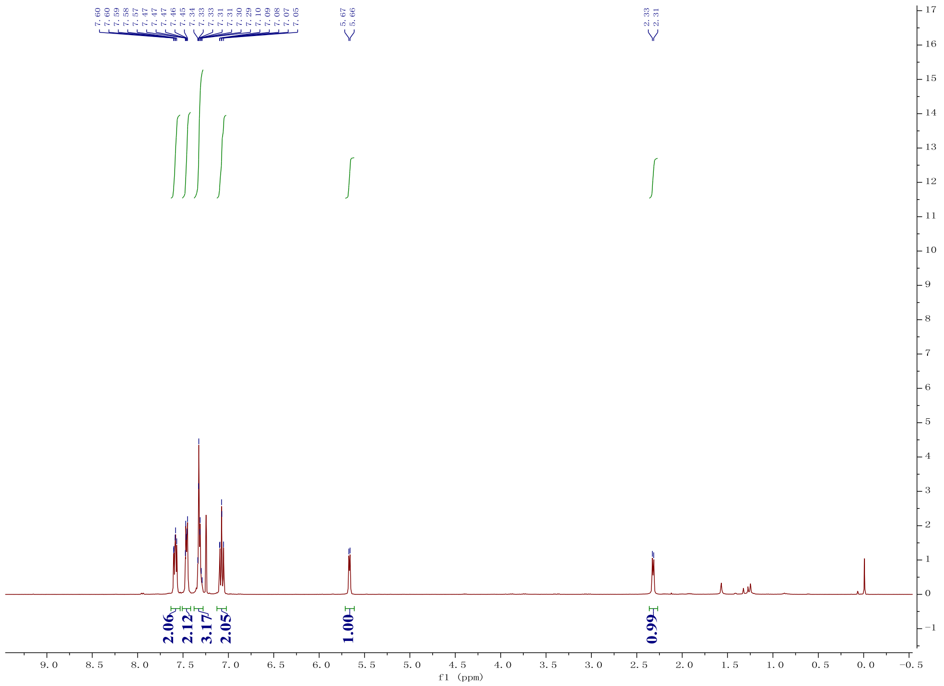

Figure S139. **^{1}H NMR spectrum of 25f, Related to Figure 7**

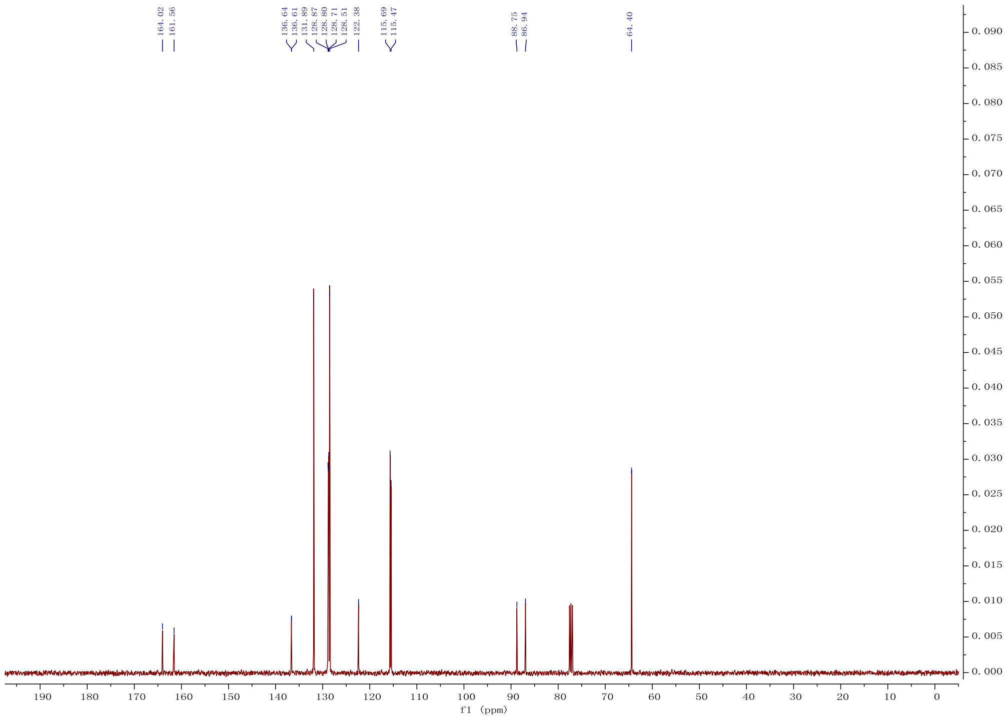

Figure S140. **^{13}C NMR spectrum of 25f, Related to Figure 7**

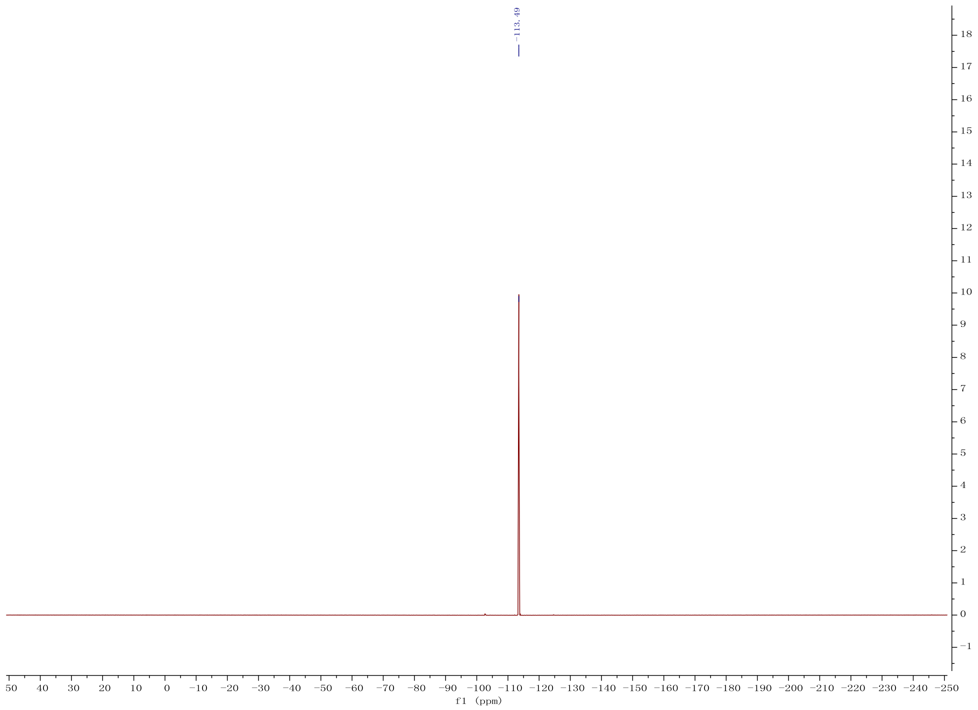

Figure S141. ^{19}F NMR spectrum of 25f, Related to Figure 7

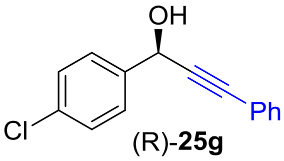

(R)-**25g**

HPLC: enantiomeric excess of **25g** (ee = 93%) was determined by high-performance liquid chromatography (HPLC) using a chiral stationary phase (OD-H column, flow rate = 1.0 mL/ min, eluent: hexane/ isopropanol = 80/20, 254 nm absorbance), retention times: major enantiomer (t_R = 6.41 min), minor enantiomer (t_R = 14.83 min).

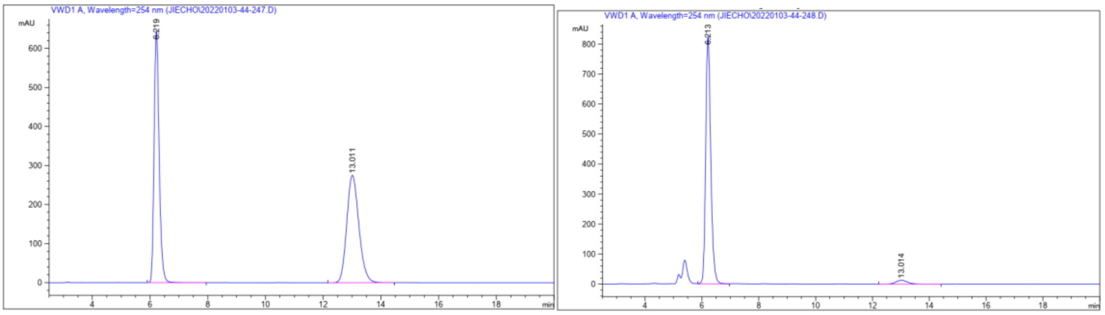

Figure S142. HPLC traces of 25g, Related to Figure 7

Signal 1: VWD1 A, Wavelength=254 nm

Peak #	RetTime [min]	Type	Width [min]	Area mAU *s	Height [mAU]	Area %
1	6.219	BB	0.1878	7879.58838	644.23895	49.5521
2	13.011	BB	0.4507	8022.04492	275.26920	50.4479
Totals :				1.59016e4	919.50815	

Signal 1: VWD1 A, Wavelength=254 nm

Peak #	RetTime [min]	Type	Width [min]	Area mAU *s	Height [mAU]	Area %
1	6.213	VV	0.1902	1.02558e4	824.87305	96.5139
2	13.014	BB	0.4454	370.43948	12.79881	3.4861
Totals :				1.06262e4	837.67185	

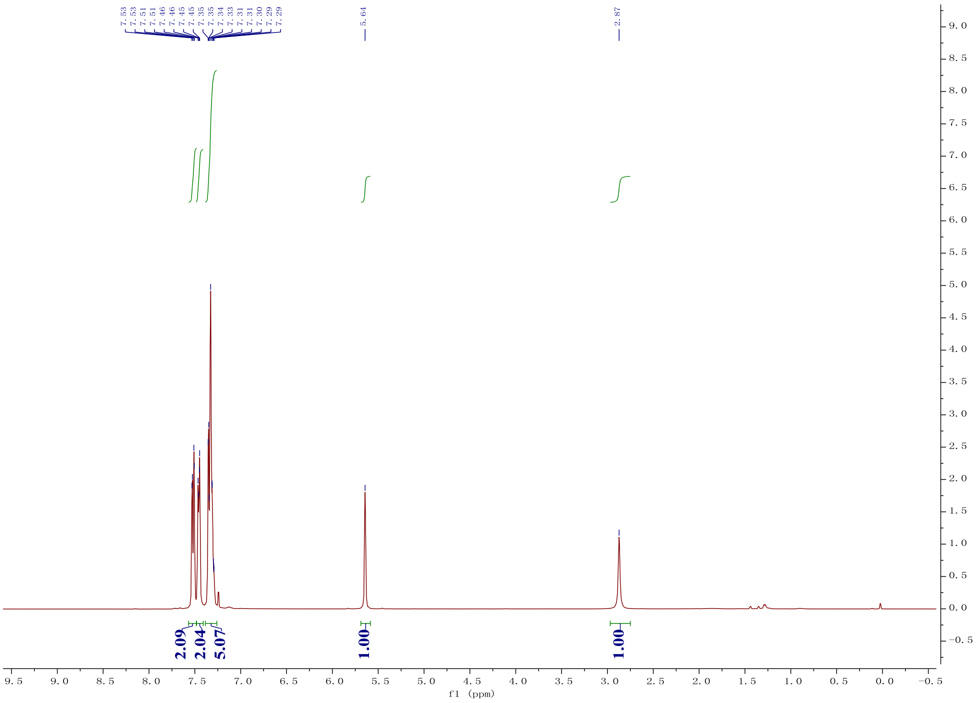

Figure S143. ^{1}H NMR spectrum of 25g, Related to Figure 7

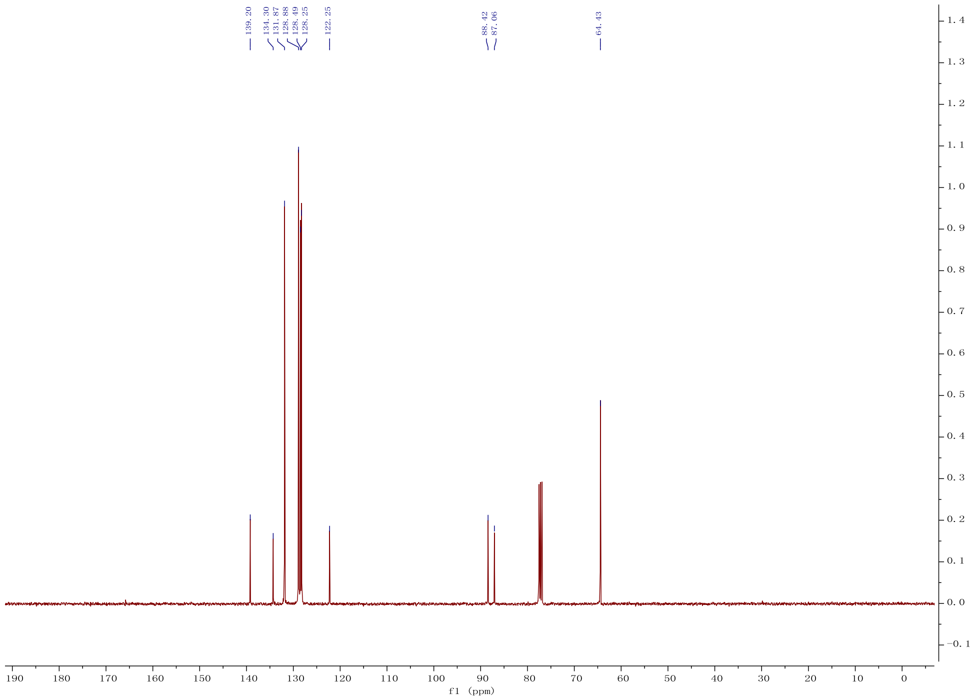

Figure S144. ^{13}C NMR spectrum of 25g, Related to Figure 7

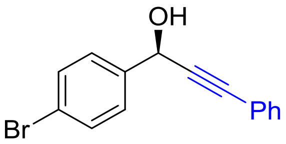

(R)-**25h**

HPLC: enantiomeric excess of **25h** (ee = 94%) was determined by high-performance liquid chromatography (HPLC) using a chiral stationary phase (OD-H column, flow rate = 1.0 mL/ min, eluent: hexane/ isopropanol = 80/20, 254 nm absorbance), retention times: major enantiomer (t_R = 6.45 min), minor enantiomer (t_R = 14.07 min).

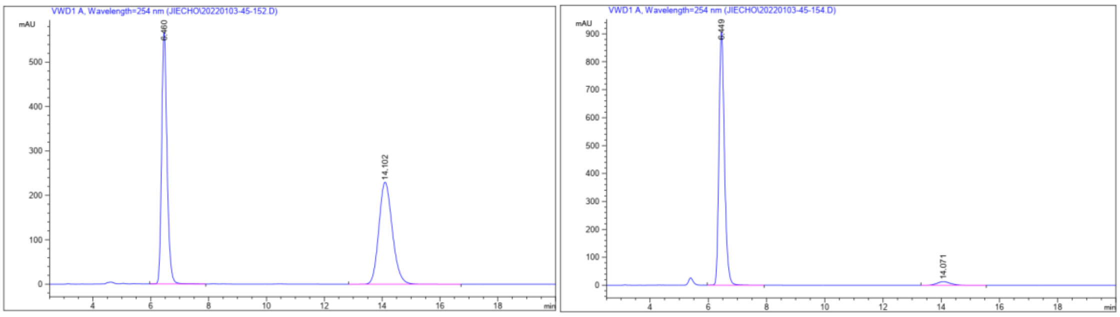

Figure S145. HPLC traces of 25h, Related to Figure 7

Signal 1: VWD1 A, Wavelength=254 nm

Peak #	RetTime [min]	Type	Width [min]	Area mAU *s	Height [mAU]	Area %
1	6.460	VB	0.1992	7334.90674	566.02692	49.7396
2	14.102	BB	0.4988	7411.71191	229.68924	50.2604
Totals :				1.47466e4	795.71616	

Signal 1: VWD1 A, Wavelength=254 nm

Peak #	RetTime [min]	Type	Width [min]	Area mAU *s	Height [mAU]	Area %
1	6.449	VV	0.2022	1.18749e4	907.28491	96.6934
2	14.071	BB	0.4952	406.08936	12.70429	3.3066
Totals :				1.22810e4	919.98921	

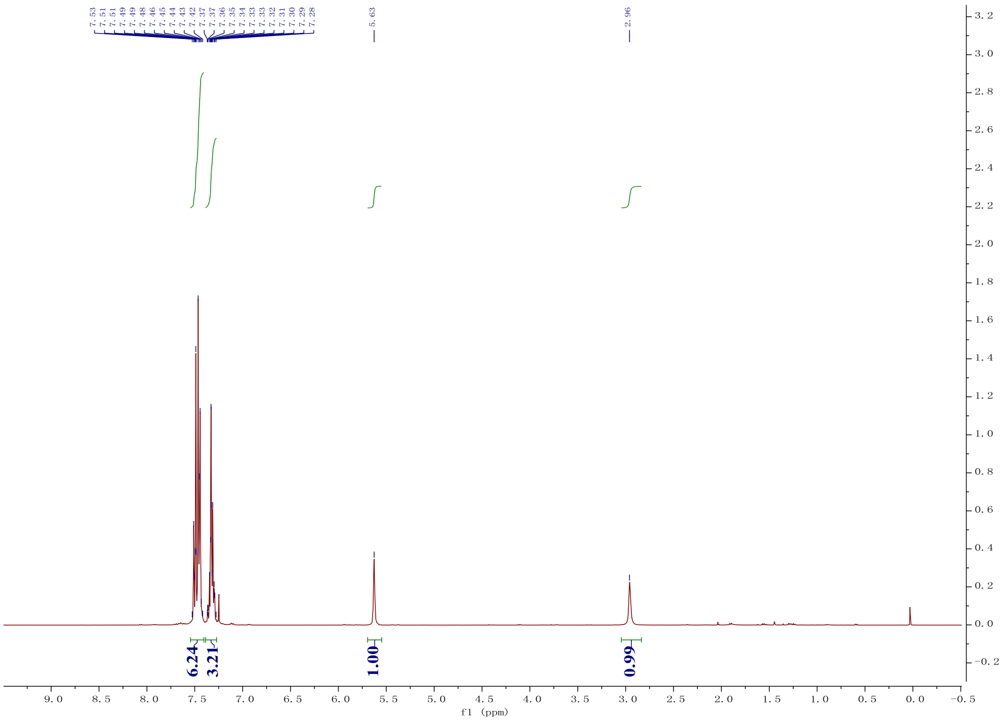

Figure S146. ^{1}H NMR spectrum of 25h, Related to Figure 7

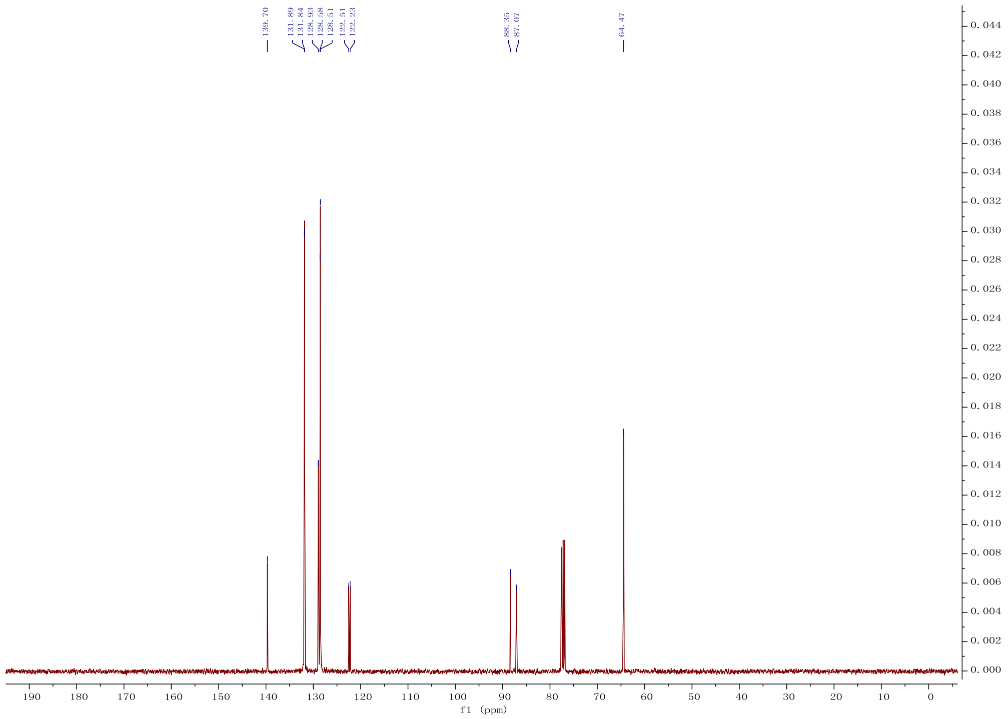

Figure S147. ^{13}C NMR spectrum of 25h, Related to Figure 7

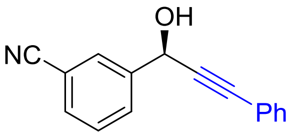

(R)-**25i**

HPLC: enantiomeric excess of **25i** (ee = 94%) was determined by high-performance liquid chromatography (HPLC) using a chiral stationary phase (OD-H column, flow rate = 1.0 mL/ min, eluent: hexane/ isopropanol = 80/20, 254 nm absorbance), retention times: major enantiomer (t_R = 8.10 min), minor enantiomer (t_R = 39.84 min).

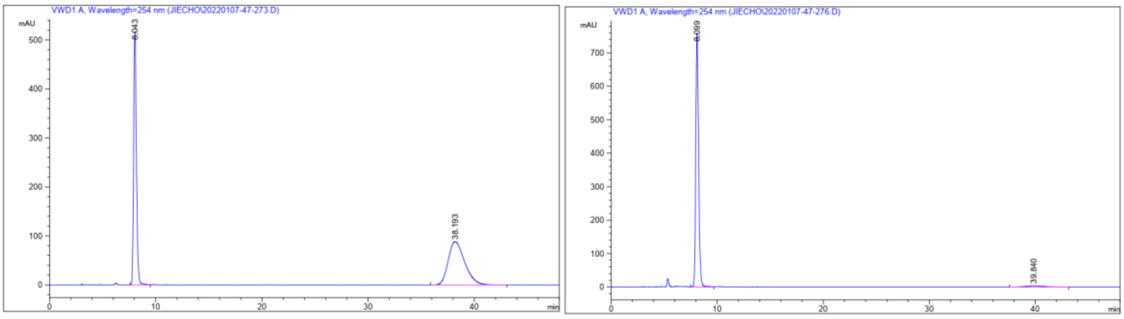

Figure S148. HPLC traces of 25i, Related to Figure 7

Signal 1: VWD1 A, Wavelength=254 nm

Peak #	RetTime [min]	Type	Width [min]	Area mAU *s	Height [mAU]	Area %
1	8.043	BV	0.2780	9289.15527	517.03033	49.4777
2	38.193	BB	1.6141	9485.27148	88.31342	50.5223
Totals :				1.87744e4	605.34376	

Signal 1: VWD1 A, Wavelength=254 nm

Peak #	RetTime [min]	Type	Width [min]	Area mAU *s	Height [mAU]	Area %
1	8.099	BV	0.2823	1.39957e4	758.36975	96.8890
2	39.840	BB	1.3285	449.38931	4.04404	3.1110
Totals :				1.44451e4	762.41379	

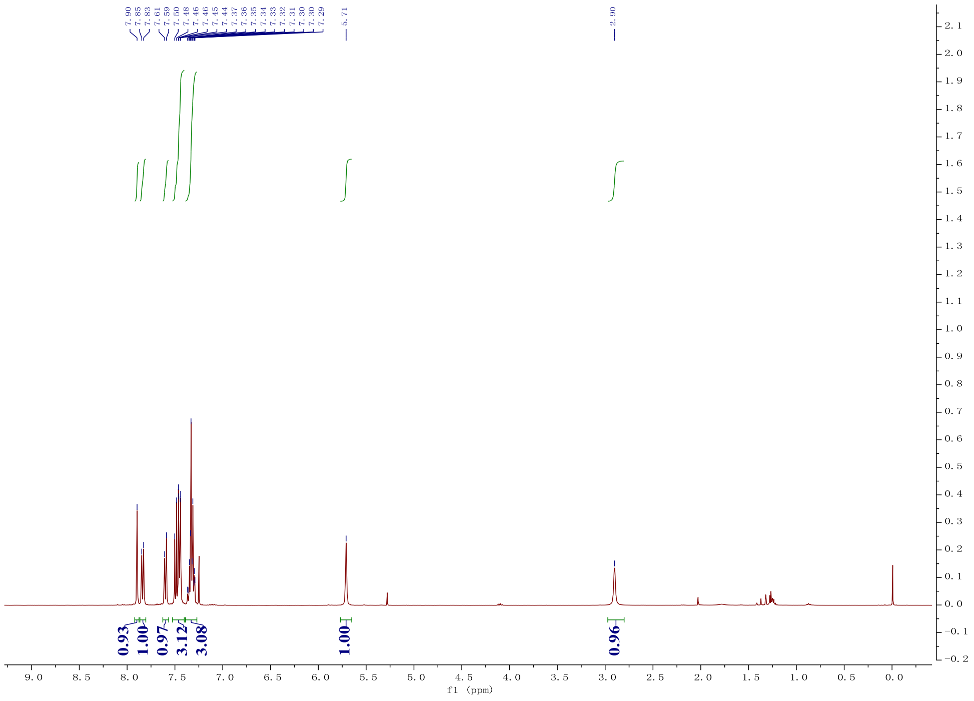

Figure S149. ^{1}H NMR spectrum of 25i, Related to Figure 7

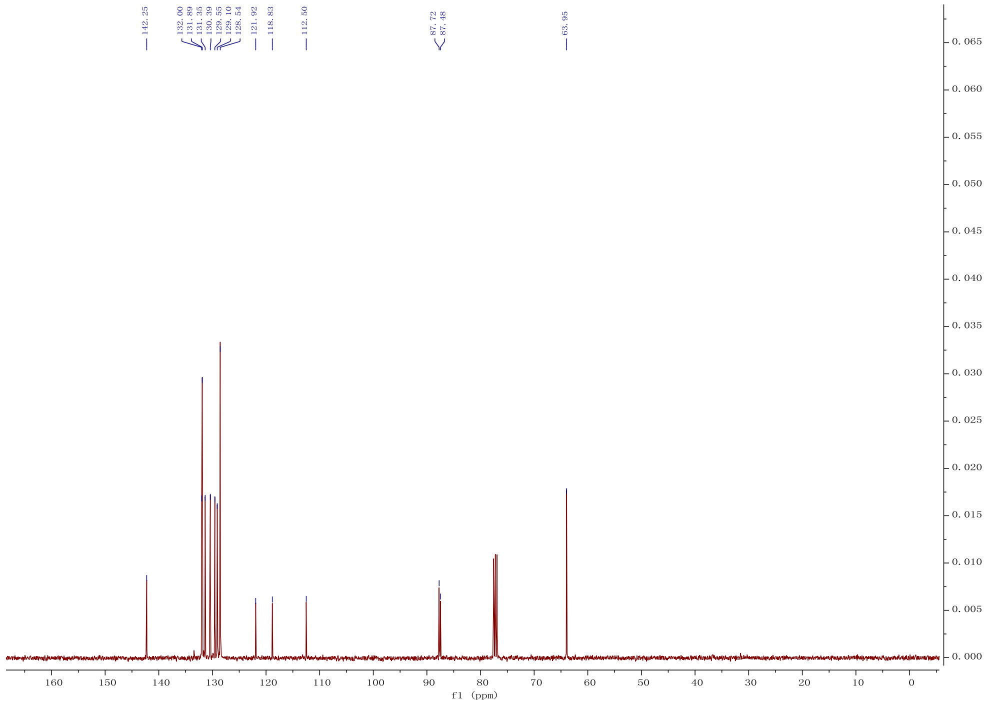

Figure S150. ^{13}C NMR spectrum of 25i, Related to Figure 7

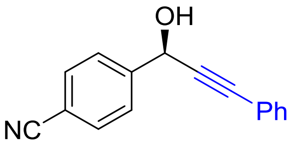

(R)-**25j**

HPLC: enantiomeric excess of **25j** (ee = 93%) was determined by high-performance liquid chromatography (HPLC) using a chiral stationary phase (OD-H column, flow rate = 1.0 mL/ min, eluent: hexane/ isopropanol = 80/20, 254 nm absorbance), retention times: major enantiomer (t_R = 9.15 min), minor enantiomer (t_R = 21.22 min).

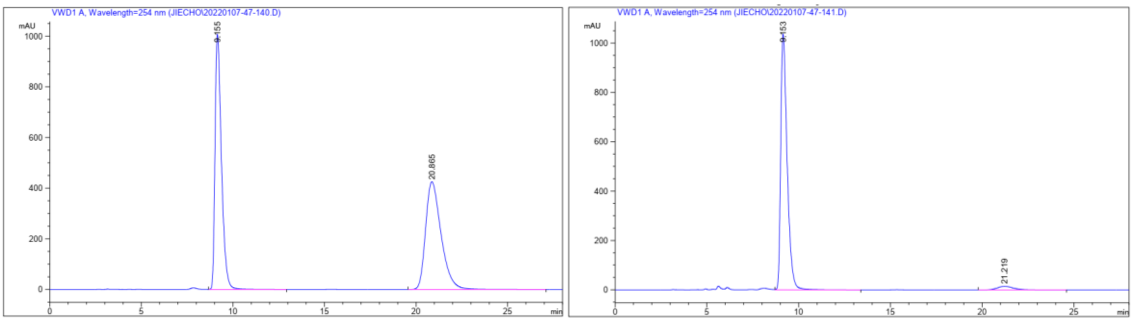

Figure S151. HPLC traces of 25j, Related to Figure 7

Signal 1: VWD1 A, Wavelength=254 nm

Peak #	RetTime [min]	Type	Width [min]	Area mAU *s	Height [mAU]	Area %
1	9.155	VB	0.3677	2.44719e4	1008.05927	49.4920
2	20.865	BB	0.8948	2.49743e4	425.26822	50.5080
Totals :				4.94462e4	1433.32748	

Signal 1: VWD1 A, Wavelength=254 nm

Peak #	RetTime [min]	Type	Width [min]	Area mAU *s	Height [mAU]	Area %
1	9.153	VB	0.3689	2.52481e4	1035.69177	96.6068
2	21.219	BB	0.9280	886.80157	14.57918	3.3932
Totals :				2.61349e4	1050.27095	

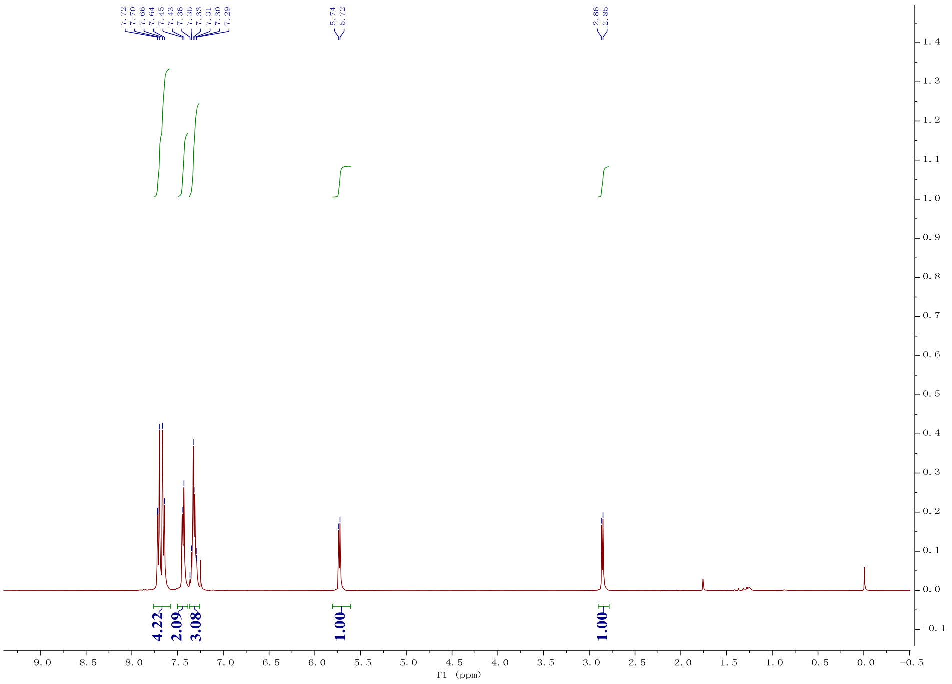

Figure S152. ^{1}H NMR spectrum of 25j, Related to Figure 7

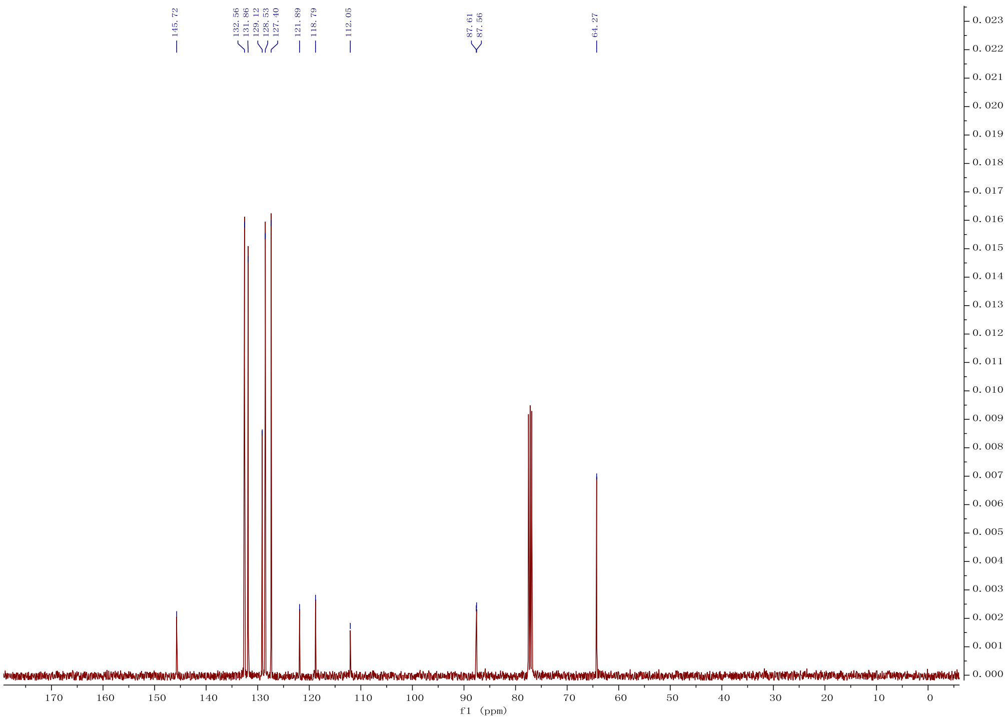

Figure S153. ^{13}**C NMR spectrum of 25j, Related to Figure 7**

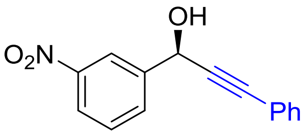

(R)-**25k**

HPLC: enantiomeric excess of **25k** (ee > 99%) was determined by high-performance liquid chromatography (HPLC) using a chiral stationary phase (OD-H column, flow rate = 1.0 mL/ min, eluent: hexane/ isopropanol = 80/20, 254 nm absorbance), retention times: major enantiomer (t_R = 8.18 min), minor enantiomer (t_R = 77.60 min).

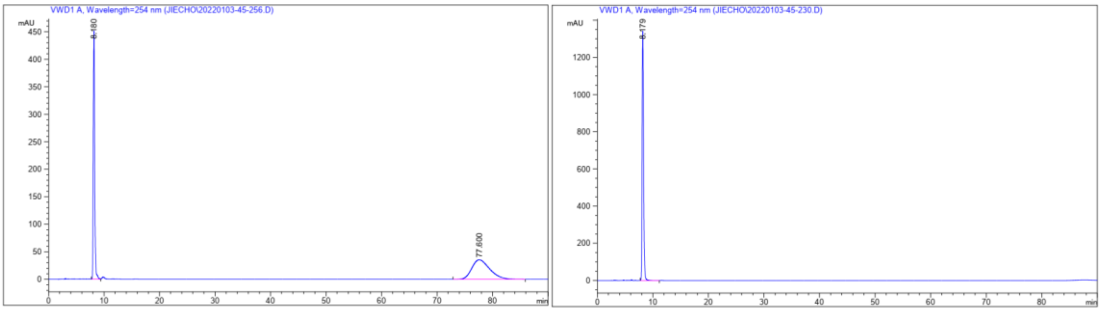

Figure S154. HPLC traces of 25k, Related to Figure 7

Signal 1: VWD1 A, Wavelength=254 nm

Peak #	RetTime [min]	Type	Width [min]	Area mAU *s	Height [mAU]	Area %
1	8.180	BV	0.2711	8007.78418	451.22012	50.2630
2	77.600	BB	3.1187	7923.98975	35.47136	49.7370

Totals : 1.59318e4 486.69149

Signal 1: VWD1 A, Wavelength=254 nm

Peak #	RetTime [min]	Type	Width [min]	Area mAU *s	Height [mAU]	Area %
1	8.179	VV	0.2793	2.43978e4	1340.50867	100.0000

Totals : 2.43978e4 1340.50867

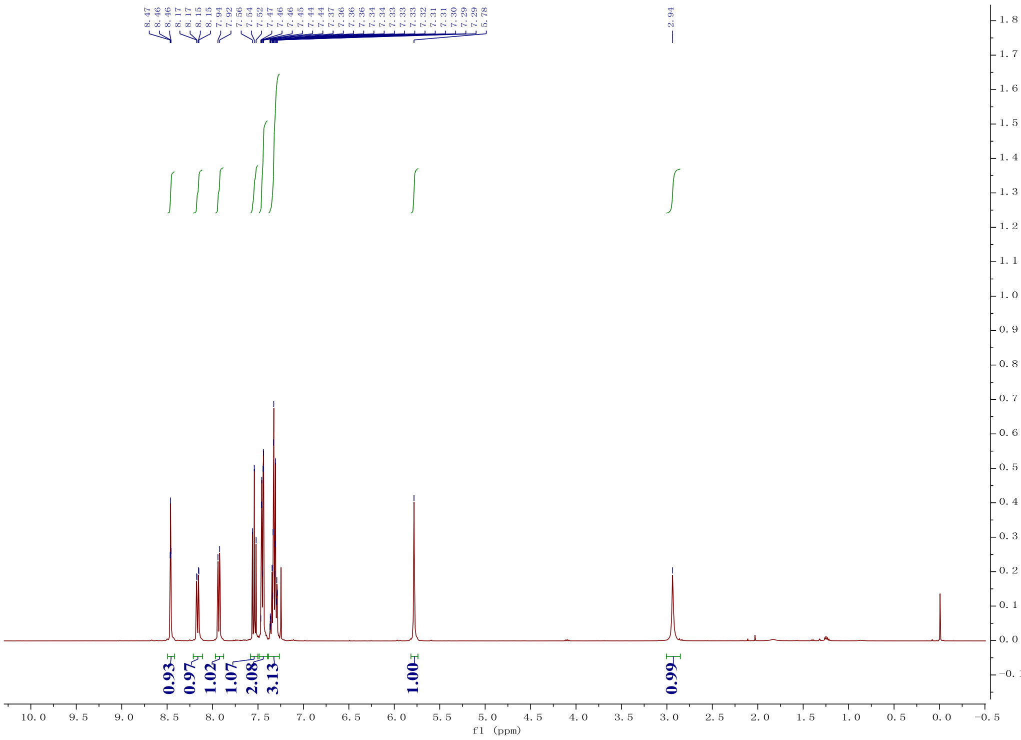

Figure S155. ^{1}H NMR spectrum of 25k, Related to Figure 7

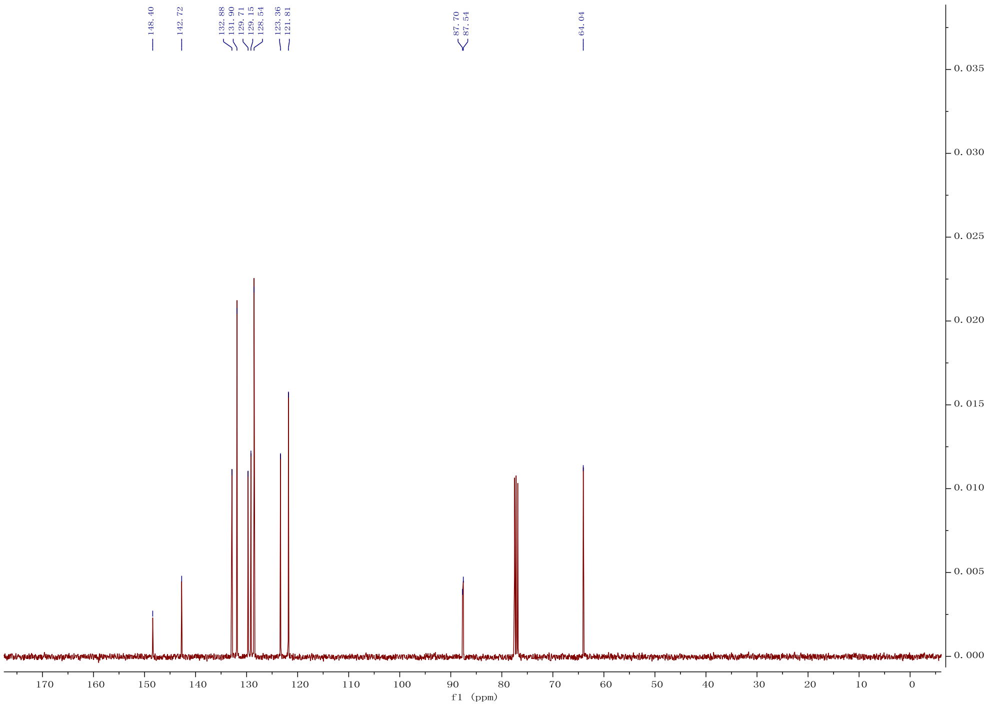

Figure S156. ^{13}C NMR spectrum of 25k, Related to Figure 7

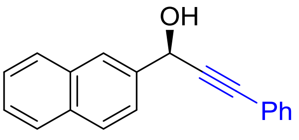

(R)-**25l**

HPLC: enantiomeric excess of **25l** (ee = 94%) was determined by high-performance liquid chromatography (HPLC) using a chiral stationary phase (OD-H column, flow rate = 1.0 mL/ min, eluent: hexane/ isopropanol = 80/20, 254 nm absorbance), retention times: major enantiomer (t_R = 9.24 min), minor enantiomer (t_R = 21.47 min).

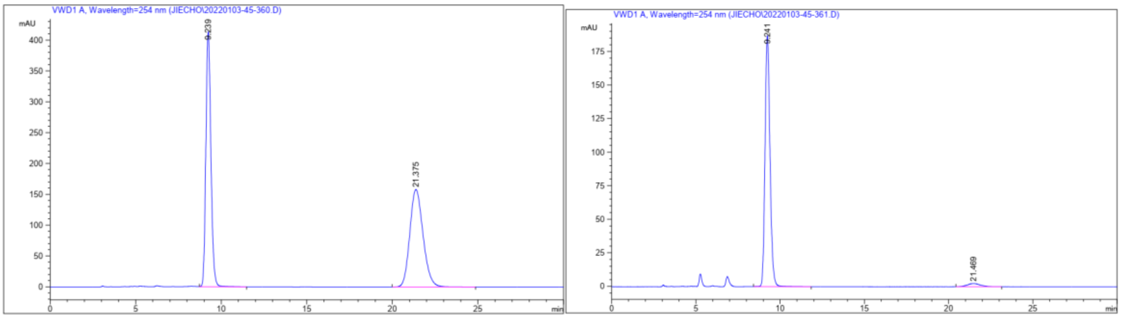

Figure S157. HPLC traces of 25l, Related to Figure 7

Signal 1: VWD1 A, Wavelength=254 nm

Peak #	RetTime [min]	Type	Width [min]	Area mAU *s	Height [mAU]	Area %
1	9.239	VB	0.3142	8449.82617	413.37766	49.9266
2	21.375	BB	0.8329	8474.68555	157.89297	50.0734
Totals :				1.69245e4	571.27063	

Signal 1: VWD1 A, Wavelength=254 nm

Peak #	RetTime [min]	Type	Width [min]	Area mAU *s	Height [mAU]	Area %
1	9.241	BB	0.3155	3838.93774	186.76086	96.8165
2	21.469	BB	0.7728	126.22954	2.33721	3.1835
Totals :				3965.16728	189.09807	

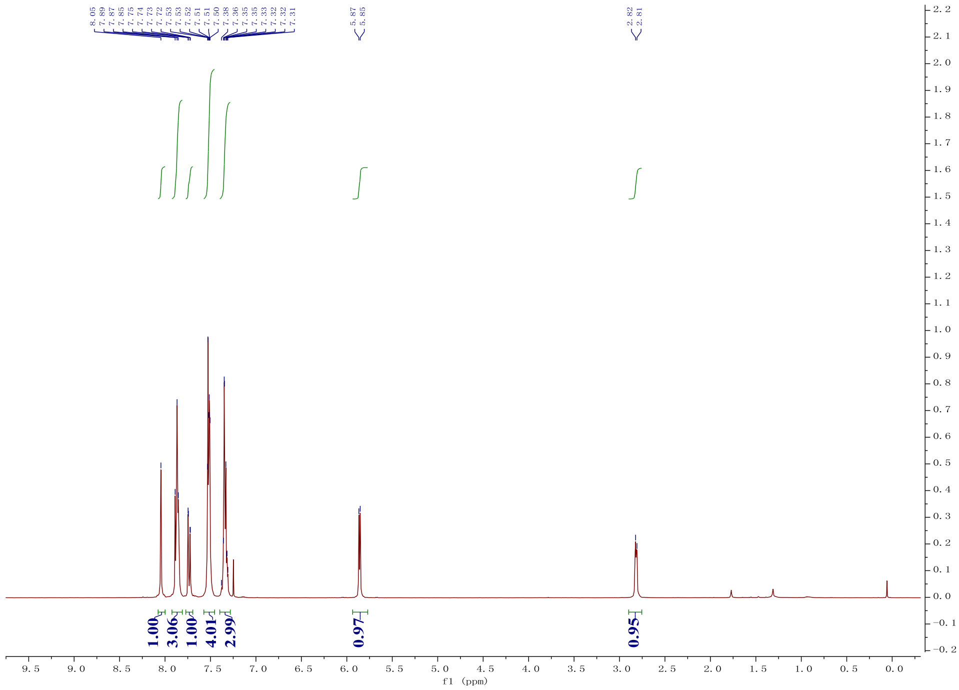

Figure S158. ^{1}H NMR spectrum of 25l, Related to Figure 7

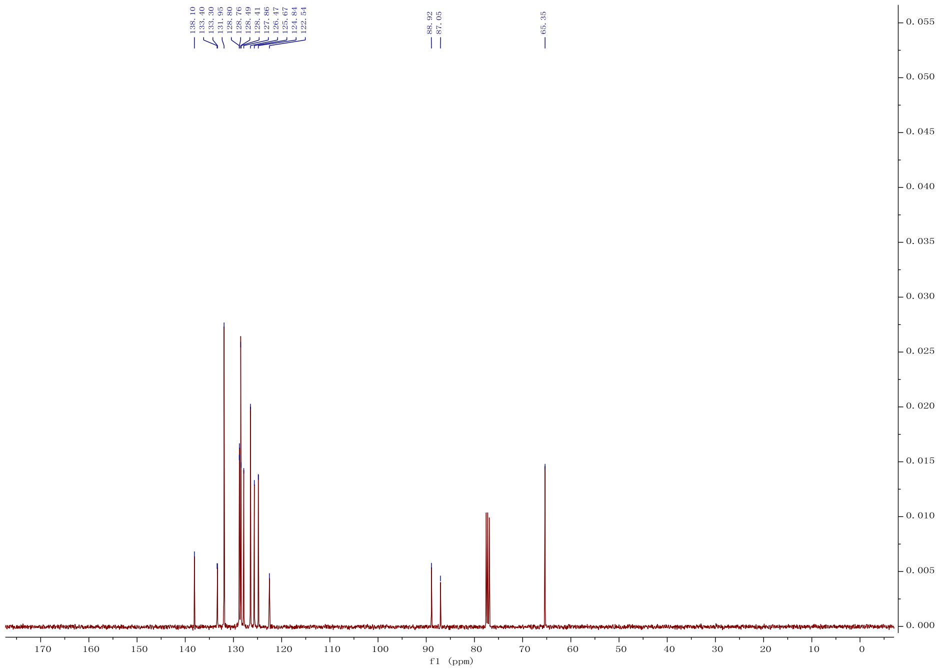

Figure S159. ^{13}C NMR spectrum of 25l, Related to Figure 7

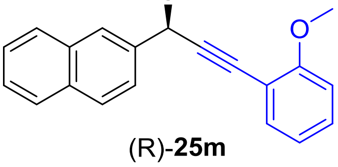

(R)-**25m**

HPLC: enantiomeric excess of **25m** (ee = 96%) was determined by high-performance liquid chromatography (HPLC) using a chiral stationary phase (OD-H column, flow rate = 1.0 mL/ min, eluent: hexane/ isopropanol = 80/20, 254 nm absorbance), retention times: major enantiomer (t_R = 14.75 min), minor enantiomer (t_R = 53.13 min).

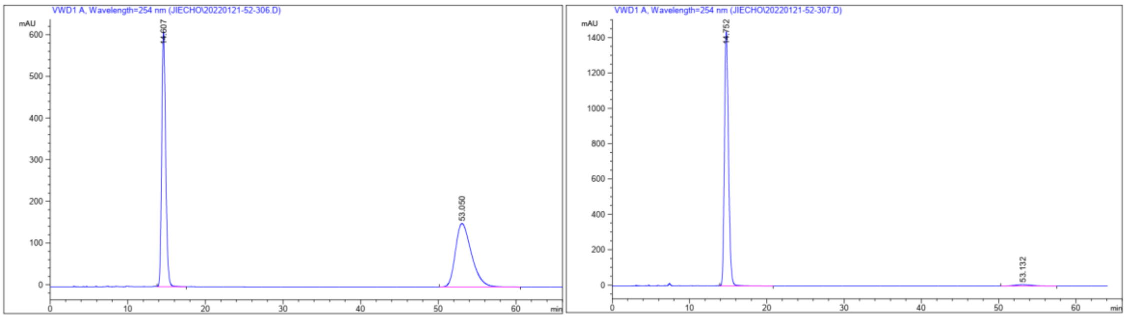

Figure S160. HPLC traces of 25m, Related to Figure 7

Signal 1: VWD1 A, Wavelength=254 nm

Peak #	RetTime [min]	Type	Width [min]	Area mAU *s	Height [mAU]	Area %
1	14.607	VB	0.5462	2.16083e4	611.47522	49.8286
2	53.050	BB	2.1608	2.17569e4	152.11385	50.1714
Totals :				4.33652e4	763.58907	

Signal 1: VWD1 A, Wavelength=254 nm

Peak #	RetTime [min]	Type	Width [min]	Area mAU *s	Height [mAU]	Area %
1	14.752	VB	0.5666	5.25805e4	1437.38306	97.7760
2	53.132	BB	1.6938	1195.98914	8.35280	2.2240
Totals :				5.37765e4	1445.73586	

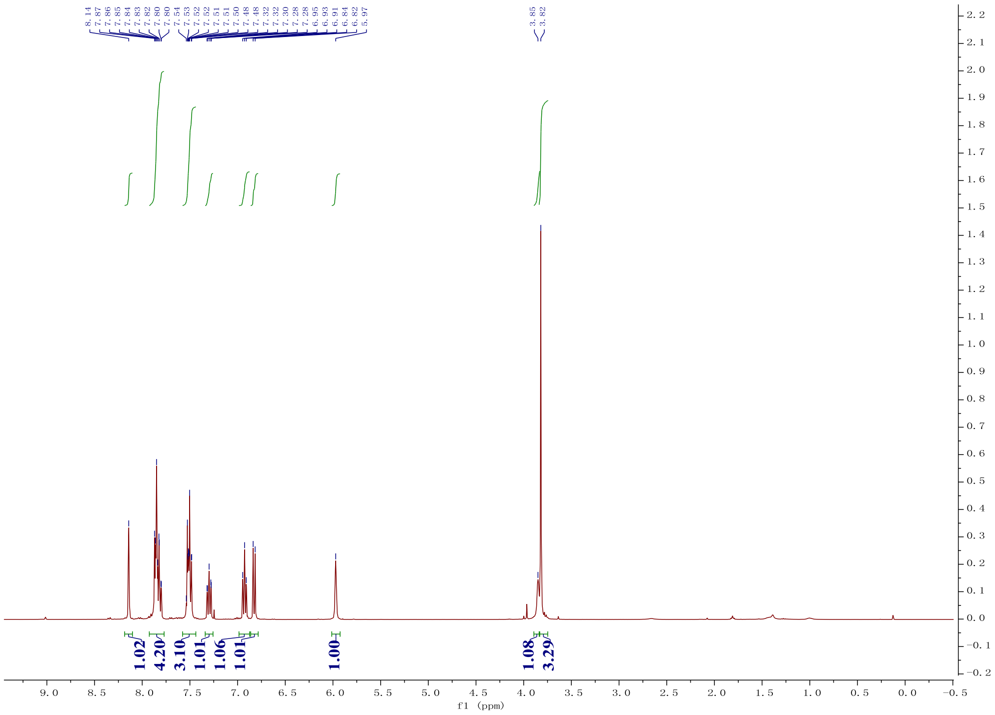

Figure S161. ¹H NMR spectrum of 25m, Related to Figure 7

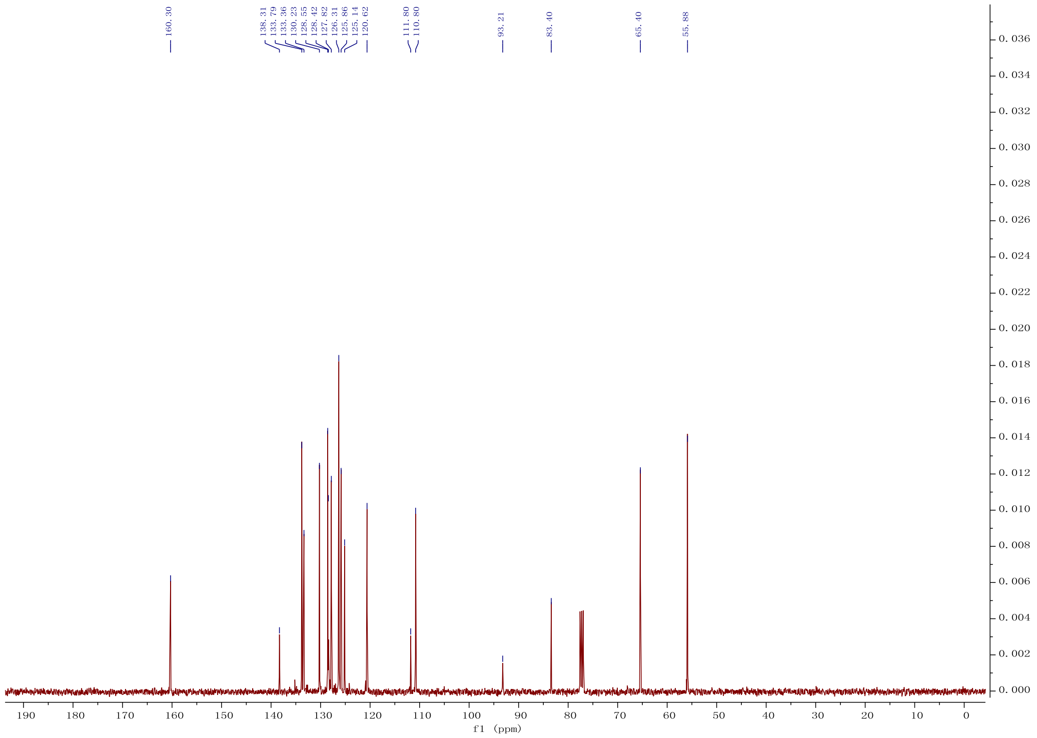

Figure S162. ¹³C NMR spectrum of 25m, Related to Figure 7

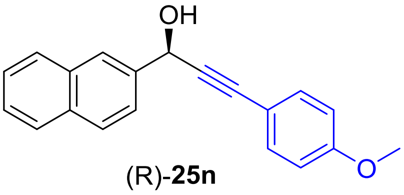

(R)-**25n**

HPLC: enantiomeric excess of **25n** (ee = 95%) was determined by high-performance liquid chromatography (HPLC) using a chiral stationary phase (OD-H column, flow rate = 1.0 mL/ min, eluent: hexane/ isopropanol = 80/20, 254 nm absorbance), retention times: major enantiomer (t_R = 12.38 min), minor enantiomer (t_R = 44.32 min).

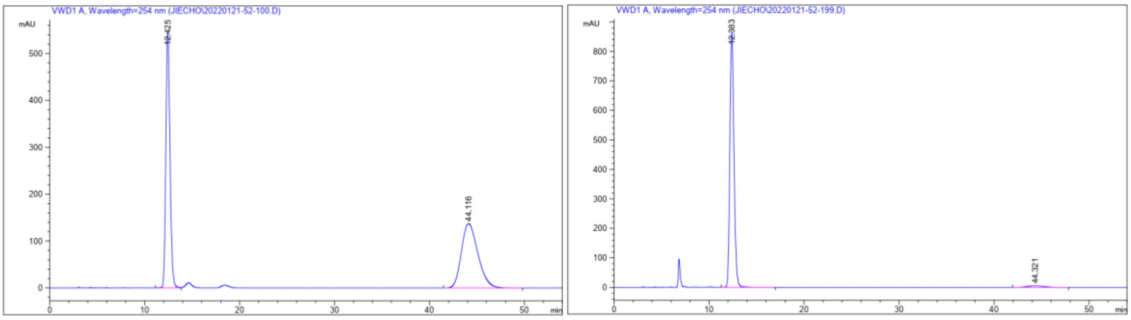

Figure S163. HPLC traces of 25n, Related to Figure 7

Signal 1: VWD1 A, Wavelength=254 nm

Peak #	RetTime [min]	Type	Width [min]	Area mAU *s	Height [mAU]	Area %
1	12.425	BV	0.4716	1.65797e4	544.69702	49.9773
2	44.116	BB	1.8565	1.65947e4	137.49406	50.0227
Totals :				3.31744e4	682.19109	

Signal 1: VWD1 A, Wavelength=254 nm

Peak #	RetTime [min]	Type	Width [min]	Area mAU *s	Height [mAU]	Area %
1	12.383	VB	0.4683	2.61103e4	865.95581	97.5221
2	44.321	BB	1.3989	663.41992	5.66243	2.4779
Totals :				2.67737e4	871.61824	

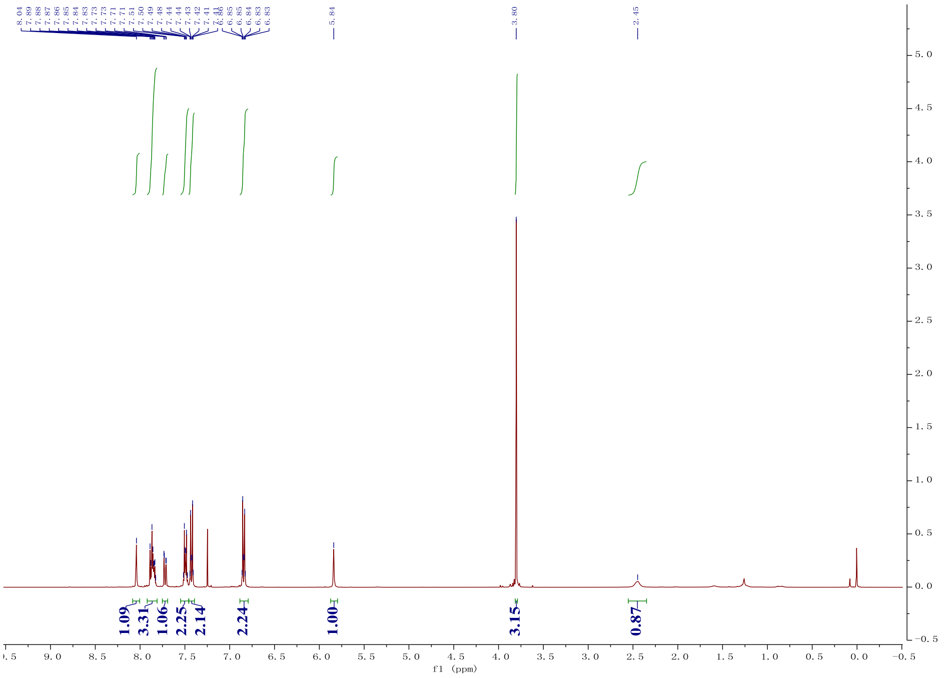

Figure S164. ^{1}H NMR spectrum of 25n, Related to Figure 7

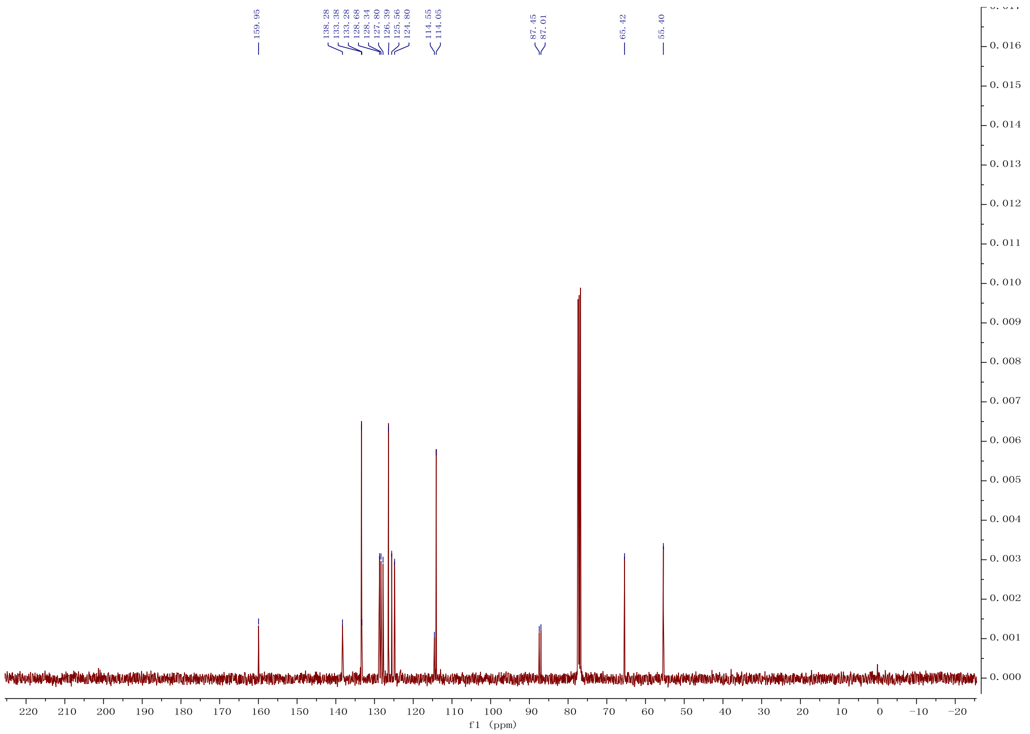

Figure S165. ^{13}**C NMR spectrum of 25n, Related to Figure 7**

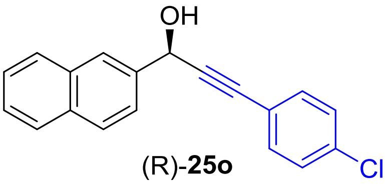

HPLC: enantiomeric excess of **25o** (ee = 95%) was determined by high-performance liquid chromatography (HPLC) using a chiral stationary phase (OD-H column, flow rate = 1.0 mL/ min, eluent: hexane/ isopropanol = 80/20, 254 nm absorbance), retention times: major enantiomer (t_R = 8.96 min), minor enantiomer (t_R = 10.90 min).

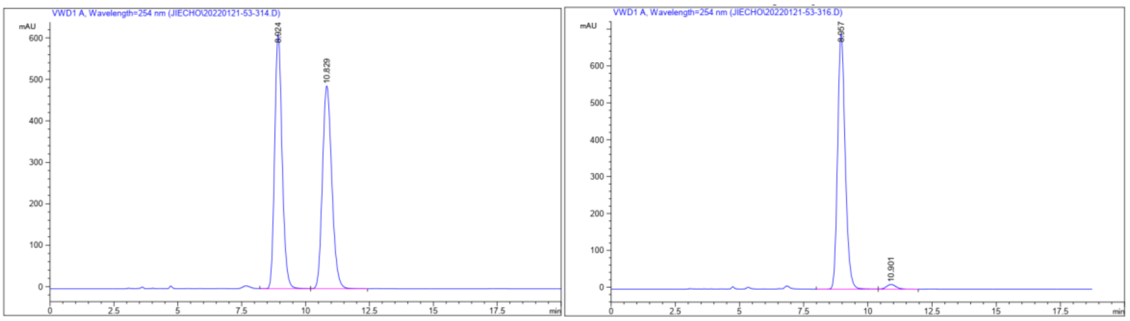

Figure S166. HPLC traces of 25o, Related to Figure 7

Signal 1: VWD1 A, Wavelength=254 nm

Peak #	RetTime [min]	Type	Width [min]	Area mAU *s	Height [mAU]	Area %
1	8.924	VB	0.3123	1.24218e4	612.58630	50.0228
2	10.829	BB	0.3922	1.24105e4	488.92938	49.9772
Totals :				2.48324e4	1101.51569	

Signal 1: VWD1 A, Wavelength=254 nm

Peak #	RetTime [min]	Type	Width [min]	Area mAU *s	Height [mAU]	Area %
1	8.957	BV	0.3195	1.42897e4	692.14722	97.6505
2	10.901	VB	0.4076	343.81674	12.99990	2.3495
Totals :				1.46335e4	705.14712	

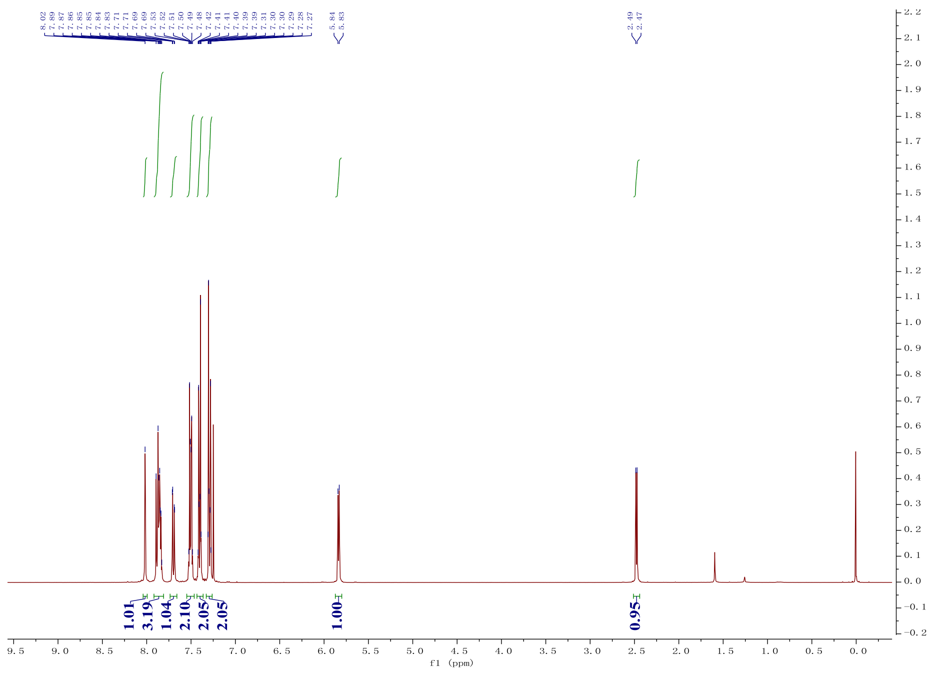

Figure S167. ^{1}H NMR spectrum of 25o, Related to Figure 7

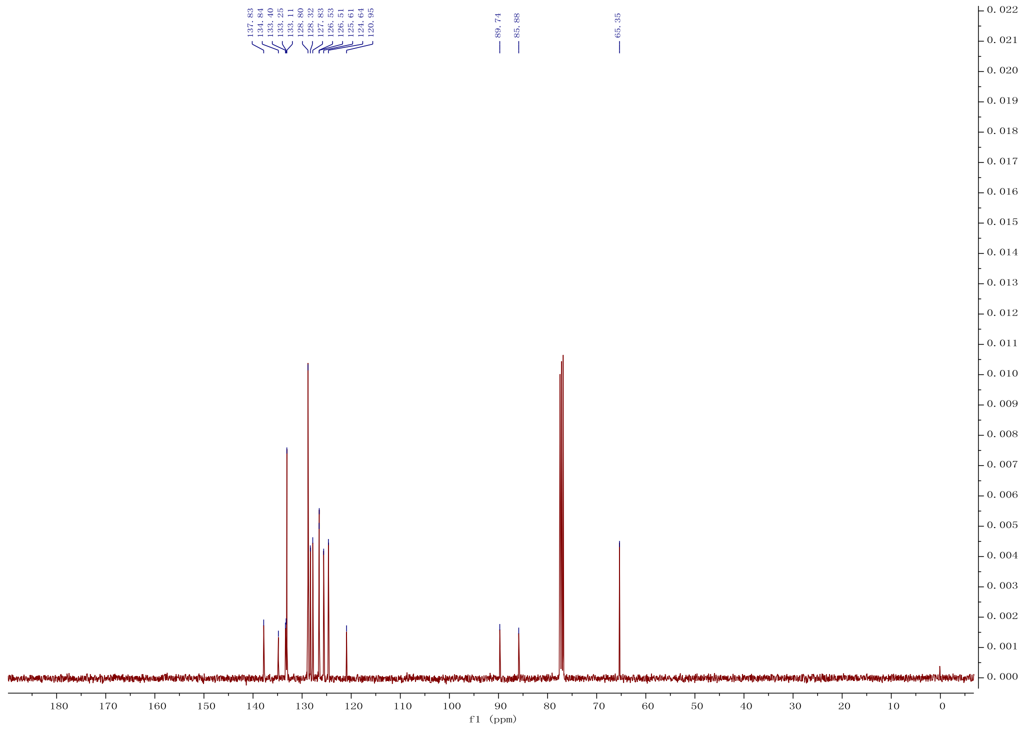

Figure S168. ^{13}C NMR spectrum of 25o, Related to Figure 7

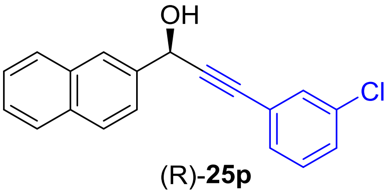

(R)-**25p**

HPLC: enantiomeric excess of **25p** (ee = 94%) was determined by high-performance liquid chromatography (HPLC) using a chiral stationary phase (OD-H column, flow rate = 1.0 mL/ min, eluent: hexane/ isopropanol = 80/20, 254 nm absorbance), retention times: major enantiomer (t_R = 10.82 min), minor enantiomer (t_R = 16.58 min).

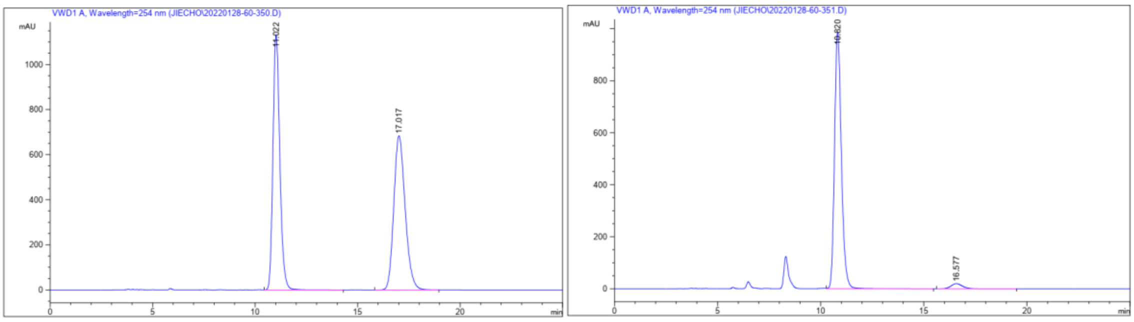

Figure S169. HPLC traces of 25p, Related to Figure 7

Signal 1: VWD1 A, Wavelength=254 nm

Peak #	RetTime [min]	Type	Width [min]	Area mAU *s	Height [mAU]	Area %
1	11.022	VB	0.3579	2.62452e4	1131.84509	49.9879
2	17.017	BB	0.5938	2.62579e4	683.50439	50.0121
Totals :				5.25032e4	1815.34949	

Signal 1: VWD1 A, Wavelength=254 nm

Peak #	RetTime [min]	Type	Width [min]	Area mAU *s	Height [mAU]	Area %
1	10.820	VB	0.3557	2.26982e4	986.98358	96.8286
2	16.577	BB	0.5911	743.43365	19.34341	3.1714
Totals :				2.34416e4	1006.32699	

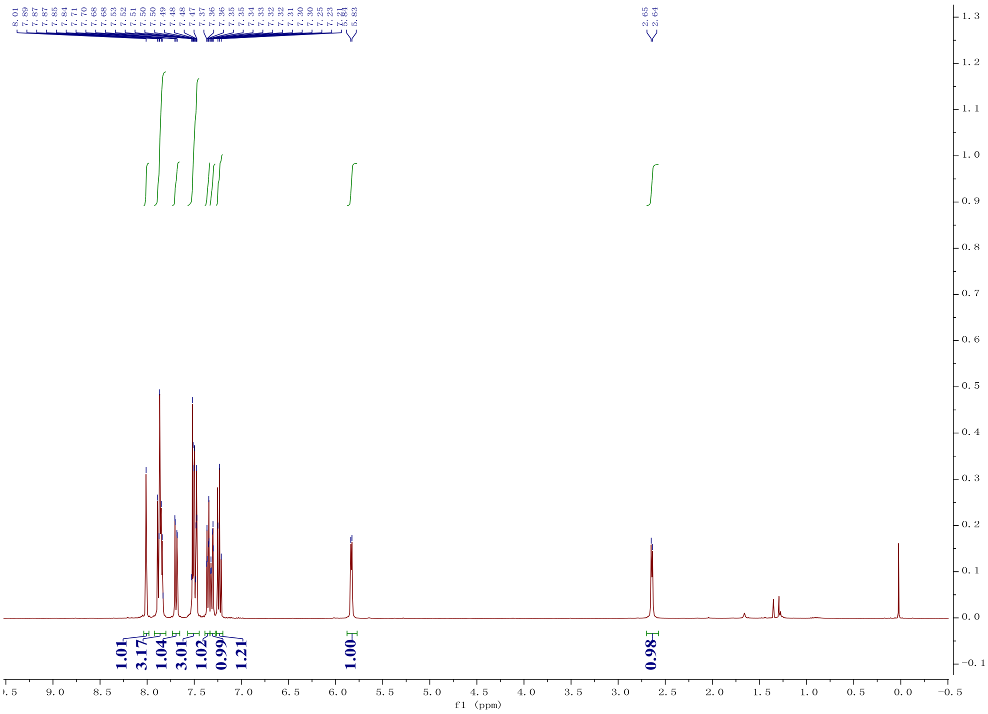

Figure S170. ^{1}H NMR spectrum of 25p, Related to Figure 7

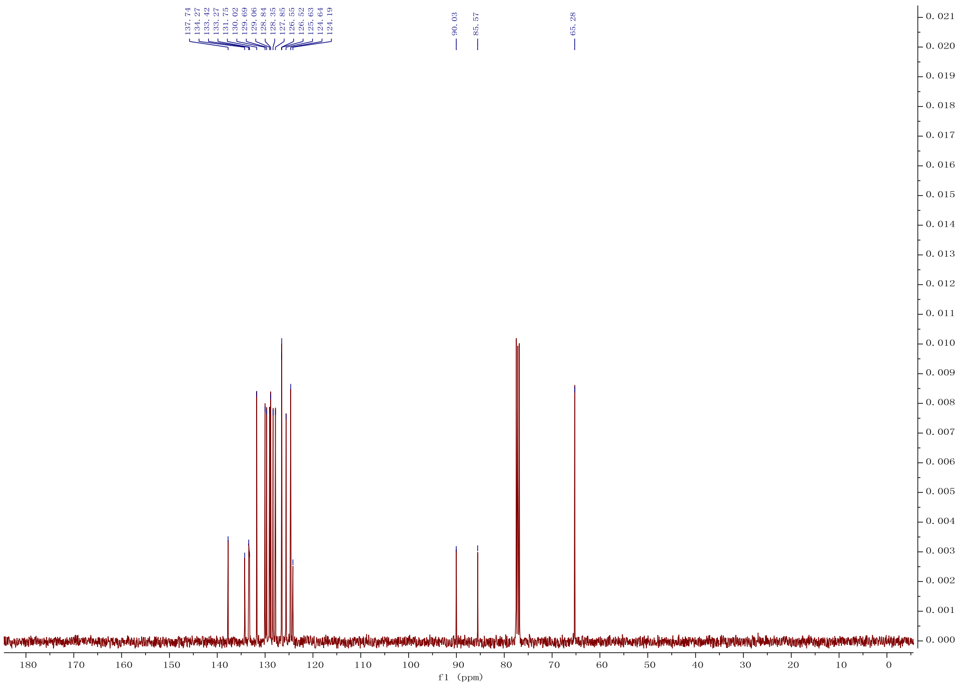

Figure S171. ^{13}**C NMR spectrum of 25p, Related to Figure 7**

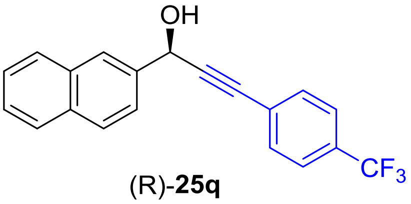

(R)-**25q**

HPLC: enantiomeric excess of **25q** (ee = 96%) was determined by high-performance liquid chromatography (HPLC) using a chiral stationary phase (OD-H column, flow rate = 1.0 mL/ min, eluent: hexane/ isopropanol = 80/20, 254 nm absorbance), retention times: major enantiomer (t_R = 27.62 min), minor enantiomer (t_R = 30.75 min).

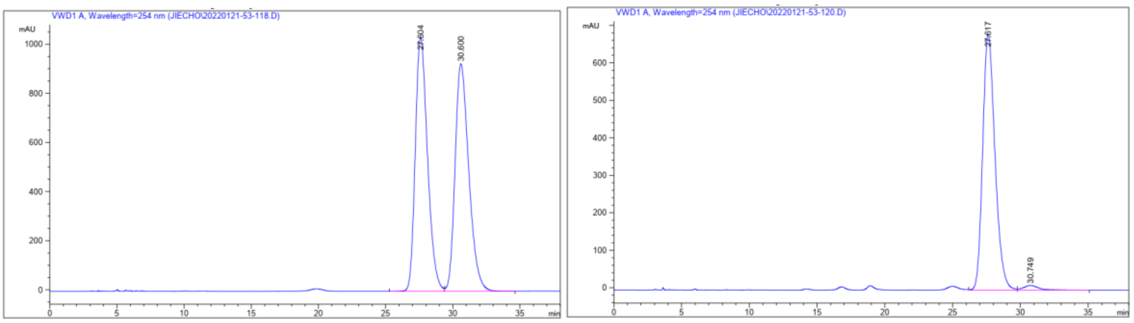

Figure S172. HPLC traces of 25q, Related to Figure 7

Signal 1: VWD1 A, Wavelength=254 nm

Peak #	RetTime [min]	Type	Width [min]	Area mAU *s	Height [mAU]	Area %
1	27.604	BV	0.9776	6.54600e4	1033.74756	49.8623
2	30.600	VB	1.0877	6.58216e4	926.86078	50.1377
Totals :				1.31282e5	1960.60834	

Signal 1: VWD1 A, Wavelength=254 nm

Peak #	RetTime [min]	Type	Width [min]	Area mAU *s	Height [mAU]	Area %
1	27.617	VV	0.9769	4.32115e4	682.96478	97.7025
2	30.749	VB	1.1873	1016.13416	12.30901	2.2975
Totals :				4.42277e4	695.27379	

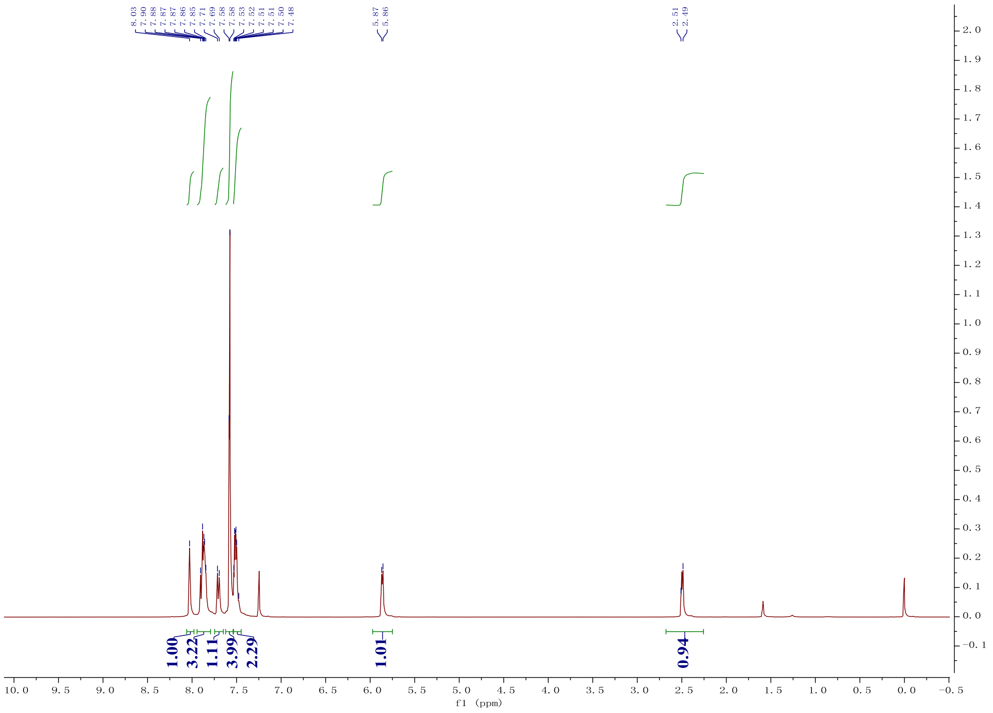

Figure S173. ^{1}H NMR spectrum of 25q, Related to Figure 7

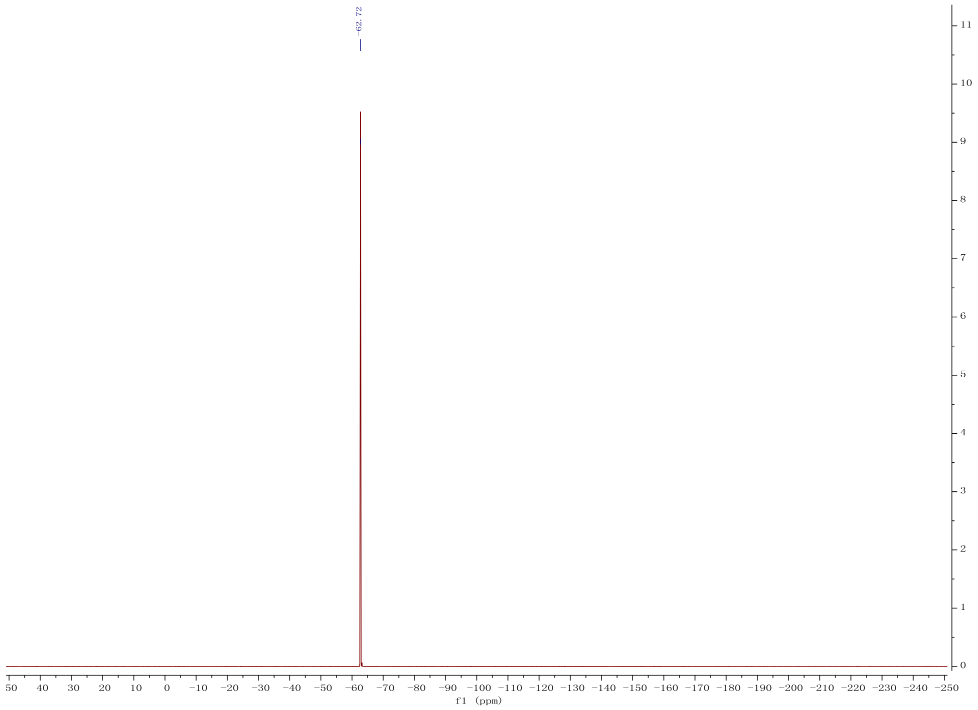

Figure S174. ^{13}C NMR spectrum of 25q, Related to Figure 7

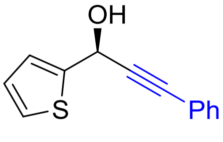

(S)-**25r**

HPLC: enantiomeric excess of **25r** (ee = 92%) was determined by high-performance liquid chromatography (HPLC) using a chiral stationary phase (OD-H column, flow rate = 1.0 mL/ min, eluent: hexane/ isopropanol = 80/20, 254 nm absorbance), retention times: major enantiomer (t_R = 6.80 min), minor enantiomer (t_R = 10.49 min).

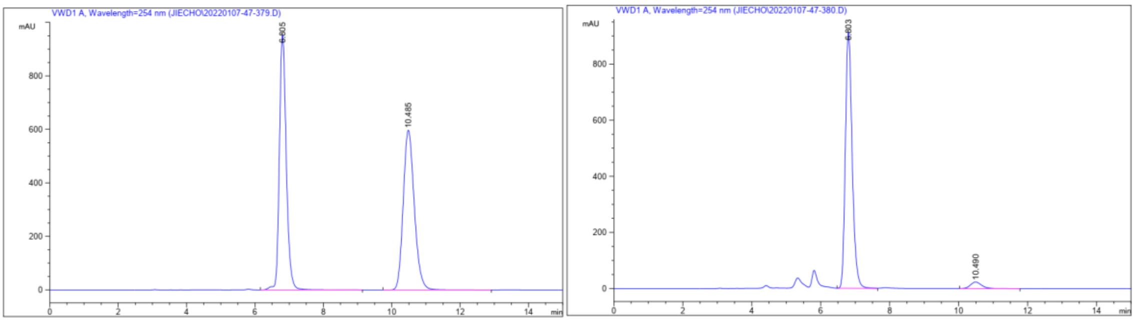

Figure S175. HPLC traces of 25r, Related to Figure 7

Signal 1: VWD1 A, Wavelength=254 nm

Peak #	RetTime [min]	Type	Width [min]	Area mAU *s	Height [mAU]	Area %
1	6.805	BB	0.2140	1.33301e4	953.83557	49.8414
2	10.485	BB	0.3480	1.34149e4	597.02478	50.1586
Totals :				2.67450e4	1550.86035	

Signal 1: VWD1 A, Wavelength=254 nm

Peak #	RetTime [min]	Type	Width [min]	Area mAU *s	Height [mAU]	Area %
1	6.803	BV	0.2126	1.25409e4	913.13562	96.0378
2	10.490	VB	0.3419	517.39539	23.18516	3.9622
Totals :				1.30583e4	936.32078	

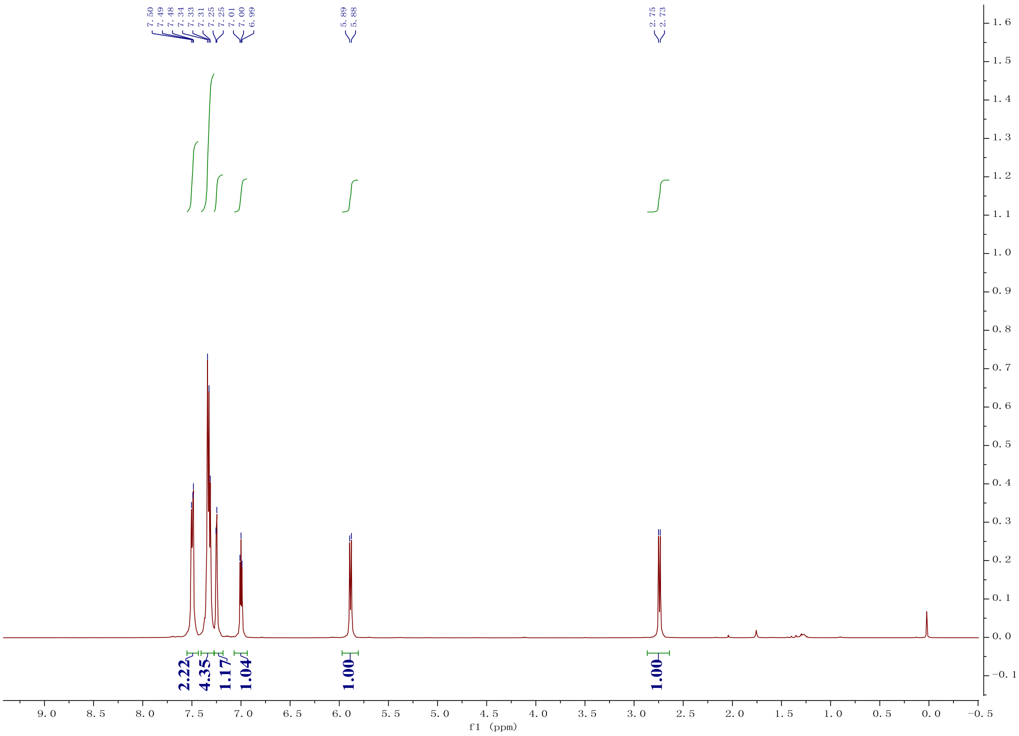

Figure S176. ^{1}H NMR spectrum of 25r, Related to Figure 7

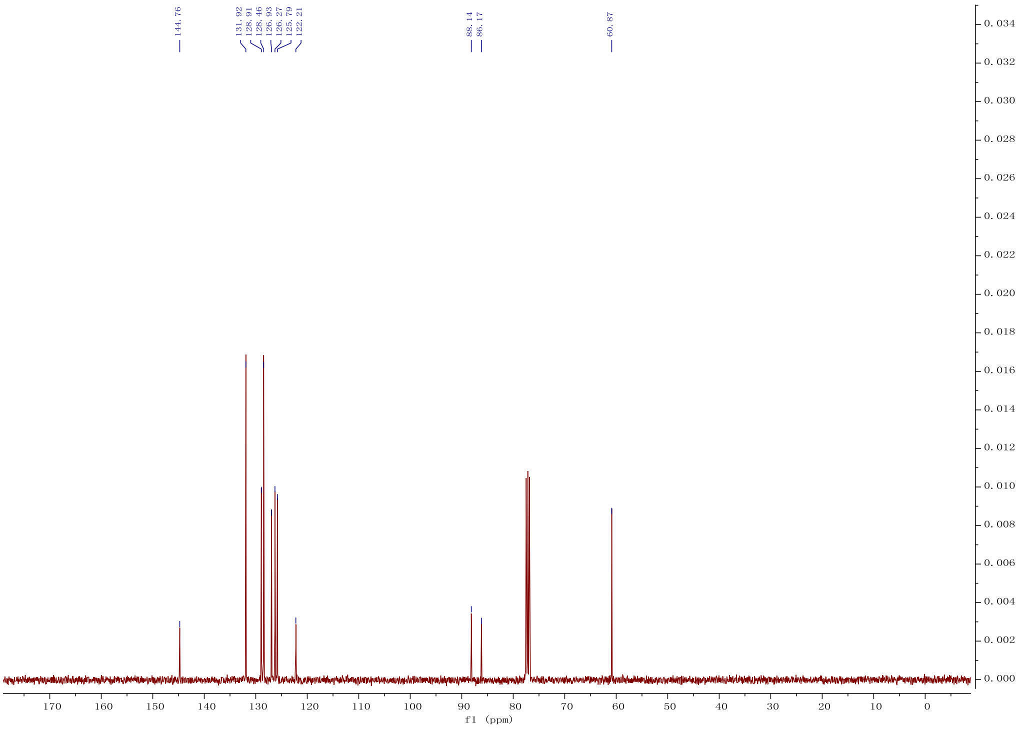

Figure S177. **^{13}C NMR spectrum of 25r, Related to Figure 7**

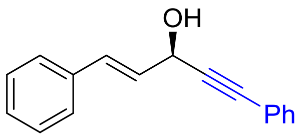

(R)-**25s**

HPLC: enantiomeric excess of **25s** (ee = 87%) was determined by high-performance liquid chromatography (HPLC) using a chiral stationary phase (OD-H column, flow rate = 1.0 mL/ min, eluent: hexane/ isopropanol = 80/20, 254 nm absorbance), retention times: major enantiomer (t_R = 11.22 min), minor enantiomer (t_R = 25.40 min).

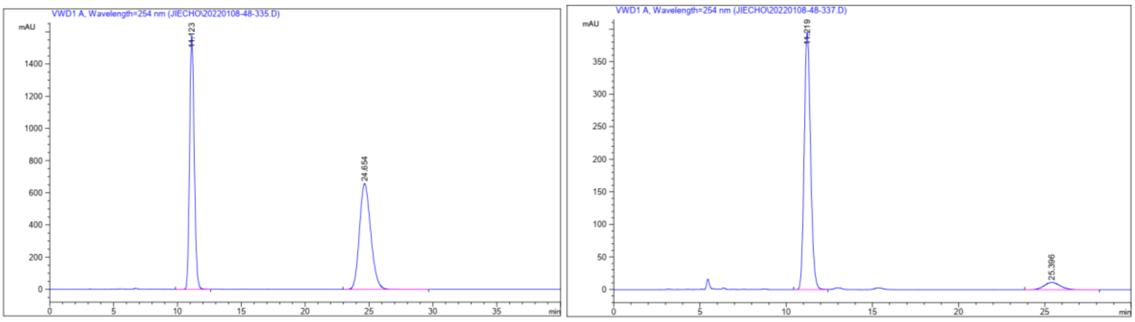

Figure S178. HPLC traces of 25s, Related to Figure 7

Signal 1: VWD1 A, Wavelength=254 nm

Peak #	RetTime [min]	Type	Width [min]	Area mAU *s	Height [mAU]	Area %
1	11.123	BV	0.3914	3.99480e4	1578.25146	49.7510
2	24.654	BB	0.9546	4.03480e4	657.57599	50.2490
Totals :				8.02960e4	2235.82745	

Signal 1: VWD1 A, Wavelength=254 nm

Peak #	RetTime [min]	Type	Width [min]	Area mAU *s	Height [mAU]	Area %
1	11.219	BV	0.3990	1.01706e4	395.47998	93.4696
2	25.396	BB	1.0058	710.58063	11.01670	6.5304
Totals :				1.08811e4	406.49668	

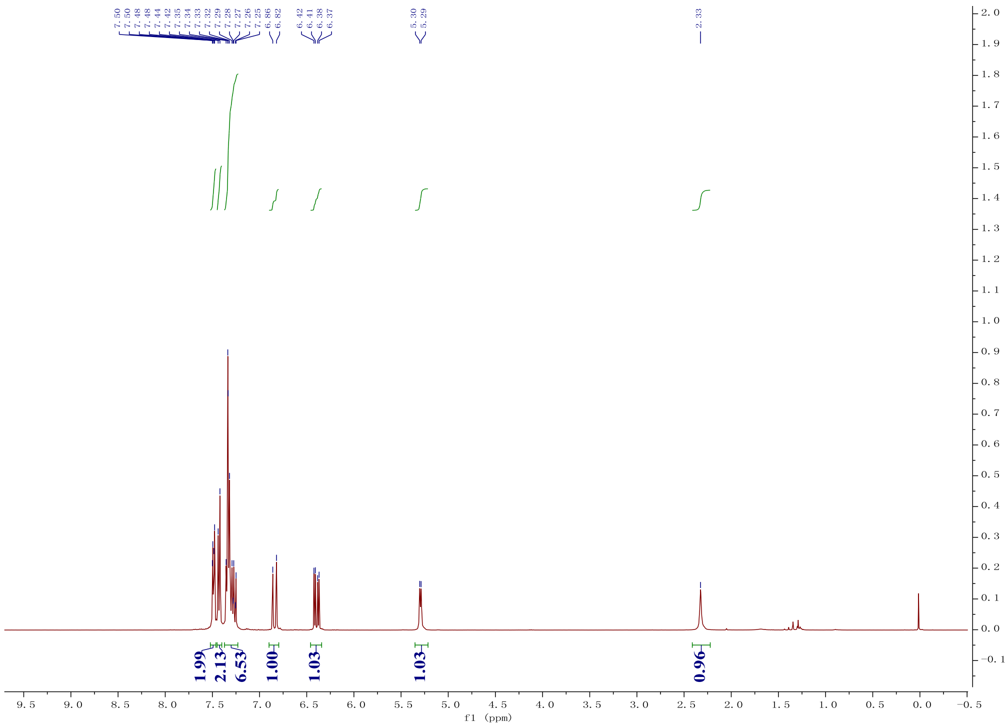

Figure S179. ^{1}H NMR spectrum of 25s, Related to Figure 7

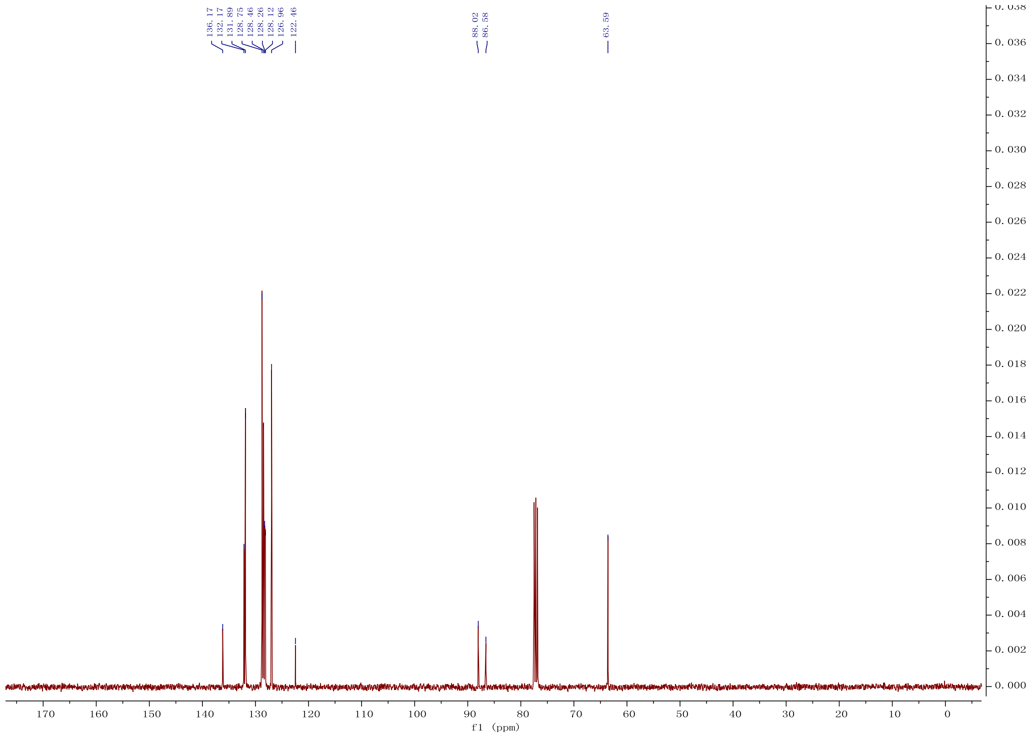

Figure S180. ^{13}C NMR spectrum of 25s, Related to Figure 7

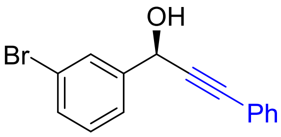

(R)-25t

HPLC: enantiomeric excess of **27b** (ee = 90%) was determined by high-performance liquid chromatography (HPLC) using a chiral stationary phase (OD-H column, flow rate = 1.0 mL/ min, eluent: hexane/ isopropanol = 80/20, 254 nm absorbance), retention times: minor enantiomer (t_R = 14.83 min), major enantiomer (t_R = 6.41 min).

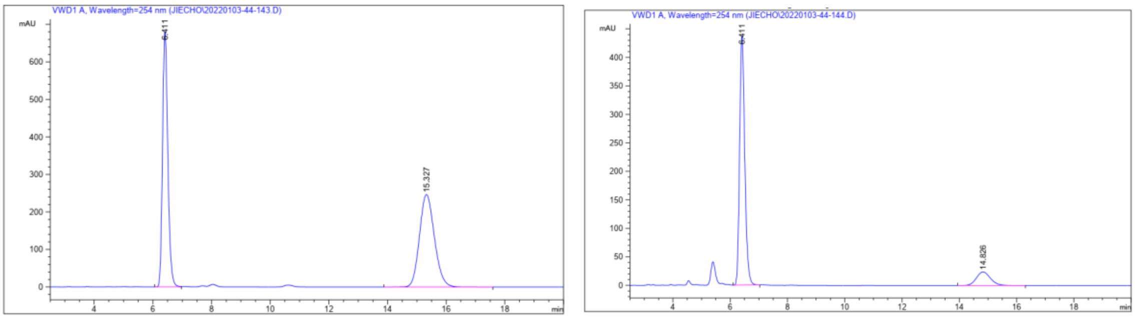

Figure S181. HPLC traces of 25t, Related to Figure 7

Signal 1: VWD1 A, Wavelength=254 nm

Peak #	RetTime [min]	Type	Width [min]	Area mAU *s	Height [mAU]	Area %
1	6.411	BV	0.1930	8532.71191	679.88855	49.4259
2	15.327	BB	0.5498	8730.94629	246.66498	50.5741
Totals :				1.72637e4	926.55353	

Signal 1: VWD1 A, Wavelength=254 nm

Peak #	RetTime [min]	Type	Width [min]	Area mAU *s	Height [mAU]	Area %
1	6.411	BB	0.1924	5454.53662	436.43701	87.0850
2	14.826	BB	0.5266	808.92413	23.85645	12.9150
Totals :				6263.46075	460.29346	

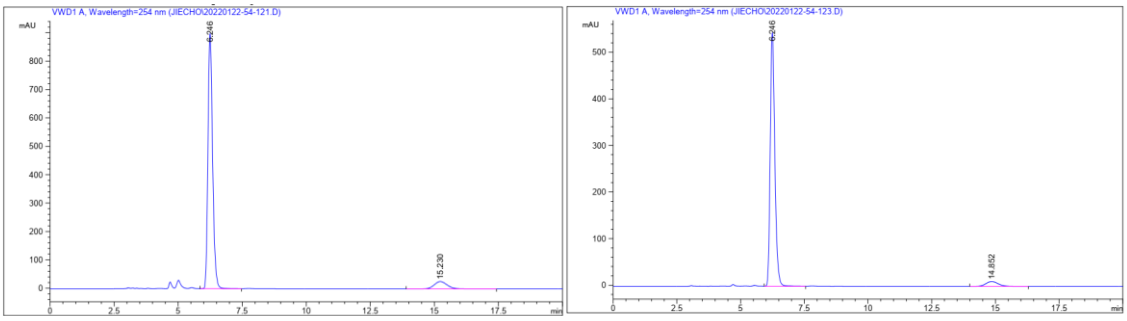

Figure S182. HPLC traces of 25t, Related to Figure 7

Signal 1: VWD1 A, Wavelength=254 nm

Peak #	RetTime [min]	Type	Width [min]	Area mAU *s	Height [mAU]	Area %
1	6.246	BB	0.1956	1.12420e4	897.99084	92.5124
2	15.230	BB	0.5423	909.89117	25.99649	7.4876
Totals :				1.21519e4	923.98733	

Signal 1: VWD1 A, Wavelength=254 nm

Peak #	RetTime [min]	Type	Width [min]	Area mAU *s	Height [mAU]	Area %
1	6.246	VV	0.1921	6779.37891	543.51331	95.0759
2	14.852	BB	0.5299	351.10962	10.34656	4.9241
Totals :				7130.48853	553.85987	

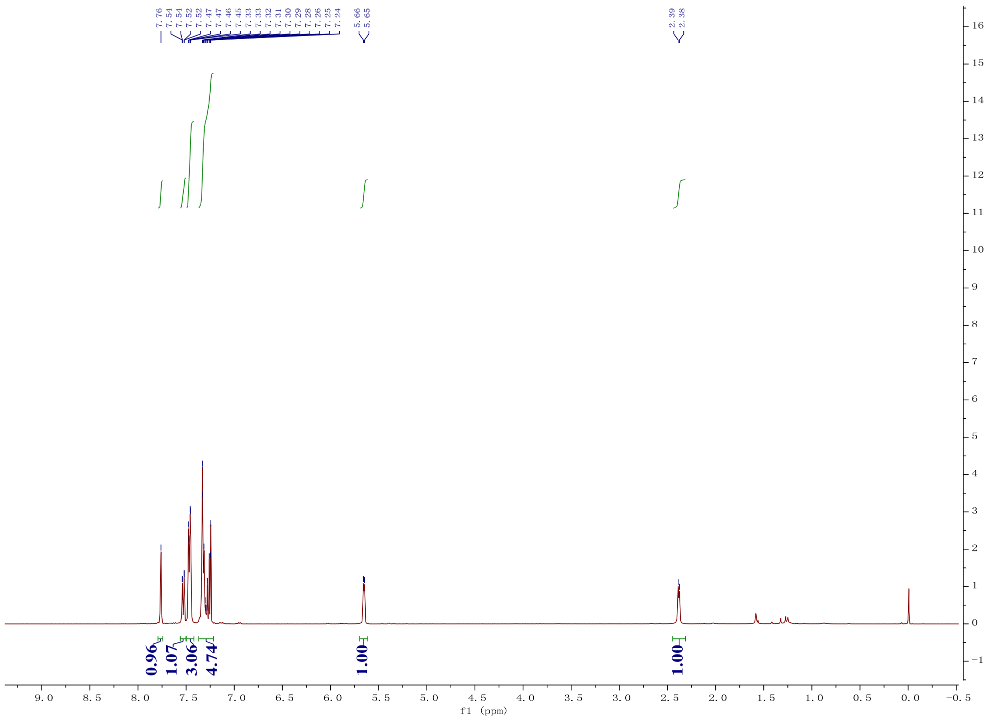

Figure S183. ^{1}H NMR spectrum of 25t, Related to Figure 7

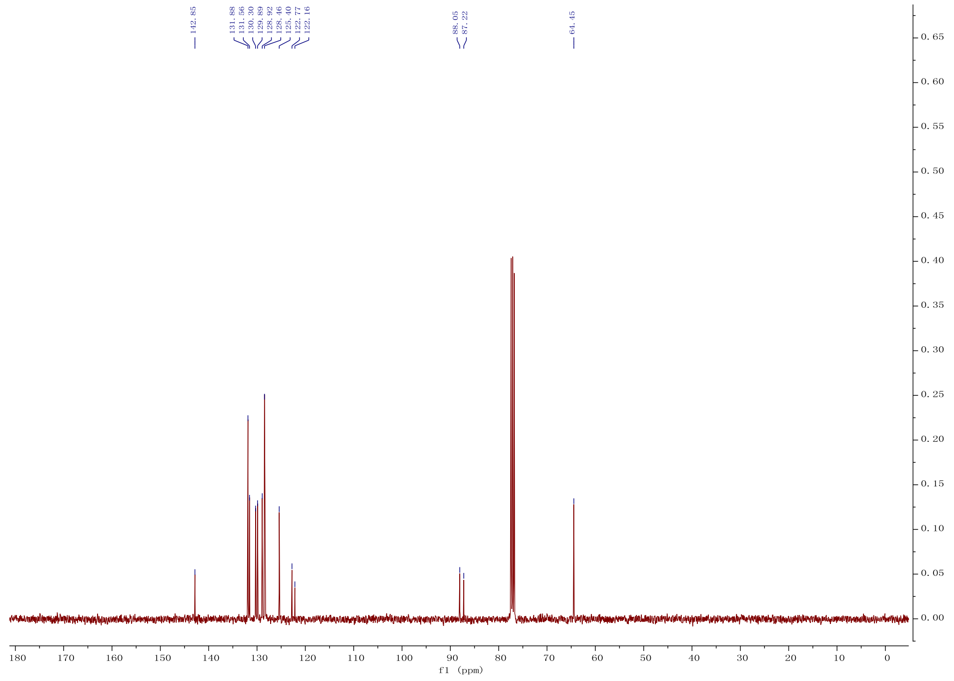

Figure S184. ^{13}C NMR spectrum of 25t, Related to Figure 7

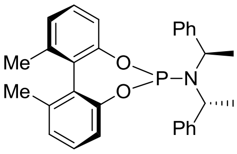

(*S*)-**L14**

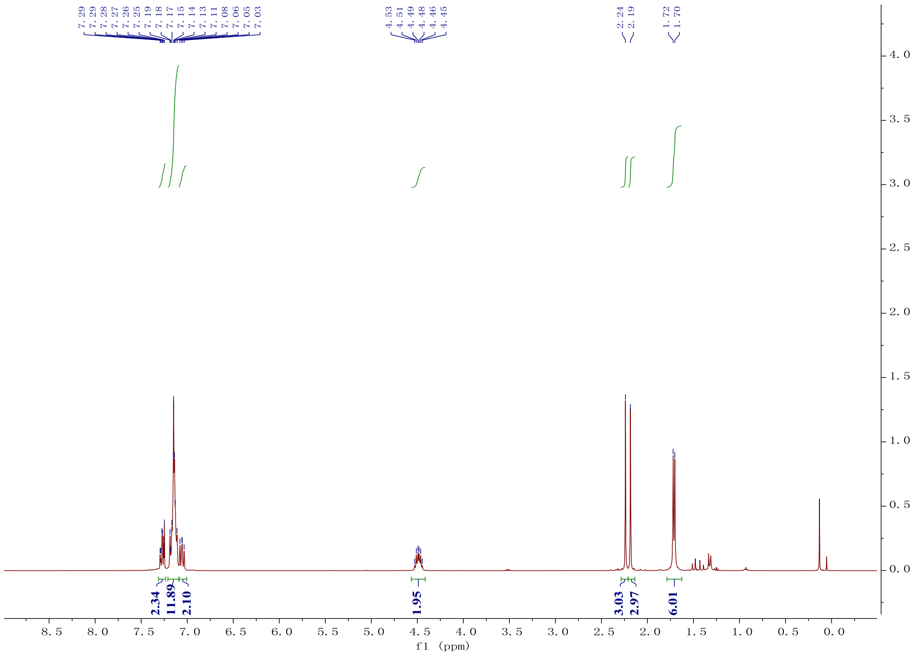

Figure S185. ^{1}H NMR spectrum of (*S*)-L14, Related to Figure 8a

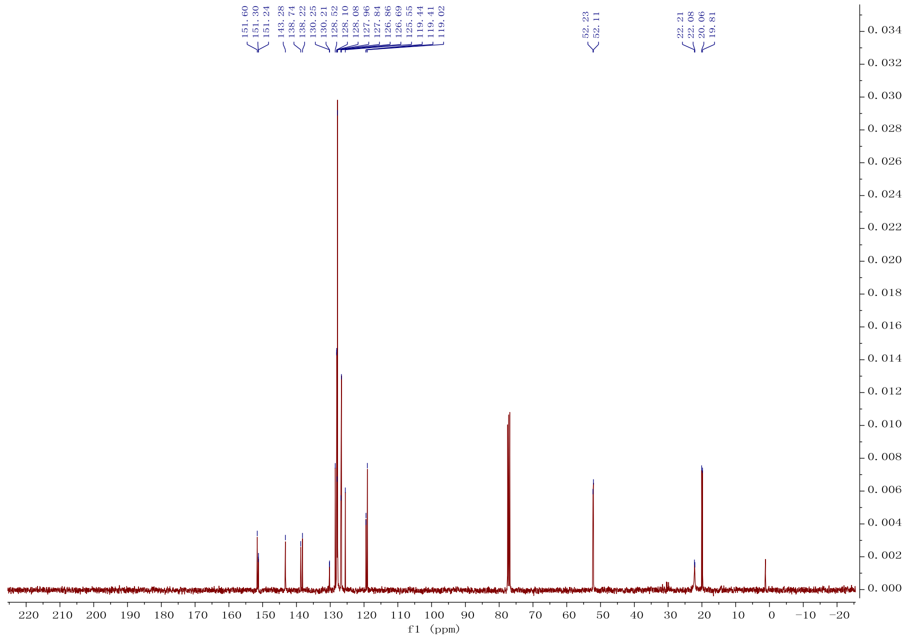

Figure S186. ^{13}C NMR spectrum of (*S*)-L14, Related to Figure 8a

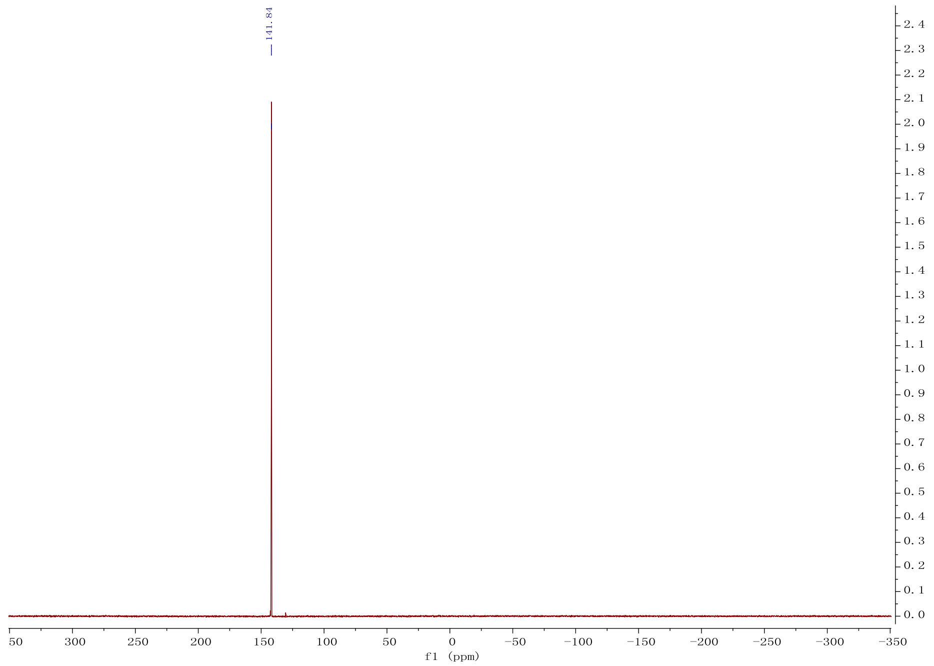

Figure S187. **^{19}F NMR spectrum of (*S*)-L14, Related to Figure 8a**

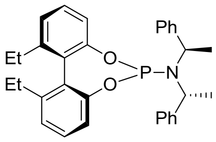

(*S*)-**L15**

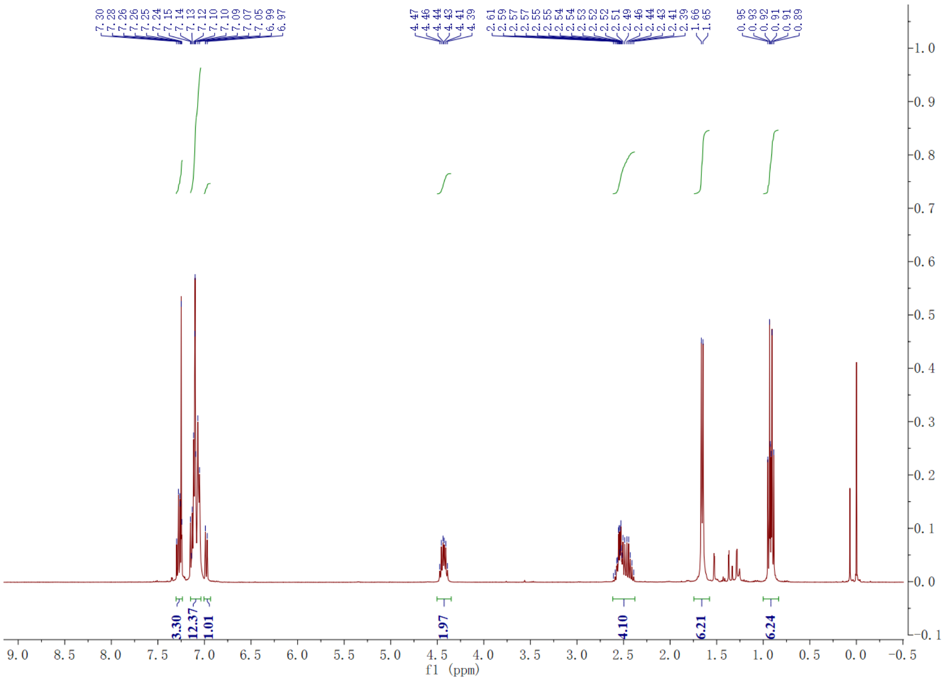

Figure S188. **^{1}H NMR spectrum of (*S*)-L15, Related to Figure 8a**

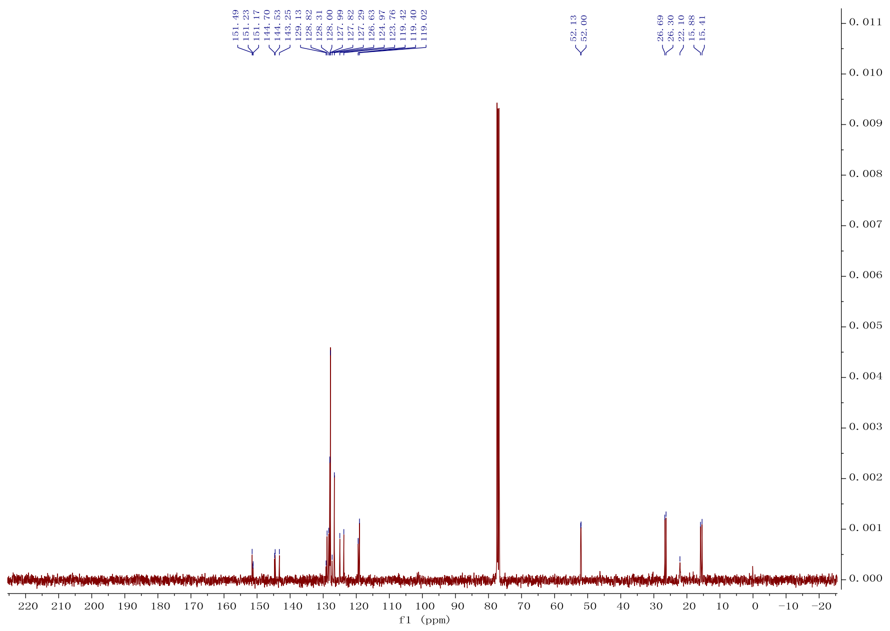

Figure S189. ^{13}C NMR spectrum of (*S*)-L15, Related to Figure 8a

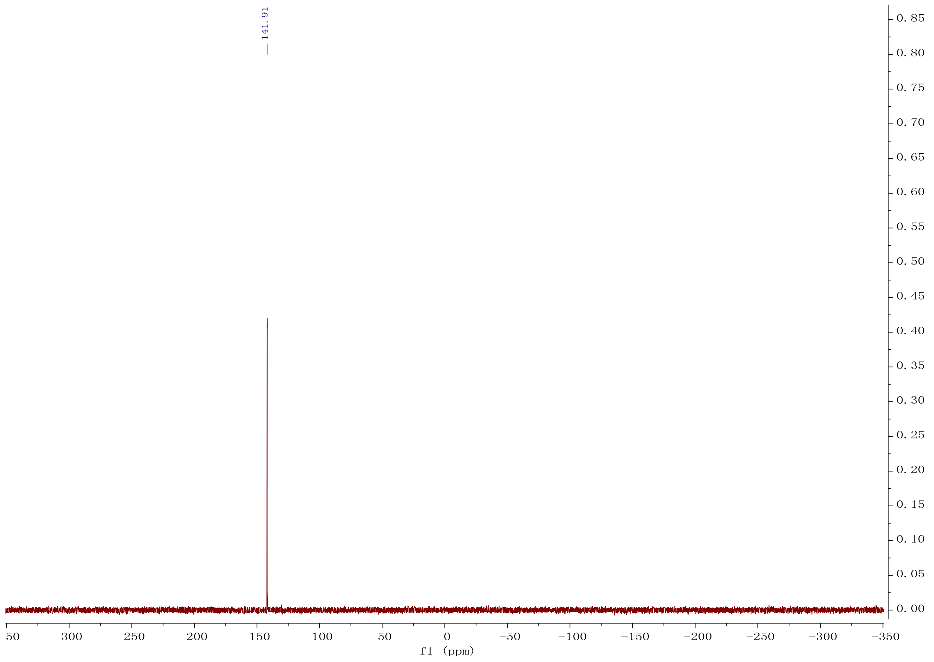

Figure S190. ^{19}F NMR spectrum of (*S*)-L15, Related to Figure 8a

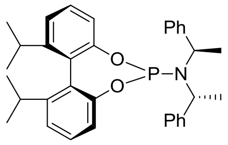

(*S*)-**L16**

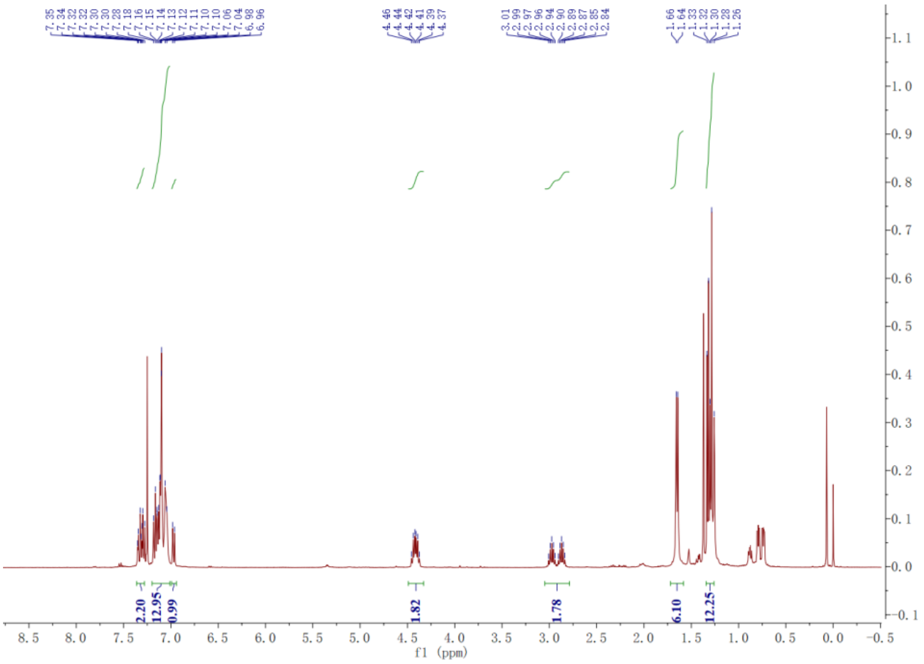

Figure S191. ^{1}H NMR spectrum of (*S*)-L16, Related to Figure 8a

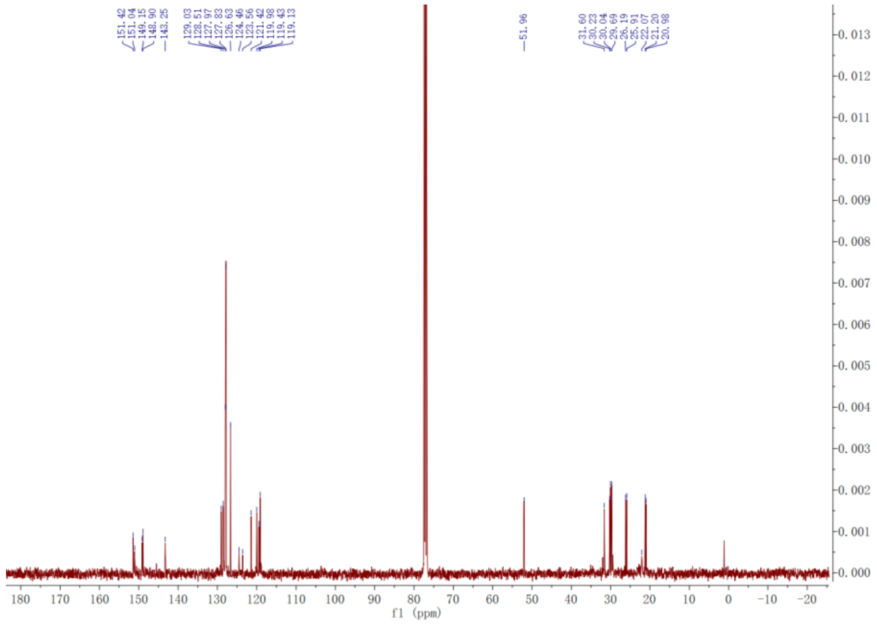

Figure S192. ^{13}C NMR spectrum of (*S*)-L16, Related to Figure 8a

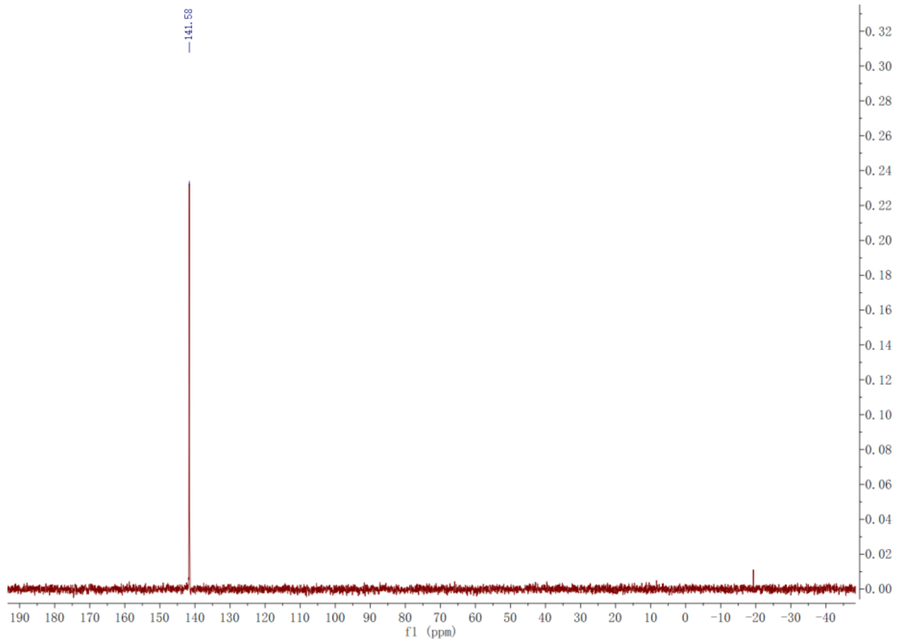

Figure S193. ^{19}F NMR spectrum of (*S*)-L16, Related to Figure 8a

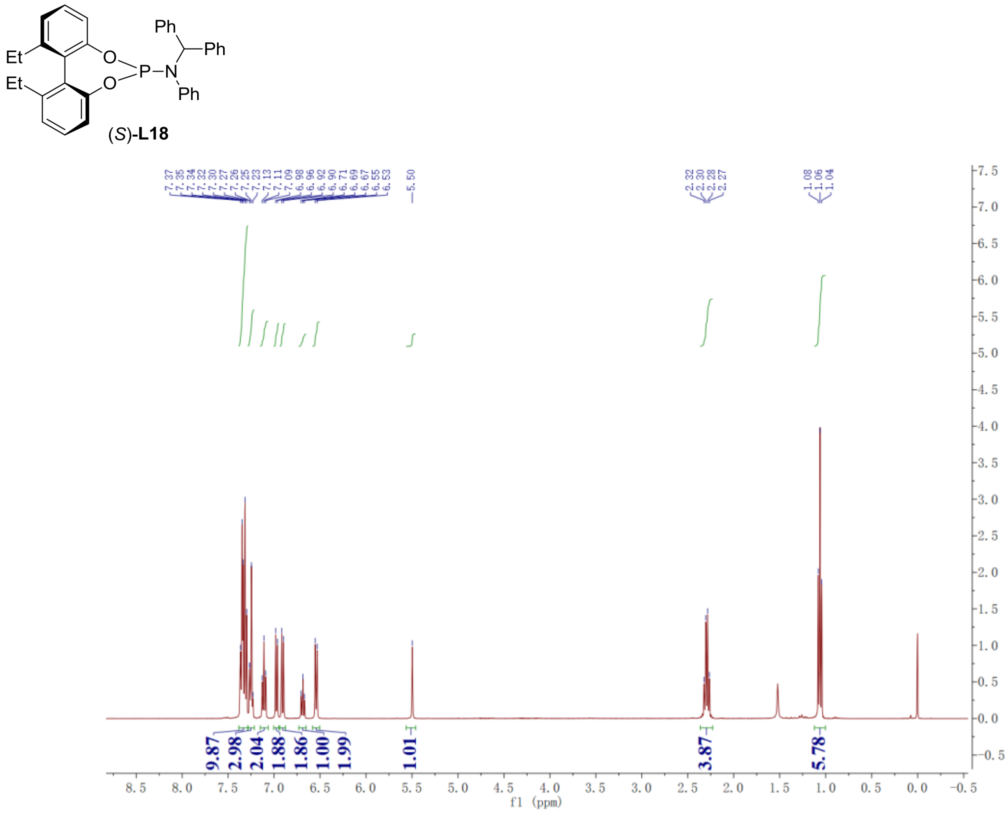

Figure S194. ^{1}H NMR spectrum of (*S*)-L18, Related to Figure 8a

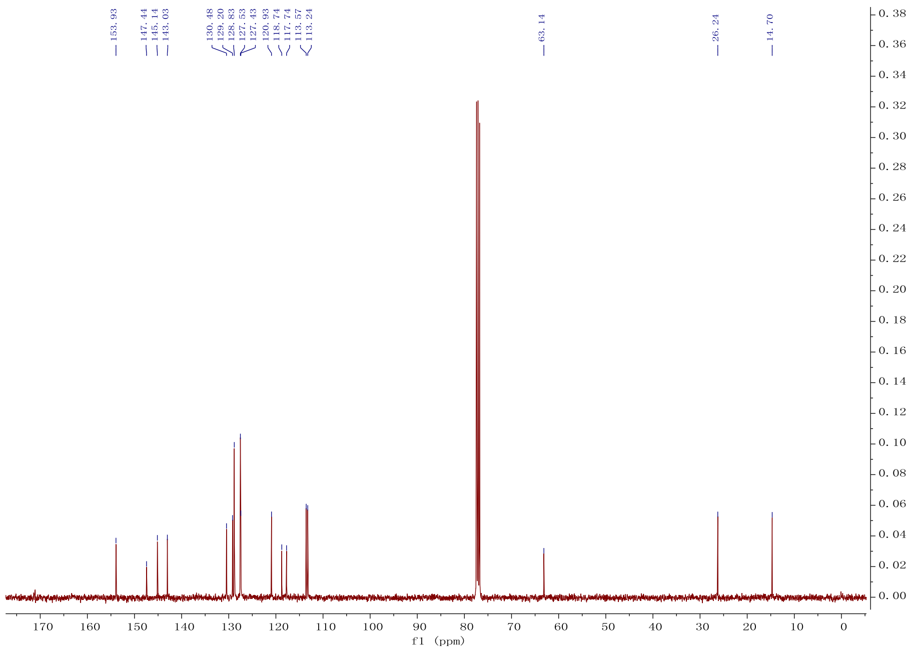

Figure S195. ^{13}C NMR spectrum of (*S*)-L18, Related to Figure 8a

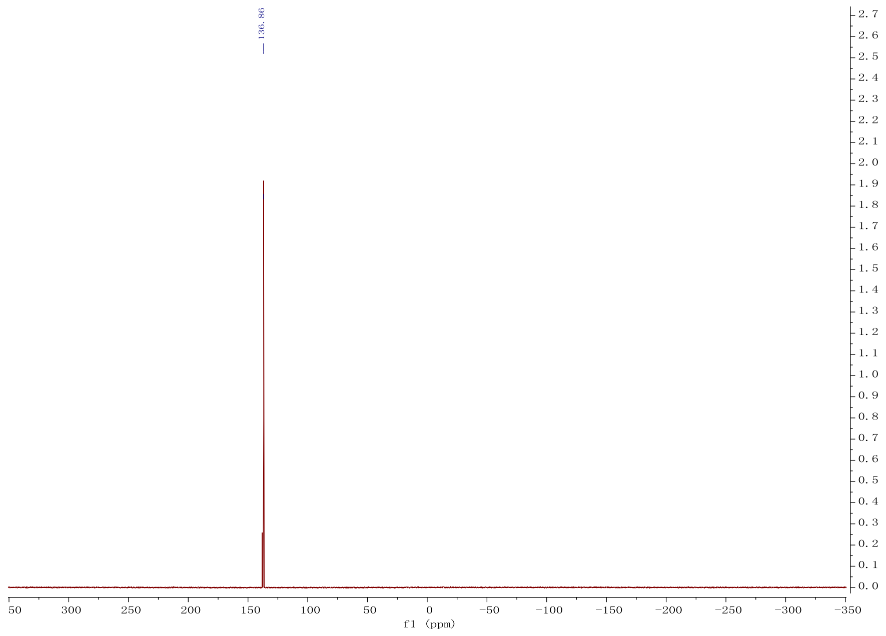

Figure S196. ^{19}F NMR spectrum of (*S*)-L18, Related to Figure 8a

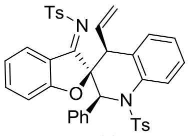

32

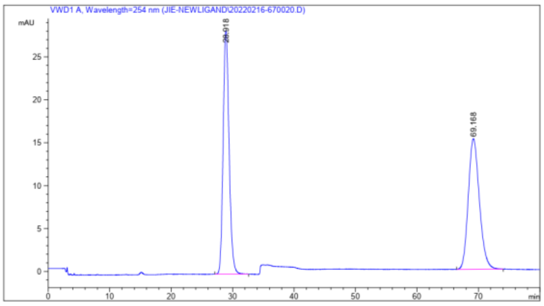

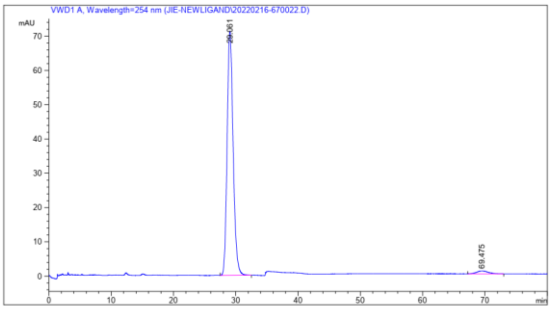

Figure S197. HPLC traces of 32, Related to Figure 8c

Signal 1: VWD1 A, Wavelength=254 nm

Peak #	RetTime [min]	Type	Width [min]	Area mAU *s	Height [mAU]	Area %
1	28.918	BB	1.0304	1902.83435	28.35816	49.9660
2	69.168	BB	1.8868	1905.42725	15.20878	50.0340
Totals :				3808.26160	43.56694	

Signal 1: VWD1 A, Wavelength=254 nm

Peak #	RetTime [min]	Type	Width [min]	Area mAU *s	Height [mAU]	Area %
1	29.061	BB	1.0259	4754.27344	71.25988	97.8079
2	69.475	BB	1.5139	106.55446	8.35630e-1	2.1921
Totals :				4860.82790	72.09551	

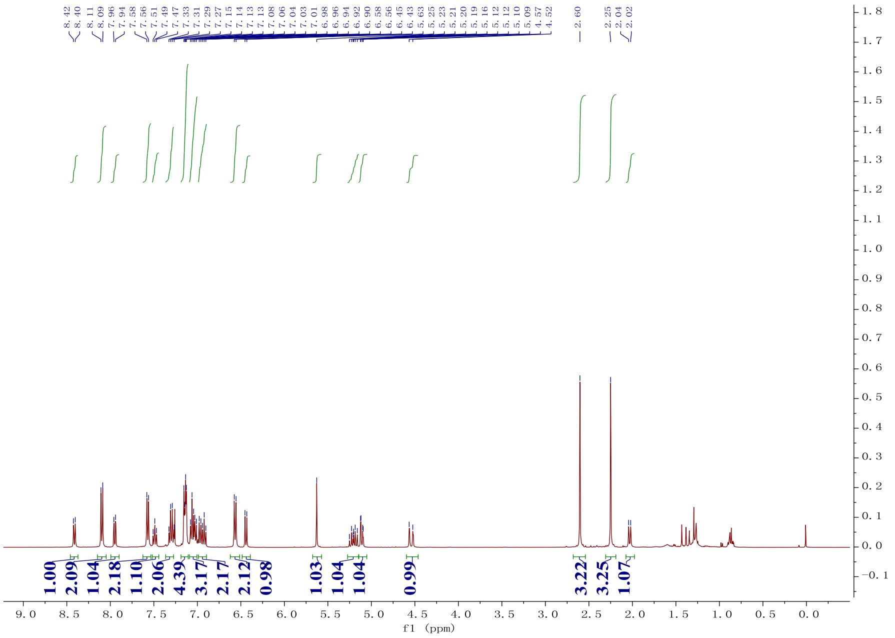

Figure S198. ^{1}H NMR spectrum of 32, Related to Figure 8c

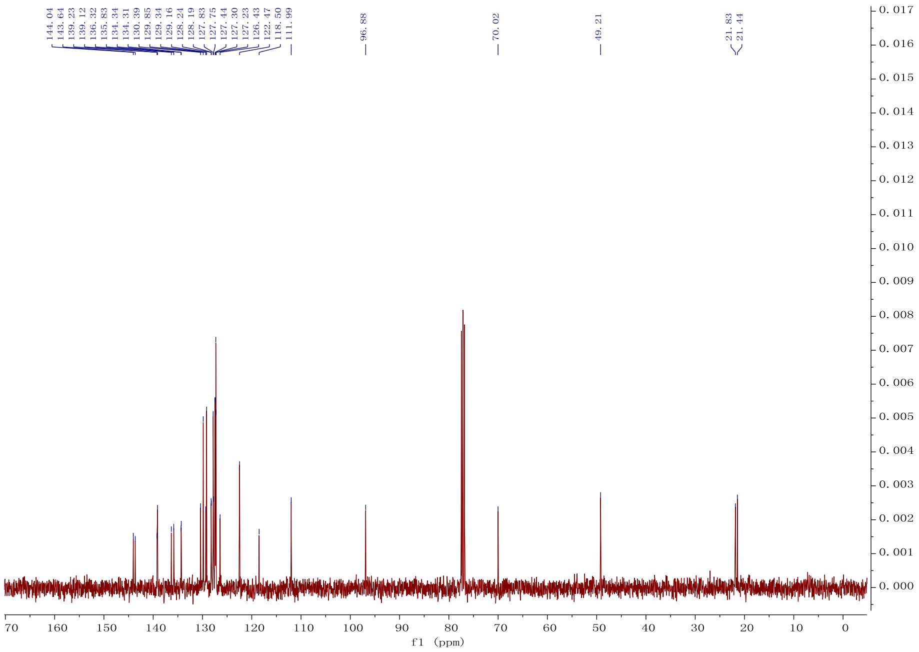

Figure S199. ^{13}**C NMR spectrum of 32, Related to Figure 8c**

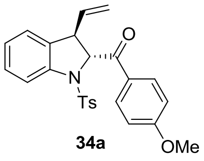

34a

HPLC: enantiomeric excess of **34a** (ee = 88%) was determined by high-performance liquid chromatography (HPLC) using a chiral stationary phase (AD-H column, flow rate = 1.0 mL/ min, eluent: hexane/ isopropanol = 80/20, 254 nm absorbance), retention times: minor enantiomer (t_R = 80.44 min), major enantiomer (t_R = 27.49 min).

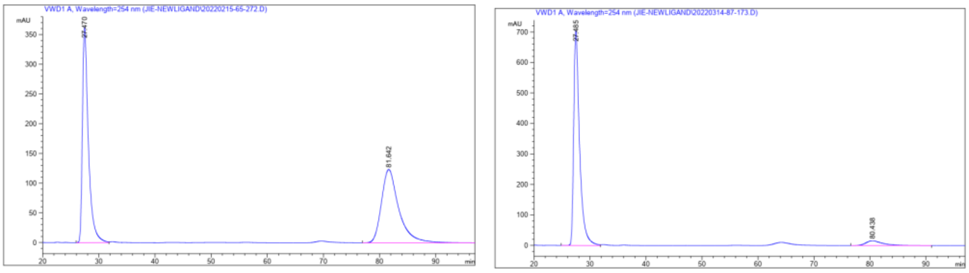

Figure S200. HPLC traces of 34a, Related to Figure 8d

Signal 1: VWD1 A, Wavelength=254 nm

Peak #	RetTime [min]	Type	Width [min]	Area mAU *s	Height [mAU]	Area %
1	27.470	BV	1.0771	2.66595e4	362.21869	50.0862
2	81.642	BB	3.1399	2.65678e4	123.44628	49.9138
Totals :				5.32273e4	485.66497	

Signal 1: VWD1 A, Wavelength=254 nm

Peak #	RetTime [min]	Type	Width [min]	Area mAU *s	Height [mAU]	Area %
1	27.485	BV	1.0653	5.12773e4	703.98627	94.1151
2	80.438	BB	2.8810	3206.31689	15.34100	5.8849
Totals :				5.44836e4	719.32727	

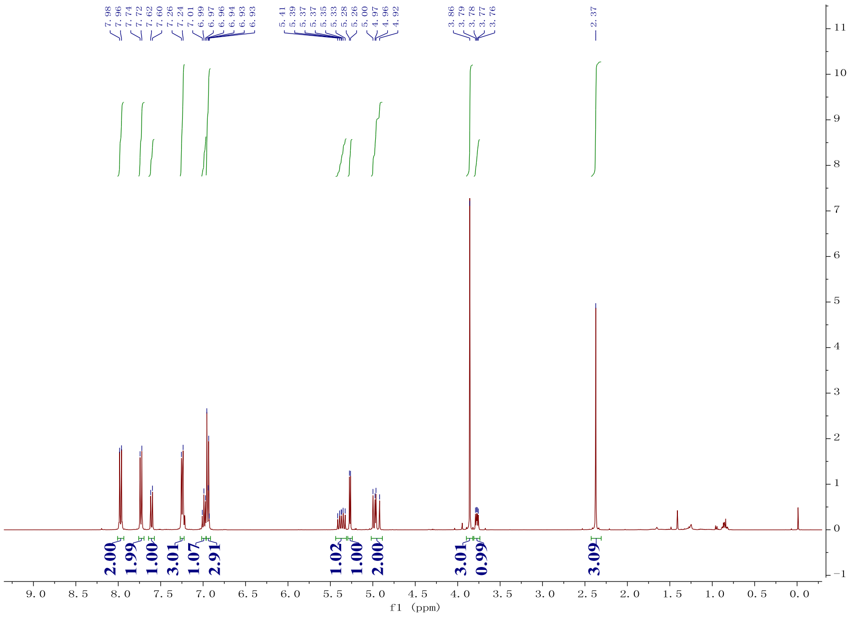

Figure S201. ^{1}H NMR spectrum of 34a, Related to Figure 8d

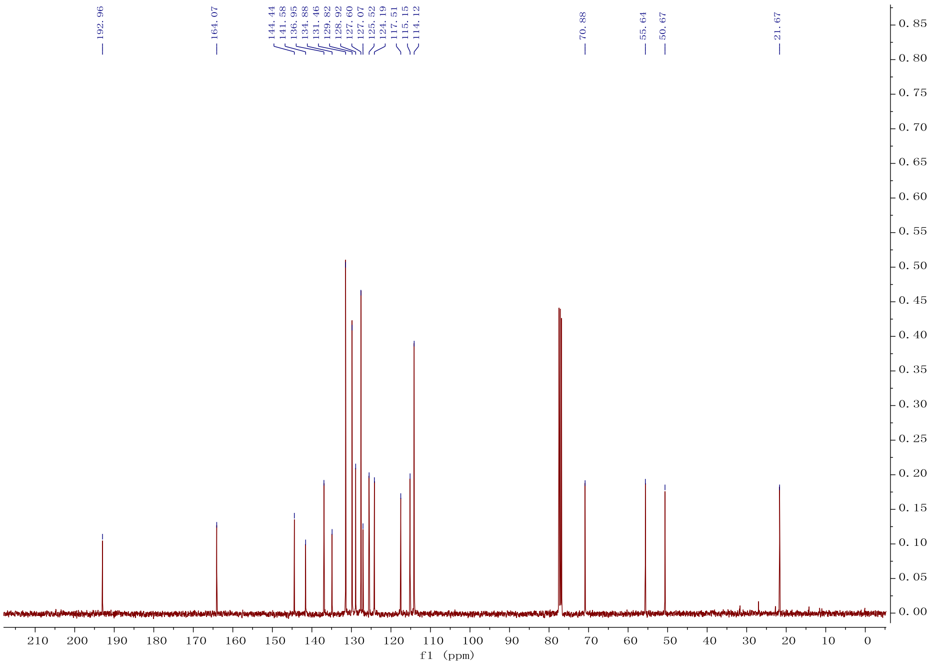

Figure S202. ^{13}C NMR spectrum of 34a, Related to Figure 8d

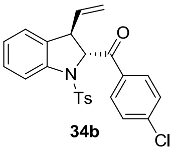

34b

HPLC: enantiomeric excess of **34b** (ee = 94%) was determined by high-performance liquid chromatography (HPLC) using a chiral stationary phase (AD-H column, flow rate = 1.0 mL/ min, eluent: hexane/ isopropanol = 80/20, 254 nm absorbance), retention times: minor enantiomer (t_R = 37.44 min), major enantiomer (t_R = 17.80 min).

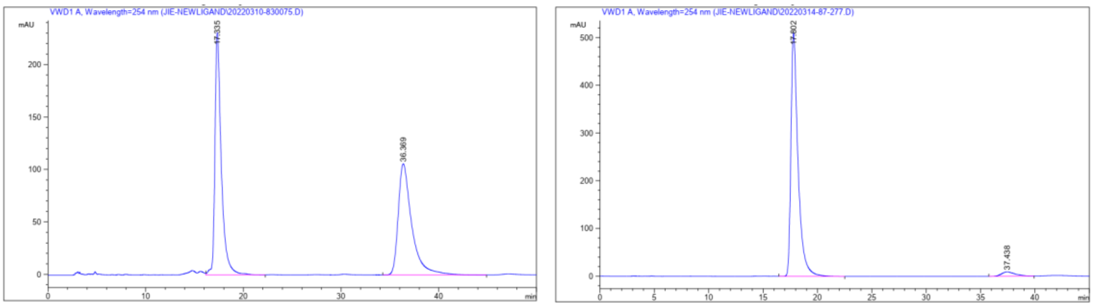

Figure S203. HPLC traces of 34b, Related to Figure 8d

Signal 1: VWD1 A, Wavelength=254 nm

Peak #	RetTime [min]	Type	Width [min]	Area mAU *s	Height [mAU]	Area %
1	17.335	VB	0.6587	1.05049e4	230.97310	50.7707
2	36.369	BB	1.4011	1.01860e4	105.96171	49.2293
Totals :				2.06910e4	336.93481	

Signal 1: VWD1 A, Wavelength=254 nm

Peak #	RetTime [min]	Type	Width [min]	Area mAU *s	Height [mAU]	Area %
1	17.802	BB	0.6647	2.31042e4	510.65353	96.7496
2	37.438	BB	1.2914	776.22021	8.95008	3.2504
Totals :				2.38804e4	519.60361	

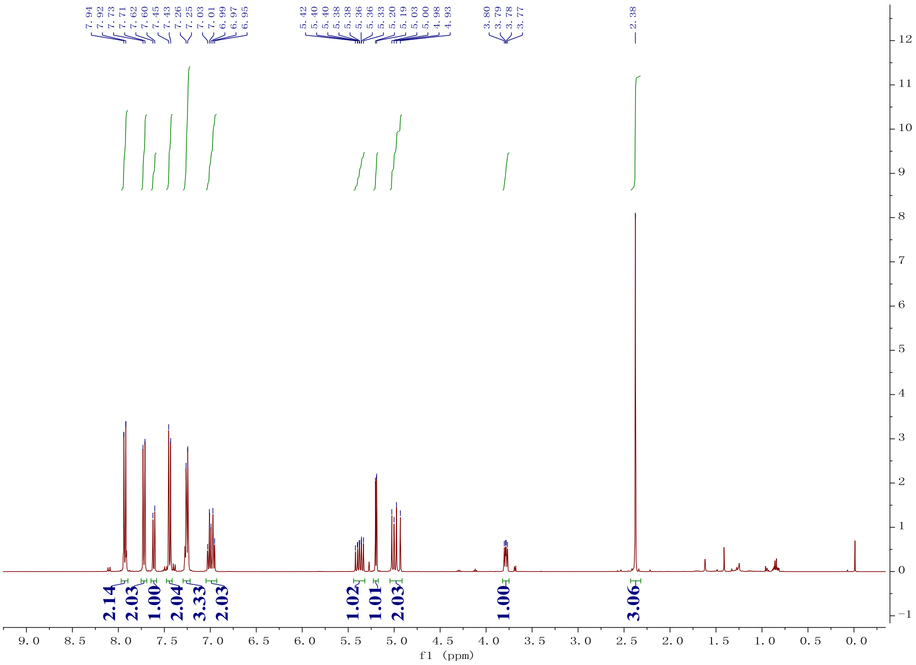

Figure S204. ^{1}H NMR spectrum of 34b, Related to Figure 8d

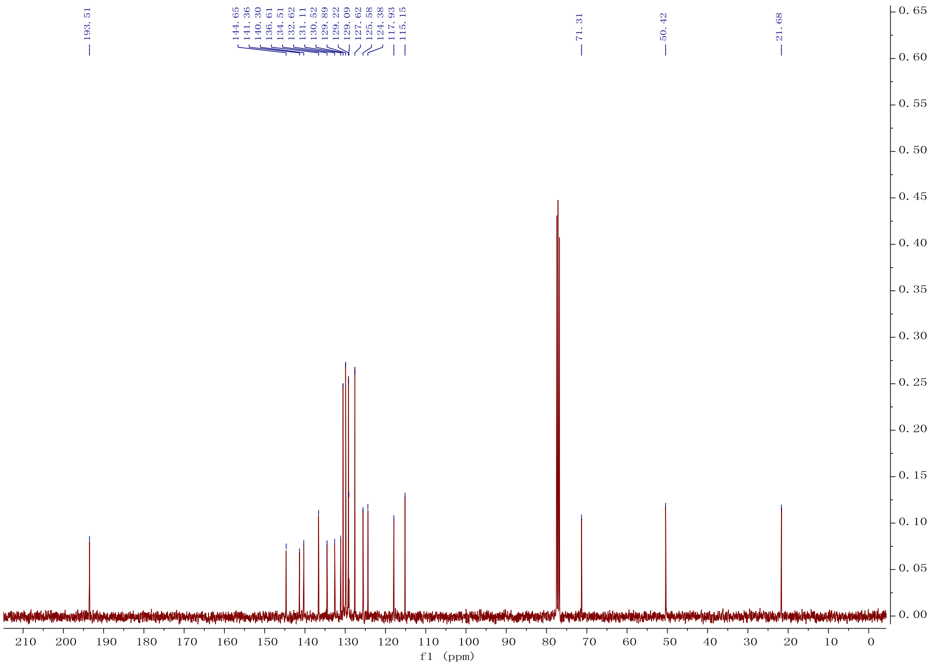

Figure S205. ^{13}C NMR spectrum of 34b, Related to Figure 8d

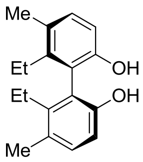

(*S*)-**39**

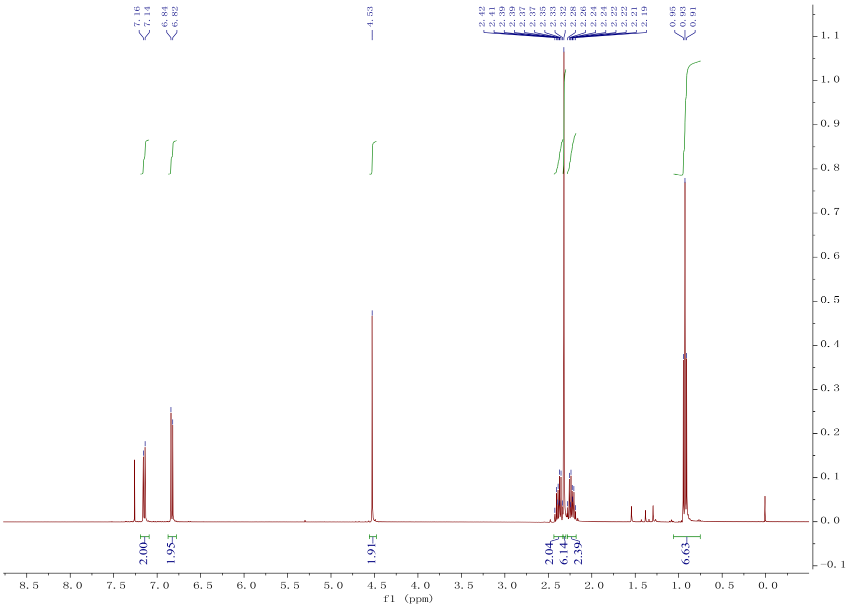

Figure S206. ^{1}H NMR spectrum of (*S*)-39, Related to Figure 9a

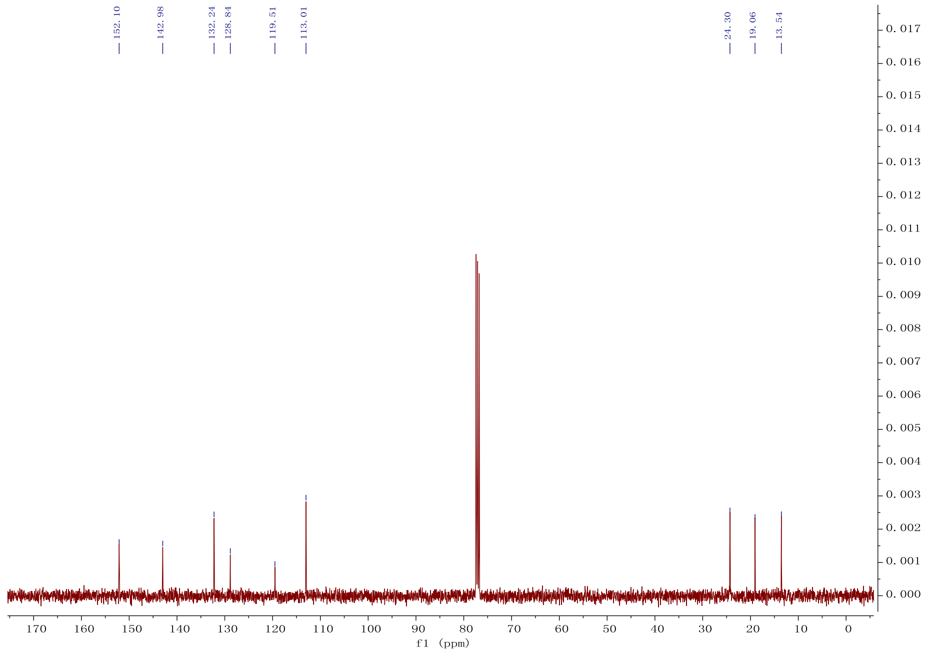

Figure S207. ^{13}C NMR spectrum of (*S*)-39, Related to Figure 9a

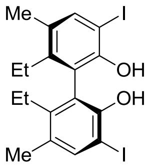

(*S*)-**42**

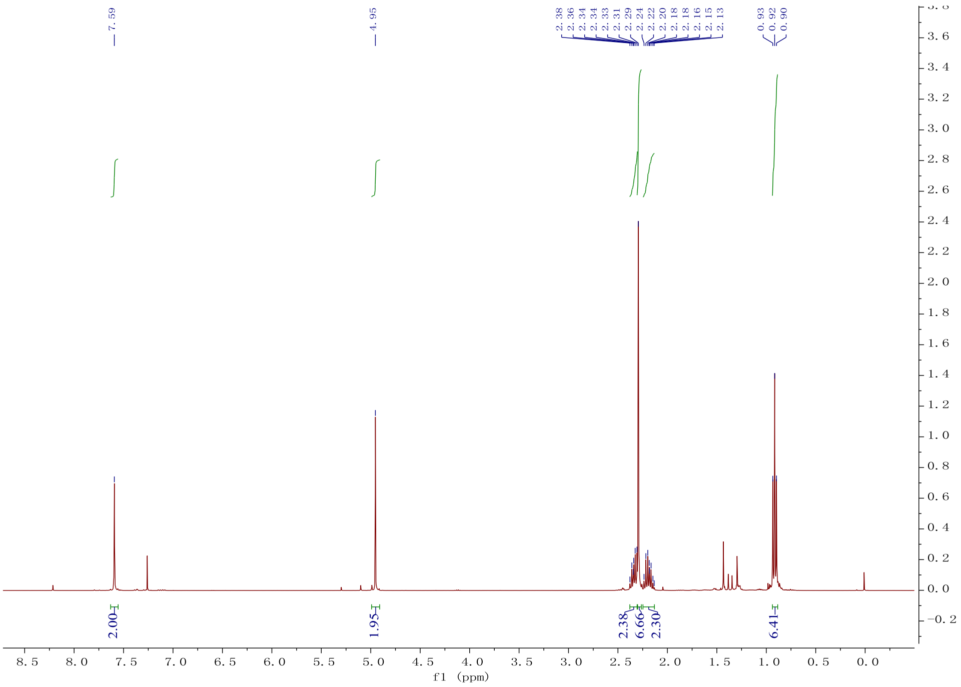

Figure S208. ^{1}H NMR spectrum of (*S*)-42, Related to Figure 9a

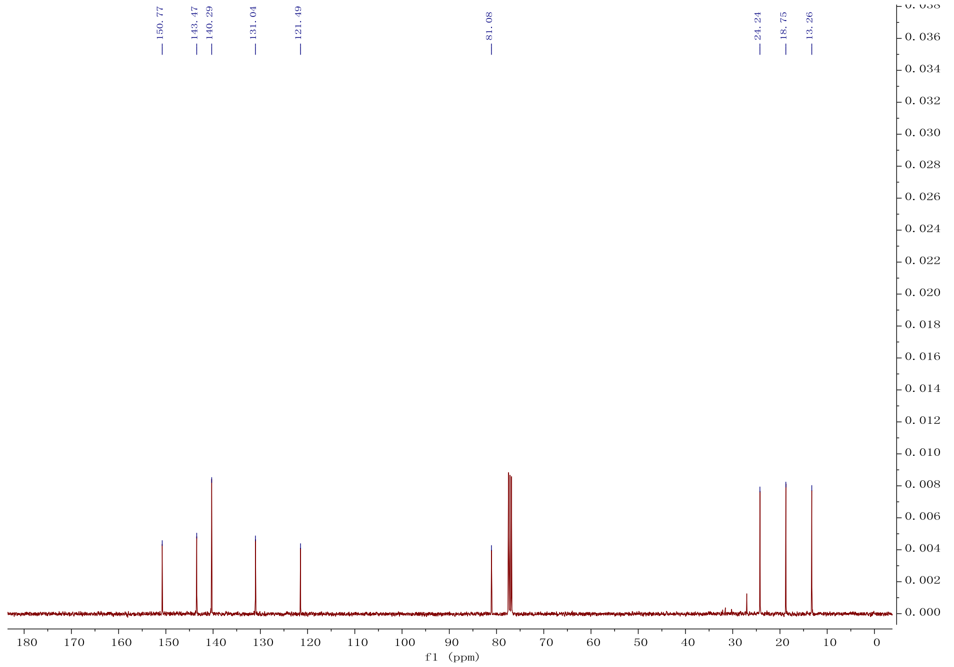

Figure S209. ^{13}C NMR spectrum of (*S*)-42, Related to Figure 9a

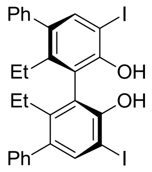

(*S*)-**43**

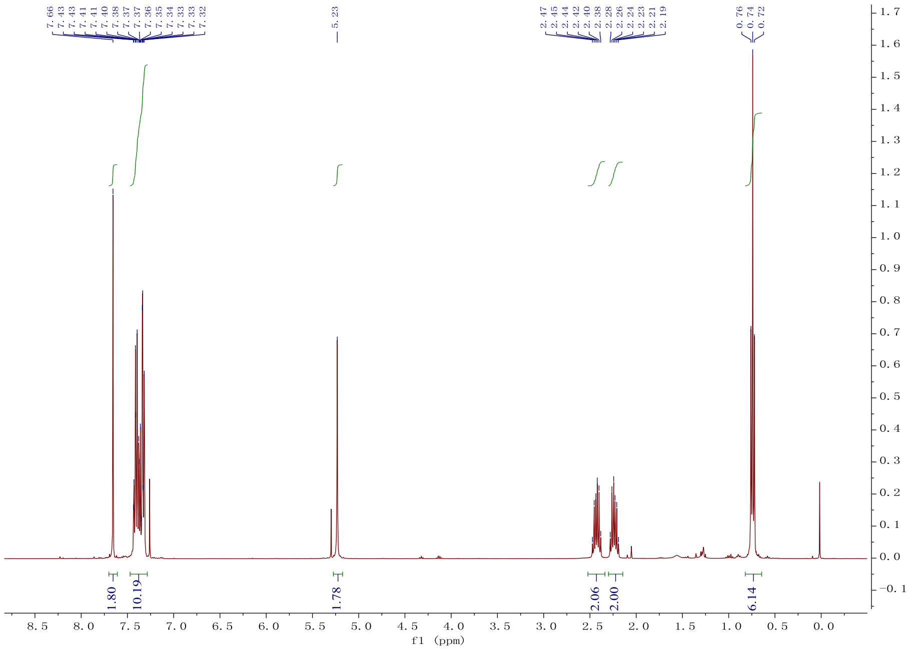

Figure S210. ^{1}H NMR spectrum of (*S*)-43, Related to Figure 9a

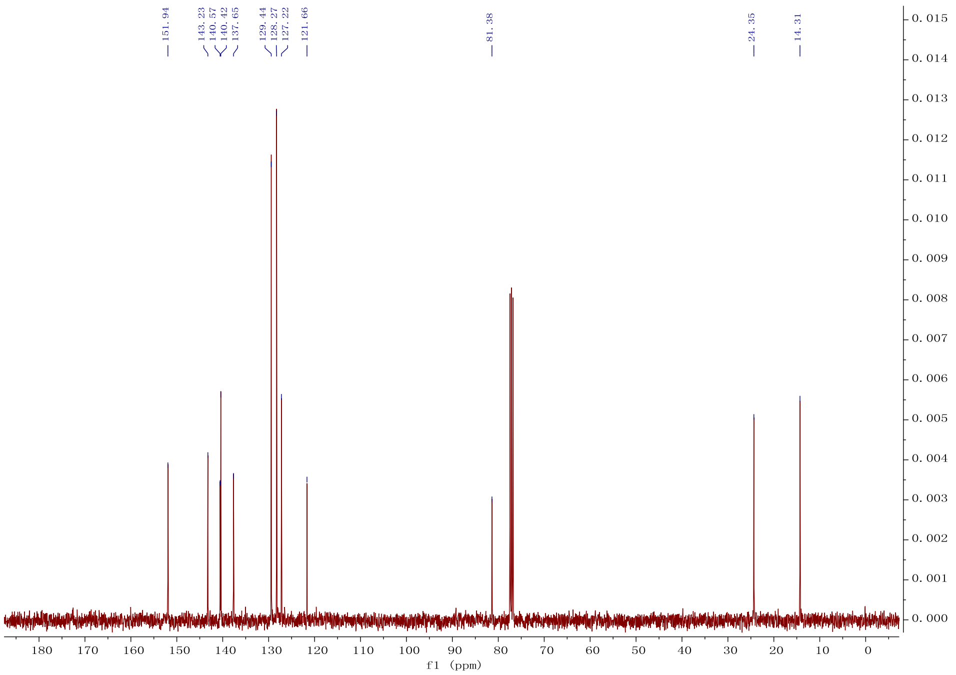

Figure S211. ^{13}C NMR spectrum of (*S*)-43, Related to Figure 9a

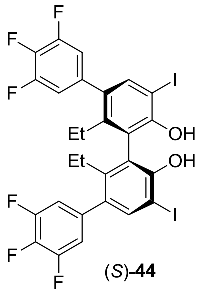

(*S*)-**44**

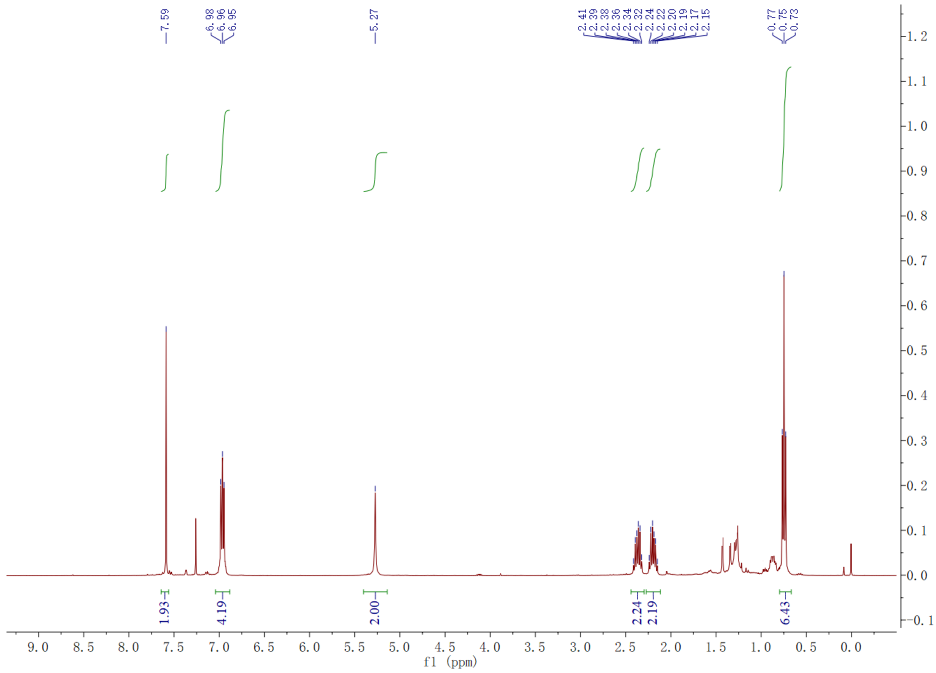

Figure S212. ^{1}H NMR spectrum of (*S*)-44, Related to Figure 9a

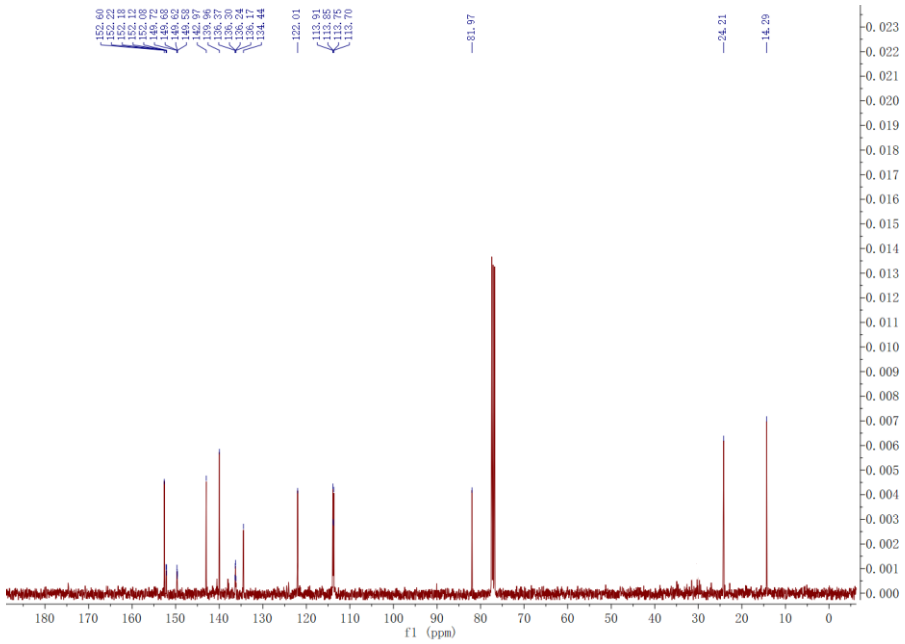

Figure S213. ^{13}C NMR spectrum of (*S*)-44, Related to Figure 9a

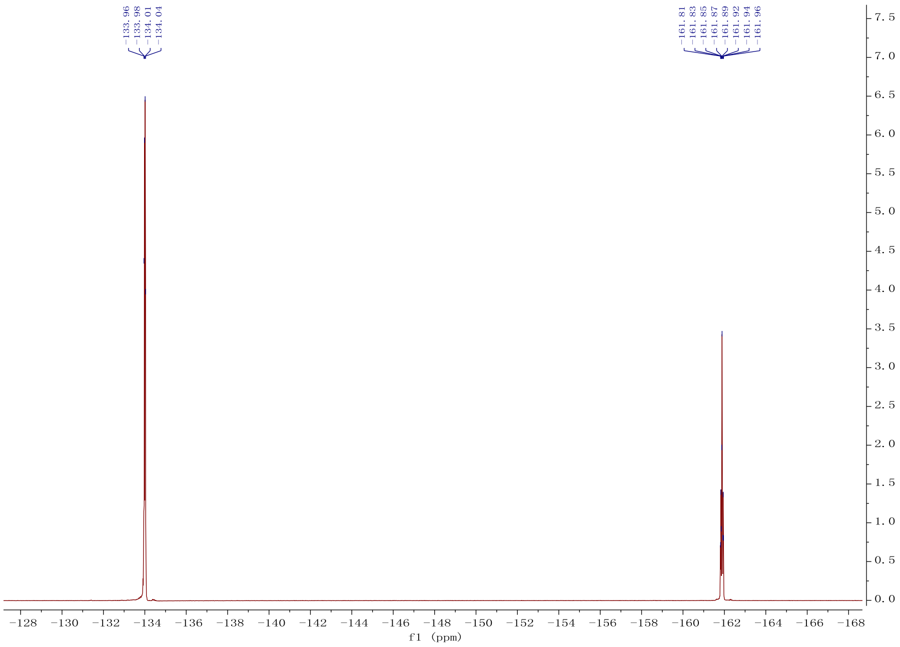

Figure S214. ^{19}F NMR spectrum of (*S*)-44, Related to Figure 9a

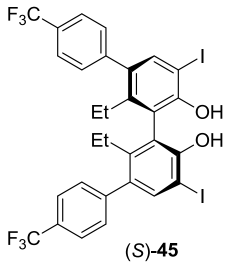

(*S*)-**45**

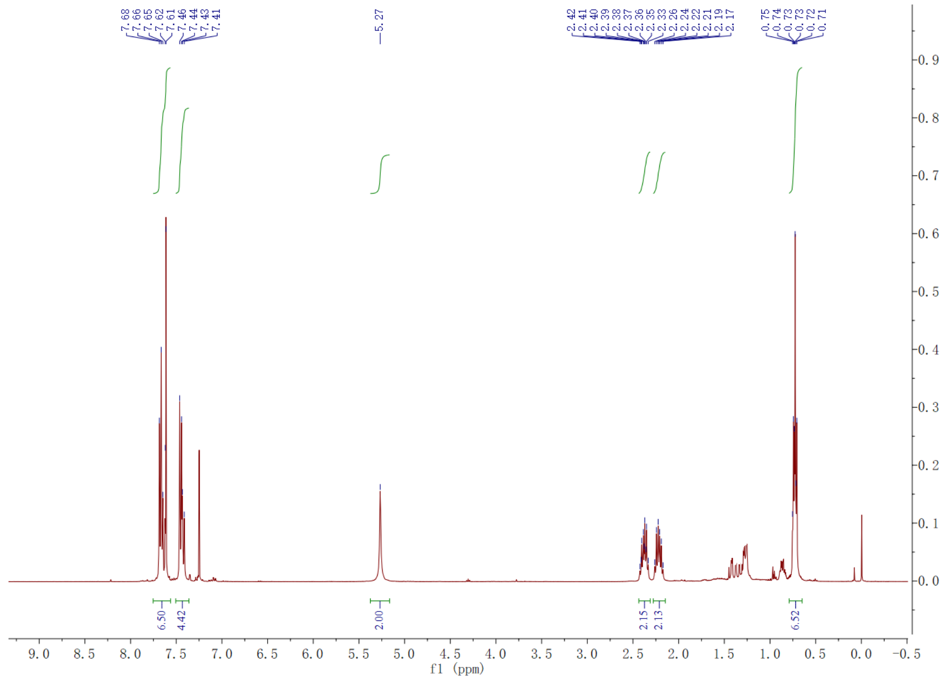

Figure S215. ^{1}H NMR spectrum of (*S*)-45, Related to Figure 9a

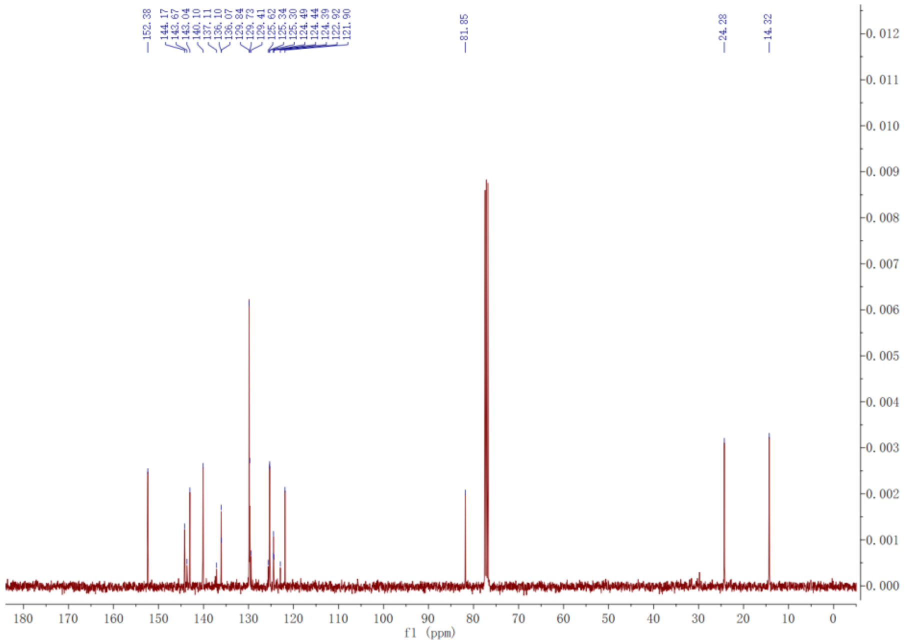

Figure S216. ^{13}C NMR spectrum of (*S*)-45, Related to Figure 9a

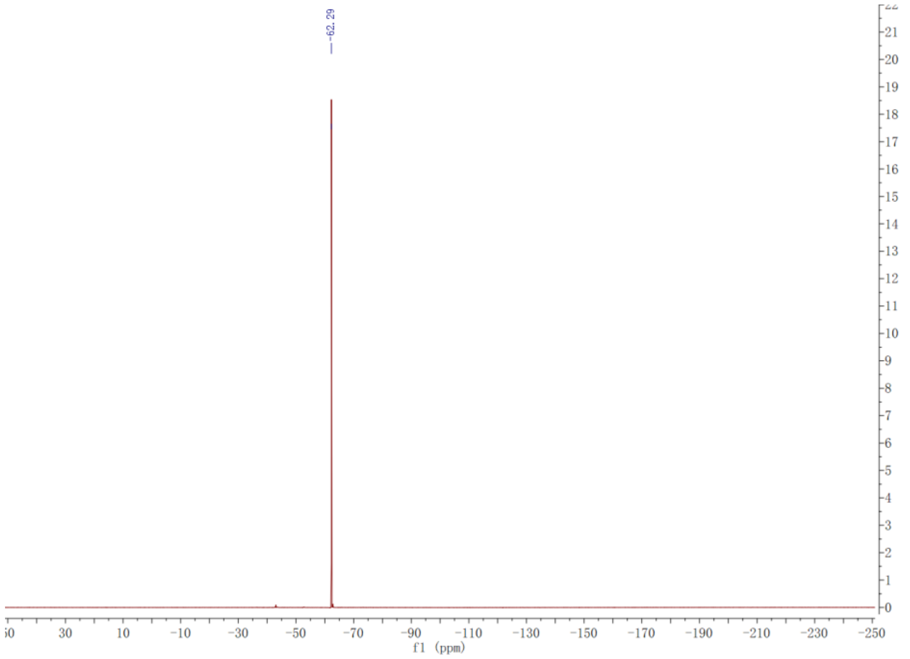

Figure S217. ^{19}F NMR spectrum of (*S*)-45, Related to Figure 9a

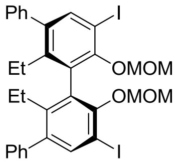

(*S*)-**47**

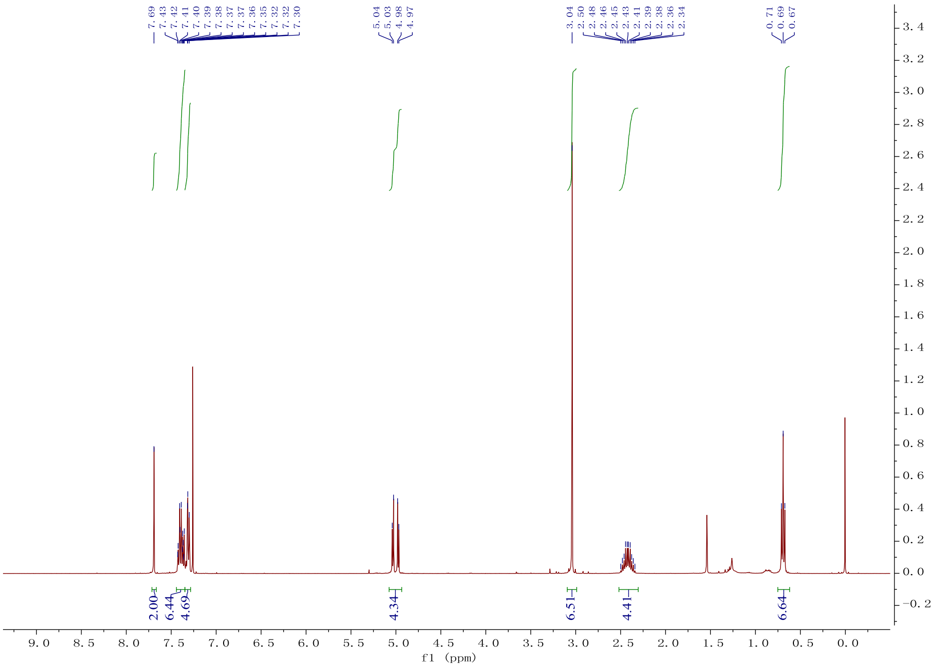

Figure S218. ^{1}H NMR spectrum of (*S*)-47, Related to Figure 9a

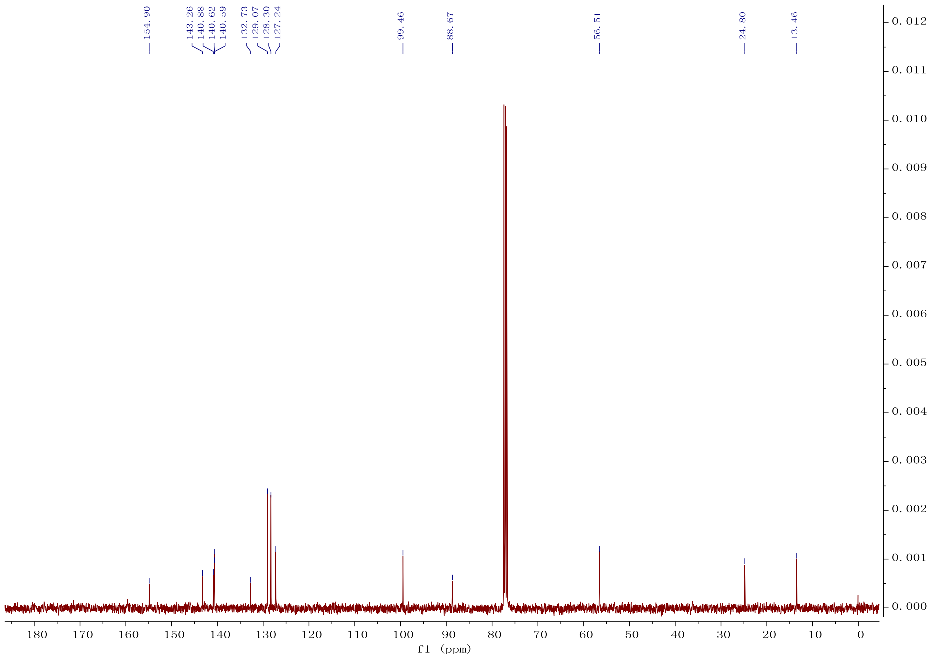

Figure S219. ^{13}C NMR spectrum of (*S*)-47, Related to Figure 9a

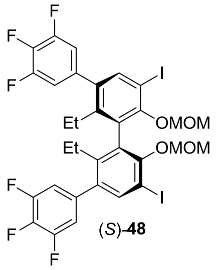

(*S*)-**48**

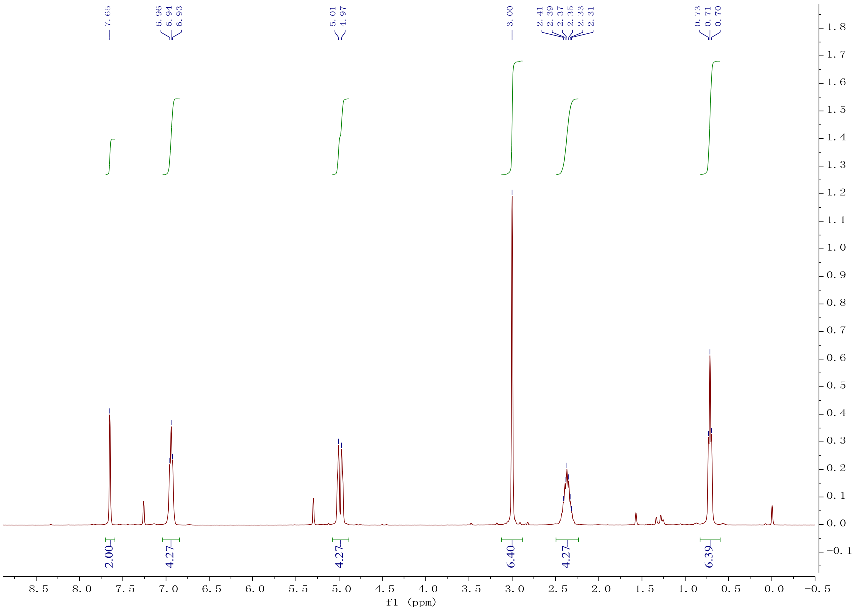

Figure S220. ^{1}H NMR spectrum of (*S*)-48, Related to Figure 9a

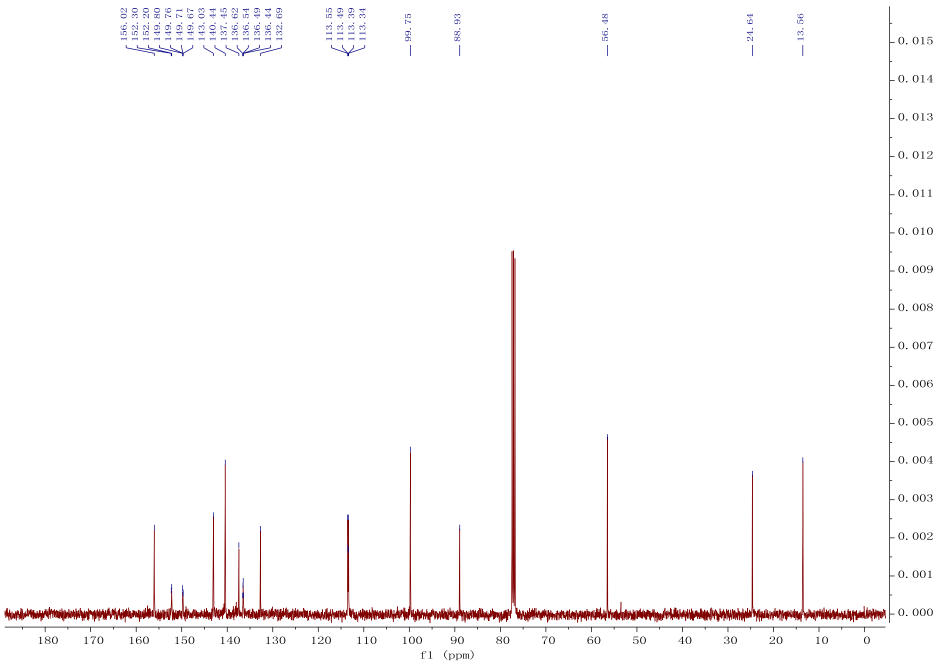

Figure S221. ^{13}C NMR spectrum of (*S*)-48, Related to Figure 9a

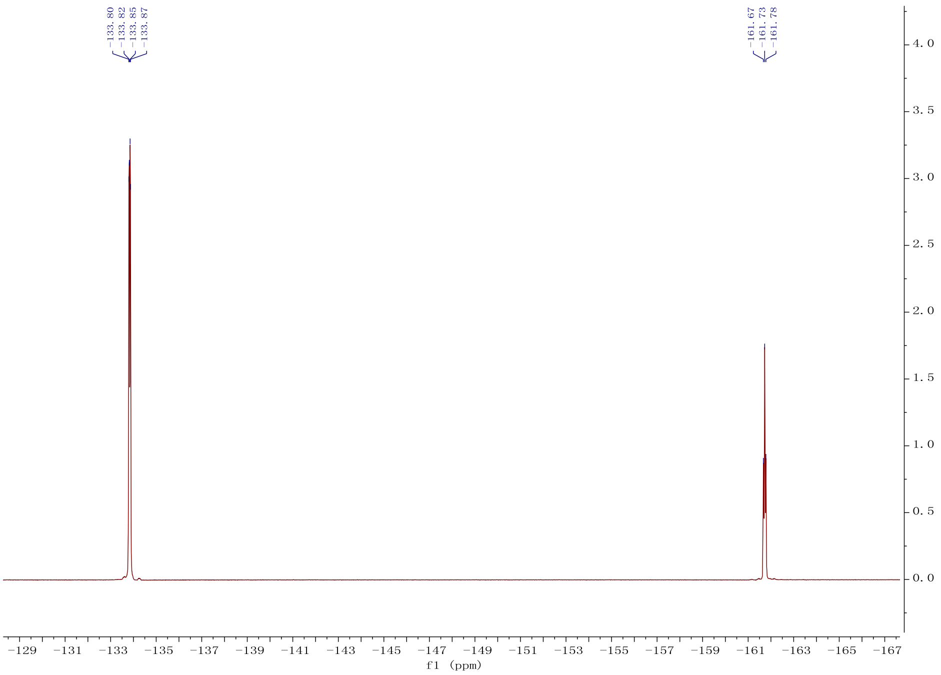

Figure S222. ^{19}F NMR spectrum of (*S*)-48, Related to Figure 9a

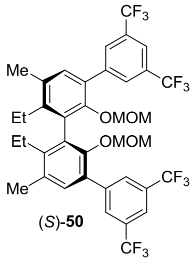

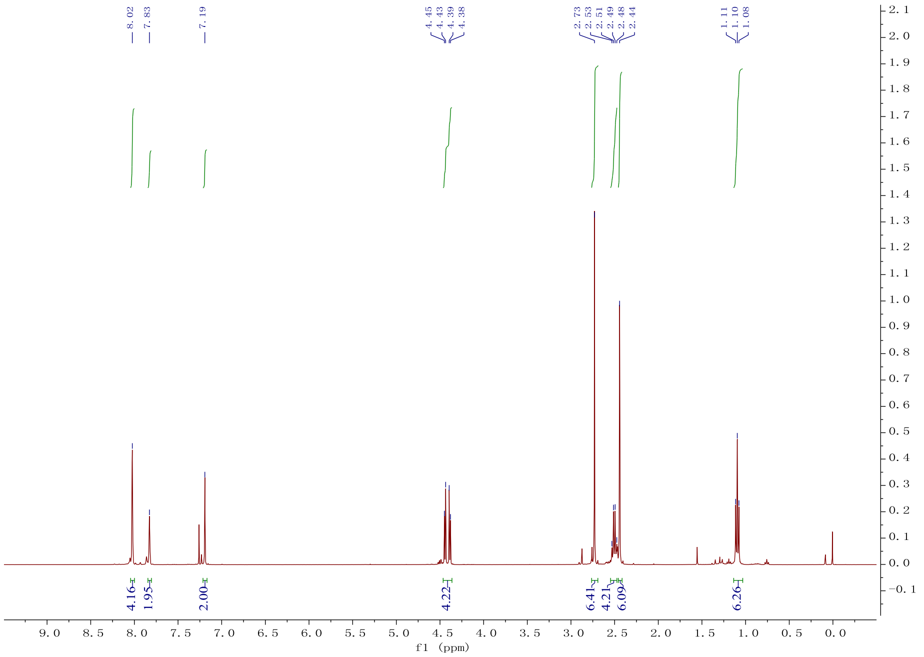

Figure S223. ^{1}H NMR spectrum of (*S*)-50, Related to Figure 9a

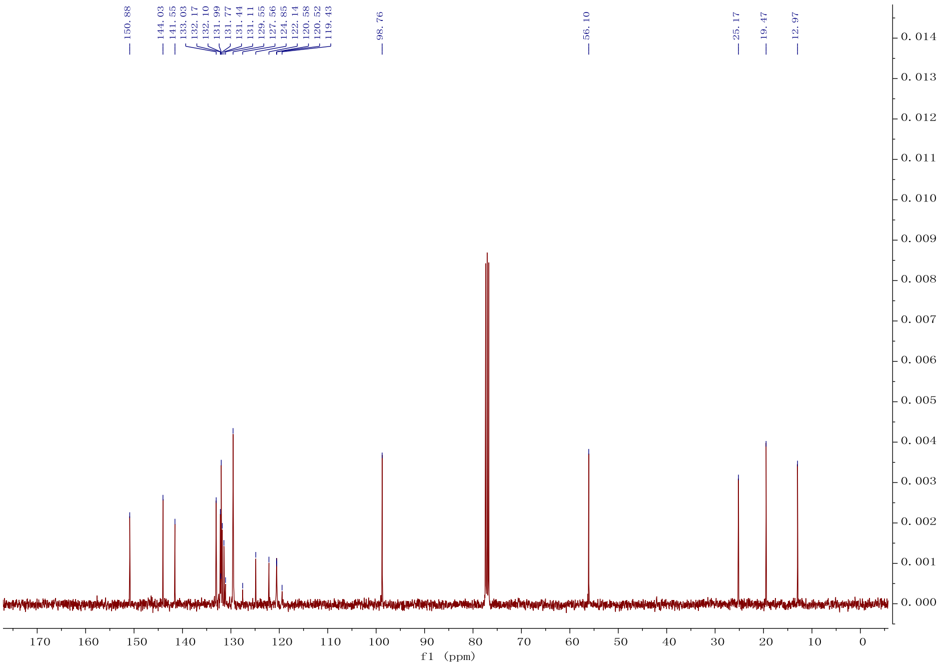

Figure S224. ^{13}C NMR spectrum of (*S*)-50, Related to Figure 9a

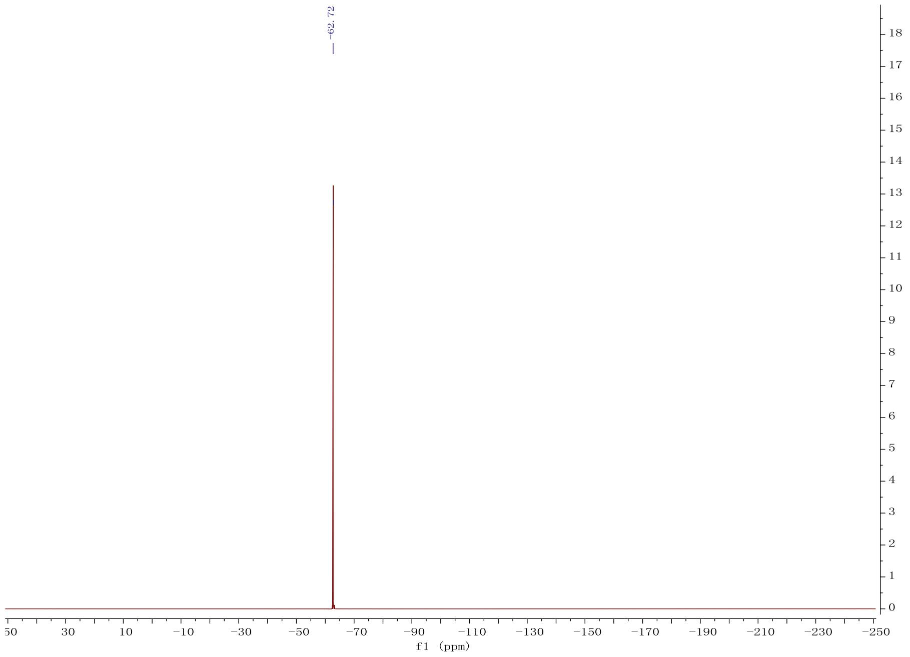

Figure S225. ^{19}F NMR spectrum of (*S*)-50, Related to Figure 9a

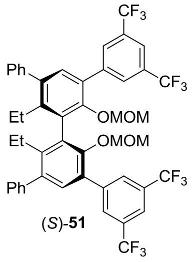

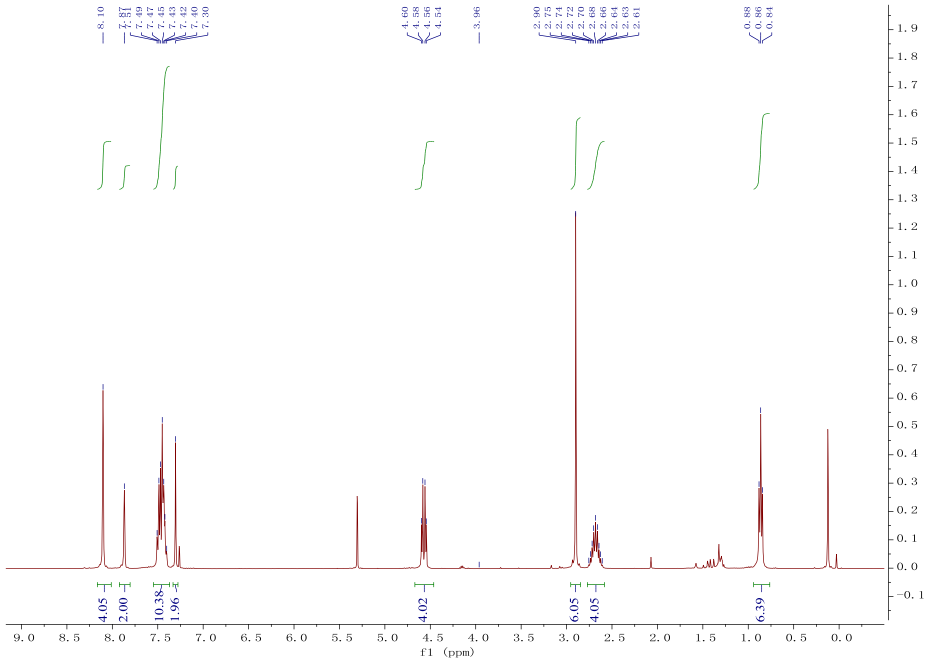

Figure S226. ^{1}H NMR spectrum of (*S*)-51, Related to Figure 9a

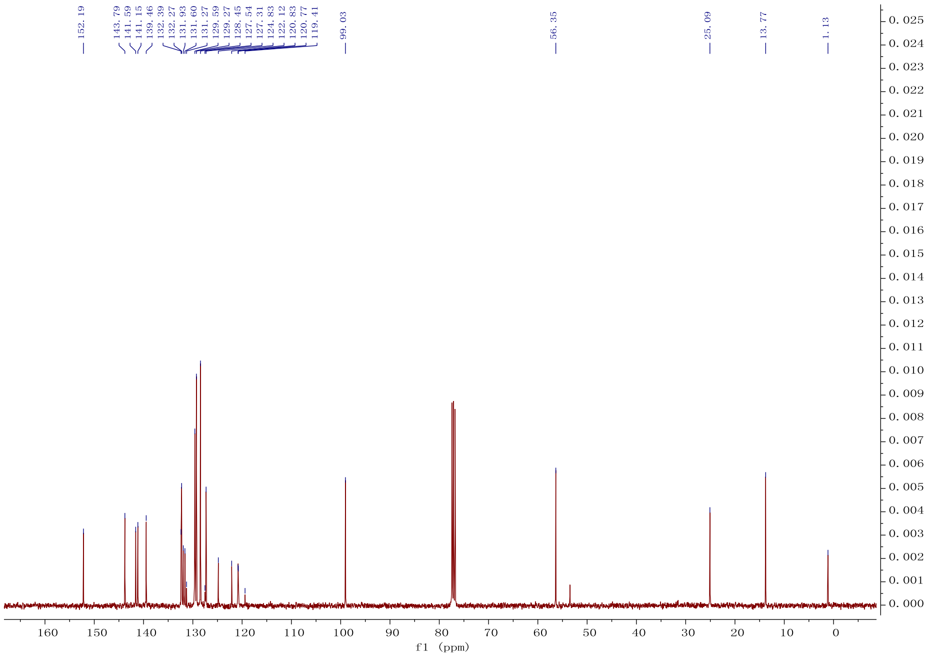

Figure S227. ^{13}C NMR spectrum of (*S*)-51, Related to Figure 9a

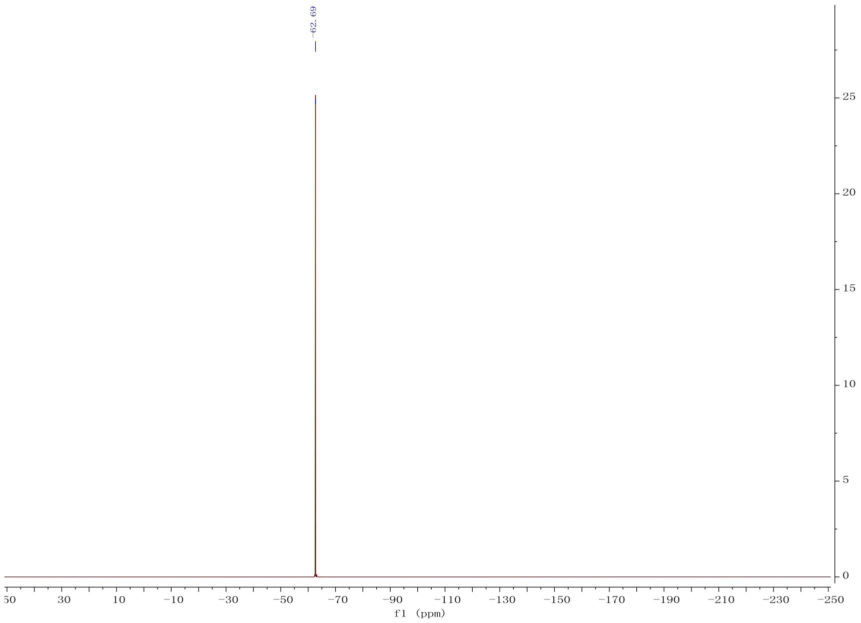

Figure S228. ^{19}F NMR spectrum of (*S*)-51, Related to Figure 9a

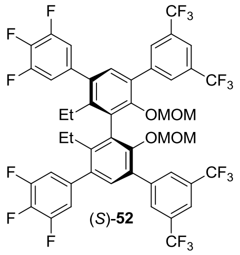

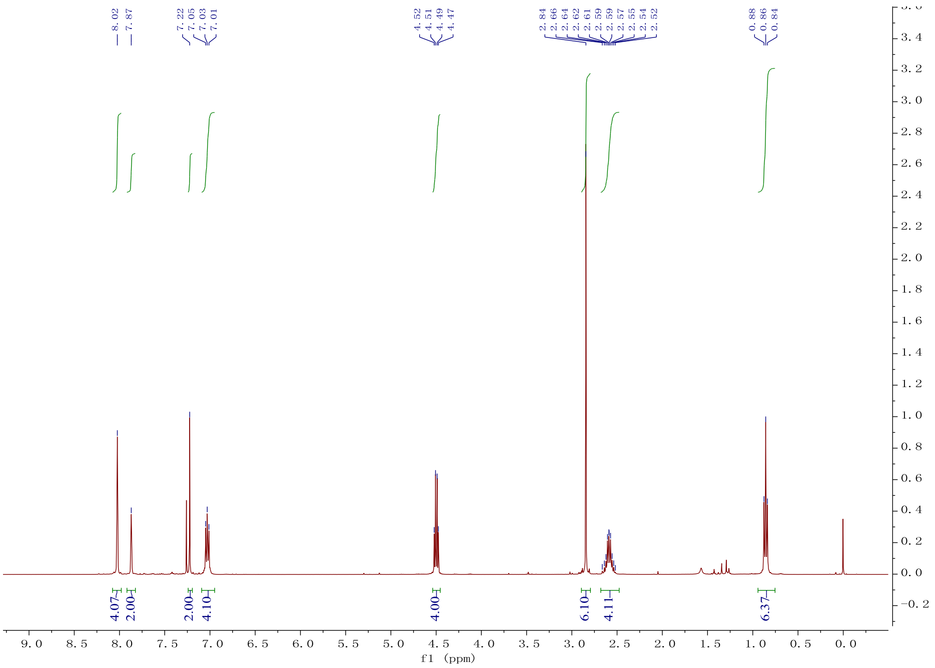

Figure S229. ^{1}H NMR spectrum of (*S*)-52, Related to Figure 9a

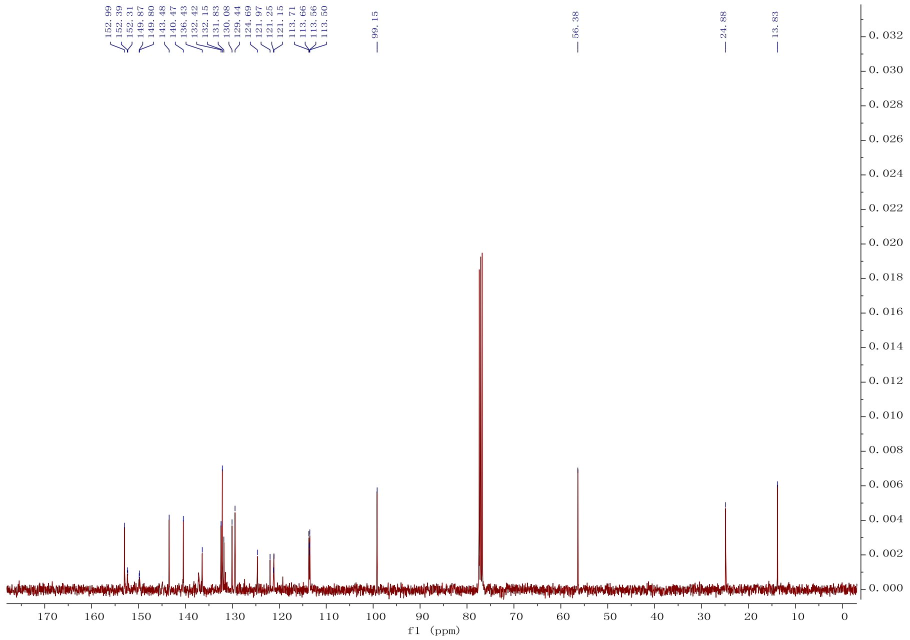

Figure S230. ^{13}C NMR spectrum of (*S*)-52, Related to Figure 9a

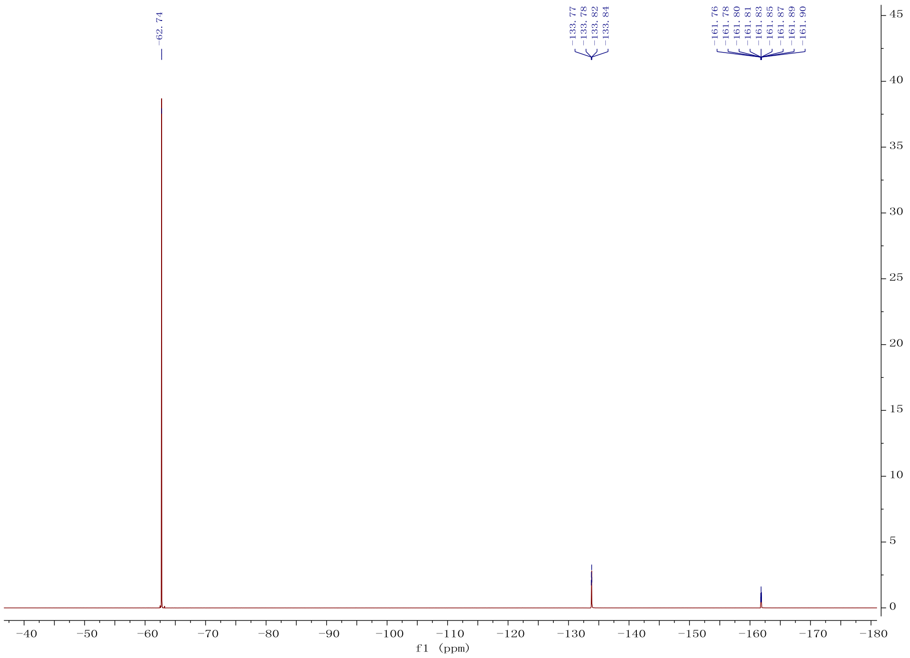

Figure S231. ^{19}F NMR spectrum of (*S*)-52, Related to Figure 9a

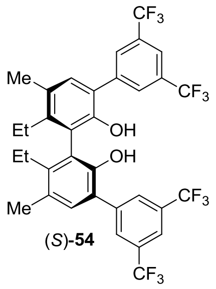

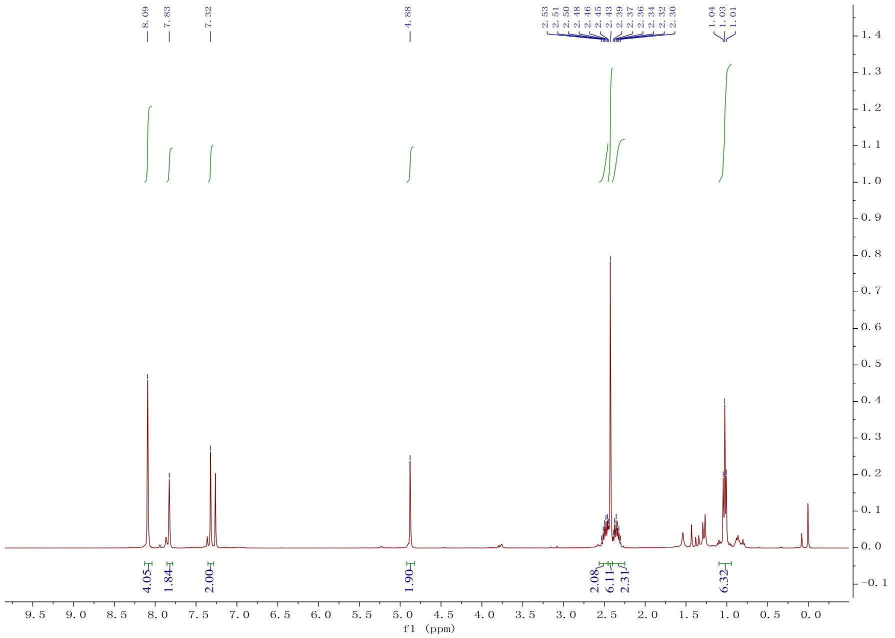

Figure S232. ^{1}H NMR spectrum of (*S*)-54, Related to Figure 9a

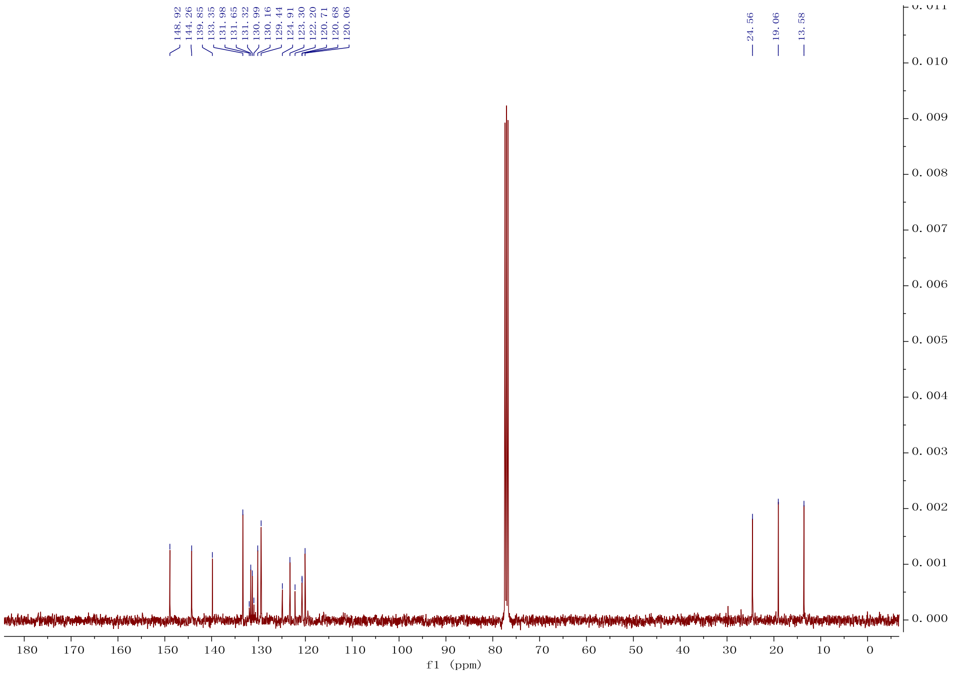

Figure S233. [13]F NMR spectrum of (*S*)-54, Related to Figure 9a

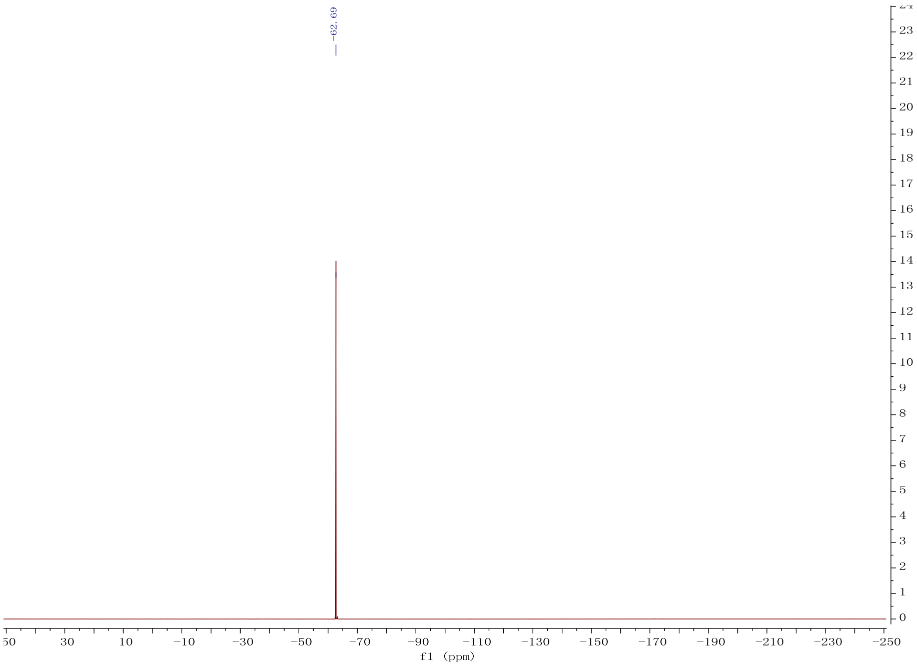

Figure S234. ^{19}F NMR spectrum of (*S*)-54, Related to Figure 9a

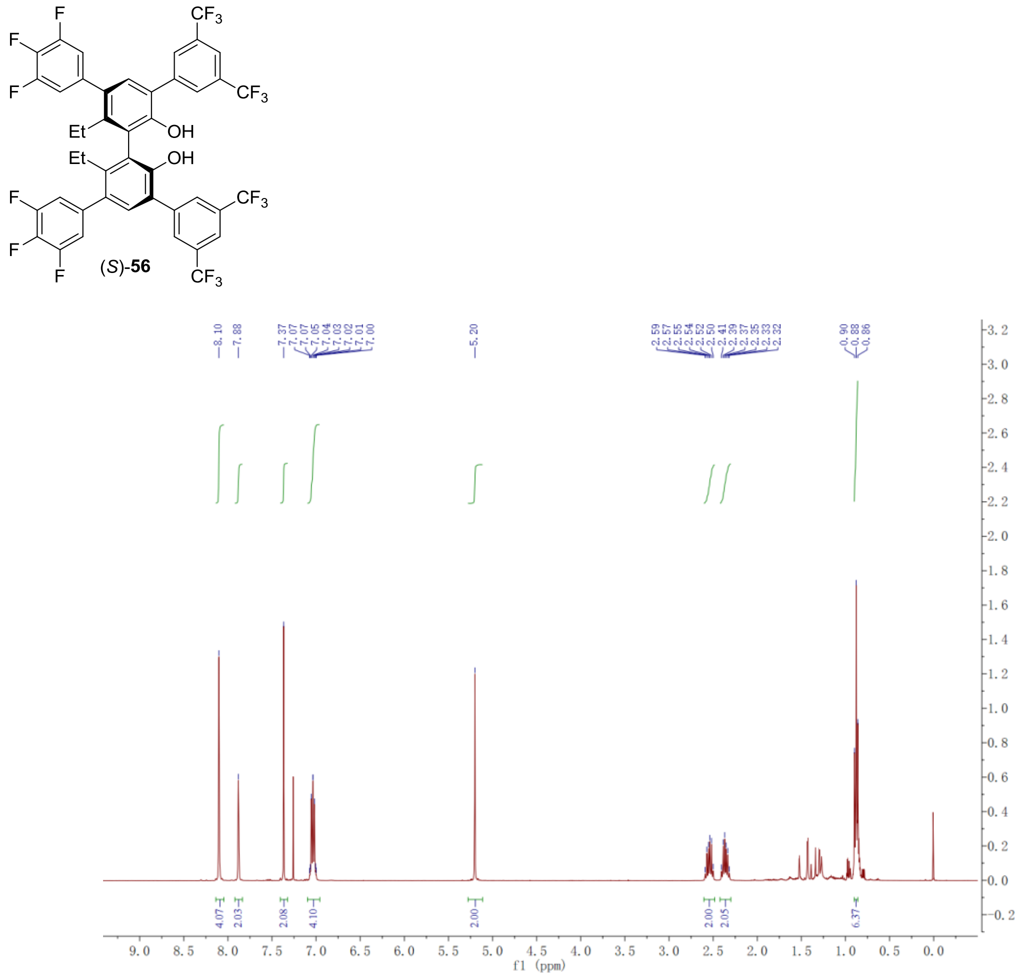

Figure S235. ¹H NMR spectrum of (*S*)-56, Related to Figure 9a

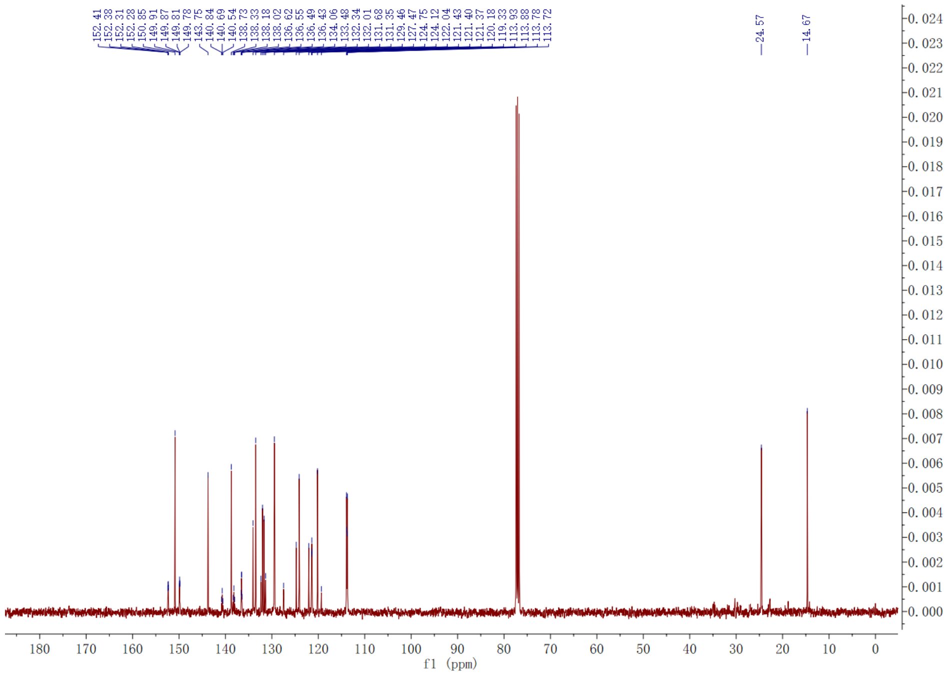

Figure S236. ¹³C NMR spectrum of (*S*)-56, Related to Figure 9a

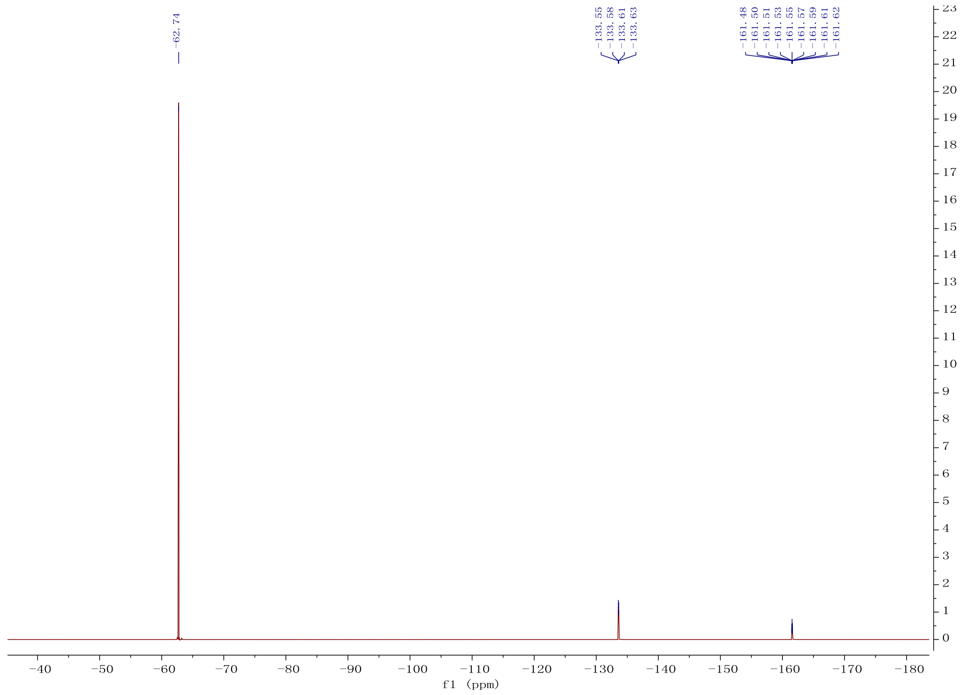

Figure S237. ^{19}F NMR spectrum of (*S*)-56, Related to Figure 9a

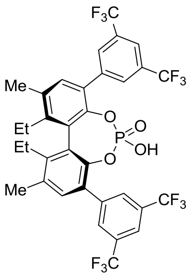

(*S*)-**CPA-2**

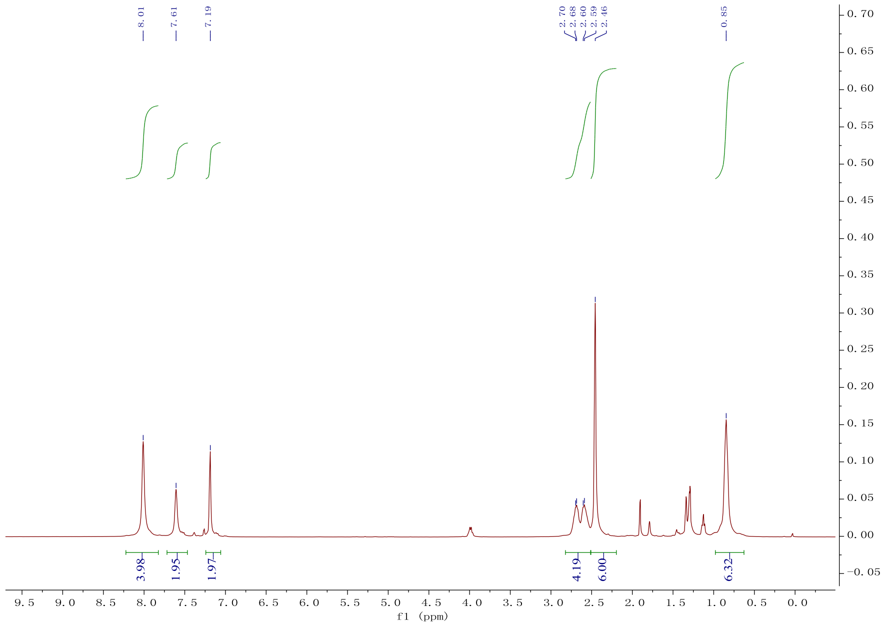

Figure S238. ^{1}H NMR spectrum of (*S*)-CPA-2, Related to Figure 9a

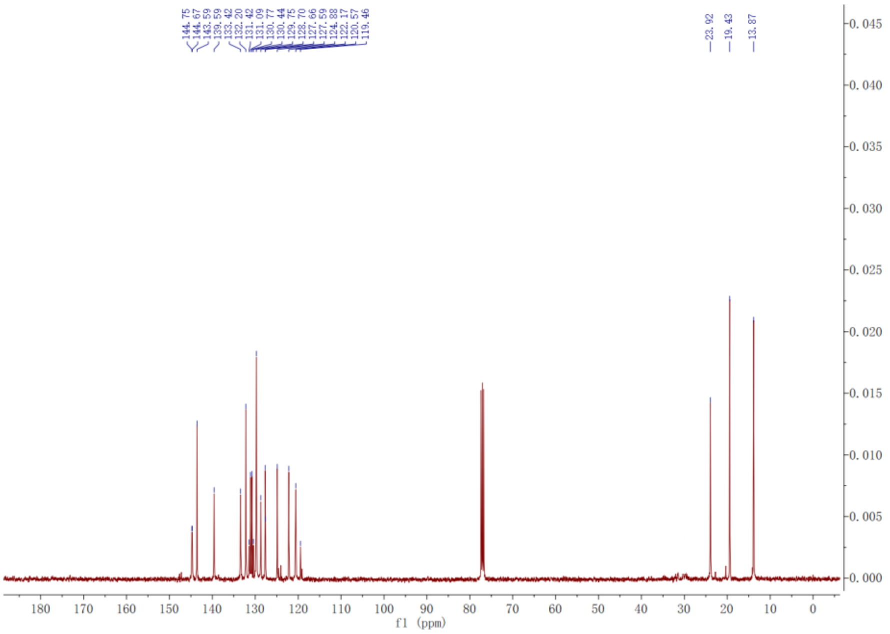

Figure S239. ^{13}C NMR spectrum of (*S*)-CPA-2, Related to Figure 9a

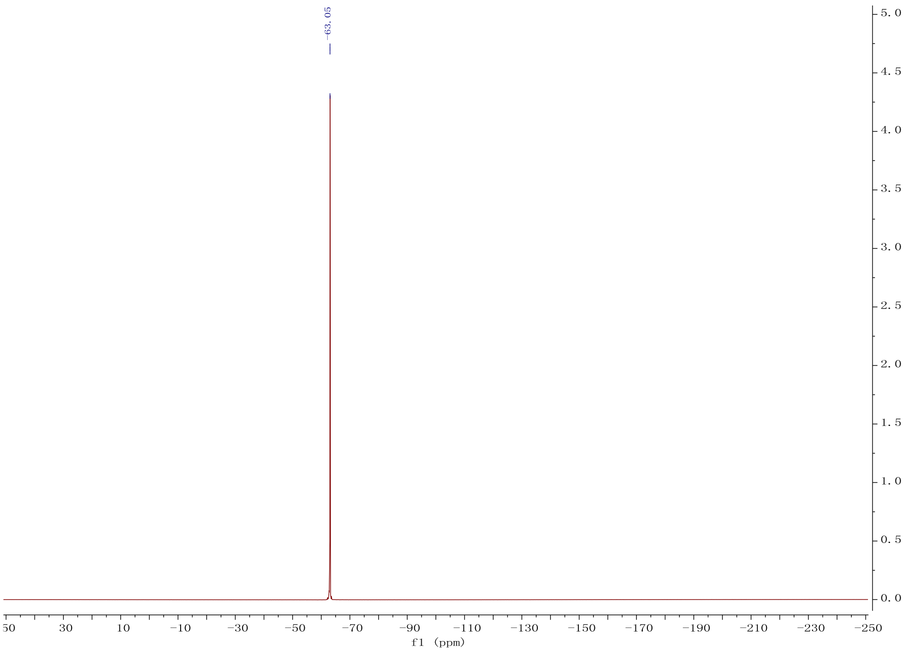

Figure S240. ^{19}F NMR spectrum of (*S*)-CPA-2, Related to Figure 9a

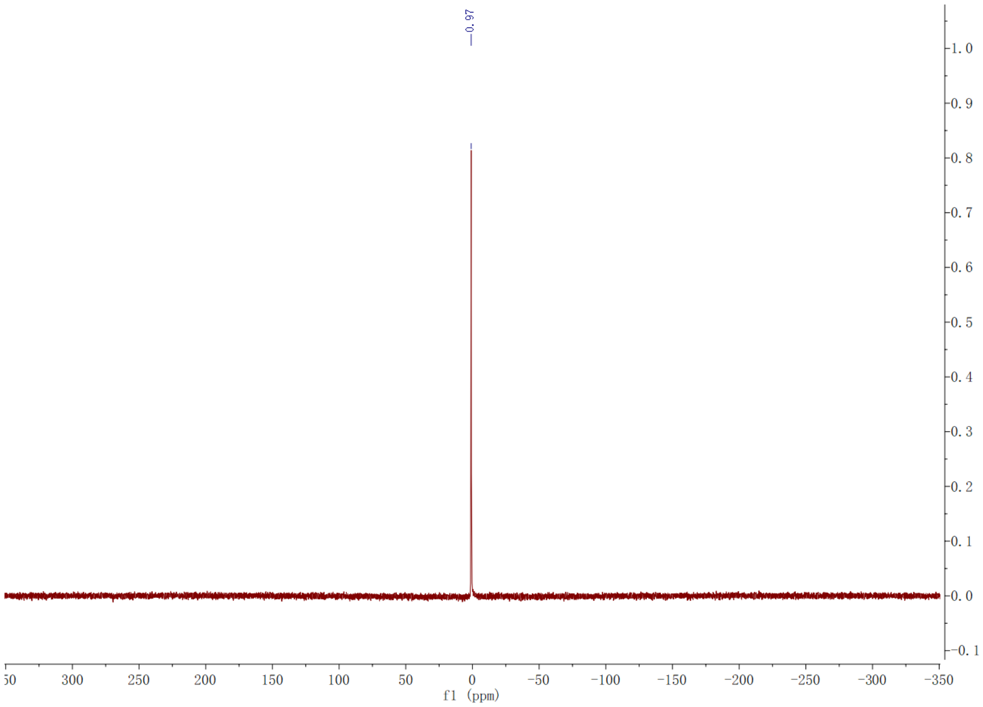

Figure S241. ^{31}P NMR spectrum of (*S*)-CPA-2, Related to Figure 9a

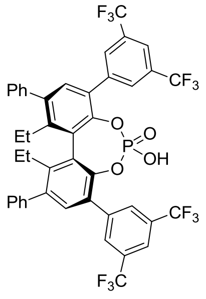

(*S*)-**CPA-3**

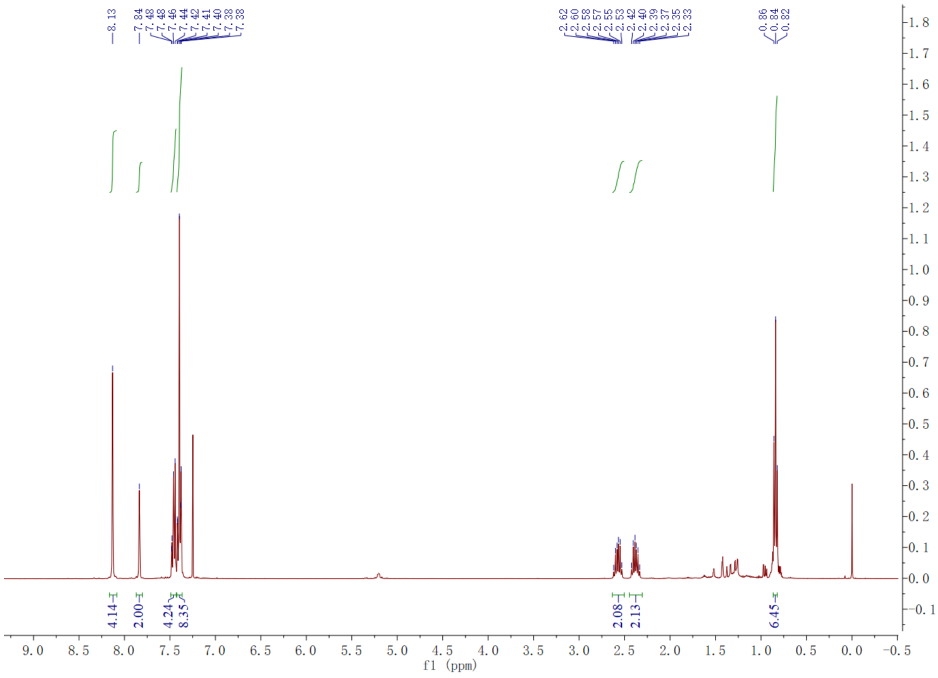

Figure S242. ^{1}**H NMR spectrum of (*S*)-CPA-3, Related to Figure 9a**

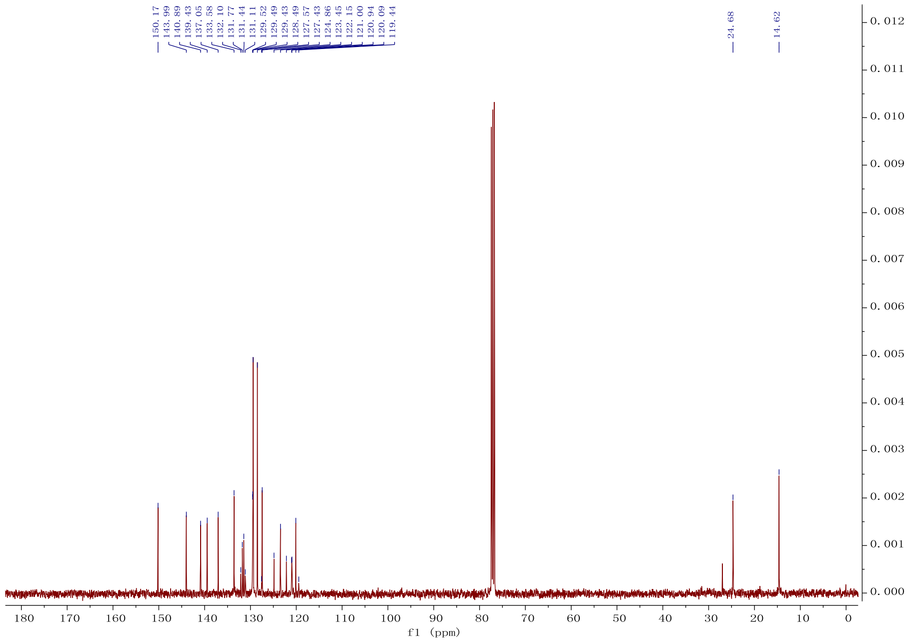

Figure S243. ^{13}**C NMR spectrum of (*S*)-CPA-3, Related to Figure 9a**

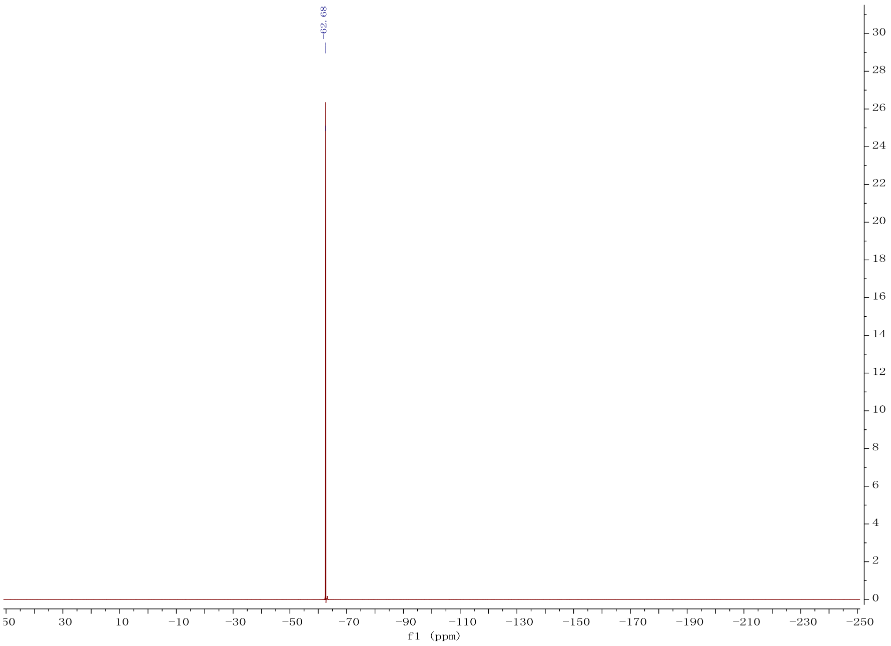

Figure S244. ^{19}F NMR spectrum of (*S*)-CPA-3, Related to Figure 9a

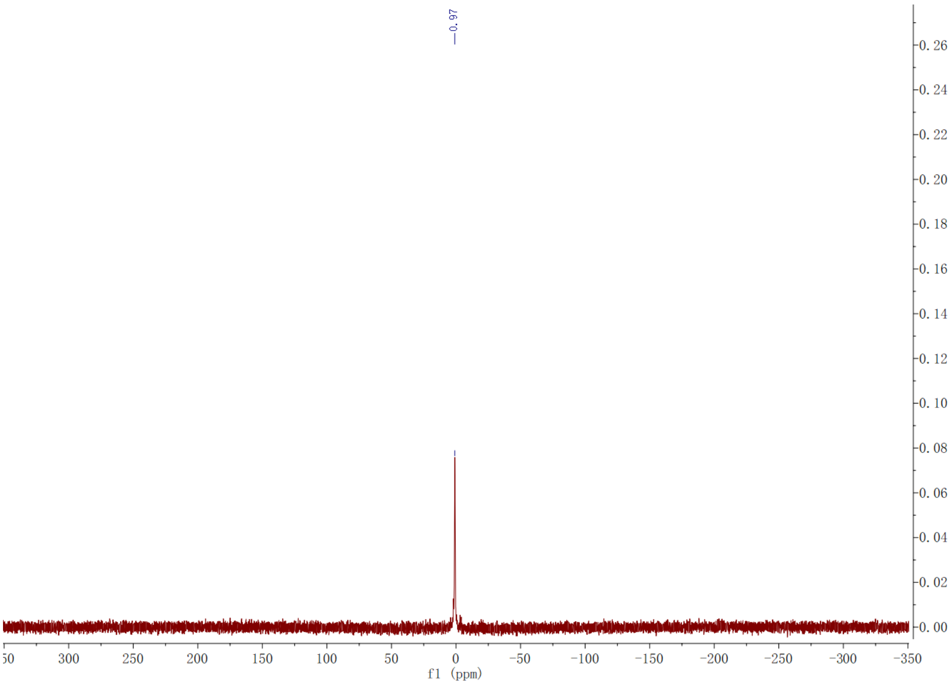

Figure S245. ^{31}P NMR spectrum of (*S*)-CPA-3, Related to Figure 9a

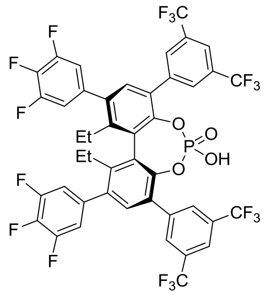

(*S*)-**CPA-4**

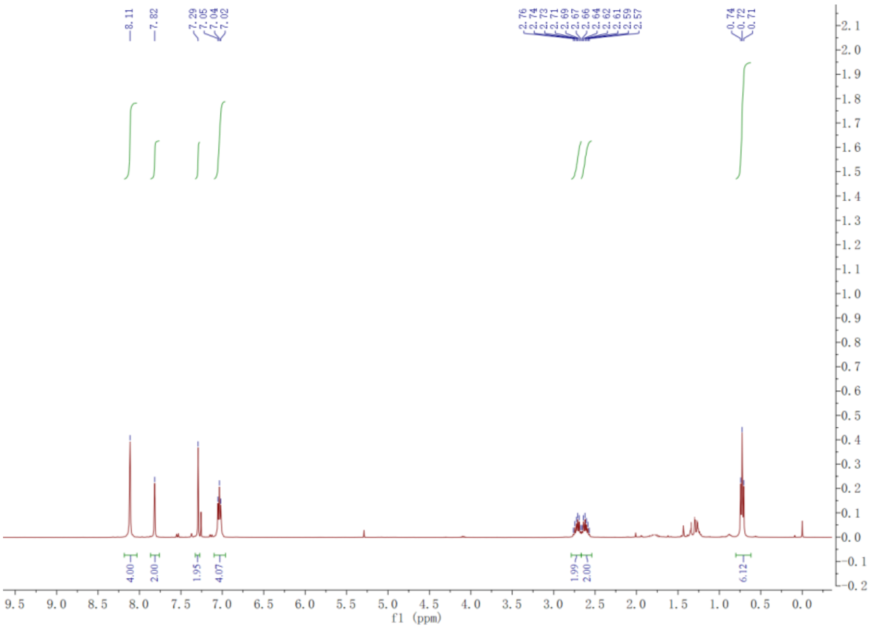

Figure S246. ^{1}H NMR spectrum of (*S*)-CPA-4, Related to Figure 9a

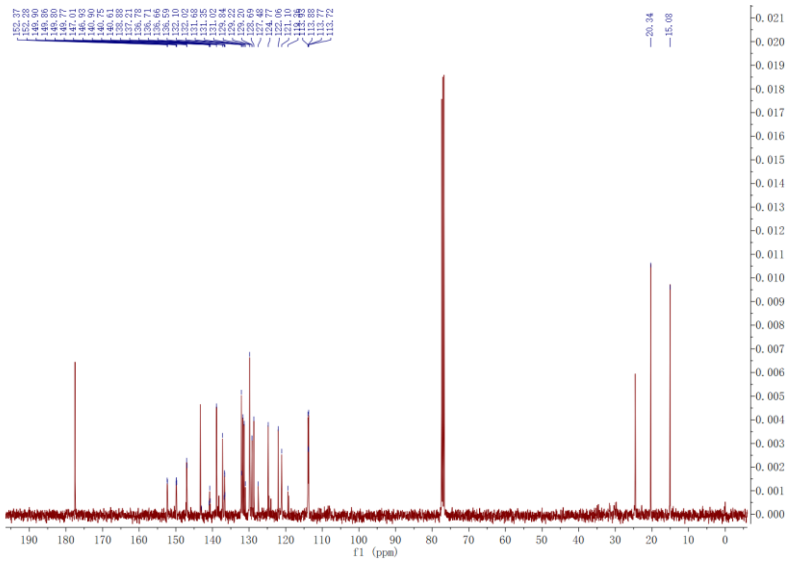

Figure S247. ^{13}C NMR spectrum of (*S*)-CPA-4, Related to Figure 9a

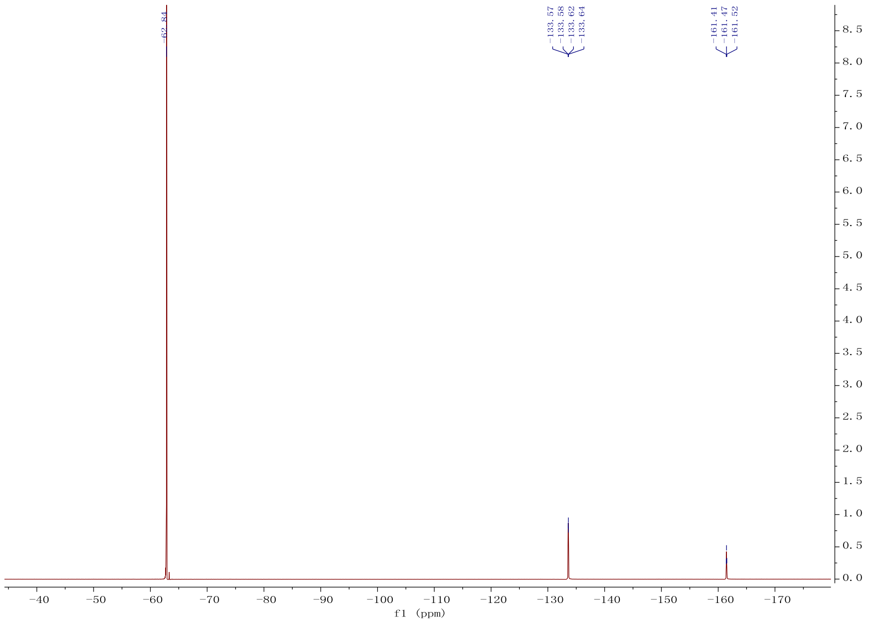

Figure S248. ^{19}F NMR spectrum of (*S*)-CPA-4, Related to Figure 9a

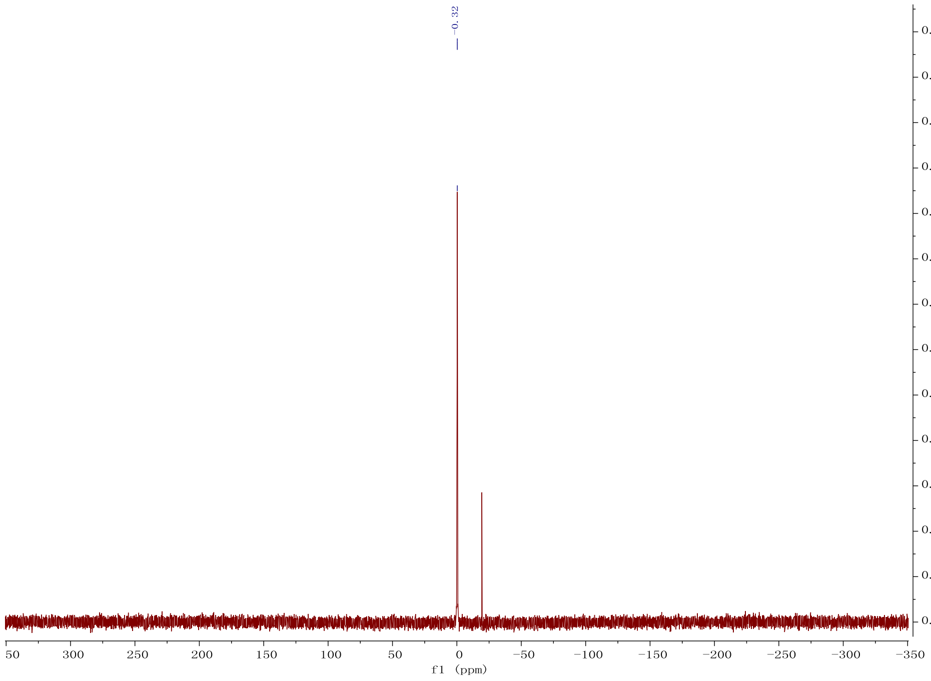

Figure S249. ^{31}P NMR spectrum of (*S*)-CPA-4, Related to Figure 9a

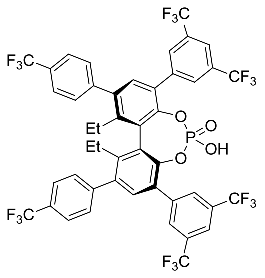

(*S*)-**CPA-5**

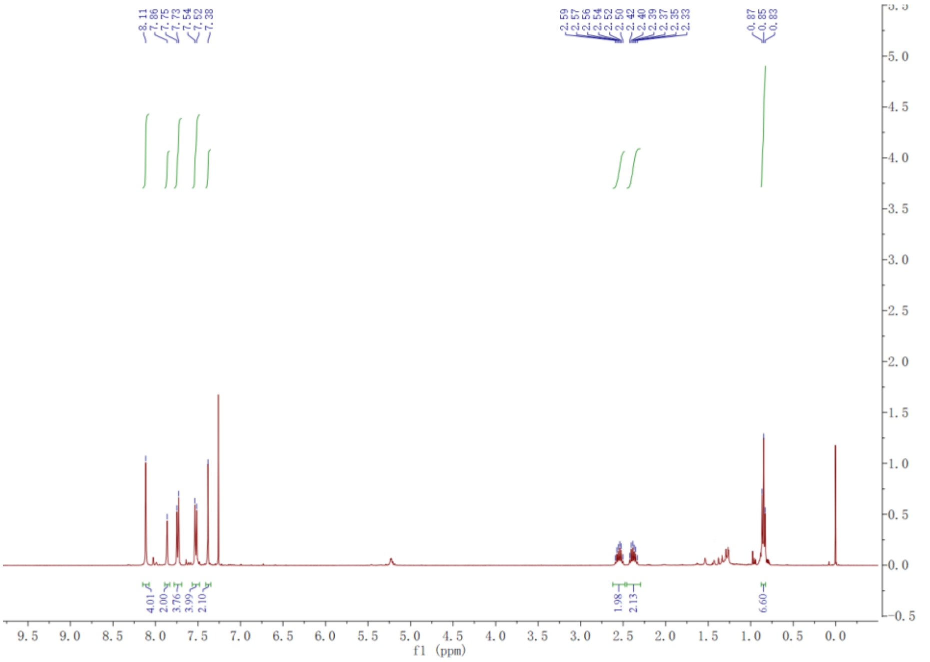

Figure S250. ^{1}H NMR spectrum of (*S*)-CPA-5, Related to Figure 9a

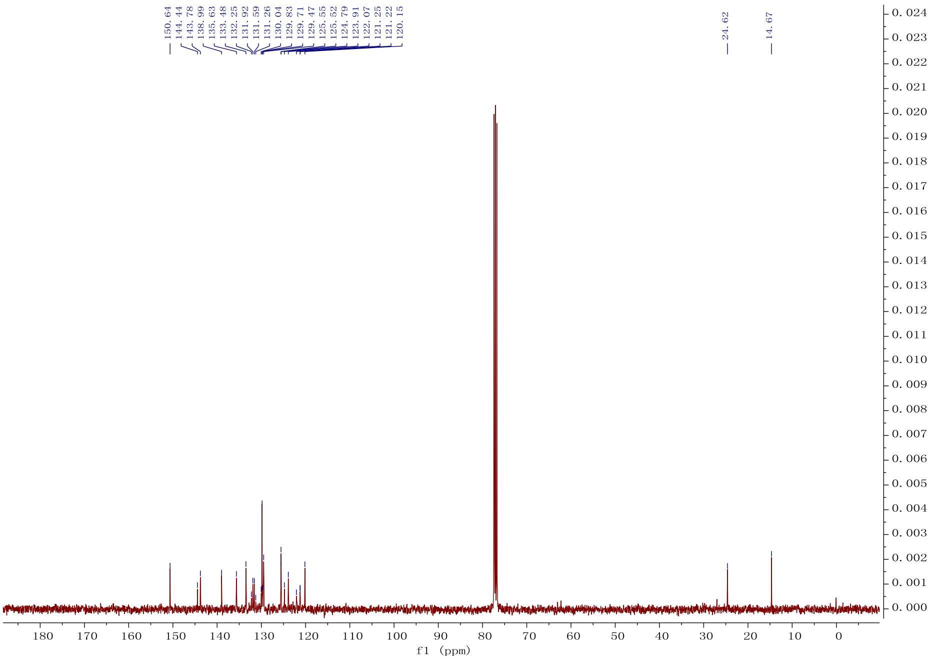

Figure S251. ^{13}C NMR spectrum of (*S*)-CPA-5, Related to Figure 9a

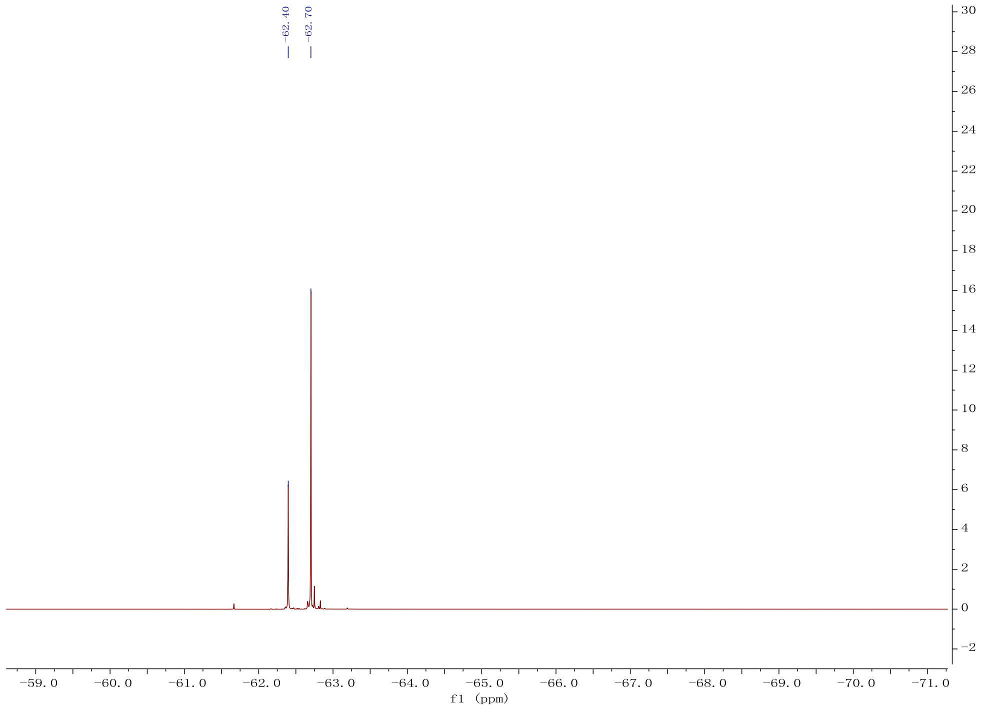

Figure S252. ^{19}F NMR spectrum of (*S*)-CPA-5, Related to Figure 9a

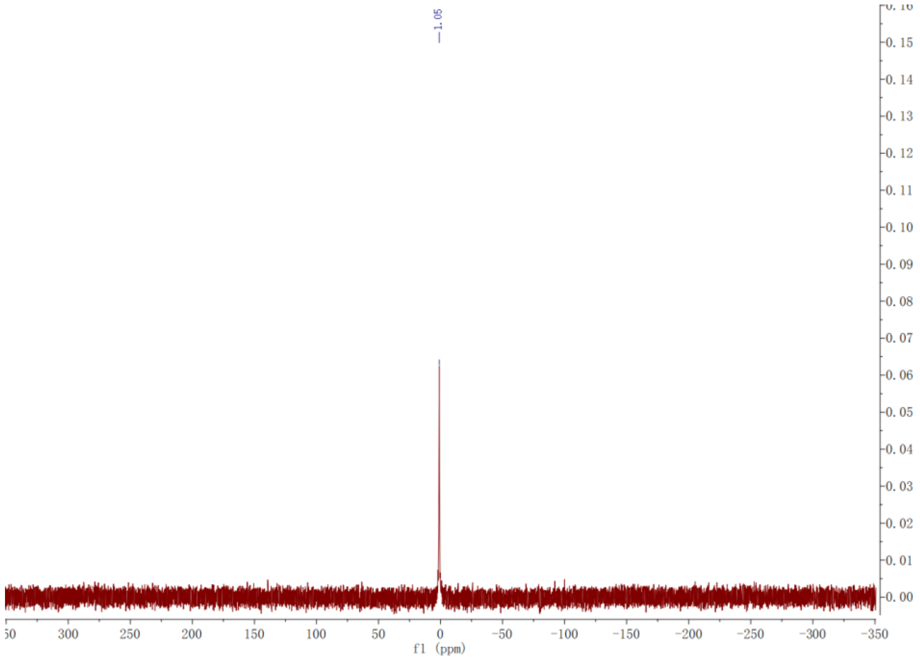

Figure S253. ^{31}P NMR spectrum of (*S*)-CPA-5, Related to Figure 9a

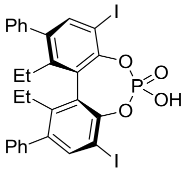

(*S*)-**CPA-1**

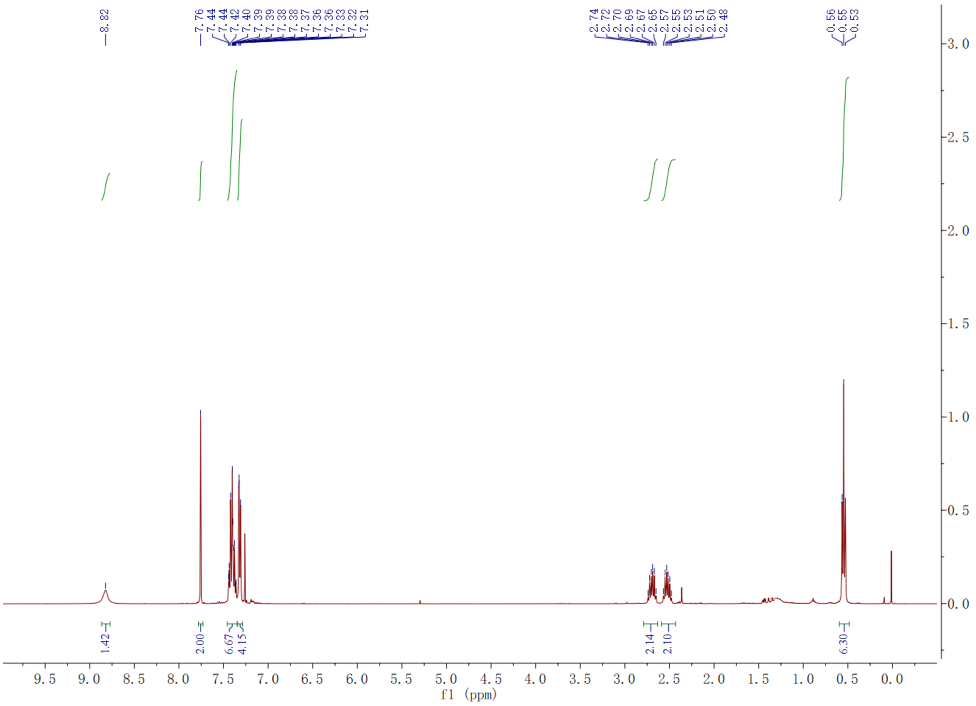

Figure S254. ^{1}H NMR spectrum of (*S*)-CPA-1, Related to Figure 9b

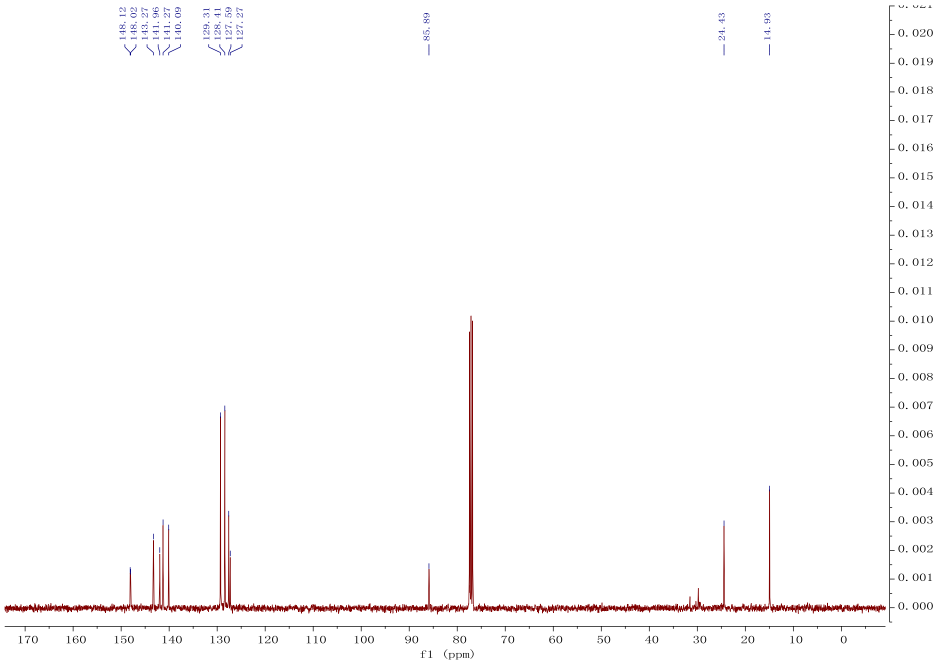

Figure S255. ^{13}C NMR spectrum of (*S*)-CPA-1, Related to Figure 9b

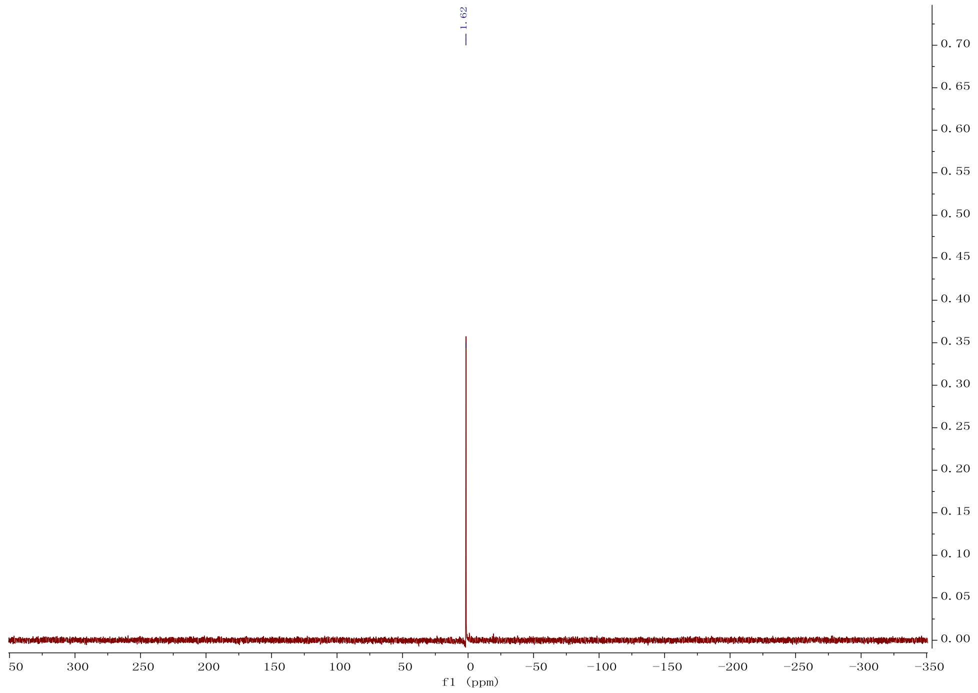

Figure S256. ^{31}**P NMR spectrum of (*S*)-CPA-1, Related to Figure 9b**

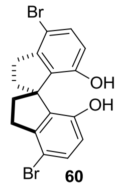

60

HPLC: enantiomeric excess of **60** (ee = 98%) was determined by high-performance liquid chromatography (HPLC) using a chiral stationary phase (AD-H column, flow rate = 1.0 mL/ min, eluent: hexane/ isopropanol = 85/15, 254 nm absorbance), retention times: minor enantiomer (t_R = 10.21 min), major enantiomer (t_R = 15.83 min).

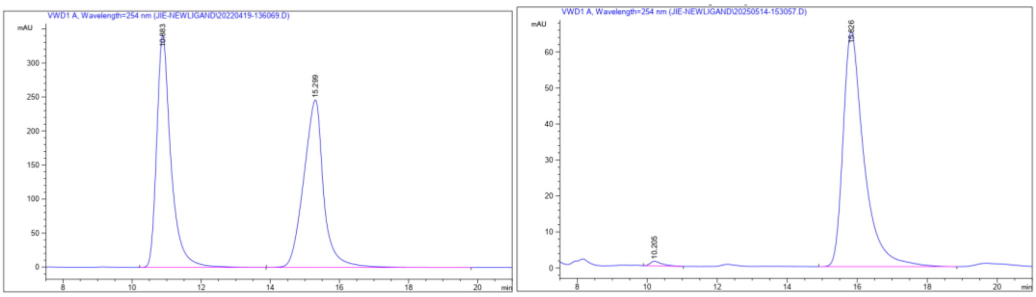

Figure S257. HPLC traces of 60, Related to Figure 10b

Signal 1: VWD1 A, Wavelength=254 nm

Peak #	RetTime [min]	Type	Width [min]	Area mAU *s	Height [mAU]	Area %
1	10.883	BB	0.4238	9749.66211	341.05734	49.8378
2	15.299	BB	0.5990	9813.13770	246.20227	50.1622
Totals :				1.95628e4	587.25961	

Signal 1: VWD1 A, Wavelength=254 nm

Peak #	RetTime [min]	Type	Width [min]	Area mAU *s	Height [mAU]	Area %
1	10.205	BB	0.3491	30.08746	1.27047	1.0986
2	15.826	BB	0.6126	2708.69141	65.22279	98.9014
Totals :				2738.77887	66.49326	

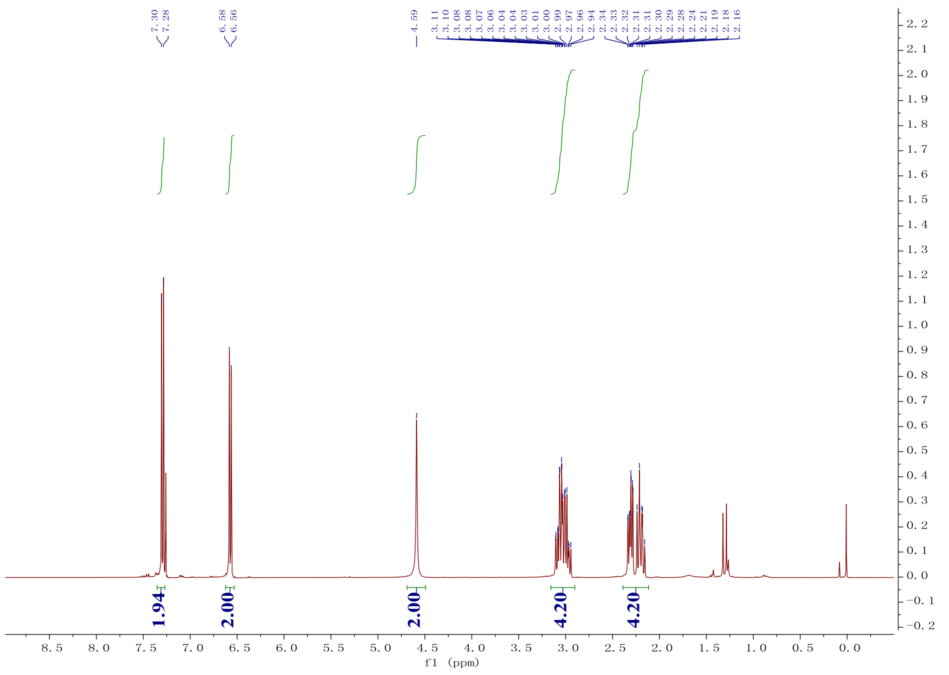

Figure S258. ^{1}H NMR spectrum of 60, Related to Figure 10b

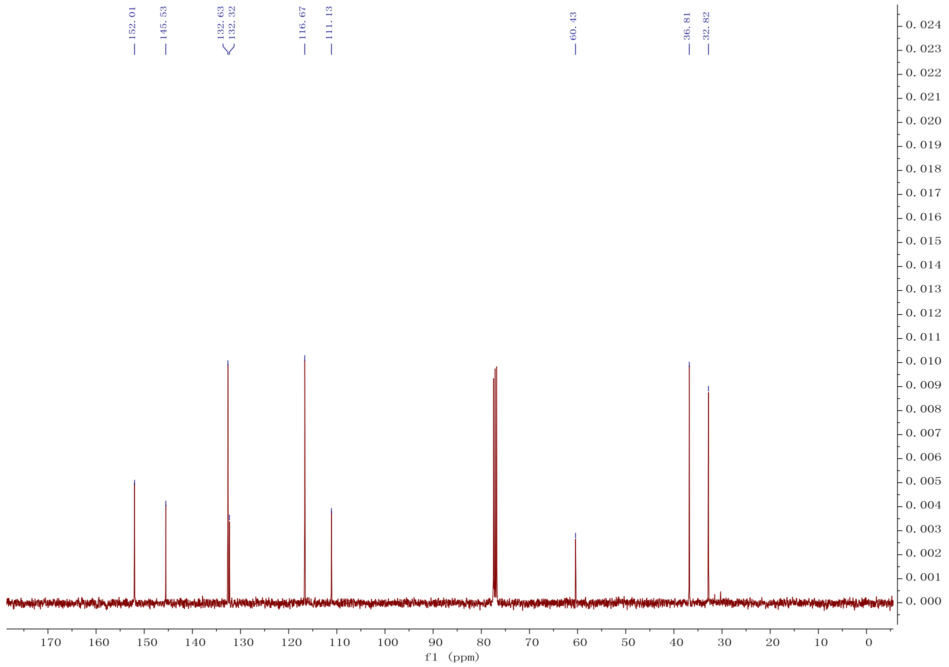

Figure S259. ^{13}C NMR spectrum of 60, Related to Figure 10b

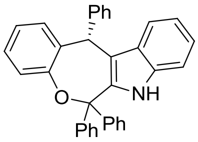

63

HPLC: enantiomeric excess of **63** (ee = 93%) was determined by high-performance liquid chromatography (HPLC) using a chiral stationary phase (AD-H column, flow rate = 1.0 mL/ min, eluent: hexane/ isopropanol = 90/10, 254 nm absorbance), retention times: minor enantiomer (t_R = 7.43 min), major enantiomer (t_R = 5.51 min).

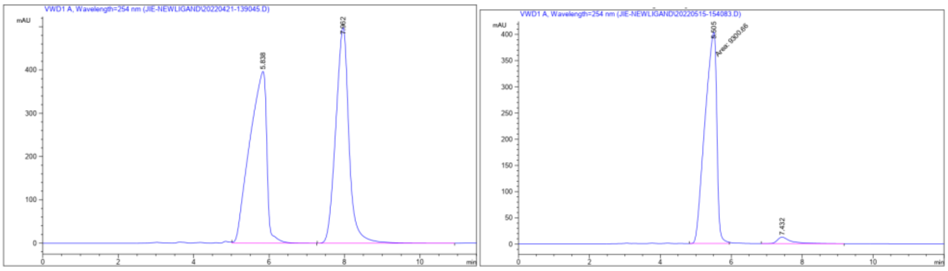

Figure S260. HPLC traces of 63, Related to Figure 10c

Signal 1: VWD1 A, Wavelength=254 nm

Peak #	RetTime [min]	Type	Width [min]	Area mAU *s	Height [mAU]	Area %
1	5.838	VB	0.5698	1.24342e4	396.37225	50.6079
2	7.962	BB	0.3713	1.21354e4	498.61105	49.3921
Totals :				2.45696e4	894.98331	

Signal 1: VWD1 A, Wavelength=254 nm

Peak #	RetTime [min]	Type	Width [min]	Area mAU *s	Height [mAU]	Area %
1	5.505	MM	0.3840	9300.66016	403.66016	96.5880
2	7.432	BB	0.3825	328.55179	12.25985	3.4120
Totals :				9629.21194	415.92001	

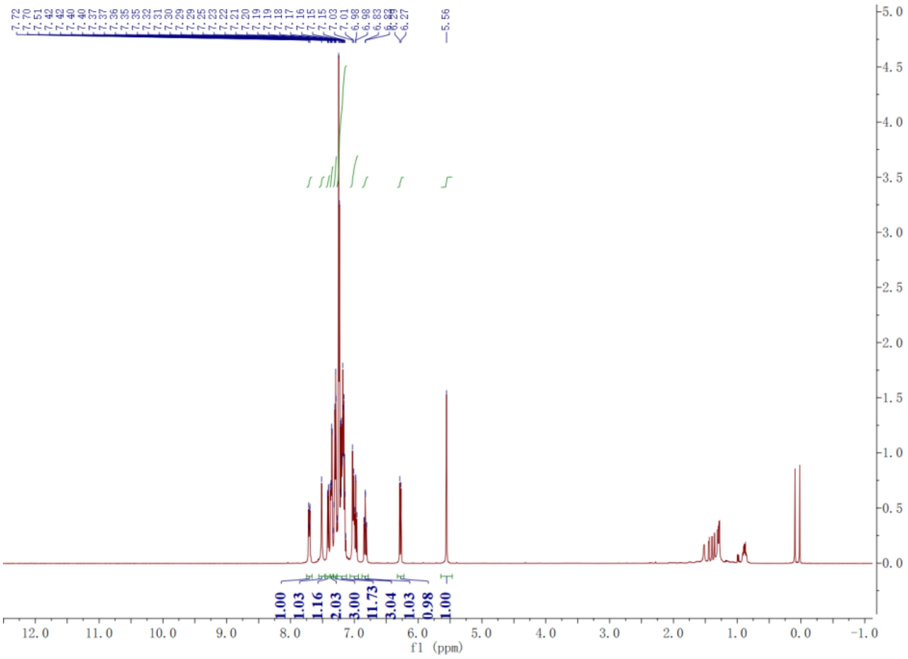

Figure S261. ^{1}H NMR spectrum of 63, Related to Figure 10c

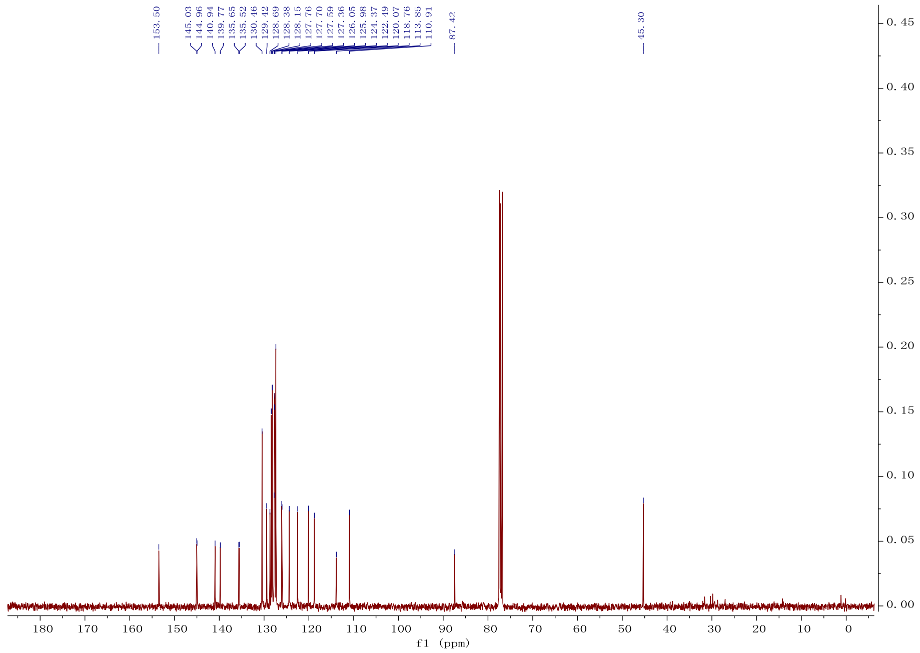

Figure S262. ^{13}C NMR spectrum of 63, Related to Figure 10c
